# Applied Behavior Analysis in Children and Youth with Autism Spectrum Disorders: A Scoping Review

**DOI:** 10.1007/s40614-022-00338-x

**Published:** 2022-05-18

**Authors:** Mojgan Gitimoghaddam, Natalia Chichkine, Laura McArthur, Sarabjit S. Sangha, Vivien Symington

**Affiliations:** 1 University of British Columbia Faculty of Medicine, Vancouver, British Columbia Canada; 2 Club Aviva Recreation Ltd., Coquitlam, British Columbia Canada; 3 University of Melbourne Faculty of Medicine, Dentistry and Health Sciences, Melbourne, Australia

**Keywords:** children and youth, neurodevelopmental disabilities and disorders, applied behavior analysis, autism spectrum disorder

## Abstract

This manuscript provides a comprehensive overview of the impact of applied behavior analysis (ABA) on children and youth with autism spectrum disorders (ASD). Seven online databases and identified systematic reviews were searched for published, peer-reviewed, English-language studies examining the impact of ABA on health outcomes. Measured outcomes were classified into eight categories: cognitive, language, social/communication, problem behavior, adaptive behavior, emotional, autism symptoms, and quality of life (QoL) outcomes. Improvements were observed across seven of the eight outcome measures. There were no included studies that measured subject QoL. Moreover, of 770 included study records, only 32 (4%) assessed ABA impact, had a comparison to a control or other intervention, and did not rely on mastery of specific skills to mark improvement. Results reinforce the need for large-scale prospective studies that compare ABA with other non-ABA interventions and include measurements of subject QoL to provide policy makers with valuable information on the impacts of ABA and other existing and emerging interventions.

**Supplementary Information:**

The online version contains supplementary material available at 10.1007/s40614-022-00338-x.

## Introduction

### Neurodevelopmental Disorders and Disabilities (NDD/D)

NDD/D consist of a range of diagnoses and functional impairments of a neurological origin that can present as functional deficits in developmental milestones such as language, communication, social skills, intellect, executive functioning, and motor development (American Psychiatric Association, 2013; Miller et al., 2013; World Health Organization [WHO], 2001, 2020). The prevalence of NDD/D across developed countries in children and youth 18 years of age and younger ranges from 8% to 15% (Arim et al., 2017; Boyle et al., 2011; Olusanya et al., 2018). Many different conditions and functional limitations are included within the scope of NDD/D, including autism spectrum disorders (ASD), attention deficit/hyperactivity disorder (ADHD), Down syndrome, and intellectual disabilities (ID). In particular, ASD has garnered much attention worldwide due to its high prevalence and associated socioeconomic and familial costs (Reichow et al., 2018).

ASD is a spectrum of diagnosable neurodevelopmental disorders that include pervasive developmental disorders (PDD), Asperger’s syndrome (AS) and autism. ASD typically presents during the developmental period and includes social communication and interaction difficulties, along with restricted and repetitive behaviors, interests, or activities (WHO, 2020). The prevalence of these disorders has increased over the past 20 years due to many combining factors. The global estimated prevalence in children and youth 18 years of age or younger is 0.62%–0.70% but could be as high as 1%–2% (Elsabbagh et al., 2012; Fombonne, 2009; Idring et al., 2012; Russell et al., 2014). The lifetime cost for families with a member diagnosed with ASD can range from approximately US$1.4 million in the United States and the United Kingdom, when diagnosed without an additional ID, to US$2.4million in the United States and US$2.2million in the United Kingdom if diagnosed concurrently with an ID (Buescher et al., 2014). Due to its increasing prevalence, the need for effective, evidence-based interventions for ASD has grown exponentially. Applied behavior analysis (ABA) and the interventions that are developed from its principles are some of the most often cited evidence-based interventions developed for the treatment of those diagnosed with ASD. As such, ASD will be the primary diagnosis of consideration within the current scoping review.

### Applied Behavior Analysis

At its core, ABA is the practice of utilizing the psychological principles of learning theory to enact change on the behaviors seen commonly in individuals diagnosed with ASD (Lovaas et al., 1974). Ole Ivar Lovaas produced a method based on the principles of B. F. Skinner’s theory of operant conditioning in the 1970s to help treat children diagnosed with ASD (or “autism” at the time) with the goal of altering their behaviors to improve their social interactions (Lovaas et al., 1973; Skinner, 1953; Smith & Eikeseth, 2011). To evaluate this method, the University of California at Los Angeles (UCLA) Young Autism Project model was developed and empirically tested by measuring the effects of the intervention when administered one-to-one to children diagnosed with ASD for 40 hr per week over the span of 2–3 years (Lovaas, 1987). The remarkable findings revealed that 47% of the children who participated in this treatment reached normal intellectual and educational functioning compared to only 2% of a control group (Lovaas, 1987).

ABA has evolved over the past 60 years from the core principles established in the early Lovaas model and subsequent UCLA Young Autism Project into many comprehensive treatment models and focused intervention practices, methods, and teaching strategies, all of which aim to address deficits for children and youth with ASD across all levels of functioning, including cognition, language, social skills, problem behavior, and daily living skills (Reichow et al., 2018). One notable and often cited foundational model is “antecedents, behavior, and consequences,” otherwise known as the ABC model, in which manipulating either or both the antecedents and consequences of behavior is intended to increase, decrease, or modify the behavior, thus resulting in a transferrable tool to target behaviors of interest effectively (Bijou et al., 1968; Dyer, 2013). There are also a number of techniques commonly associated with ABA that are worth noting, including reinforcement, extinction, prompting, video modeling, as well as the Picture Exchange Communication System (PECS), though many of these are widely used in other intervention and education settings (Granpeesheh et al., 2009; Sandbank et al., 2020; Stahmer et al., 2005).

Some specific comprehensive ABA-based treatment models that are investigated in this review include early intensive behavioral intervention (EIBI), Early Start Denver Model (ESDM), and Learning Experiences: An Alternative Program for Preschoolers and Their Parents (LEAP). EIBI is an intensive, comprehensive ABA-based treatment model for young children diagnosed with ASD. EIBI targets children under the age of 5 and is often administered 20–40 hr per week for multiple consecutive years (Matson & Smith, 2008; Reichow et al., 2018). It is conducted one-to-one in a structured setting such as in the home or school, and often utilizes the discrete trial training (DTT) method (Cohen et al., 2006; Smith, 2001) in conjunction with other, less structured teaching methods such as natural environment training (Granpeesheh et al., 2009). Because this is a comprehensive treatment model, the target of the intervention is across all aspects of functioning such as independent living skills, social skills, motor skills, pre-academic and academic skills, and language (Granpeesheh et al., 2009). Another comprehensive ABA-based treatment model is ESDM. This model was developed for children with ASD that fall within the age range of 12–60 months. This intervention builds upon the naturalistic teaching methods within ABA to provide a comprehensive, developmental, and relationship-based behavioral intervention targeted at children early in development (Dawson et al., 2010). More recently, some comprehensive ABA treatment models have further shifted away from intensive, operant conditioning based one-to-one models into more naturalistic and generalizable programming. LEAP is one such model for children with ASD because it takes place in public school settings (Strain & Bovey, 2011). LEAP was developed from fundamental principles of ABA and includes a variety of methods commonly used in ABA such as Pivotal Response Training (PRT), time delay and incidental teaching, in addition to utilizing peer-mediated interventions and the PECS (Strain & Bovey, 2011). It is significant that a core principle of LEAP is to strongly emphasize parental and peer involvement with respect to teaching behavioral strategies and relies on naturally occurring, incidental teaching arrangements, in contrast to the directional, adult-driven instruction used in most other segregated ABA intervention strategies (Hoyson et al., 1984; Strain & Bovey, 2011).

Within these comprehensive treatment models, focused intervention practices that are often utilized and independently investigated can include, but are not limited to, DTT and naturalistic teaching strategies such as PRT and functional communication training (FCT). DTT is one of the most fundamental focused intervention practices of ABA and utilizes sequences of instruction and repetition in a distraction free, one-to-one setting (Smith, 2001). The primary focus of DTT is to teach children new behaviors and discriminations. These new behaviors encompass any behavior that was not previously performed by the child knowingly or unknowingly (Smith, 2001). Naturalistic teaching forms of ABA have sought to improve the ability to generalize and maintain the positive effects of behavioral interventions while upholding many of the fundamental principles and behaviorism of ABA (Schreibman et al., 2015). One such method of naturalistic teaching is through the focused intervention practice of PRT, developed by Koegel and Koegel (2006), which is focused on improving the self-initiative and motivation of a child to communicate effectively in common real-life settings (Mohammadzaheri et al., 2015). Of note, most of these treatments can involve a professional, though many of the more recent studies and iterations of these treatments seek to involve peers, siblings and family members to encourage generalization to real-world settings and people in the child’s personal life (Mohammadzaheri et al., 2015; Steiner et al., 2012). Another focused intervention practice and naturalistic teaching method is FCT, a differential reinforcement-based procedure developed by Carr and Durand (1985) that reduces problem behaviors by replacing them with more appropriate communicative responses. This training is commonly used in conjunction with other ABA methods.

Given the history and range in interventions, there is a degree of variability and confusion in the definition of ABA as a system. Definitions range from rigid protocols for some ABA-based programs to collections of specific techniques associated with ABA, to ABA as a system to evaluate practices rather than as an intervention itself. Granpeesheh et al. (2009) define ABA as “the application of principles of learning and motivation to the solution of problems of social significance” (p. 163). This definition of ABA as a research strategy echoes that of Baer et al. (1968) through the later 20th century, in particular in terms of behavior study being: (1) applied, (2) behavioral, (3) analytic, (4) technological, (5) conceptually systematic, (6) effective, and (7) capable of generalized outcomes. Agency definitions tend to define it as a therapy, likewise noted by Schreibman et al. (2015), with different approaches listed as types. For instance, the Centers for Disease Control and Prevention (CDC) defines ABA as a treatment approach, with examples such as DTT, EIBI, ESDM, PRT, and verbal behavior intervention (VBI; CDC & National Center on Birth Defects & Developmental Disabilities, 2019). The National Institute of Child Health and Human Development (NIH) lists positive behavioral support (PBS), PRT, EIBI, and DTT as types of ABA (Eunice Kennedy Shriver National Institute of Child Health & Human Development, 2021). The Autism Society(n.d.) follows the same definition as Baer et al., whereas other intervention types such as PRT and extinction are described as ABA procedures or as sharing principles of ABA. Many ABA-derived programs define certain expectations of their practices specifically, such as EIBI setting, intensity, duration, and personnel, although their methods list a variety of techniques deemed ABA-based, such as DTT, precision teaching, and incidental teaching. As combined approaches become more common, it is becoming more difficult to differentiate interventions considered to be ABA-derived from other non-ABA labeled interventions (Smith, 2012).

All of the research into these methods, programs, and comprehensive models, combined with the continued investigations into the traditional applications of the ABA-based interventions, results in a wealth of research about the impact of ABA on children and youth with ASD, in particular with respect to improvements in cognitive measures, language skills, and adaptive skills (Eldevik et al., 2009; Virués-Ortega, 2010). The ensuing amount of scientific evidence has resulted in ABA being considered a “best practice” and thus endorsed by the governments of Canada and the United States for the treatment of children and youth with ASD (Government of Canada, 2018; U.S. Department of Health & Human Services, 1999).

### Rationale for Current Scoping Review

As ABA is a broad intervention which includes many different methods and programs, reviews of the entire scope of the current research are uncommon. To our knowledge, a comprehensive review of the current ABA literature that spans all ABA methods and outcomes for children and youth with ASD, and that includes randomized controlled trials (RCT), clinical controlled trials (CCT), and single-case experimental design (SCED) studies, has not been completed. The current literature consists primarily of systematic reviews and meta-analyses that have investigated the quantifiable and qualitative outcomes of ABA on children with ASD, but few of these studies include SCED, and the results across the reviews inconsistently show significant improvement with ABA interventions.

For example, in a meta-analysis by Virués-Ortega (2010), the effectiveness of ABA was investigated across 22 included studies with respect to as many outcomes as possible, including language development, social functioning, intellectual functioning, and daily living skills, for those diagnosed with ASD (Virués-Ortega, 2010). The results of this meta-analysis suggested that ABA interventions that were implemented in early childhood and were long-term and comprehensive in design did result in a positive medium to large effect in the areas of language development (pooled effect size of 1.48 for receptive language, 1.47 for expressive language), intellectual functioning (pooled effect size 1.19), acquisition of daily living skills (pooled effect size 0.62), and social functioning (pooled effect size 0.95), when compared to a control group that did not receive ABA intervention. This mirrors the meta-analysis of 29 articles conducted by Makrygianni et al. (2018), where it was found that ABA programs for children with ASD resulted in moderate to very effective improvements in expressive and receptive language skills, communication skills, nonverbal IQ scores, total adaptive behavior, and socialization, but lesser improvements in daily living skills. In a 2018 meta-analysis by Reichow et al. (2018), the changes in autism severity, functional behaviors and skills, intelligence, and communication skills were investigated across five articles that included one RCT and four CCTs for EIBI. After conducting meta-analyses of these studies, it was found that the evidence for EIBI improving adaptive behavior compared to treatment as usual comparison groups was positive but weak (mean difference [*MD*] = 9.58; 95% confidence interval (*CI*) 5.57–13.60), whereas there was no evidence that EIBI improved autism symptom severity (standardized mean difference [*SMD*] = −0.34; 95% *CI* −0.79–0.11; Reichow et al., 2018). Therefore, the current literature appears to indicate inconsistent results with respect to the magnitude of improvements seen as a result of ABA interventions for children and youth with ASD.

With respect to the wealth of SCEDs included throughout the ABA literature, Wong et al. (2013) have noted that existing reviews rarely capture these types of studies, with two notable exceptions conducted by the National Autism Center (2009) and the National Professional Development Center on ASD (NPDC; Odom et al., 2010). These studies still had some key exclusions: the National Autism report excluded articles that (1) did not have statistical analyses, (2) did not include linear graphical presentation of the data for SCEDs, or (3) used qualitative methods, whereas the NPDC report searched for studies on behavioral strategies that fulfilled the requirements of being an evidence-based practice, as defined by the authors (National Autism Center, 2009, 2015; Odom et al., 2010). Neither of these reports evaluated the entire scope of the available ABA research with respect to children and youth with ASD, potentially missing the value of the studies that were excluded.

The purpose of the current review therefore is to evaluate the available literature on ABA as an intervention approach in the treatment of ASD in children and youth in an effort to help instruct the scientific community on the most beneficial directions for future research. Moreover, as ABA is commonly recognized at a governmental level as evidence-based, a review of the current ABA literature will help inform other existing and emerging therapies and interventions, researchers, policy makers, and the public of the standard to which established, evidence-based interventions are held. This is accomplished by collecting, compiling, and discussing the available data on the most common outcomes and methods. This includes the most common journals of publication, population metrics, and the transferability of this prominent therapy approach to the real world. As such, the objectives of this scoping review are to examine the extent, range, and nature of research activities regarding the impact of ABA on children and youth with ASD and to identify any gaps in the existing literature regarding ABA outcomes and research designs.

## Methods

A scoping review study design was selected for the current investigation. According to Colquhoun et al. (2014), “a scoping review is a form of knowledge synthesis that addresses an exploratory research question aimed at mapping key concepts, types of evidence, and gaps in research related to a defined area or field by systematically searching, selecting, and synthesizing existing knowledge” (p. 1293). Scoping reviews differ from systematic reviews in that they provide an overview of existing evidence regardless of the quality (Tricco et al., 2016), and may not formally assess study rigor (Arksey & O’Malley, 2005).

The current scoping review was conducted to gather an understanding of the scope of available research regarding the use of ABA as an intervention for children and youth living with NDD/D, and in particular ASD. For the purposes of the current review, ABA will be defined as an intervention informed and developed from behavioral analytic approaches for the treatment of children and youth with ASD. The effect of ABA is defined as the measurable changes in a participant's various outcomes as a result of receiving ABA intervention. These outcomes were not predefined to prevent missing any possible impact. The review comprised a database search, as well as a reference search of selected reviews. A second phase of the literature search was conducted to update the sample to reflect more recent literature. A guiding document by Tricco et al. (2016) was used for direction and as a reference for conducting this review.

### Search Strategy

An initial search was conducted across PubMed, MEDLINE (EBSCOHost), Cumulative Index to Nursing and Allied Health Literature (CINAHL), PsychINFO, Educational Resources Information Center (ERIC), Cochrane Central Register of Controlled Trials (CENTRAL), and Cochrane Database of Systematic Reviews (CDSR) utilizing medical subject heading (MeSH) search terms and limitations to describe the relevant population in the initial search (children and youth with NDD/D) and intervention (ABA) (see Appendix 1 for a full list of search terms for each database). Additional limitations of the search were English language publications, subject age range of 0–18 years, and publication date range. The search was conducted in two phases: January 1, 1997 through December 31, 2017, and January 1, 2018 through December 31, 2020.

Several reviews were selected for a further text search. Data were not extracted directly from eligible reviews. Instead, their selected articles were screened and added to the sample if they were not already included in the initial search. This process was repeated for any secondary reviews that occurred as well. These additions were excluded from the publication date limitation, resulting in the inclusion of a number of studies outside of the initial search date range. Review and meta-analysis results were not coded.

### Selection Criteria

A PICO (population, intervention, comparison, outcome) framework was used to guide the selection of articles. Population and intervention were used as eligibility criteria. Although the intervention was restricted to ABA, the population was originally defined broadly as NDD/D in an effort to capture as much of the applicable literature as possible, and later revised to focus on ASD and mixed diagnoses (ASD and other). This included populations where some subjects had other non-ASD diagnoses, such as ADHD, Down syndrome, or ID, whether they co-occurred with ASD within subjects or presented across subjects. Non-ASD diagnoses observed in the mixed-diagnoses category of the current review are described in the results (“Results: Description of Included Studies”) and in Appendix 2. Outcome was not considered because one objective of the current scoping review was to identify the measured outcomes. Comparison was not used so as not to limit the scope of the review. Study design was not limited in the initial search.

Inclusion criteria for article selection during the initial search comprised (1) English language articles that are (2) about the effects of ABA on (3) children and youth (birth to 18 years) with NDD/D, within (4) the timeframe of January 1, 1997 through December 31, 2020. As described above, screened articles included from selected reviews and secondary reviews were exempt from the date range limitations.

Exclusion criteria comprised (1) hospital-based (inpatient) settings and mixed-setting studies (i.e., those including some inpatient subjects); (2) use of qualitative research methods; (3) publications that are not “research-based” (e.g., newsletters, books); (4) populations exceeding 18 years of age; and (5) combined interventions if not looking specifically at the effectiveness of ABA intervention. In cases of mixed age (i.e. including subjects over 18 years of age) or mixed population (i.e., including typically developing subjects), studies were excluded if it was not possible to extract results for the target population separately. Inpatient settings were excluded because the focus of the current scoping review was on community offerings, not hospital services. A small number of studies were excluded when the methods did not align with typical ABA outcome measures, such as those training response hierarchies or attempting to condition new reinforcers. A library search was conducted for studies that could not be accessed in full online, and any that could not be found were subsequently excluded.

When the diagnostic criteria were narrowed to focus primarily on ASD, articles that contained only non-ASD diagnoses were excluded.

### Screen Process and Study Selection

Articles from the original search of online databases were exported to Mendeley® Desktop versions 1.19–2.62.0, a reference management software, where most duplicate studies were automatically identified and removed. Any remaining duplicates from both the database and review search were removed manually. Titles and abstracts of all retrieved articles were then independently reviewed by two researchers following the outlined inclusion and exclusion criteria. Studies were included if the independent reviewers reached agreement, or after further discussion with a third reviewer. Retained articles then underwent full text review for inclusion, following the same steps.

### Data Extraction

Articles included following the full text review then underwent data extraction. Extracted data comprised first author, title, year of publication, origin of study, funding sources, study aim, study design, duration of intervention, duration of study, population size, population description, setting, measurement outcomes, measurement tools, and key findings. In cases where results were reported individually for each subject, they were extracted as such. In larger scale studies where only group results were reported, group results were extracted, so long as the group included only the target population.

### Data Coding and Synthesis

#### Coding

**Fig. 1:**
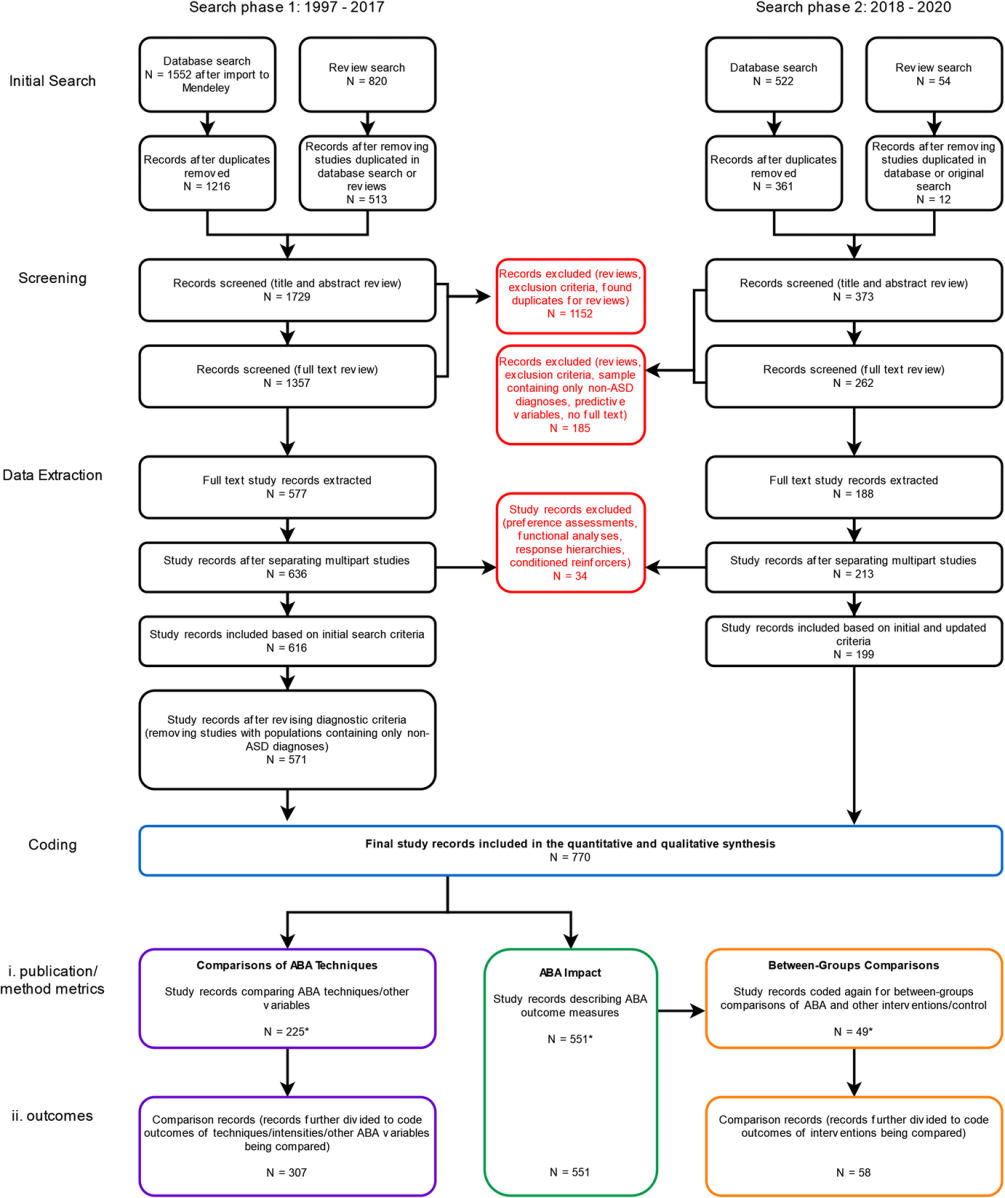
Flowchart Describing the Process of the Current Scoping Review Search, Screening, Data Extraction, and Coding. *Note.* From an initial search comprising 2,948 records, after screening studies and subdividing multipart studies, a total of 770 study records remained. These were coded in three categories: Comparisons of ABA Techniques, ABA Impact, and Between-Groups Comparisons. Designed with reference to Tricco et al. (2016) and created using diagrams.net™/draw.io® from JGraph Ltd. Note that three study records were included in both the ABA Impact section and the Comparisons of ABA Techniques section (Mello et al., 2018; Rad et al., 2019; Vietze & Lax, 2020), and three study records were included in all three coding sections (Dugan, 2006; Kalgotra et al., 2019; Kovshoff et al., 2011).

In general, the entire sample of records included for coding and synthesis was subdivided into three sections concerned with: (1) general ABA Impact, (2) Comparisons of ABA Techniques, and (3) Between-Groups Comparisons of ABA to control or other interventions. These divisions are visually summarized in Figure 1 and are described below. All records underwent general data coding of basic study information, as well as specific outcome coding, also described below. (Details about coding definitions can be found in Appendix 2.) Simplified extraction tables for these three subdivisions are available in Appendix 3 (Tables S1, S2, and S3).

During the process of coding, articles containing multiple concurrent or consecutive studies were separated into discrete rows, and will hereafter be treated as self-contained studies in this review. In all figures and further text, all coded rows are referred to as “study records.” Once separated, researchers identified and excluded (1) functional analyses or studies focused on their use, (2) preference assessments or studies focused on their use, and (3) predictive studies. Study records were coded independently by two researchers and then discussed to obtain agreement, or referred to a third researcher to obtain agreement. During coding, any further study records found to satisfy the exclusion criteria were excluded.

Items selected for general data coding included publication details, population metrics, and several specific study methods. The population metrics were age, sex, and diagnosis of participants. (Detail on the population coding values can be found in Appendix 2). Study records were additionally coded and compared by two independent researchers to identify inclusion of the following methods: (1) follow-up or maintenance, (2) mastery or criterion measures, (3) generalization. Studies including comparison groups were further coded by one researcher to identify the presence of (1) a control group (typically consisting of “eclectic” or treatment as usual), (2) comparisons to other non-ABA intervention/s, or (3) a mix of these.

After general data coding, the sample was separated into two groups for outcome coding: ABA Impact and Comparisons of ABA Techniques. The majority of study records fell into the ABA Impact section, in which study records measured the change in outcomes (e.g., amount improved) as a result of exposure to ABA intervention. In contrast, study records that were primarily concerned with comparing multiple techniques or intensities of ABA were reserved for the Comparisons of ABA Techniques section, because general ABA impact could not easily be determined for the entire study population in these studies. Finally, a select number of study records from the ABA Impact section where ABA interventions were also compared to a control or different intervention were coded a second time to describe these comparisons in the Between-Groups Comparisons section. As noted in Fig. 1, some studies from the ABA Impact section also fell into the Comparisons of ABA Techniques section, or into all three sections.

Although the search was not restricted, the observed outcome measures were classified into eight categories: cognitive, language, social/communication, problem behavior, adaptive behavior, emotional, autism symptoms, and quality of life (QoL) outcomes. At first, QoL was included to help describe the generalizability and real-life utility of ABA interventions, following the example of Reichow et al. (2018). However, as no instances of subject QoL measures occurred in this search, this outcome is not included in the subsequent synthesis. Within each category, outcomes were generally classified as improvement, regression, mix, or no change, as can be seen in the extraction tables (Tables S1, S2, and S3 in Appendix 3).

When more than two variables or interventions were compared, which sometimes occurred in the Comparisons of ABA Techniques and Between-Groups Comparison sections, study records were discussed and split into discrete rows by two researchers to represent simplified or single-variable comparisons in each row. These are termed “comparison records” for the purpose of coding and synthesis. As seen in Tables S2 and S3 in Appendix 3, further detail was extracted regarding the category of techniques or interventions compared and the relative effectiveness of each.

Prior to coding, researchers categorized outcome measures, measurement scales or strategies, and intervention categories observed during the extraction process into tables in an effort to mitigate potential inconsistencies in coding. For example, in the Comparisons of ABA Techniques section, categories were broadly defined as Teaching, Stimulus Characteristics, Reinforcement, Subject/Setting Characteristics, and Comparisons of ABA Interventions. Further descriptions of these and other categories can be found in Appendix 2.

Further details on general data coding, as well as outcome coding for ABA Impact, Comparisons of ABA Techniques, and Between-Groups Comparisons can be found in Appendix 2. Extractions for all three sections can be found in Tables S1, S2, and S3, respectively, in Appendix 3.

#### Synthesis

All statistical analyses, compilations, and tabulations were completed using Microsoft® Excel® versions 1805-2111. Descriptive analyses (means, medians, etc.) were calculated using native Excel® functions. Pivot tables were utilized to tabulate frequencies. Figures were generated using Microsoft® Excel® version 2016 MSO, Microsoft® Word® versions 2011–2111, and diagrams.net™/draw.io® by JGraph Ltd.

In addition, some qualitative characteristics were explored as well, such as observations about the types of methods used in the interventions encountered, the degree of mastery and generalization measures, and how targeted the interventions and measurement tools were.

## Results

### Identified Studies

As shown in Fig. 1, the record selection process differed slightly between the two searches spanning 1997–2017 and 2018–2020. This is because the diagnostic criteria for the current manuscript were updated to exclude populations that only contained non-ASD diagnoses, and the removal of records satisfying the new criteria took place at different points for each search.

The database searches yielded a total of 2,074 entries after import to Mendeley®, and 874 entries from selected reviews and secondary reviews. Ten systematic reviews were identified and investigated for the literature search (Brunner & Seung, 2009; Dawson & Bernier, 2013; Makrygianni et al., 2018; Mohammadzaheri et al., 2015; Reichow et al., 2014, 2018; Rodgers et al., 2020; Shabani & Lam, 2013; Spreckley & Boyd, 2009; Virués-Ortega, 2010). After pulling references from the first five (Brunner & Seung, 2009; Dawson & Bernier, 2013; Makrygianni et al., 2018; Rodgers et al., 2020; Shabani & Lam, 2013), it was found that the references in the remaining five reviews were duplicates of previously identified references. Secondary reviews from Seida et al. (2009) and Dawson and Burner (2011), both cited by Dawson and Bernier (2013), were also investigated for references (Bassett et al., 2000; Bellini & Akullian, 2007; Delano, 2007; Diggle et al., 2002; Horner et al., 2002; Hwang & Hughes, 2000; Lee et al., 2007; McConachie & Diggle, 2007; Odom et al., 2003; Reichow & Volkmar, 2010; Smith, 1999). Records from Brunner and Seung (2009) that were categorized into treatment models that did not fulfill the definition of ABA as per the current review were not considered. In addition, the secondary review by Vismara and Rogers (2010) was not considered because it was a narrative review. After removing duplicates or entries already existing in the database search, 1,577 entries remained from the database search and 525 from reviews, for a total of 2,102 records.

A total of 1,337 records were removed during title, abstract, and full-text screening because they met the exclusion criteria, were duplicate records, were reviews, or contained only non-ASD diagnoses. Multipart studies were separated into discrete records, yielding a total of 849 study records. A further 34 were excluded at this stage as they were preference assessments, functional analyses, or were concerned with training response hierarchies or conditioning reinforcers, leaving 815 study records. When the diagnostic inclusion criteria were revised, any remaining records containing only non-ASD diagnoses were excluded.

Thus, the total sample included in the quantitative and qualitative synthesis comprised 770 study records. This entire sample was analyzed for general data metrics (see Fig. 1). References for the 709 included articles can be found in Appendix 4.

### Description of Included Studies

Overall, agreement between raters was approximately 80% across all coding categories. The range of included outcome categories was selected in order not to limit the scope of the literature search and synthesis for this review so that a comprehensive review of the application of ABA for ASD and mixed-diagnosis populations across the entire time span and age range of the search could be conducted. Frequently occurring other diagnoses in the mixed-diagnoses category included ADHD; ID; global developmental delay (GDD) or other developmental delays; oppositional defiant disorder (ODD); Down syndrome; cerebral palsy (CP); fetal alcohol spectrum disorders (FASD); Angelman syndrome; Fragile X; obsessive-compulsive disorder (OCD); Tourette syndrome; traumatic brain injury (TBI); epilepsy or seizure disorders; sensory integration or processing disorders; speech/language delays; learning disabilities; and behavior, emotional, or mood disorders.

The most frequently occurring publication year was 2020. The earliest publication reviewed was from 1977 and the most recent from 2020. Thirty percent were from 2000–2009 and 61% were from 2010–2020. The remaining years comprised 9% of the journals reviewed.

The 5-year impact factor (IF) characteristics were determined by removing duplicate journals prior to calculation. IFs were accessed from Journal Citation Reports, via Clarivate™. The unique median IF was 2.56. The lowest impact journal had an IF of 0.71 and the highest had an IF of 9.92. Most of the reviewed study records were from the *Journal of Applied Behavior Analysis* (55%). The next most frequent journal was the *Journal of Autism and Developmental Disorders*, representing 4% of the journal cohort. Dissertations accounted for 4% of the cohort. *Analysis of Verbal Behavior* and *Behavioral Interventions* each made up 3% of our journal cohort, and the remaining journals contributed 1%–2% each. Journals contributing less than 1% were grouped as “Other,” making up 16% of the total cohort. Within the cohort of study records, 48% of records had participants that were solely male, 45% were of mixed sex, and 4% of the publications had solely female participants. Seventy-six percent of study records had participants with only ASD, and 24% had participants in the mixed-diagnoses category.

In the study records reviewed, 33% had one or two participants, whereas 31% of the publications had three participants, and 13% had four. Study records with 5 to 9 participants accounted for 11% of the total and 13% had more than 10 participants. The median number of participants was 3, whereas the mean number of participants was 8.12.

Overall, it was found that study records that included a smaller sample size (e.g., *N* ≤ 4) often investigated specific skills, tasks, or responses that varied based on each specific child (Gongola, 2009; Plavnick & Ferreri, 2011; Sullivan et al., 2020). Many studies modified the intervention or the definition of mastery dependent on the child or task given (Charlop-Christy & Daneshvar, 2003; Charlop et al., 1985; Ezzeddine et al., 2020; Lyons et al., 2007; Romaniuk et al., 2002).

Within the cohort of study records, 41% had some follow-up measure, 40% had some criterion or mastery measure, and 31% of publications had some generalization measure.

### Study Outcomes and Findings

#### ABA Impact

After the general data coding stage, any study records from the total sample (*N* = 770) looking only at ABA Impact were coded for outcomes (*N* = 551), i.e., improvement, regression, mix, or no change in the eight outlined outcome categories. Any study records comparing different ABA techniques (*N* = 225) were designated for the next section (see “Comparisons of ABA Techniques,” below). The eight outcomes considered were cognitive, language, social/communication, problem behavior, adaptive behavior, emotional, autism symptoms, and QoL outcomes. Subject QoL is not reported in any tables, as there were no instances of this outcome being measured in the current cohort of study records.

**Fig. 2:**
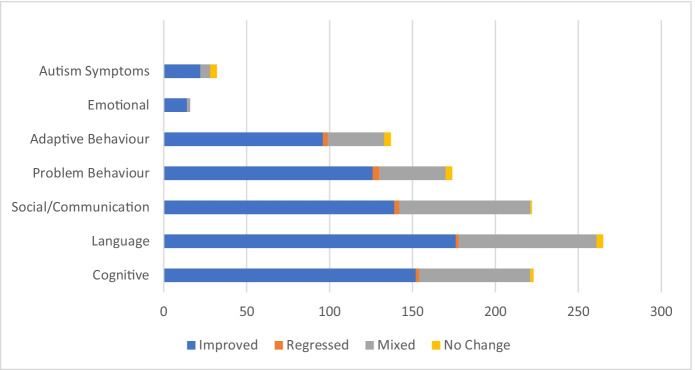
Distribution of Improved, Regressed, Mixed, and Unchanged Results in the ABA Impact Section across the Measured Outcomes (*N* = 551 study records)

The majority of study records reported improvement across all outcome categories, with 63%–88% of study records reporting improvement across the various outcome measures. In contrast, 0%–2% reported regression, 13%–36% reported mixed results, and 0%–13% reported no change (Fig. 2).

**Fig. 3:**
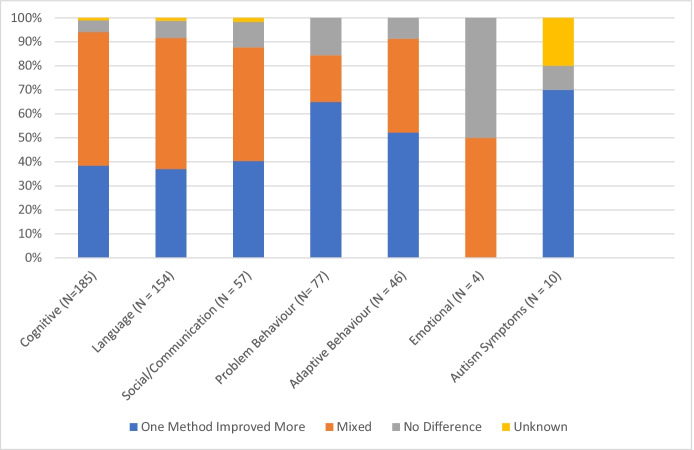
Percentage Distribution of Results Where One Method Improved More, Results were Mixed, Results had No Change, or Results were Unknown (had No Quantifiable Measure) in Comparisons of ABA Techniques Group across the Measured Outcomes (*N* = 225 comparison records)

When observing outcome measures by age group (see Appendix 5, Table S4), among study records conducted with participants between ages 0–5 years, cognitive, language, and social/communication were the most commonly studied outcomes, at 22%, 23%, and 23% respectively. Of these, 66%, 68%, and 57% reported an improvement, respectively. Meanwhile, for ages 6–12, problem behavior and language were the most commonly studied outcomes at 25% each. Among these respective outcomes, 86% and 71% reported improvement. For ages 13–18, the most commonly studied outcome was cognitive (26%), followed by adaptive behavior (20%). Of these, 83% and 86% reported improvement, respectively. Finally, in the mixed-age groups, the most commonly studied outcome was language (28%), followed by social/communication (20%) and cognitive (20%). Of these three most studied outcomes, improvement was reported at 61%, 65%, and 62%, respectively. Detailed findings are available in Table S4 of Appendix 5.

Outcome measures were also divided by sex. Among the study records that only observed females, the most commonly studied outcome was problem behavior at 33%, with social/communication following at 23%. Improvement was recorded 85% and 67% of the time, respectively, for these outcomes. Among records looking at only males, language was the most studied outcome at 26%, followed by cognitive and social/communication at 21% each. These improved at 62%, 66%, and 59%, respectively. Among publications with mixed sexes, the most studied outcome measures were language (25%), cognitive (22%), and social/communication (21%). Of these, 65%, 71%, and 67% showed improvement, respectively.

Outcome measures were then divided by diagnosis (Tables S5 and S6). Among study records solely studying ASD, the most commonly studied outcomes were language, cognitive, and social/communication, making up 25%, 22%, and 22% respectively. Among these respective outcome measures, 68%, 68%, and 63% reported improvement. In the mixed-diagnoses category, the most studied outcomes were problem behavior (31%) and language (22%), with 70% and 58% reporting improvements, respectively. Detailed findings are available in Tables S5 and S6 in Appendix 5.

Next, secondary measures were classified. These included the presence of follow-up, whether interventions assessed mastery or criterion, and whether interventions assessed generalization. Out of the ABA Impact cohort, 41% had some follow-up, 40% had some measure of mastery/criterion, and 31% had some measure of generalization. Among study records that showed improvement within the various outcome measures, use of follow-up measures varied. Records that recorded improvements in cognitive, language, social/communication, and problem behavior outcomes had follow-up measures 47%–59% of the time. Records recording improvement in adaptive behavior and emotional outcomes had follow-up measures 67% and 64% of the time, respectively. Studies reporting improvement in autism symptoms had follow-up measures 100% of the time (see Appendix 5, Table S7). Within the current cohort, out of the study records that signified some improvement, the frequency of mastery/criterion measures varied. Measures of mastery/criterion ranged from 0% and 14%, respectively, for autism symptoms and problem behavior improved outcomes, to 25% and 29%, respectively, for adaptive behavior and social/communication, and 43%–49% for cognitive, language, and emotional improved outcomes (Table S7). With regard to generalization, no study records showing improvements in autism symptoms assessed any measure of generalization. Among other outcomes, generalization measures ranged from 14% for emotional improved outcomes, 24%–29% for problem behavior, adaptive behavior, and cognitive improved outcomes, and 39% and 46%, respectively, for language and social/communication improved outcomes (Table S7).

#### Comparisons of ABA Techniques

Many records from the current search investigated the effectiveness of different ABA methods or variables in delivery. This section of study records was further divided into discrete records wherever more than two variables were compared, for a total of 307 comparison records, which were then coded for outcomes. In this case, coding included which category of comparison was studied, and indicated whether one ABA method performed better, or if the results were mixed or had no change.

Five categories of variables were defined: Teaching, Stimulus Characteristics, Reinforcement, Subject/Setting Characteristics, and Comparing ABA Interventions. These are further described in Appendix 2. Within these categories, most comparison records were unique in the methods examined and thus could not be easily compared across this selection of records. That said, some trends were identified. First, many different teaching procedures were compared, such as how instructions were provided, tact versus listener training, or serial versus concurrent training (Arntzen & Almås, 2002; Delfs et al., 2014; Lee & Singer-Dudek, 2012). Several comparison records investigated the quality of the teaching procedures, commonly with respect to the integrity of reinforcement or teaching techniques (Carroll et al., 2013; Odluyurt et al., 2012). Others investigated the differences in personnel delivering the ABA interventions, such as a parent or clinician (Hayward et al., 2009; Lindgren et al., 2016), or differences in program delivery, such as via specific modeling, reinforcing, or prompting techniques (Campanaro et al., 2020; Jessel et al., 2020; Quigley et al., 2018). A number of comparison records compared time characteristics, such as reinforcement schedules or delays (Majdalany et al., 2016; Sy & Vollmer, 2012). Factors related to reinforcement in general were commonly compared and diverse in nature, spanning the quality, preference, presentation, and other aspects of reinforcement (Allison et al., 2012; Carroll et al., 2016; Fisher et al., 2000; Groskreutz et al., 2011). A few comparison records examined subject characteristics, such as the effectiveness of an ABA intervention based on the age of participant entry into the program or their diagnosis (Luiselli et al., 2000; Schreck et al., 2000), but slightly more commonly measured was the effectiveness of interventions administered in different settings such as at school, at a clinic, or at home (Hayward et al., 2009; Sallows & Graupner, 2005; Schreck et al., 2000). Some comparison records compared specific ABA intervention techniques, such as PRT, the Lovaas/UCLA model, or response interruption and redirection (RIRD), to one another (Dwiggins, 2009; Fernell et al., 2011; Lydon et al., 2011; Mohammadzaheri et al., 2014; Saini et al., 2015).

Table S8 (located in Appendix 5) displays the Comparisons of ABA Techniques group analysis of various intervention categories compared in the outcome measures. Teaching was the most commonly compared intervention category across six outcome measures, ranging from 38% to 64%, except for emotional (25%), and autism symptoms (10%). Comparing ABA interventions was the most commonly studied comparison in the emotional outcome (50%; 2 out of 4 comparison records), and subject/setting characteristics was the most commonly studied comparison in the autism symptom outcome (70%; 7 out of 10 comparison records). The improvement of one method over another was not always prevalent (Fig. 3). Within the cognitive, language, and social/communication outcomes, 37%–40% of comparison records found that one method exhibited greater improvement than the other, whereas 47%–56% had mixed outcomes. This is similar for adaptive behavior, where 52% found that one method exhibited greater improvement and 39% were mixed. On the other hand, outcome measures for problem behavior and autism symptoms more clearly showed that one method exhibited greater improvement, at 65% and 70% (7 out of 10 records), respectively.

#### Between-Groups Comparisons

Many records also investigated the effectiveness of ABA against other interventions or control groups. From the ABA Impact section, these study records comparing measures between groups (*N* = 49) were coded a second time. These were also divided into discrete records whenever more than two groups were compared, for a total of 58 comparison records, which were then coded for outcomes. In this section, coding indicated whether one intervention performed better, or whether there was a mix, no change, or regression. The main interventions of interest in this section were categorized into ABA, EIBI, and I-ABA. Frequent comparisons were to control, which included eclectic (nonspecified), treatment as usual (TAU), or waitlist groups; nursing; portage; the Developmental, Individual Differences, Relationship-based intervention (DIR); or other interventions such as sensory integration therapy and the modified sequential-oral-sensory approach (M-SOS). These categories are further detailed in Appendix 2.

Due to the nature of these interventions, most were longitudinal in study duration, as results were measured after 1 or more years. Moreover, validated measurement tools including Vineland Adaptive Behavior Scales (VABS), Reynell Developmental Language Scales (RDLS), and Bayley Scales of Infant Development-Revised (BSID-R), were more often used to measure changes in this section than in the ABA Impact and Comparisons of ABA Techniques sections, as well as validated parent/caregiver surveys such as the Achenbach Child Behavior Checklist or the Nisonger Child Behavior Rating (Eikeseth et al., 2007; Kovshoff et al., 2011; Smith et al., 2000). Few study records in this category included specific and differentiated probes into the generalization of the improvements seen (*n* = 3; Dugan, 2006; Leaf et al., 2017; Peterson et al., 2019), and few included measurements of mastery or criterion (*n* = 3; Birnbrauer & Leach, 1993; Dugan, 2006; Hilton & Seal, 2007).

Among the Between-Groups Comparisons (see Appendix 5, Table S9), the ABA coding category was the most often improved, showing improvement over the comparison group at least 36% of the time across all outcomes. I-ABA showed improvement over the comparison 18%–30% of the time among cognitive, language, social/communication, adaptive behavior, and autism symptom outcomes. EIBI showed improvement over the comparison 21%–25% of the time among the cognitive, language, social/communication, and adaptive behavior outcomes. TAU and Other interventions occasionally showed greater improvement in some outcome measures (≤ 22% of the time). Nursery, portage, and DIR showed little to no improvement over ABA treatment groups.

### Further Observations between Coding Groups

**Fig 4:**
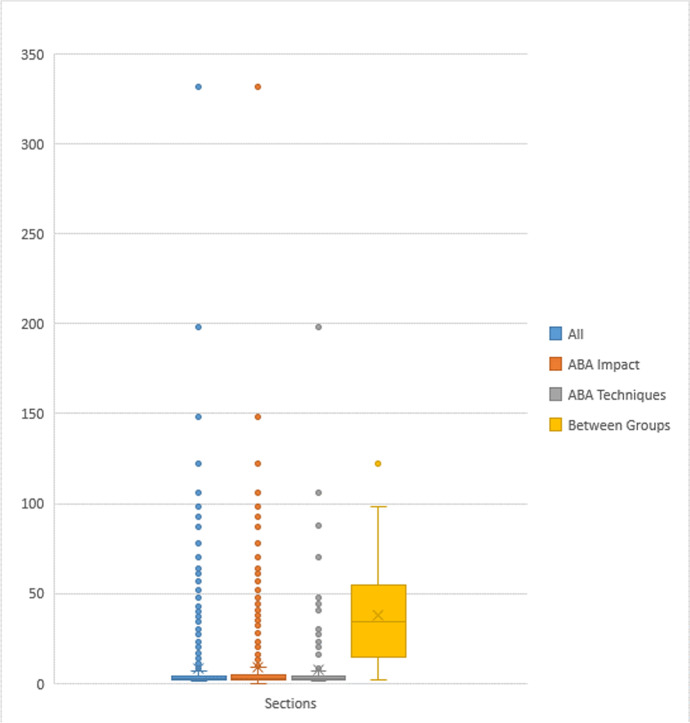
Distribution of the Number of Participants in the Entire Cohort, ABA Impact, Comparisons of ABA Techniques, and Between-Groups Comparisons sections. *Note.* The entire cohort, ABA Impact section, and Comparisons of ABA Techniques section each had a median of 3 participants and an IQR of 1, whereas the Between-Groups Comparisons section had a median of 34 participants and an IQR of 37

Figure 4 shows the distribution of the number of participants across the whole sample, ABA Impact, Comparisons of ABA Techniques, and Between-Groups Comparisons cohorts. The highest number of participants in a study record was 332, whereas the lowest was 1. The Between-Groups Comparisons section had the highest median number of participants at 34, and the largest variation in the number of samples with an interquartile range (IQR) of 37. The entire cohort, ABA Impact section and Comparisons of ABA Techniques section each had a median number of 3 and an IQR of 1, respectively.

In addition to having larger sample sizes and more frequent use of validated measurement scales, records in the Between-Groups Comparisons section more often incorporated statistical analyses, approximately 85% of the time compared with approximately 15% of the entire cohort. Although statistical significance was not considered when initially coding the results in order to align with the rest of the sample, an informal review was conducted based on the reported statistical significance of the improvement of one condition over another. Overall, it was found that not all improvements were significant or assessed for statistical significance (Dawson et al., 2010; Dugan, 2006; Howard et al., 2014; Kovshoff et al., 2011). Among the outcome measures defined in the current review, some records showed significant improvement in some but not all contributing measures (Eikeseth et al., 2002; Reed et al., 2007a; Zachor et al., 2007). Others had statistically significant improvement in all contributing measures of a given outcome (Dixon et al., 2018; Howard et al., 2005; Lovaas, 1987; Novack et al., 2019; Smith et al., 2000; Zachor et al., 2007).

**Fig. 5:**
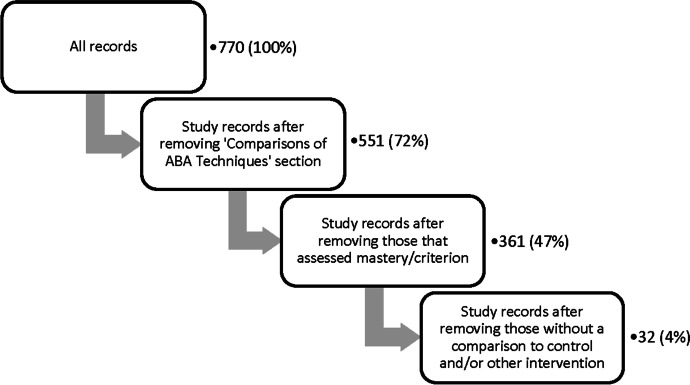
Filter Flow Sheet Representing Study Records after the Subsequent Removal of Various Factors. *Note.* The first filter removed study records that compared various ABA techniques, where 551 of 770 (72%) of records remained. Next, study records that assessed mastery/criterion were removed, leaving 361 of 770 (47%) of records. Next, study records without any comparison group were removed, leaving 32 of 770 (4% records)

The entire cohort of records explored had few occurrences of RCTs, the “gold standard” of research. Of the 12 identified RCTs, 5 were categorized into this review’s Comparisons of ABA Techniques section, whereas the remaining 7 included comparisons to controls or other interventions (Cihon et al., 2020; Dawson et al., 2010; Koenig et al., 2010; Landa et al., 2011; Leaf et al., 2017, 2020; Mohammadzaheri et al., 2014, 2015; Peterson et al., 2019; Reitzel et al., 2013; Scheithauer et al., 2020; Smith et al., 2000). In the interest of identifying a subset of more rigorous records, a three-step filter was conducted (Fig. 5). This was not a formal assessment of study quality, but rather a way to identify the proportion of investigated studies with several specific characteristics. After removing the section of studies looking at Comparisons of ABA Techniques, as well as any studies assessing mastery or criterion, and following with a filter for any inclusion of a comparison to control or other intervention, 32 study records (4%) remained out of 770. That is, only 4% of the entire sample assessed ABA impact, had a comparison group, and did not rely on mastery of specific skills to mark improvement.

There was an observed increase in the amount of ABA literature between 2018 and 2020 compared to the 20-year search between 1997 and 2017. There was also an observed increase in larger scale studies between 2018 and 2020, as also evidenced by the higher frequency of RCTs (*N* = 4; Cihon et al., 2020; Leaf et al., 2020; Peterson et al., 2019; Scheithauer et al., 2020) compared to the preceding 20-year period (*N* = 8, Dawson et al., 2010; Koenig et al., 2010; Landa et al., 2011; Leaf et al., 2017; Mohammadzaheri et al., 2014, 2015; Reitzel et al., 2013; Smith et al., 2000), but overall no notable change in the demographics, sample size, frequencies of outcomes measured, or teaching procedures.

## Discussion

The increasing prevalence of ASD in children and youth across the world has placed evidence-based interventions that treat these disabilities and disorders in high demand. ABA has been at the forefront of these interventions for decades and is recommended by many governments, including in the United States and Canada, as a well-established, scientifically proven therapy (Government of Canada, 2018; U.S. Department of Health & Human Services, 1999). Due to these prominent endorsements, existing and emerging interventions should be held to the same standard as established ABA interventions. That said, to our knowledge, a scoping review into all of the pertinent scientific evidence surrounding ABA has not yet been undertaken. This may result in knowledge gaps regarding this long-standing and widely used intervention and was the reasoning behind the current scoping review.

The results of the current scoping review are consistent with previous review articles and meta-analyses into the overall trend of positive effects of ABA. For example, there were overwhelming positive improvements in the majority of study records with respect to cognition, language development, social skills and communication, and adaptive behavior, along with reductions in problem behavior (Dawson & Bernier, 2013). In the ABA Impact section of the current review, 63%–88% of study records reported improvement across these same outcome measures, in addition to improvements in emotional and autism symptoms outcome measures (Fig. 2). The results of the current analysis into the demographics of these studies are also consistent with the existing literature, as the majority of the participants were male (48%) or there was a mix of females and males (45%) within multiparticipant studies (Kim et al., 2011; Lai et al., 2014; Miller et al., 2016). Further, the sole diagnosis of ASD was more common than mixed diagnoses, as 76% of study records recorded ASD without other diagnoses or comorbidities, again consistent with previous research into ABA (Dawson & Bernier, 2013). With respect to age distribution within the current review, the current results further mirror the previously published literature on EIBI, as children of a younger age tended to be predominately measured on outcomes of cognition, language skills, and social skills (Dawson & Burner, 2011; Reichow et al., 2012; Virués-Ortega, 2010). Children aged 6–12 years were most often measured with respect to changes in problem behavior and language skills, and those 13–18 years of age were most often measured with respect to changes in adaptive behavior and cognitive outcomes, again similar to previous research in older children and youth (Granpeesheh et al., 2009). As reported in other research, participants diagnosed solely with ASD were most often measured upon changes in cognition, language, and social skills and communication (Reichow et al., 2012). It is interesting that the mixed-diagnoses category was also commonly measured on language outcomes, but the most common outcome measure was problem behavior, at 31% of study records in the ABA Impact section.

Based on the number of study records (*N* = 770, Fig. 1), the current findings confirm there is a wealth of scientific knowledge regarding the effect of ABA on children and youth with ASD. Many studies have been published in peer-reviewed journals, but the quality of these studies requires further consideration. The lack of non-ABA comparison groups, rigorous study design, follow-up measures or investigation into generalization of reported outcomes, as well as factors such as small sample sizes, assessment of mastery or criterion, and the use of individualized methods to attain a particular skill or behavior for individual participants, could all contribute to and potentially confound the overarching positive findings seen in ABA research studies.

The gold standard of research is typically denoted as a RCT, followed by CCT or prospective studies. As evident through this scoping review, 64% of all the study records included three or fewer participants, and the median number of participants was three, indicating methods more consistent with SCED. SCEDs are exceedingly valuable within the field of ABA as they inform practitioners of the most effective methods and improve the delivery of ABA services (Tincani & Travers, 2019), in addition to facilitating innovation and detecting changes upon intervention (Smith, 2012). Specific attention can be given to measuring individual changes over time, across differing experimental conditions, in repeated conditions, and with other individuals in order to help establish validity (Perone, 2018). However, this type of study design may not measure statistical significance, lacks generalizability (Tincani & Travers, 2019), and does not assess long-term global effects (Smith, 2012). Although the overall positive results seen across all outcome measures may reflect the individualized impact of ABA, they may not reflect the more global changes or potential impacts on other children or youth with ASD that undergo the same treatment. In addition, many of these study records investigated specific skills, tasks, or responses that varied based on the child (Plavnick & Ferreri, 2011; Romaniuk et al., 2002), potentially making replication and generalization of the overall positive findings to the general population of children and youth with ASD difficult (Smith, 2012).

Few (6%) study records compared ABA interventions to control groups or other non-ABA interventions. Study records that did investigate ABA compared to a control group (typically TAU) or other intervention more often measured statistical significance, had larger sample sizes (Kamio et al., 2015; Koenig et al., 2010), and/or used validated measurement tools such as RDLS and BSID-R (Cohen et al., 2006; Eikeseth et al., 2002; Howard et al., 2005; Kovshoff et al., 2011; Remington et al., 2007). It is interesting that more recent meta-analyses have trended towards fewer statistically significant improvements than what has been previously reported (Reichow et al., 2018; Rodgers et al., 2020). The comparison records in the current review that did have large enough sample sizes to warrant a statistical analysis against a comparison group often did not find significance across all values or measurement tools used (Cohen et al., 2006). That said, a number of study records in the current review, some of which were also investigated by Reichow and colleagues (Cohen et al., 2006; Howard et al., 2014; Magiati et al., 2007; Remington et al., 2007), had comparison groups that differed to varying degrees from the treatment groups in terms of intensity, duration, location, or qualifications of intervention administrators, potentially raising questions about comparisons made between the groups (Reichow et al., 2018).

The current findings are also consistent with other publications with respect to the comparison of ABA techniques, as 225 of the study records investigated the efficacy of various ABA methods compared to one another. Another review found that approximately half of the comparison articles investigated found that one method was better than the other(s), and the other half of the sample indicated that the methods were equally effective (Shabani & Lam, 2013). Thus, this result indicated that only half of the comparisons analyzed truly contributed to the best practices of ABA (Shabani & Lam, 2013). In the current review, this was showcased through cognitive and language outcome measures, which found that only 38% and 37% of the comparison records, respectively, reported greater improvement with one method over the other. These investigations, often SCED, are undoubtedly important within the ABA field of research and to further analyze the effectiveness of one technique or method over another in order to optimize intervention strategies, particularly if rigorously designed (Lobo et al., 2017; Smith, 2012), or designed with an effort to assess and understand social validity (Snodgrass et al., 2021), but do not provide enough information on the overall effectiveness of ABA as a whole on the larger population of children and youth with ASD (Shabani & Lam, 2013).

Approximately 40% of the study records measured success in the given treatment through the assessment or attainment of some level of mastery or criterion for the desired skill or behavior (Grannan & Rehfeldt, 2012; Grow et al., 2011; Toussaint et al., 2016). Because study methods frequently continue until mastery or criterion in order to solidify behaviors and promote better maintenance (Luiselli et al., 2008; McDougale et al., 2020), positive improvements occur organically as subjects attain these desired measures. However, this may not accurately indicate the ability of a participant to maintain such a skill, particularly if the mastery criterion is low (McDougale et al., 2020; Richling et al., 2019). In some instances, criterion parameters and/or experimental procedures were altered in order to reach the desired measure (Charlop et al., 1985; Valentino et al., 2015). Thus, discretion should be taken when evaluating outcomes reliant on the mastery or extinction of skills or behaviors (McDougale et al., 2020). In addition, only 41% of the records conducted some form of investigation into follow-up or maintenance of the given outcome measure(s). This may not be reflective of the long-term effects of the overall positive outcomes. Likewise, generalization was only investigated in 31% of the study records, again prompting the question of whether or not these task- or behavior-specific improvements resulted in overall changes in the child’s skills, function, or behaviors. Further research may be required to assess retained changes rather than changes upon intervention (Bishop-Fitzpatrick et al., 2013; Smith, 2012).

In summary, the above results can be visualized through a filter of the study records (Fig. 5). Out of the 770 (100%) study records that were reviewed in depth, most showed positive results. When study records that used a method with a potential bias for positive results—such as those that compared one ABA treatment to another or assessed the mastery or criterion of a skill or behavior—were excluded, 361 (47%) study records remained. Furthermore, when study records that did not compare to a control or other intervention were excluded, 32 (4%) of the study records remained. These results may indicate gaps in the current ABA research approach, further supporting previous research about the standard of existing ABA literature (Reichow et al., 2018; Smith, 2012). These findings also support recommendations from Smith (2012), suggesting that RCTs comparing ABA to other interventions may be instrumental in evaluating both individual and global changes, as well as revising existing intervention models.

### Limitations of the Current Review

The limitations of the current scoping review are: (1) the broadness of the outcome measures investigated; (2) the potential confounding measure of generalization independently versus within a standardized scale; (3) the definition of ABA itself versus its many treatment derivatives; and (4) the continual development of the diagnostic tools used to assess ASD. Each of these will be described in turn below.

Many of the study records investigated specific tasks, responses, or skills. Thus, improvements in areas such as cognition may be misleading, because both improvements on specific tasks and improvements on full-scale cognitive assessments were scored as improvements in the cognitive outcome category (Grow et al., 2011; Howard et al., 2005). In addition, some of the outcome measures had considerable overlap in definitions, such as the cognition, language, social/communication, and adaptive behavior categories, thus potentially resulting in the coding of multiple outcome measures for a similar task. For example, receptive labeling tasks were coded under both cognitive and language outcome measures (Grow et al., 2011).

The infrequent use of generalization seen in the Between-Groups Comparison section could be a result of the greater use of validated tools in this section of records (Cohen et al., 2006; Remington et al., 2007). Measurement tools such as VABS incorporate measures of generalization into the scale, and though not often specified as an independent measure of generalization, multiple environmental locations for the interventions (e.g., home and school) or multiple individuals interacting with the participants may have been measured.

Given the length of time that ABA has been utilized in treating children with ASD, and its having become the basis for many intervention techniques, it can be difficult to discern whether a particular treatment follows all of the principles of ABA and to what extent. This was seen in a recent review investigating all available interventions for children and youth with ASD (Whitehouse et al., 2020). It may be difficult for families, governments, and policy makers to evaluate available evidence appropriately (Whitehouse et al., 2020). For example, PECS was developed utilizing ABA principles and is commonly used in conjunction with ABA therapy, but it is also used throughout speech and language therapy, education systems that are not solely ABA, and simply as a communication-based intervention (Howlin et al., 2007; Lerna et al., 2012; Pasco & Tohill, 2011). Even within the ABA field there are conflicting definitions of ABA between the research community and public sector (Schreibman et al., 2015), adding another layer of complexity for policy makers when it comes to deciding whether to fund specific programs, specific types of professionals, or a combination of both. For the same reason, there may be some treatments, methods or techniques that have not been included within this scoping review. Further, although the use of “applied behavior analysis” as a search term may not have captured the full extent of behavioral research, its inclusion as both a MeSH term and keyword will have returned any records indexed by the reviewed databases as “applied behavior analysis,” satisfying the initial search criteria for the current scoping review.

As the understood spectrum of ASD and the diagnostic tools for ASD have changed drastically over the decades in which the investigated articles were published, the represented population may have also changed throughout the years, potentially influencing the acceptability of study findings (Reichow et al., 2018). Furthermore, the initial objective for this scoping review included searching across all NDD/D, not just ASD. Thus, the ASD MeSH term of “autistic disorder and autism spectrum disorder” may have potentially resulted in missed studies that included only AS or PDD-NOS diagnoses. That said, as this review was intended to find the scope of the research surrounding the impact of ABA on children and youth with ASD over a time frame of 23 years and across all available research, the authors believe all of the applicable scope was covered within reason.

### Recommendations for Future Research

Recommendations for the further advancement in the field of ABA interventions for children and youth with ASD often include increasing the duration of the study, investigating comparisons to other non-ABA interventions, conducting follow-up studies for adults who participated in ABA interventions as children, and increasing the overall sample sizes. There has been an ongoing recommendation for larger scale studies over the last 20 years with respect to children and youth with ASD (Eldevik et al., 2009; Reichow et al., 2018; Smith, 2012), as well as for long-term outcomes for adults with ASD (Bishop-Fitzpatrick et al., 2013; Rodgers et al., 2020). With respect to EIBI in particular, there is increasing importance for large-scale studies comparing the effectiveness of EIBI against other non-ABA interventions, including developmental social pragmatic (DSP) interventions (Rodgers et al., 2020), which was also evident in the current review, as most comparison records that measured the effectiveness of EIBI compared their results to those of TAU or eclectic treatment approaches (90%; 9 out of 10 comparison records). Overall, although there are merits to both SCEDs and larger-scale group study designs (Lobo et al., 2017; Smith, 2012) there is a greater need for the latter when evaluating ABA. Our findings are in line with the perspective that ABA literature already has a wealth of SCEDs and is overdue for large scale studies such as RCTs to assess existing practices and, perhaps more importantly, to reevaluate and revise evolving ABA practices in the rapidly developing field of intervention for ASD (Smith, 2012).

An important note in terms of finding appropriate and effective interventions in the treatment for ASD, which is not limited to ABA, is the establishment of standards of care (SoC). Unfortunately, even though there is a wealth of knowledge regarding the assessment, diagnosis and treatment of ASD, there is still no clear SoC for the treatment of ASD (Department of Defense, 2019, 2020). In general, outcome measures should indicate a true measure of benefit to the child and their family, in addition to providing relevance within practice and the ability to replicate across research (Rodgers et al., 2020). Recent studies have questioned outcome measures such as cognition and adaptive behaviors when evaluating ASD treatments, and a call for standardized outcome measures that are truly reflective of the benefit for the child and family is beginning to grow (Rodgers et al., 2020). Our recommendation is for more rigorous large-scale prospective comparison studies between ABA and emerging interventions, such as DSP interventions, to be conducted in order to develop gold standard treatment options with a defined SoC for children and families with ASD.

The results of the between-groups comparisons in this scoping review indicated that 23 comparison records compared intensive ABA (20–40 hr of intervention per week) to control or other interventions. Existing literature indicates that 30–40 intervention hours per week for children under the age of 6 results in greater improvements in cognition, language development, social skills, and more (Kovshoff et al., 2011; Reed et al., 2007b). That said, more recent large-scale analyses on children who received 12 months of ABA services indicated that increased intensity does not necessarily predict better outcomes (Department of Defense, 2020). In a meta-analysis completed by Rodgers et al. (2020), autism symptoms showed no statistically significant improvements with higher intensity EIBI treatments as opposed to lower intensity EIBI treatments. It was also found that no one age group demonstrated improvement when correlated with the number of hours of rendered ABA services (Department of Defense, 2020). This evidence suggests there may be insufficient recent research justifying the need for high-intensity interventions, indicating that more research studies need to be conducted in the field of ABA in terms of assessing ABA impact with different or lower intensity interventions.

Most of the current literature surrounding ABA-based interventions lacks investigations into the QoL of children with ASD and instead focuses on aberrant behaviors (Reichow et al., 2018; Whitehouse et al., 2020). A recent meta-analysis found that, upon analyzing five articles of higher scientific credence, none conducted investigations into the changes with respect to QoL for the children or parents (Reichow et al., 2018). The present scoping review likewise found no occurrences of subject QoL measures in the sample analyzed. Overall changes in QoL for children living with ASD is of the utmost importance, as QoL is “individuals’ perception of their position in life in the context of the culture and value systems in which they live and in relation to their goals, expectations, standards and concerns” (WHO, 1997, p. 1). The continued lack of research into long-term effectiveness of ABA treatments is an ongoing concern and should be a focus of future research to help measure QoL (Whitehouse et al., 2020) and also to investigate any possible adverse effects (Rodgers et al., 2020). For example, recent literature investigating adults with ASD who participated in ABA treatments when they were young has shown increases in incidences of posttraumatic stress disorder (PTSD); this is an emerging field of research in adults with ASD and should be further investigated through long-term studies (Kupferstein, 2018).

Future research into the cost-effectiveness of ABA-based interventions compared to existing and emerging interventions should be conducted, as only a few articles within the current review discussed the cost effectiveness of the ABA interventions in use (Farrell et al., 2005; Kamio et al., 2015; Magiati et al., 2007; Park et al., 2020). In the few incidences where cost-effectiveness was measured, the results varied. For example, one study found that higher ABA program cost was associated with lesser improvements in language development (Kamio et al., 2015), one reported higher costs for the Lovaas/ABA model program (Farrell et al., 2005), one found little difference in cost between nursery and ABA interventions (Magiati et al., 2007), whereas Park et al. (2020) found lower costs for their specific ABA model (Korean Advancement of Behavior Analysis [KAVBA]) children’s center as compared to other Comprehensive Application of Behavioral Analysis to Schooling (CABAS) centers. In conclusion, these long-term and intensive interventions should be further investigated with respect to their cost-effectiveness and overall improvements in QoL (Rodgers et al., 2020; Whitehouse et al., 2020).

## Conclusion

As ever in the scientific process, interventions and treatments need consistent and replicative investigations under stringent protocols to ensure the continued efficacy and generalizability of a given intervention. According to the U.S. Department of Health and Human Services (1999), ABA is the gold standard treatment for ASD, and is funded almost exclusively across North America. The current scoping review spanning 770 study records showed positive and beneficial effects of ABA for children with ASD across seven outcome measures. However, only 32 (4%) assessed ABA impact, had a comparison group, and did not rely on mastery of specific skills to mark improvement.

Without ongoing research and the development of a SoC, governments and policy makers will not have the most up-to-date information that reflects ABA-based and other interventions in terms of the ever-changing landscape of diagnoses, modern technological advancements, changes within the intervention implementation, and measurement tools of treatment efficacy. One such example is the measure of subject QoL, which, as made evident by this scoping review, was not measured in any study record included, but is of utmost importance to truly indicate the overall long-term impact of ABA. Moreover, as the children and youth who participated in ABA-based and other interventions become adults, the long-lasting effects of these interventions should be investigated more thoroughly.

Therefore, large longitudinal prospective studies comparing ABA-based and different interventions treating children and youth with ASD are needed. As ABA is historically based on an operant conditioning approach to treatment whereas many emerging interventions typically use a social pragmatic approach (Whitehouse et al., 2020), continued research comparing these two differing ideologies is particularly important, as ABA is currently the bar to which other interventions are held at the governmental level. With a holistic view of all of the scientific evidence behind ABA, governments will be able to more accurately compare any existing and emerging interventions to the well-established norm of ABA. Until a SoC is established, all interventions for children and youth with ASD must be held to the existing standard set by ABA to be considered effective.

## Supplementary Information

ESM 1 (DOCX 502 kb)

ESM 2 (DOCX 19.7 kb)

ESM 3 (DOCX 166 kb)

ESM 4 (DOCX 110 kb)

ESM 5 (DOCX 38 kb)

## Data Availability

Not applicable.
