# Supplementary material for: Applied Behavior Analysis in Children and Youth with Autism Spectrum Disorders: A Scoping Review
Source: Perspect Behav Sci. 2022 May 18;45(3):521–57. doi: 10.1007/s40614-022-00338-x (PMC9458805; doi:10.1007/s40614-022-00338-x)
Supplement: Supplementary file 1 — (DOCX 502 kb) [file 40614_2022_338_MOESM1_ESM.docx]

Appendix 1.

*Search Terms*

The below search strategy pertains to both phases of the search, with the exception of the date ranges, which were altered to match the corresponding phases: January 1, 1997 through December 31, 2017, and January 1, 2018 through December 31, 2020.

*PubMed Search History*

"Intellectual Disability"[Mesh] OR "Disabled Persons"[Mesh] OR "Developmental Disabilities"[Mesh] OR "Rett Syndrome"[Mesh] OR "Down Syndrome"[Mesh] OR "Autistic Disorder"[Mesh] OR "Autism Spectrum Disorder"[Mesh] OR "Cerebral Palsy"[Mesh] OR "Attention Deficit Disorder with Hyperactivity"[Mesh] OR "Fetal Alcohol Spectrum Disorders”[Mesh] OR “neurodevelopment disorder” [tiab] OR “neurodevelopment delay” [tiab] OR “neurodisability” [tiab] OR "Global developmental delay" [tiab] OR "developmental coordination disorder" [tiab] OR "Motor Skills Disorders"[Mesh] OR "Movement Disorders"[Mesh]

AND

"Applied Behavior Analysis"[Mesh] OR "Applied behavior Analysis" OR "Applied behavioral Analysis" OR "Applied behavioural Analysis" OR "Applied behaviour Analysis"

*Limitations:*

Publication date from 1997/01/01 to 2017/12/31; English; Child: birth-18 years

*MEDLINE (EBSCOhost) Search History*

MH “Developmental Disabilities” OR MH “Autistic Disorder” OR MH “"Child Development Disorders, Pervasive"” OR “Neurodevelopmental Disability” OR “Neurodevelopmental Delay” OR MH “Rett Syndrome” OR MH “Cerebral Palsy” OR MH “Down Syndrome” OR MH “Intellectual Disability” OR MH “Attention Deficit Disorder” OR MH “Attention Deficit Disorder with Hyperactivity” OR MH “Fetal Alcohol Spectrum Disorders” OR MH “Cerebral Palsy” OR "global developmental delay" OR "developmental coordination disorder" OR MH "Movement Disorders" OR "movement disorders" OR MH "Motor Skills Disorders" OR "motor skills disorders"

AND

MH "Applied Behavior Analysis" OR "applied behavior analysis" OR "applied behavioral analysis" OR "applied behaviour analysis" OR "applied behavioural analysis"

*Limitations:*

Date of Publication: 19970101-20171231; Age Related: All Child: 0-18 years; Language: English

*CINAHL Search History*

MH “Intellectual Disability” OR MH “Developmental Disabilities” OR MH “Child, Disabled” OR “Developmental Delay” OR MH “Autistic Disorder” OR MH “Child Development Disorders, Pervasive” OR “Neurodevelopmental Disorder” OR MH “Rett Syndrome” OR “Neurodisability” OR MH “Cerebral Palsy” OR MH “Down Syndrome” OR “Attention Deficit Disorder” OR MH “Attention Deficit Hyperactivity Disorder” OR MH “Fetal Alcohol Disorder” OR "global developmental delay" OR "developmental coordination disorder" OR "motor skills disorders" OR MH "Motor Skills Disorders" OR "movement disorders" OR MH "Movement Disorders"

AND

"applied behavior analysis" OR MH "Applied Behavior Analysis" OR "applied behavioral analysis" OR "Applied behavioural analysis" OR "applied behaviour analysis" OR "ABA"

*Limitations:*

Published Date: 19970101-20171231; Language: English; Age Groups: Infant, Newborn: birth-1 month, Infant: 1-23 months, Child, Preschool: 2-5 years, Child: 6-12 years, Adolescent: 13-18 years, All Infant, All Child

*PsychINFO Search History*

DE “Developmental Disabilities” OR DE “Autism Spectrum Disorders” OR “Neurodevelopmental Disability” OR “Neurodevelopmental Delay” OR DE “Rett Syndrome” OR DE “Cerebral Palsy” OR DE “Down’s Syndrome” OR DE “Intellectual Development Disorder” OR DE “Attention Deficit Disorder” OR DE “Attention Deficit Disorder with Hyperactivity” OR DE “Fetal Alcohol Syndrome” OR DE "Dyspraxia" OR "developmental coordination disorder" OR "global developmental delay" OR "Motor skills disorders" OR DE "Movement Disorders" OR "Movement Disorders"

AND

"Applied behavior analysis" OR "Applied behaviour analysis" OR "ABA" OR "applied behavioral analysis" OR "Applied behavioural analysis" OR DE "Behavior Analysis"

*Limitations:*

Published Date: 19970101-20171231; Language: English; Age Groups: Childhood (birth-12 yrs), Adolescence (13-17 yrs)

*ERIC Search History*

“Intellectual Disability” OR DE “Mental Retardation” OR "Neurodevelopmental Delay" OR "Neurodevelopmental Disability” OR DE “Autism” OR DE “Pervasive Developmental Disorder” OR DE “Attention Deficit Disorder” OR “Attention Deficit Hyperactivity Disorder” OR DE “Fetal Alcohol Syndrome” OR “Rett Syndrome” OR DE “Down Syndrome” OR "developmental coordination disorder" OR "Global developmental delay" OR "motor skills disorders" OR "movement disorders"

AND

DE “Children” OR DE “Pediatrics” OR “Adolescents” OR “Youth”

AND

"applied behaviour analysis" OR "applied behavior analysis" OR "ABA" OR "applied behavioral analysis" OR "Applied behavioural analysis"

*Limitations:*

Published Date: 19970101-20171231; Language: English

*CENTRAL Search History*

"developmental disabilities" OR "autism" OR "child development disorders" OR "neurodevelopmental disability" OR "neurodevelopmental delay" OR "cerebral palsy" OR "down syndrome" OR "intellectual disability" OR "attention deficit disorder with hyperactivity" or "fetal alcohol spectrum disorders"

AND

"child" OR "children" OR "adolescent" OR "youth"

AND

"applied behavior analysis" or "applied behaviour analysis"

*Limitations:*

Published Date: 19970101-20171231; Language: English

*CDSR Search History*

"developmental disabilities" OR "autism" OR "child development disorders" OR "neurodevelopmental disability" OR "neurodevelopmental delay" OR "cerebral palsy" OR "down syndrome" OR "intellectual disability" OR "attention deficit disorder with hyperactivity" or "fetal alcohol spectrum disorders"

AND

"child" OR "children" OR "adolescent" OR "youth"

AND

"applied behavior analysis" or "applied behaviour analysis"

*Limitations:*

Published Date: 19970101-20171231; Language: English
