# Supplementary material for: Applied Behavior Analysis in Children and Youth with Autism Spectrum Disorders: A Scoping Review
Source: Perspect Behav Sci. 2022 May 18;45(3):521–57. doi: 10.1007/s40614-022-00338-x (PMC9458805; doi:10.1007/s40614-022-00338-x)
Supplement: Supplementary file 2 — (DOCX 19.7 kb) [file 40614_2022_338_MOESM2_ESM.docx]

Appendix 2.

*Coding detail*

1. *General data coding*

The entire sample underwent general data coding and was later separated into groups for outcome coding. Items selected for general data coding included publication details, population metrics, and several study method details. First author, publication year, journal, and impact factors were coded by one researcher.

*Population*

Four coding values were selected for population age: 0–5 years, 6–12 years, 13–18 years, or mixed age. These were selected to align with transitional school ages. Coded by one researcher and reviewed by a second researcher.

*Diagnosis*

Diagnosis was also coded using two values: ASD (ASD/PDD-NOS/AS), or mixed-diagnoses. The mixed-diagnoses value was assigned both in cases of multiple participants having different diagnoses and in cases of single subjects having multiple co-occurring diagnoses. Frequently occurring other diagnoses included ADHD, ID, GDD or other developmental delays, ODD, Down syndrome, CP, FASD, Angelman syndrome, Fragile X, OCD, Tourette syndrome, TBI, epilepsy or seizure disorders, sensory integration or processing disorders, speech/language delays, learning disabilities, and behavior, emotional, or mood disorders. These were coded and compared by two independent researchers.

*Methods*

Studies were coded to indicate the use of the following methods: (a) follow-up or maintenance, (b) mastery or criterion measures, and/or (c) generalization or extension. For the purposes of this scoping review, “follow-up” refers to both short-term pre-/post-test studies that included a defined follow-up or maintenance phase, as well as longitudinal studies with repeated measures. This column also had a designated coding value for retrospective studies. Studies including comparison groups were further coded by one researcher to identify presence of (a) a control group (typically consisting of “eclectic” or TAU), (b) comparisons to other non-ABA intervention/s, or (c) a mix of (a) and (b).

Publication, population, and method metrics were all coded at the study record level. Outcome measures were coded at the comparison record level. That is, when more than two conditions were compared, study records were discussed and split by researchers to describe simplified or single-variable comparisons. These are termed “comparison records” in this scoping review. The Comparisons of ABA Techniques and Between-Groups Comparisons coding sections both utilize comparison records.

*Outcome measures*

Although the search was not restricted, the observed outcome measures were initially classed into eight categories: cognitive, language, social/communication, problem behavior, adaptive behavior, emotional, autism symptoms, and quality of life outcomes. Quality of life was removed from coding, as no instances of subject quality of life measures occurred in the extracted sample. Prior to coding, researchers categorized observed outcome measures and measurement scales or strategies into coding reference tables in an effort to mitigate potential inconsistencies in coding:

*Cognitive* included topics such as executive function, attention, learning, and scales such as BSID, Mullen Scales of Early Learning, and various measures of IQ.

*Language* included topics such as receptive and expressive language, matching and copying, scripting, and scales such as RDLS, Peabody Picture Vocabulary Test, and subscales of the VABS.

*Social/communication* included topics such as expressive language, nonverbal communication, play, social skills, and scales such as Early Social-Communication Scales, Structured Play Assessment, and subscales of VABS.

*Problem behavior* included topics such as self-injurious behavior, stereotyped behavior, aggression, and scales such as Developmental Behaviour Checklist, Child Behavior Checklist, Repetitive Behavior Scale - Revised, and subscales of VABS.

*Adaptive behavior* included topics such as daily living skills, vocational skills, restrictive behaviors and adaptation to change, and scales such as VABS.

*Emotional* included topics such as emotional responses and affect, emotional regulation, tolerance, and anxiety.

*Autism symptoms* included scales such as Childhood Autism Rating Scale, Gilliam Autism Rating Scale, Autism Diagnostic Interview - Revised, Autism Screening Questionnaire, Autism Behavior Checklist, and Autism Diagnostic Observation Schedule.

Within each category, outcomes were classified as improvement, regression, mix, or no change. Where methods or interventions were compared, further detail was extracted regarding the category of methods or interventions compared and the relative effectiveness of each. Although researchers consulted agreed-upon criteria for outcome measures, there was some subjectivity in coding certain outcomes due to the inherent overlap in many of these measures – such as social skills with adaptive behavior or language development with communication.

1. *Outcome measures - ABA Impact*

Study records in this section looked at outcomes with ABA intervention. Longitudinal studies and large-scale group comparisons were coded based on within-group trends for the population receiving ABA treatment, not on between-group comparisons. (See section *4. Outcome measures – Between-Groups Comparisons* for further detail on between-group comparisons).

*Outcome direction*

The presence of the eight identified ABA outcome measures were coded for each study record, and the subsequent results of these measures were coded in terms of improvement, regression, mixed results, no change, or no quantifiable measure. Results were coded as “improved” only when outcomes for all reported subjects showed a trend toward the desired outcome, or, for large-scale studies, when the group measures had a trend toward the desired outcome. “Regressed” was coded in the same manner. Results were coded as “mixed” when individually reported results varied between subjects or when there were group measures for different aspects of a given outcome that showed different trends.

1. *Outcome measures - Comparisons of ABA Techniques*

Comparison records in this section described comparisons of different ABA techniques, intensities, or other variables within the scope of ABA intervention, rather than outcomes with a single intervention type.

*Outcome direction*

Outcomes of comparison records were coded to show whether (a) one variable improved more, (b) the results were mixed, or (c) had no change.

*Comparison coding*

Comparison records were coded to indicate the comparison category. Prior to coding, researchers categorized intervention categories into coding reference tables in an effort to mitigate potential inconsistencies in coding:

*Teaching* included variables such as personnel, quality of teaching, methods of teaching (such as mand, tact, and echoic training, Discrete Trial Instruction, distributed practice versus massed practice, direct teaching, fluency versus accuracy training, variations on prompt fading, generic, tactical, intensive, or strategic instruction, and so on), as well as methods of prompting and modeling.

*Stimulus characteristics* included variables such as item preference or characteristics of stimuli (location, repetition, verbal or nonverbal, and so on).

*Reinforcement* included variables such as quality or type of reinforcer, delivery of reinforcers, paired reinforcers, or rules or schedules of reinforcement (such as immediate, contingency-based, or delayed).

*Subject/setting characteristics* included variables such as age of entry into an intervention or setting of intervention (classroom, school, telehealth).

*Comparisons of ABA interventions* included comparisons of defined ABA methods across the above categories, between different intensities, or broader treatment packages, such as PRT, expressive language training, UCLA interventions, and so on.

1. *Outcome measures - Between-Groups Comparisons*

Study records from the ABA Impact section that compared ABA techniques to a control or different intervention(s) were coded a second time to describe these between-groups results.

*Outcome direction*

As above, all comparison records were coded for selected outcomes and to indicate whether (a) one improved more, (b) results were mixed, (c) results had no change, or (d) a regression occurred. In addition, for instances where one intervention performed better than the other, the intervention with greater and lesser effect were each indicated.

*Intervention/control type*

Observed intervention and control types included ABA, EIBI, I-ABA, nursing, portage, DIR, and others. Prior to coding, researchers categorized intervention categories into coding reference tables in an effort to mitigate potential inconsistencies in coding:

*ABA:* ABA not specified as intensive or specific with respect to age.

*EIBI:* ABA specifically targeted for children under the age of 6 and intensive in delivery (30+ hours a week).

*I-ABA:* ABA specified as intensive (30+ hours a week) but not specifically targeted at children under the age of 6.

*Control:* non-treatment group, TAU, waitlist, generic programs, undefined eclectic programs, education as usual, or parent care.

*Nursery:* as stated.

*Portage:* as stated.

*DIR:* as stated.

*Other:* other interventions targeted specifically with set goals and/or intensities, such as eclectic developmental or Autism programming, Lancashire Under Fives Autism Project (LUFAP), sensory integration therapy, modified sequential oral sensory approach (M-SOS), and Treatment and Education of Autistic and related Communication-handicapped CHildren (TEACCH).

Overall, most study records fell into either the Comparison of ABA Techniques section alone or into the general ABA Impact coding section, from which the Between-Groups Comparisons were also pulled for coding. Three study records were unique in that they had comparisons valid for both the Comparison of ABA Techniques section and the Between-Groups Comparison section and were thus coded in all three sections (Dugan, 2006; Kalgotra et al., 2019; Kovshoff et al., 2011). Three study records were coded in both the ABA Impact section and the Comparisons of ABA Techniques section (Mello et al., 2018; Rad et al., 2019; Vietze & Lax, 2020).

The reference list for all extracted articles can be found in Appendix 4.
