# Supplementary material for: Applied Behavior Analysis in Children and Youth with Autism Spectrum Disorders: A Scoping Review
Source: Perspect Behav Sci. 2022 May 18;45(3):521–57. doi: 10.1007/s40614-022-00338-x (PMC9458805; doi:10.1007/s40614-022-00338-x)
Supplement: Supplementary file 3 — (DOCX 166 kb) [file 40614_2022_338_MOESM3_ESM.docx]

Appendix 3.

*Extraction Tables*

Table S1. Extraction data from the ABA Impact group. In the ‘Study Author & Year’ column, records contain letter labels when multiple articles with the same first author were published in the same year. Records contain letter and number labels when extracted articles had multiple parts and were split into discrete study records. In some cases, not all split study records were retained due to meeting exclusion criteria.

|  | Study Author & Year | Total Number of Participants | Age Group | Gender | Diagnosis | Measured Outcomes |
| --- | --- | --- | --- | --- | --- | --- |
| 1 | Ahearn 2003 | 1 | 13 to 18 years | Male | ASD | Adaptive Behavior (+) |
| 2 | Ahearn 2007 | 4 | Mixed Age Groups | Mixed Gender | ASD | Social/Communication (+/-) Problem Behavior (+) |
| 3 | Ahrens 2011 - A3 | 1 | 0 to 5 years | Male | ASD | Problem Behavior (+/-) |
| 4 | Akers 2016 | 3 | 0 to 5 years | Male | ASD | Adaptive Behavior (+) |
| 5 | Akers 2018 | 3 | 0 to 5 years | Mixed Gender | ASD | Language (+) Social/Communication (+) |
| 6 | Alcantara 1994 | 3 | 6 to 12 years | Mixed Gender | ASD | Cognitive (+) |
| 7 | Allan 2015 | 4 | Mixed Age Groups | Male | ASD | Language (+/-) |
| 8 | Almon-Morris 2007 | 1 | 6 to 12 years | Female | ASD | Social/Communication (+) Emotional (+) |
| 9 | Alsharif 2020 - A1 | 3 | 6 to 12 years | Mixed Gender | ASD | Cognitive (+) |
| 10 | Alsharif 2020 - A2 | 4 | 6 to 12 years | Mixed Gender | ASD | Cognitive (+) |
| 11 | Alzrayer 2020 | 3 | Mixed Age Groups | Male | ASD | Cognitive (+) Language (+) |
| 12 | Anderson 1987 | 14 | 0 to 5 years | Mixed Gender | ASD | Cognitive (+) Language (+) Social/Communication (+/-) Adaptive Behavior (+) |
| 13 | Anderson 2001 | 1 | 0 to 5 years | Male | Mixed Diagnoses | Problem Behavior (+) Adaptive Behavior (+) |
| 14 | Anderson 2002 | 4 | Mixed Age Groups | Mixed Gender | Mixed Diagnoses | Problem Behavior (+/-) |
| 15 | Anderson 2016 | 1 | 0 to 5 years | Male | ASD | Language (+) Social/Communication (+) Problem Behavior (+) |
| 16 | Apple 2005 - A1 | 2 | 0 to 5 years | Male | ASD | Language (+/-) Social/Communication (+/-) |
| 17 | Apple 2005 - A2 | 3 | 0 to 5 years | Mixed Gender | ASD | Language (+) Social/Communication (+) |
| 18 | Aravamudhan 2020 | 2 | Mixed Age Groups | Mixed Gender | ASD | Language (+) Social/Communication (+) |
| 19 | Argyropoulou 2012 | 1 | 6 to 12 years | Male | ASD | Social/Communication (+/-) |
| 20 | Armstrong 2014 | 1 | 13 to 18 years | Female | Mixed Diagnoses | Problem Behavior (+) |
| 21 | Asmus 1999 | 3 | 0 to 5 years | Male | Mixed Diagnoses | Cognitive (+/-) Social/Communication (+/-) Problem Behavior (+/-) Adaptive Behavior (+/-) |
| 22 | Athens 2008 | 1 | 6 to 12 years | Male | Mixed Diagnoses | Problem Behavior (+) |
| 23 | Axe 2010 | 4 | 0 to 5 years | Mixed Gender | ASD | Cognitive (+/-) |
| 24 | Baker 2000 | 3 | Mixed Age Groups | Mixed Gender | ASD | Social/Communication (+) |
| 25 | Barkaia 2017 | 3 | Mixed Age Groups | Male | ASD | Language (+/-) |
| 26 | Barnoy 2009 | 1 | 6 to 12 years | Female | ASD | Problem Behavior (+) |
| 27 | Bartman 2003 | 1 | 0 to 5 years | Female | ASD | Language (+) |
| 28 | Beaulieu 2018 | 1 | 6 to 12 years | Female | ASD | Social/Communication (+) Problem Behavior (+) Emotional (+) |
| 29 | Beglinger 2005 | 35 | Mixed Age Groups | Mixed Gender | ASD | Cognitive (+) |
| 30 | Belisle 2016 | 3 | Mixed Age Groups | Male | ASD | Social/Communication (+) |
| 31 | Belisle 2019 | 2 | 13 to 18 years | Male | Mixed Diagnoses | Cognitive (+) |
| 32 | Belisle 2020 - A1 | 1 | 13 to 18 years | Male | ASD | Cognitive (+) |
| 33 | Belisle 2020 - A2 | 1 | 13 to 18 years | Male | ASD | Cognitive (+) |
| 34 | Belisle 2020 - B1 | 2 | 0 to 5 years | Mixed Gender | ASD | Cognitive (+) Language (+) |
| 35 | Belisle 2020 - B2 | 2 | 6 to 12 years | Male | ASD | Cognitive (+) Language (+) |
| 36 | Belisle 2020 - C | 3 | Mixed Age Groups | Male | ASD | Cognitive (+) Language (+) Emotional (+) |
| 37 | Ben-Itzchak 2007 | 25 | 0 to 5 years | Mixed Gender | ASD | Cognitive (+) Language (+) Social/Communication (+) Problem Behavior (+) |
| 38 | Bergstrom 2012 | 3 | 6 to 12 years | Male | ASD | Social/Communication (+) Adaptive Behavior (+) |
| 39 | Bergstrom 2014 | 3 | 6 to 12 years | Male | ASD | Social/Communication (+) Adaptive Behavior (+) |
| 40 | Bergstrom 2016 | 3 | Mixed Age Groups | Mixed Gender | ASD | Social/Communication (+) |
| 41 | Betz 2008 | 6 | 0 to 5 years | Mixed Gender | ASD | Social/Communication (+/-) Adaptive Behavior (+/-) |
| 42 | Betz 2011 | 3 | 0 to 5 years | Mixed Gender | ASD | Language (+/-) |
| 43 | Bibby 2002 | 66 | Mixed Age Groups | Mixed Gender | ASD | Cognitive (+/-) Language (+/-) Problem Behavior (+) Adaptive Behavior (+) ASD Symptoms (=) |
| 44 | Bicard 2010 | 4 | 13 to 18 years | Mixed Gender | Mixed Diagnoses | Cognitive (+) Adaptive Behavior (+) |
| 45 | Birnbrauer 1993 | 14 | 0 to 5 years | Mixed Gender | ASD | Cognitive (+) Language (+/-) Social/Communication (+/-) Problem Behavior (+/-) Adaptive Behavior (+) Parent Stress (+) |
| 46 | Bloom 2013 | 3 | 0 to 5 years | Male | Mixed Diagnoses | Problem Behavior (+) |
| 47 | Bourret 2004 - A2 | 3 | Mixed Age Groups | Male | Mixed Diagnoses | Language (+/-) |
| 48 | Boutain 2020 | 3 | 0 to 5 years | Mixed Gender | ASD | Cognitive (+) Problem Behavior (+) Adaptive Behavior (+) |
| 49 | Brodhead 2016 | 3 | 0 to 5 years | Male | ASD | Language (+) |
| 50 | Brodhead 2018 | 3 | Mixed Age Groups | Mixed Gender | ASD | Cognitive (+) Adaptive Behavior (+) |
| 51 | Buckley 2006 | 1 | 6 to 12 years | Male | ASD | Problem Behavior (+) |
| 52 | Buckley 2020 | 2 | 13 to 18 years | Male | ASD | Problem Behavior (+) Emotional (+) |
| 53 | Budzińska 2009 | 1 | 0 to 5 years | Female | ASD | Problem Behavior (+) |
| 54 | Budzińska 2014 | 1 | 0 to 5 years | Female | ASD | Language (+) Social/Communication (+) |
| 55 | Byrne 2014 | 3 | 6 to 12 years | Mixed Gender | Mixed Diagnoses | Language (+/-) |
| 56 | Calise 2009 | 1 | 6 to 12 years | Female | ASD | Language (+) |
| 57 | Carbone 2010 | 3 | Mixed Age Groups | Male | Mixed Diagnoses | Language (+) |
| 58 | Carlile 2018 | 6 | Mixed Age Groups | Male | ASD | Cognitive (+/-) Language (+/-) Social/Communication (+/-) Adaptive Behavior (+/-) |
| 59 | Carnett 2020 - A1 | 3 | Mixed Age Groups | Mixed Gender | Mixed Diagnoses | Language (+) |
| 60 | Carnett 2020 - A2 | 2 | Mixed Age Groups | Male | ASD | Language (+) |
| 61 | Carter 2001 | 3 | Mixed Age Groups | Mixed Gender | ASD | Language (+) Social/Communication (+) Problem Behavior (+) |
| 62 | Casey 2008 | 1 | 6 to 12 years | Male | Mixed Diagnoses | Problem Behavior (+) |
| 63 | Cassella 2011 | 2 | Mixed Age Groups | Male | ASD | Language (=) Problem Behavior (+/-) |
| 64 | Chan 2008 | 2 | Mixed Age Groups | Male | ASD | Cognitive (+) Social/Communication (+) Problem Behavior (+) |
| 65 | Charania 2010 | 3 | 6 to 12 years | Male | ASD | Cognitive (+) |
| 66 | Charlop 1985 | 7 | Mixed Age Groups | Male | ASD | Cognitive (+/-) Language (+/-) |
| 67 | Charlop 1989 | 3 | 6 to 12 years | Male | ASD | Cognitive (+) Language (+) Social/Communication (+) |
| 68 | Charlop 1991 | 3 | 6 to 12 years | Male | ASD | Language (+/-) Social/Communication (+/-) |
| 69 | Charlop 2003 | 3 | 6 to 12 years | Male | ASD | Cognitive (+/-) Language (+/-) |
| 70 | Charlop-Christy 1998 | 3 | 6 to 12 years | Mixed Gender | ASD | Problem Behavior (+) |
| 71 | Charlop-Christy 2002 | 3 | Mixed Age Groups | Male | ASD | Cognitive (+) Language (+) Social/Communication (+) Problem Behavior (+) |
| 72 | Charlop-Christy 2003 | 3 | 6 to 12 years | Male | ASD | Cognitive (+) Language (+) Social/Communication (+) |
| 73 | Cihon 2019 | 4 | Mixed Age Groups | Mixed Gender | ASD | Cognitive (+) Social/Communication (+) |
| 74 | Cihon 2020 - A | 2 | Mixed Age Groups | Male | ASD | Cognitive (+) Language (+) |
| 75 | Cohen 2006 | 42 | 0 to 5 years | Mixed Gender | ASD | Cognitive (+) Language (+) Adaptive Behavior (+) |
| 76 | Cohenour 2018 | 3 | Mixed Age Groups | Male | Mixed Diagnoses | Cognitive (+/-) |
| 77 | Colon 2012 | 3 | 6 to 12 years | Mixed Gender | ASD | Language (+) Problem Behavior (+) |
| 78 | Colon 2019 - A1 | 2 | 13 to 18 years | Mixed Gender | ASD | Problem Behavior (+) |
| 79 | Conallen 2017 - A1 | 10 | Mixed Age Groups | Mixed Gender | ASD | Language (+) Social/Communication (+) |
| 80 | Conallen 2017 - A2 | 10 | Mixed Age Groups | Mixed Gender | ASD | Cognitive (+) Language (+) Social/Communication (+) |
| 81 | Conine 2020 - A | 13 | Mixed Age Groups | Mixed Gender | ASD | Cognitive (+/-) Language (+/-) Social/Communication (+/-) |
| 82 | Conine 2020 - B | 4 | Mixed Age Groups | Mixed Gender | ASD | Cognitive (+) Language (+) Social/Communication (+) |
| 83 | Connolly 2016 | 1 | 0 to 5 years | Female | Mixed Diagnoses | Cognitive (+/-) Language (+) Social/Communication (+/-) Problem Behavior (+) Adaptive Behavior (+) Emotional (+) |
| 84 | Contreras 2016 | 3 | Mixed Age Groups | Male | ASD | Cognitive (+/-) Language (+/-) |
| 85 | Contrucci Kuhn 2006 - A1 | 3 | Mixed Age Groups | Male | Mixed Diagnoses | Problem Behavior (+/-) |
| 86 | Contrucci Kuhn 2006 - A2 | 4 | Mixed Age Groups | Mixed Gender | Mixed Diagnoses | Problem Behavior (+) |
| 87 | Contrucci Kuhn 2006 - A3 | 3 | Mixed Age Groups | Mixed Gender | Mixed Diagnoses | Problem Behavior (+/-) |
| 88 | Cook 2015 | 1 | 6 to 12 years | Male | ASD | Problem Behavior (+) |
| 89 | Coolican 2010 | 8 | 0 to 5 years | Mixed Gender | ASD | Language (=) Social/Communication (+) Problem Behavior (=) |
| 90 | Cornelius Habarad 2015 | 1 | 6 to 12 years | Male | ASD | Language (+) Problem Behavior (+) |
| 91 | Cox 2017 - A1 | 7 | Mixed Age Groups | Mixed Gender | ASD | Adaptive Behavior (+) |
| 92 | Cox 2017 - A2 | 5 | Mixed Age Groups | Mixed Gender | ASD | Adaptive Behavior (+) |
| 93 | Cox 2017 - A3 | 3 | Mixed Age Groups | Mixed Gender | ASD | Adaptive Behavior (+) |
| 94 | Coyle 2004 | 3 | 6 to 12 years | Male | ASD | Adaptive Behavior (+) |
| 95 | Crowley 2020 | 7 | Mixed Age Groups | Mixed Gender | Mixed Diagnoses | Problem Behavior (+) Adaptive Behavior (+) |
| 96 | Daou 2014 | 3 | Mixed Age Groups | Mixed Gender | ASD | Adaptive Behavior (+) |
| 97 | Dass 2018 | 3 | Mixed Age Groups | Mixed Gender | ASD | Cognitive (+) Language (+) |
| 98 | Dawson 2010 | 45 | 0 to 5 years | Mixed Gender | ASD | Cognitive (+) Language (+) Social/Communication (+) Problem Behavior (=) Adaptive Behavior (+) ASD Symptoms (+) |
| 99 | Day-Watkins 2014 | 3 | 13 to 18 years | Male | ASD | Adaptive Behavior (+) |
| 100 | Demiri 2004 | 5 | Mixed Age Groups | Male | ASD | Problem Behavior (+/-) |
| 101 | DeQuinzio 2007 | 3 | Mixed Age Groups | Unspecified | ASD | Social/Communication (+/-) Emotional (+/-) |
| 102 | DeQuinzio 2015 | 4 | 6 to 12 years | Mixed Gender | ASD | Language (+/-) Adaptive Behavior (+/-) |
| 103 | DeQuinzio 2018 | 3 | 6 to 12 years | Male | ASD | Cognitive (+) Language (+) Social/Communication (+) |
| 104 | DeSouza 2019 | 4 | 0 to 5 years | Mixed Gender | ASD | Cognitive (+) Language (+) |
| 105 | Devlin 2011 | 4 | 6 to 12 years | Male | Mixed Diagnoses | Problem Behavior (+) |
| 106 | Dickes 2015 | 3 | Mixed Age Groups | Mixed Gender | ASD | Language (+/-) |
| 107 | DiGenarro 2007 | 3 | Mixed Age Groups | Mixed Gender | Mixed Diagnoses | Adaptive Behavior (+/-) |
| 108 | Dixon 2001 | 3 | Mixed Age Groups | Male | ASD | Adaptive Behavior (+) |
| 109 | Dixon 2017 | 3 | 0 to 5 years | Mixed Gender | ASD | Cognitive (+) Language (+) |
| 110 | Dixon 2018 - A | 1 | 13 to 18 years | Male | ASD | Cognitive (+) Language (+) |
| 111 | Dixon 2018 - B | 34 | Mixed Age Groups | Mixed Gender | ASD | Cognitive (+) |
| 112 | Doenyas 2014 | 3 | Mixed Age Groups | Male | ASD | Cognitive (+/-) |
| 113 | Dolezal 2010 | 1 | 13 to 18 years | Male | Mixed Diagnoses | Problem Behavior (+) |
| 114 | Dowdy 2018 | 2 | Mixed Age Groups | Male | Mixed Diagnoses | Problem Behavior (+) Adaptive Behavior (+) |
| 115 | Dowdy 2020 | 2 | Mixed Age Groups | Male | Mixed Diagnoses | Problem Behavior (+) |
| 116 | Drasgow 1998 | 3 | 0 to 5 years | Mixed Gender | Mixed Diagnoses | Language (+) |
| 117 | Drasgow 2001 | 1 | 0 to 5 years | Male | ASD | Language (+) |
| 118 | Dufour 2020 | 2 | Mixed Age Groups | Male | ASD | Adaptive Behavior (+) |
| 119 | Dugan 2006 | 7 | 0 to 5 years | Mixed Gender | ASD | Cognitive (=) Adaptive Behavior (=) |
| 120 | Dupere 2013 | 3 | Mixed Age Groups | Mixed Gender | ASD | Language (+/-) |
| 121 | Dupuis 2015 | 1 | 6 to 12 years | Male | Mixed Diagnoses | Problem Behavior (+) |
| 122 | Durand 1999 - A2 | 5 | Mixed Age Groups | Mixed Gender | Mixed Diagnoses | Problem Behavior (+) |
| 123 | Durand 1999 - A3 | 5 | Mixed Age Groups | Mixed Gender | Mixed Diagnoses | Problem Behavior (+) |
| 124 | Easterbrooks 2006 | 1 | 6 to 12 years | Male | Mixed Diagnoses | Problem Behavior (+) |
| 125 | Eby 2010 | 3 | 6 to 12 years | Male | ASD | Cognitive (+) Language (+) |
| 126 | Egan 2009 | 4 | Mixed Age Groups | Male | ASD | Language (+/-) |
| 127 | Eikeseth 2002 | 23 | Mixed Age Groups | Mixed Gender | ASD | Cognitive (+) Language (+) Social/Communication (+) Problem Behavior (+) Adaptive Behavior (+) |
| 128 | Eikeseth 2007 | 25 | Mixed Age Groups | Mixed Gender | ASD | Cognitive (+) Social/Communication (+) Problem Behavior (+) Adaptive Behavior (+) |
| 129 | Eikeseth 2009 - B | 20 | 0 to 5 years | Mixed Gender | ASD | Cognitive (+) Language (=) Adaptive Behavior (=) |
| 130 | Eikeseth 2012 | 59 | Mixed Age Groups | Mixed Gender | ASD | Language (+) Social/Communication (+) Problem Behavior (+) Adaptive Behavior (+) ASD Symptoms (+) |
| 131 | Eldevik 2006 | 28 | 0 to 5 years | Mixed Gender | ASD | Cognitive (+) Language (+) Social/Communication (+) Adaptive Behavior (+/-) ASD Symptoms (+) |
| 132 | Eldevik 2011 | 43 | 0 to 5 years | Mixed Gender | ASD | Cognitive (+) Language (+) Social/Communication (+) Adaptive Behavior (+) |
| 133 | Ellingson 2000 | 2 | Mixed Age Groups | Male | Mixed Diagnoses | Problem Behavior (+) Adaptive Behavior (+) |
| 134 | Elliott 2016 | 3 | 0 to 5 years | Male | ASD | Cognitive (+/-) |
| 135 | Ervin 1998 | 2 | 13 to 18 years | Male | Mixed Diagnoses | Adaptive Behavior (+) |
| 136 | Esch 2009 | 3 | 0 to 5 years | Mixed Gender | ASD | Language (+/-) |
| 137 | Evans 2012 | 100 | Mixed Age Groups | Mixed Gender | ASD | Social/Communication (+/-) |
| 138 | Ezzeddine 2020 | 6 | Mixed Age Groups | Mixed Gender | ASD | Cognitive (+) Language (+) Social/Communication (+) |
| 139 | Fabrizio 2007 | 1 | 0 to 5 years | Male | ASD | Cognitive (+/-) |
| 140 | Falcomata 2010 | 1 | 0 to 5 years | Male | ASD | Problem Behavior (+) |
| 141 | Falcomata 2018 | 2 | 6 to 12 years | Male | ASD | Language (+) Problem Behavior (+) |
| 142 | Farber 2016 | 5 | Mixed Age Groups | Mixed Gender | Mixed Diagnoses | Cognitive (+) |
| 143 | Farrell 2005 | 17 | 0 to 5 years | Mixed Gender | ASD | Cognitive (+) Language (+) Social/Communication (+) Adaptive Behavior (+) |
| 144 | Ferguson 2020 - A | 6 | Mixed Age Groups | Male | ASD | Cognitive (+/-) Language (+/-) Social/Communication (+/-) |
| 145 | Ferguson 2020 - B | 1 | 6 to 12 years | Female | ASD | Social/Communication (+) Problem Behavior (+) Adaptive Behavior (+) Emotional (+) Parent Stress (+) |
| 146 | Fienup 2008 | 1 | 6 to 12 years | Male | ASD | Language (+) |
| 147 | Finn 2012 | 4 | Mixed Age Groups | Male | ASD | Language (+/-) |
| 148 | Fischer 2016 | 4 | Mixed Age Groups | Mixed Gender | ASD | Language (+/-) |
| 149 | Fischer 2010 | 4 | 0 to 5 years | Mixed Gender | ASD | Language (+) |
| 150 | Fisher 2014 | 3 | Mixed Age Groups | Male | ASD | Cognitive (+) |
| 151 | Fisher 2019 - A1 | 4 | 0 to 5 years | Mixed Gender | ASD | Cognitive (+) Language (+) |
| 152 | Fisher 2019 - A2 | 2 | 0 to 5 years | Male | ASD | Cognitive (+) Language (+) |
| 153 | Fisher 2019 - B1 | 2 | 0 to 5 years | Male | ASD | Problem Behavior (+) Emotional (+) |
| 154 | Fisher 2019 - B2 | 2 | Mixed Age Groups | Male | ASD | Problem Behavior (+) Emotional (+) |
| 155 | Flanagan 2012 | 122 | 0 to 5 years | Mixed Gender | ASD | Social/Communication (+) Adaptive Behavior (+) ASD Symptoms (+) |
| 156 | Frampton 2018 | 3 | Mixed Age Groups | Mixed Gender | ASD | Cognitive (+/-) Language (+/-) |
| 157 | Frampton 2020 | 2 | 6 to 12 years | Male | ASD | Language (+) |
| 158 | Francisco 2008 - A2 | 2 | 0 to 5 years | Male | Mixed Diagnoses | Adaptive Behavior (+) |
| 159 | Fritz 2017 | 5 | Mixed Age Groups | Male | Mixed Diagnoses | Language (+) Problem Behavior (+/-) |
| 160 | Fu 2015 | 2 | 6 to 12 years | Male | ASD | Problem Behavior (+/-) |
| 161 | Gale 2011 | 3 | 0 to 5 years | Male | ASD | Problem Behavior (+) |
| 162 | Gamby 2001 | 4 | 0 to 5 years | Male | ASD | Cognitive (+/-) Language (+/-) |
| 163 | Ganz 2008 | 3 | 0 to 5 years | Male | Mixed Diagnoses | Language (+) Social/Communication (+) |
| 164 | Garcia 2016 | 3 | 0 to 5 years | Male | ASD | Adaptive Behavior (+) |
| 165 | Garcia-Albea 2014 | 4 | Mixed Age Groups | Male | ASD | Language (+) Social/Communication (+) |
| 166 | Garfinkle 1999 | 4 | Mixed Age Groups | Male | ASD | Social/Communication (+/-) |
| 167 | Garfinkle 2002 | 4 | 0 to 5 years | Male | Mixed Diagnoses | Social/Communication (+/-) |
| 168 | Gena 2006 | 4 | 0 to 5 years | Mixed Gender | ASD | Language (+) Social/Communication (+) |
| 169 | Gevarter 2016 | 4 | Mixed Age Groups | Male | ASD | Language (+/-) |
| 170 | Ghaemmaghami 2018 - A1 | 2 | Mixed Age Groups | Male | Mixed Diagnoses | Social/Communication (+) Problem Behavior (+) |
| 171 | Ghaemmaghami 2018 - A2 | 2 | Mixed Age Groups | Mixed Gender | Mixed Diagnoses | Social/Communication (+) Problem Behavior (+) |
| 172 | Glodowski 2019 | 4 | 0 to 5 years | Mixed Gender | ASD | Cognitive (+/-) Language (+/-) |
| 173 | Gokey 2013 | 3 | Mixed Age Groups | Mixed Gender | ASD | Problem Behavior (+) Adaptive Behavior (+) |
| 174 | Goldstein 1992 - A | 5 | Mixed Age Groups | Male | Mixed Diagnoses | Language (+/-) Social/Communication (+/-) |
| 175 | Goldstein 1992 - B | 3 | Unspecified | Male | Mixed Diagnoses | Social/Communication (+) |
| 176 | Gomes 2020 | 4 | 0 to 5 years | Mixed Gender | ASD | Cognitive (+) Language (+) Social/Communication (+) |
| 177 | Gongola 2008 | 2 | 6 to 12 years | Mixed Gender | ASD | Problem Behavior (+) |
| 178 | Grannan 2012 | 2 | 0 to 5 years | Male | ASD | Language (+) |
| 179 | Granpeesheh 2009 | 38 | 0 to 5 years | Unspecified | ASD | Cognitive (+) Adaptive Behavior (+) |
| 180 | Green 2002 | 1 | 0 to 5 years | Female | ASD | Cognitive (+) Language (+) Social/Communication (+) Adaptive Behavior (+) |
| 181 | Greener-Wooten 2015 | 3 | Mixed Age Groups | Mixed Gender | ASD | Language (+) Social/Communication (+) |
| 182 | Grey 2005 | 11 | Mixed Age Groups | Mixed Gender | ASD | Cognitive (+) Language (+) Problem Behavior (+) Adaptive Behavior (+) |
| 183 | Grindle 2012 | 11 | Mixed Age Groups | Mixed Gender | ASD | Cognitive (+) Social/Communication (+) Adaptive Behavior (+) |
| 184 | Groskreutz 2010 | 6 | Mixed Age Groups | Mixed Gender | ASD | Cognitive (+) Language (+) |
| 185 | Groskreutz 2014 | 2 | 6 to 12 years | Male | ASD | Language (+) Problem Behavior (+) |
| 186 | Groskreutz 2015 | 1 | 0 to 5 years | Mixed Gender | ASD | Language (+) Social/Communication (+) |
| 187 | Gunby 2010 | 3 | 6 to 12 years | Male | ASD | Adaptive Behavior (+) |
| 188 | Gunby 2014 | 3 | Mixed Age Groups | Mixed Gender | ASD | Adaptive Behavior (+) |
| 189 | Gunby 2018 | 3 | Mixed Age Groups | Mixed Gender | ASD | Cognitive (+) Social/Communication (+) |
| 190 | Gunning 2020 | 7 | Mixed Age Groups | Mixed Gender | ASD | Cognitive (+) Language (+) Social/Communication (+/-) Problem Behavior (+/-) |
| 191 | Guptill 2019 - A1 | 2 | 13 to 18 years | Male | ASD | Language (+) Social/Communication (+) |
| 192 | Guptill 2019 - A2 | 2 | 13 to 18 years | Male | ASD | Language (+) Social/Communication (+) |
| 193 | Gutierrez Jr 2007 | 4 | Mixed Age Groups | Mixed Gender | Mixed Diagnoses | Language (+/-) |
| 194 | Guzinski 2012 | 4 | Mixed Age Groups | Male | ASD | Language (+) Problem Behavior (+/-) |
| 195 | Hanley 2014 | 3 | Mixed Age Groups | Mixed Gender | ASD | Social/Communication (+) Problem Behavior (+) |
| 196 | Harris 1991 | unspecified | 0 to 5 years | Mixed Gender | ASD | Cognitive (+) Language (+) |
| 197 | Hartz 2020 | 1 | 6 to 12 years | Male | Mixed Diagnoses | Problem Behavior (+) |
| 198 | Hatzenbuhler 2019 | 3 | Mixed Age Groups | Mixed Gender | ASD | Language (+) Social/Communication (+) Adaptive Behavior (+/-) |
| 199 | Heldt 2012 | 2 | Mixed Age Groups | Male | Mixed Diagnoses | Language (+) |
| 200 | Hicks 2011 | 2 | 13 to 18 years | Male | Mixed Diagnoses | Language (+) |
| 201 | Hill 2020 | 4 | 6 to 12 years | Mixed Gender | ASD | Cognitive (+) |
| 202 | Hilton 2005 - A1 | 10 | Mixed Age Groups | Mixed Gender | ASD | Language (+) Social/Communication (+) |
| 203 | Hilton 2005 - A2 | 10 | Mixed Age Groups | Mixed Gender | ASD | Cognitive (+/-) Language (+) Social/Communication (+/-) |
| 204 | Hilton 2007 | 2 | 0 to 5 years | Male | Mixed Diagnoses | Social/Communication (-) |
| 205 | Hoch 2002 - A | 3 | 6 to 12 years | Mixed Gender | Mixed Diagnoses | Cognitive (+) Problem Behavior (+) |
| 206 | Hoch 2002 - B | 3 | 6 to 12 years | Male | ASD | Social/Communication (+/-) |
| 207 | Hoffman 2014 | 3 | 6 to 12 years | Male | ASD | Language (+) Problem Behavior (+) |
| 208 | Hood 2017 | 3 | Mixed Age Groups | Mixed Gender | ASD | Social/Communication (+) |
| 209 | Hood 2020 | 3 | 13 to 18 years | Mixed Gender | Mixed Diagnoses | Language (+) Social/Communication (+) |
| 210 | Horner 1997 | 3 | Mixed Age Groups | Male | Mixed Diagnoses | Problem Behavior (+) |
| 211 | Horton 2020 - A1 | 6 | 6 to 12 years | Mixed Gender | ASD | Cognitive (+/-) Language (+/-) Social/Communication (+/-) |
| 212 | Horton 2020 - A2 | 3 | 6 to 12 years | Mixed Gender | ASD | Cognitive (+/-) Language (+/-) Social/Communication (+/-) |
| 213 | Howard 2005 | 61 | 0 to 5 years | Mixed Gender | ASD | Cognitive (+) Language (+) Social/Communication (+) Adaptive Behavior (+) |
| 214 | Howard 2014 | 61 | 0 to 5 years | Mixed Gender | ASD | Cognitive (-) Language (+) Social/Communication (+/-) Adaptive Behavior (-) |
| 215 | Huskens 2012 | 5 | 6 to 12 years | Mixed Gender | ASD | Social/Communication (+) |
| 216 | Hutchinson-Harris 2003 | 79 | 0 to 5 years | Mixed Gender | ASD | Cognitive (+) Language (+) Social/Communication (+) Problem Behavior (+) Adaptive Behavior (+) |
| 217 | Ilg 2018 | 10 | 0 to 5 years | Mixed Gender | Mixed Diagnoses | Language (+/-) Social/Communication (+/-) Problem Behavior (+/-) Adaptive Behavior (+/-) Parent Stress (+/-) |
| 218 | Ingenmey 1991 | 1 | 6 to 12 years | Male | ASD | Language (+/-) |
| 219 | Ingersoll 2001 | 6 | 0 to 5 years | Mixed Gender | ASD | Language (+/-) Social/Communication (+/-) Problem Behavior (+/-) |
| 220 | Ingersoll 2006 | 5 | 0 to 5 years | Mixed Gender | ASD | Cognitive (+/-) Language (+/-) Social/Communication (+/-) |
| 221 | Ingersoll 2007 | 3 | 0 to 5 years | Mixed Gender | ASD | Cognitive (+/-) Social/Communication (+/-) |
| 222 | Ingvarsson 2010 | 4 | Mixed Age Groups | Male | ASD | Language (+) Social/Communication (+) |
| 223 | Jahr 2001 | 5 | Mixed Age Groups | Mixed Gender | ASD | Language (+) |
| 224 | Jahr 2007 | 3 | Mixed Age Groups | Male | ASD | Cognitive (+) Social/Communication (+) Adaptive Behavior (+) |
| 225 | Jansson 2016 | 71 | 0 to 5 years | Mixed Gender | ASD | Adaptive Behavior (=) ASD Symptoms (=) |
| 226 | Jessel 2016 - A2 | 3 | Mixed Age Groups | Male | ASD | Problem Behavior (+) Adaptive Behavior (+) |
| 227 | Jessel 2017 | 1 | 13 to 18 years | Male | ASD | Cognitive (+/-) |
| 228 | Jessel 2018 | 24 | Mixed Age Groups | Mixed Gender | Mixed Diagnoses | Problem Behavior (+) |
| 229 | Jin 2013 | 2 | 6 to 12 years | Male | ASD | Adaptive Behavior (+) |
| 230 | Jones 2006 - A1 | 5 | 0 to 5 years | Male | ASD | Cognitive (+) Social/Communication (+) |
| 231 | Jones 2006 - A2 | 2 | 0 to 5 years | Male | ASD | Cognitive (+) Social/Communication (+) |
| 232 | Jones 2006 - A3 | 4 | 0 to 5 years | Male | ASD | Language (+) Social/Communication (+/-) |
| 233 | Jones 2007 | 2 | 0 to 5 years | Male | Mixed Diagnoses | Language (+) |
| 234 | Jones 2009 | 3 | 0 to 5 years | Mixed Gender | ASD | Cognitive (+) Social/Communication (+) |
| 235 | Jones 2014 | 4 | Mixed Age Groups | Mixed Gender | ASD | Social/Communication (+/-) |
| 236 | Jung 2008 | 3 | Mixed Age Groups | Male | ASD | Social/Communication (+) Problem Behavior (+) |
| 237 | Kahlow 2019 | 3 | Mixed Age Groups | Mixed Gender | ASD | Cognitive (+) Language (+) |
| 238 | Kalgotra 2019 - A | 52 | Mixed Age Groups | Mixed Gender | Mixed Diagnoses | Language (+) |
| 239 | Kalgotra 2019 - B | 70 | Mixed Age Groups | Mixed Gender | Mixed Diagnoses | Social/Communication (+) |
| 240 | Kamio 2015 | 17 | Mixed Age Groups | Mixed Gender | ASD | Cognitive (=) Language (=) Problem Behavior (+/-) ASD Symptoms (=) Parent Stress (=) |
| 241 | Karanth 2010 | 30 | 0 to 5 years | Mixed Gender | ASD | Cognitive (+) Language (+) Social/Communication (+) Adaptive Behavior (+) Emotional (+) |
| 242 | Kay 2020 | 4 | 6 to 12 years | Male | ASD | Cognitive (+) Language (+) |
| 243 | Keen 2001 | 4 | Mixed Age Groups | Mixed Gender | ASD | Language (+/-) Adaptive Behavior (+/-) |
| 244 | Keintz 2011 | 2 | 6 to 12 years | Male | ASD | Cognitive (+/-) |
| 245 | Kelley 2002 | 3 | 6 to 12 years | Mixed Gender | Mixed Diagnoses | Problem Behavior (+/-) |
| 246 | Kelley 2007 | 3 | Mixed Age Groups | Male | Mixed Diagnoses | Language (+/-) |
| 247 | Keohane 2008 | 3 | 6 to 12 years | Mixed Gender | ASD | Cognitive (+) Language (+) Social/Communication (+) Problem Behavior (+) |
| 248 | Kerr 2002 | 3 | 0 to 5 years | Mixed Gender | ASD | Cognitive (+) |
| 249 | Kisamore 2016 | 7 | Mixed Age Groups | Male | ASD | Language (+) |
| 250 | Kittenbrink 2015 | 6 | Mixed Age Groups | Mixed Gender | Mixed Diagnoses | Language (+) Social/Communication (+) |
| 251 | Kobari-Wright 2014 | 4 | 0 to 5 years | Mixed Gender | ASD | Cognitive (+/-) Language (+/-) |
| 252 | Kodak 2003 - B | 2 | 0 to 5 years | Male | ASD | Problem Behavior (+) |
| 253 | Kodak 2012 - B | 2 | Mixed Age Groups | Male | ASD | Language (+) Social/Communication (+) |
| 254 | Kodak 2015 - B | 9 | Mixed Age Groups | Mixed Gender | Mixed Diagnoses | Cognitive (+/-) |
| 255 | Koegel 1992 | 4 | 6 to 12 years | Male | ASD | Social/Communication (+) Adaptive Behavior (+) |
| 256 | Koegel 1993 | 2 | 13 to 18 years | Male | ASD | Social/Communication (+) Adaptive Behavior (+) |
| 257 | Koegel 1998 | 3 | 0 to 5 years | Mixed Gender | ASD | Language (+) Social/Communication (+) |
| 258 | Koegel 1999 - A1 | 6 | 0 to 5 years | Unspecified | ASD | Cognitive (+/-) Language (+/-) Social/Communication (+/-) Problem Behavior (+/-) Adaptive Behavior (+/-) |
| 259 | Koegel 1999 - A2 | 4 | 0 to 5 years | Unspecified | ASD | Cognitive (+) Language (+) Social/Communication (+) Adaptive Behavior (+) ASD Symptoms (+) |
| 260 | Koenig 2010 | 34 | 6 to 12 years | Unspecified | ASD | Social/Communication (+) ASD Symptoms (+) |
| 261 | Kovshoff 2011 | 41 | Mixed Age Groups | Unspecified | ASD | Cognitive (+/-) Language (+) Social/Communication (+/-) Adaptive Behavior (+/-) |
| 262 | Krantz 1998 | 3 | 0 to 5 years | Male | ASD | Cognitive (+) Language (+) Social/Communication (+) |
| 263 | Kroeger 2006 | 1 | 6 to 12 years | Male | Mixed Diagnoses | Language (+) |
| 264 | Kuhn 2008 | 2 | 6 to 12 years | Male | ASD | Social/Communication (+/-) |
| 265 | Kuntz 2020 | 2 | Mixed Age Groups | Mixed Gender | Mixed Diagnoses | Language (+) Social/Communication (+) |
| 266 | Kuoch 2003 | 3 | Mixed Age Groups | Male | ASD | Problem Behavior (+) |
| 267 | Lafasakis 2007 | 3 | 0 to 5 years | Male | Mixed Diagnoses | Cognitive (+) |
| 268 | Lambert 2015 | 53 | Mixed Age Groups | Mixed Gender | Mixed Diagnoses | Cognitive (+) Language (+) Social/Communication (+) Adaptive Behavior (+) |
| 269 | Landa 2017 | 3 | 6 to 12 years | Mixed Gender | ASD | Language (+/-) |
| 270 | Lane 2006 | 1 | 6 to 12 years | Male | Mixed Diagnoses | Problem Behavior (-) |
| 271 | Lang 2009 | 1 | 6 to 12 years | Female | ASD | Social/Communication (+) Problem Behavior (+) |
| 272 | Lanovaz 2011 | 2 | Mixed Age Groups | Mixed Gender | ASD | Problem Behavior (+/-) |
| 273 | Lanovaz 2012 | 4 | Mixed Age Groups | Male | ASD | Problem Behavior (+/-) |
| 274 | Lanovaz 2013 | 1 | 6 to 12 years | Male | ASD | Problem Behavior (+/-) |
| 275 | Lasater 1995 | 2 | 13 to 18 years | Male | Mixed Diagnoses | Adaptive Behavior (+) |
| 276 | Leaf 2012 - B1 | 3 | Mixed Age Groups | Male | ASD | Social/Communication (+/-) |
| 277 | Leaf 2012 - B2 | 2 | Mixed Age Groups | Male | ASD | Social/Communication (+/-) |
| 278 | Leaf 2016 - A | 3 | 0 to 5 years | Mixed Gender | ASD | Social/Communication (+/-) |
| 279 | Leaf 2017 - A | 15 | 0 to 5 years | Unspecified | ASD | Social/Communication (+) Problem Behavior (+) |
| 280 | Leaf 2017 - B | 9 | Mixed Age Groups | Mixed Gender | ASD | Cognitive (+) Language (+) |
| 281 | LeBlanc 2003 - A | 2 | Mixed Age Groups | Male | ASD | Cognitive (+) |
| 282 | LeBlanc 2003 - B | 3 | Mixed Age Groups | Male | ASD | Cognitive (+/-) Language (+/-) |
| 283 | Lechago 2010 | 3 | Mixed Age Groups | Male | ASD | Language (+/-) |
| 284 | Lechago 2013 | 3 | Mixed Age Groups | Male | ASD | Language (+) |
| 285 | Ledbetter-Cho 2015 | 3 | Mixed Age Groups | Male | ASD | Language (+) |
| 286 | Lee 2006 | 3 | 13 to 18 years | Male | ASD | Language (+/-) Social/Communication (+/-) |
| 287 | Leung 1997 - A1 | 1 | 6 to 12 years | Male | ASD | Cognitive (+) Language (+) |
| 288 | Levingston 2009 | 1 | 6 to 12 years | Male | ASD | Cognitive (+) |
| 289 | Liber 2008 | 3 | Mixed Age Groups | Male | ASD | Social/Communication (+) |
| 290 | Loftin 2008 | 3 | 6 to 12 years | Male | ASD | Social/Communication (+) Problem Behavior (+) |
| 291 | Longano 2015 | 2 | 6 to 12 years | Male | ASD | Language (+) |
| 292 | Lora 2020 | 4 | Mixed Age Groups | Mixed Gender | ASD | Cognitive (+) |
| 293 | Loughrey 2014 | 2 | 0 to 5 years | Male | ASD | Cognitive (+) Language (+) |
| 294 | Lovaas 1987 | 40 | 0 to 5 years | Unspecified | ASD | Cognitive (+) |
| 295 | Love 1990 | 2 | Mixed Age Groups | Male | ASD | Social/Communication (+) Problem Behavior (+) Emotional (+) |
| 296 | Luiselli 1999 | 7 | Mixed Age Groups | Male | ASD | Problem Behavior (+) Adaptive Behavior (+) |
| 297 | Luk 2019 | 5 | Mixed Age Groups | Mixed Gender | ASD | Cognitive (+/-) |
| 298 | Lydon 2009 | 2 | 0 to 5 years | Mixed Gender | ASD | Language (+/-) Social/Communication (+/-) |
| 299 | Lyons 2007 | 2 | Mixed Age Groups | Male | Mixed Diagnoses | Problem Behavior (+/-) |
| 300 | MacDonald 2009 | 2 | Mixed Age Groups | Male | ASD | Language (+) Social/Communication (+) |
| 301 | MacDonald 2014 | 148 | 0 to 5 years | Unspecified | ASD | Cognitive (+) Language (+) Social/Communication (+/-) Problem Behavior (+/-) ASD Symptoms (+) |
| 302 | Macduff 2007 | 3 | 0 to 5 years | Male | ASD | Language (+) Social/Communication (+) |
| 303 | Mace 1998 | 1 | 6 to 12 years | Female | Mixed Diagnoses | Problem Behavior (+) |
| 304 | Mace 2011 | 1 | 13 to 18 years | Male | Mixed Diagnoses | Problem Behavior (+/-) |
| 305 | Magee 2001 | 2 | 13 to 18 years | Male | Mixed Diagnoses | Problem Behavior (-) |
| 306 | Magiati 2007 | 44 | 0 to 5 years | Mixed Gender | ASD | Cognitive (+) Language (+/-) Social/Communication (+/-) Adaptive Behavior (+) ASD Symptoms (+) |
| 307 | Magiati 2011 | 36 | 6 to 12 years | Unspecified | ASD | Cognitive (+) Language (+) Social/Communication (+) Adaptive Behavior (+) ASD Symptoms (=) |
| 308 | Maione 2006 | 1 | 0 to 5 years | Male | ASD | Language (+/-) Social/Communication (+/-) |
| 309 | Mancil 2006 | 1 | 0 to 5 years | Male | ASD | Language (+) Problem Behavior (+) |
| 310 | Mancil 2008 | 3 | Mixed Age Groups | Male | ASD | Social/Communication (+) Problem Behavior (+) |
| 311 | Marckel 2006 | 2 | 0 to 5 years | Male | ASD | Cognitive (+) Language (+) |
| 312 | Marion 2012 - A | 3 | Mixed Age Groups | Male | ASD | Cognitive (+/-) Language (+/-) |
| 313 | Marion 2012 - B | 3 | 0 to 5 years | Male | ASD | Cognitive (+/-) Language (+/-) |
| 314 | Marzullo-Kerth 2011 | 4 | 6 to 12 years | Male | ASD | Social/Communication (+/-) Adaptive Behavior (+/-) |
| 315 | Matson 1990 | 3 | 6 to 12 years | Mixed Gender | Mixed Diagnoses | Cognitive (+/-) Language (+/-) |
| 316 | Matsushita 2010 | 1 | 6 to 12 years | Male | ASD | Cognitive (+) Social/Communication (+) Adaptive Behavior (+) |
| 317 | Matsuzaki 2012 | 1 | 0 to 5 years | Male | Mixed Diagnoses | Cognitive (+) Social/Communication (+) |
| 318 | Mazza 2020 | 98 | Mixed Age Groups | Mixed Gender | ASD | Language (-) Social/Communication (+/-) Adaptive Behavior (+/-) ASD Symptoms (+) |
| 319 | McComas 2000 | 3 | 6 to 12 years | Male | Mixed Diagnoses | Problem Behavior (+) |
| 320 | McDonald 2003 | 1 | 13 to 18 years | Male | ASD | Language (+) Social/Communication (+) |
| 321 | McDonald 2012 | 1 | 0 to 5 years | Male | ASD | Problem Behavior (+) Adaptive Behavior (+) |
| 322 | McGarrell 2009 | 6 | 6 to 12 years | Unspecified | ASD | Cognitive (+) Language (+) Problem Behavior (+) Adaptive Behavior (+) |
| 323 | McGee 1992 | 3 | 0 to 5 years | Male | ASD | Social/Communication (+/-) |
| 324 | Meier 2012 | 1 | 0 to 5 years | Female | ASD | Problem Behavior (+/-) |
| 325 | Mello 2018 | 88 | 0 to 5 years | Mixed Gender | Mixed Diagnoses | Cognitive (+) Language (+) Social/Communication (+) Adaptive Behavior (+) ASD Symptoms (+) |
| 326 | Miguel 2009 | 2 | 6 to 12 years | Male | ASD | Cognitive (+) Language (+) |
| 327 | Miguel 2013 | 2 | Mixed Age Groups | Male | ASD | Cognitive (+/-) Language (+) Social/Communication (+) |
| 328 | Miller 2015 | 1 | 0 to 5 years | Male | ASD | Cognitive (+) Social/Communication (+) |
| 329 | Ming 2018 | 2 | 6 to 12 years | Unspecified | #N/A | Cognitive (+) Language (+) |
| 330 | Mitteer 2015 | 1 | 6 to 12 years | Female | ASD | Problem Behavior (+/-) |
| 331 | Moes 2002 | 3 | 0 to 5 years | Mixed Gender | ASD | Social/Communication (+) Problem Behavior (+) |
| 332 | Morrison 2001 | 4 | 6 to 12 years | Mixed Gender | ASD | Social/Communication (+) Problem Behavior (+) |
| 333 | Muething 2018 | 4 | Mixed Age Groups | Mixed Gender | Mixed Diagnoses | Language (+) Problem Behavior (+) |
| 334 | Murphy 2005 - A1 | 4 | Mixed Age Groups | Mixed Gender | Mixed Diagnoses | Cognitive (+/-) Language (+/-) |
| 335 | Murphy 2005 - A2 | 3 | Mixed Age Groups | Mixed Gender | Mixed Diagnoses | Cognitive (+) Language (+) |
| 336 | Murphy 2005 - A3 | 3 | Mixed Age Groups | Mixed Gender | Mixed Diagnoses | Cognitive (+) Language (+) |
| 337 | Murphy 2009 - A1 | 3 | 6 to 12 years | Mixed Gender | ASD | Cognitive (+) Language (+) |
| 338 | Murphy 2009 - A2 | 1 | 6 to 12 years | Male | ASD | Cognitive (+) Language (+) |
| 339 | Murphy 2010 - A | 4 | 13 to 18 years | Male | ASD | Cognitive (+/-) Language (+/-) |
| 340 | Murphy 2010 - B | 3 | 13 to 18 years | Male | ASD | Cognitive (+) Language (+) |
| 341 | Najdowski 2003 | 1 | 0 to 5 years | Male | ASD | Problem Behavior (+) |
| 342 | Najdowski 2008 | 5 | 0 to 5 years | Mixed Gender | ASD | Problem Behavior (=) |
| 343 | Najdowski 2010 | 2 | 0 to 5 years | Mixed Gender | ASD | Problem Behavior (+) |
| 344 | Najdowski 2018 | 3 | Mixed Age Groups | Mixed Gender | ASD | Cognitive (+) Language (+) Social/Communication (+) |
| 345 | Napolitano 2007 | 1 | 6 to 12 years | Male | ASD | Problem Behavior (+) |
| 346 | Napolitano 2010 | 6 | 6 to 12 years | Mixed Gender | ASD | Cognitive (+) |
| 347 | Nasr 2015 | 3 | 6 to 12 years | Male | ASD | Language (+) Social/Communication (+) Problem Behavior (+) |
| 348 | Neely 2019 | 8 | Mixed Age Groups | Unspecified | ASD | Cognitive (+) Language (+) |
| 349 | Neely 2020 - B | 2 | Mixed Age Groups | Male | ASD | Cognitive (+/-) Language (+/-) |
| 350 | Nelson 2007 | 4 | 0 to 5 years | Male | ASD | Social/Communication (+) Adaptive Behavior (+) |
| 351 | Newman 1995 | 3 | 13 to 18 years | Male | ASD | Cognitive (+/-) Adaptive Behavior (+/-) |
| 352 | Newman 1996 | 3 | 13 to 18 years | Male | ASD | Social/Communication (+) Problem Behavior (+) |
| 353 | Newman 1997 | 3 | Mixed Age Groups | Mixed Gender | Mixed Diagnoses | Problem Behavior (+/-) |
| 354 | Newman 2000 | 3 | Mixed Age Groups | Mixed Gender | ASD | Adaptive Behavior (+) |
| 355 | Nikopoulos 2004 | 3 | 6 to 12 years | Male | ASD | Social/Communication (+) |
| 356 | Noel 2016 | 2 | 6 to 12 years | Male | ASD | Problem Behavior (+) |
| 357 | Norman 2001 | 3 | 6 to 12 years | Mixed Gender | Mixed Diagnoses | Adaptive Behavior (+) |
| 358 | Normand 2011 | 2 | 0 to 5 years | Male | ASD | Cognitive (+/-) Adaptive Behavior (+/-) |
| 359 | Nottingham 2017 | 2 | Mixed Age Groups | Mixed Gender | ASD | Cognitive (+/-) |
| 360 | Novack 2018 | 28 | Mixed Age Groups | Mixed Gender | ASD | Language (+) |
| 361 | Nuzzlo-Gomes 2002 - A1 | 1 | 0 to 5 years | Male | ASD | Adaptive Behavior (+) |
| 362 | Nuzzlo-Gomez 2002 - A2 | 3 | Mixed Age Groups | Mixed Gender | ASD | Social/Communication (+) Problem Behavior (+) Adaptive Behavior (+) |
| 363 | O'Connor 2011 - A2 | 10 | 6 to 12 years | Mixed Gender | ASD | Cognitive (+) |
| 364 | O'Connor 2011 - A3 | 5 | Mixed Age Groups | Male | ASD | Cognitive (+) |
| 365 | Ogletree 1995 | 1 | 0 to 5 years | Female | ASD | Cognitive (+/-) Language (+/-) Social/Communication (+/-) |
| 366 | Olsson 2015 | 17 | 6 to 12 years | Mixed Gender | ASD | Language (+/-) Social/Communication (+/-) Problem Behavior (+/-) Adaptive Behavior (+/-) ASD Symptoms (+/-) |
| 367 | Openden 2005 | 37 | Mixed Age Groups | Mixed Gender | ASD | Language (+) Social/Communication (+) |
| 368 | O'Reilly 2012 | 3 | 6 to 12 years | Mixed Gender | ASD | Cognitive (+/-) Language (+/-) |
| 369 | Paden 2012 | 2 | 6 to 12 years | Male | ASD | Language (+) Social/Communication (+) |
| 370 | Pane 2015 | 2 | Mixed Age Groups | Male | ASD | Language (+) Problem Behavior (+) |
| 371 | Park 2020 | 11 | 0 to 5 years | Male | ASD | Cognitive (+/-) Language (+/-) Adaptive Behavior (+/-) ASD Symptoms (+) |
| 372 | Passage 2012 | 1 | 13 to 18 years | Male | Mixed Diagnoses | Adaptive Behavior (+) |
| 373 | Patel 2001 | 1 | 6 to 12 years | Male | ASD | Problem Behavior (+) |
| 374 | Pechous 2000 | 14 | Mixed Age Groups | Mixed Gender | ASD | Social/Communication (+) Adaptive Behavior (+) Parent Stress (-) |
| 375 | Peisley 2020 | 4 | 6 to 12 years | Unspecified | Mixed Diagnoses | Cognitive (+/-) |
| 376 | Penrod 2012 | 2 | 6 to 12 years | Male | ASD | Problem Behavior (+) |
| 377 | Perez 2020 | 11 | Mixed Age Groups | Mixed Gender | ASD | Adaptive Behavior (+/-) |
| 378 | Perez-Gonzalez 2007 | 2 | 6 to 12 years | Unspecified | ASD | Language (+/-) |
| 379 | Perrin 2012 - A1 | 4 | 6 to 12 years | Mixed Gender | ASD | Adaptive Behavior (+/-) |
| 380 | Perrin 2012 - A2 | 3 | 6 to 12 years | Mixed Gender | ASD | Adaptive Behavior (+) |
| 381 | Perry 2008 | 332 | Mixed Age Groups | Mixed Gender | Mixed Diagnoses | Cognitive (+) Social/Communication (+) Adaptive Behavior (+/-) ASD Symptoms (+) |
| 382 | Peters 2015 - A1 | 4 | Mixed Age Groups | Male | Mixed Diagnoses | Language (+/-) Social/Communication (+/-) |
| 383 | Peters 2015 - A2 | 6 | Mixed Age Groups | Mixed Gender | Mixed Diagnoses | Language (+) Social/Communication (+) |
| 384 | Peters 2015 - A3 | 4 | Mixed Age Groups | Mixed Gender | Mixed Diagnoses | Language (+) Social/Communication (+) |
| 385 | Peterson 2016 - A | 6 | Mixed Age Groups | Male | ASD | Problem Behavior (+) |
| 386 | Peterson 2016 - B | 4 | 6 to 12 years | Male | ASD | Cognitive (+/-) |
| 387 | Peterson 2019 - B | 6 | 0 to 5 years | Unspecified | ASD | Problem Behavior (+) Adaptive Behavior (+) |
| 388 | Peters-Scheffer 2010 | 34 | Mixed Age Groups | Mixed Gender | ASD | Social/Communication (+) Problem Behavior (+) Adaptive Behavior (+) ASD Symptoms (+) |
| 389 | Peters-Scheffer 2013 | 40 | Mixed Age Groups | Mixed Gender | ASD | Cognitive (+/-) Language (+) Social/Communication (+/-) Adaptive Behavior (+) Emotional (+) ASD Symptoms (+) Parent Stress (+) |
| 390 | Petursdottir 2007 | 1 | 0 to 5 years | Male | Mixed Diagnoses | Social/Communication (+/-) |
| 391 | Peyton 2005 | 1 | 6 to 12 years | Female | Mixed Diagnoses | Problem Behavior (+) |
| 392 | Phillips 2012 | 3 | 0 to 5 years | Male | Mixed Diagnoses | Cognitive (+/-) |
| 393 | Phillips 2019 - A1 | 3 | Mixed Age Groups | Mixed Gender | ASD | Cognitive (+) |
| 394 | Phillips 2019 - A2 | 2 | Mixed Age Groups | Male | ASD | Cognitive (+) |
| 395 | Pierce 1994 | 3 | 6 to 12 years | Male | ASD | Problem Behavior (+/-) Adaptive Behavior (+/-) |
| 396 | Pierce 1995 | 2 | 6 to 12 years | Male | ASD | Language (+) Social/Communication (+/-) |
| 397 | Pierce 1997 | 2 | 6 to 12 years | Male | ASD | Social/Communication (+) |
| 398 | Pisman 2020 | 2 | 0 to 5 years | Male | ASD | Cognitive (+) Language (+) Social/Communication (+) |
| 399 | Pitts 2019 | 16 | Mixed Age Groups | Mixed Gender | Mixed Diagnoses | Cognitive (+) Language (+) Social/Communication (+) Problem Behavior (+) Adaptive Behavior (+) |
| 400 | Plaisance 2016 | 4 | Mixed Age Groups | Mixed Gender | ASD | Cognitive (+/-) |
| 401 | Plavnick 2010 | 2 | 0 to 5 years | Mixed Gender | Mixed Diagnoses | Social/Communication (+) Adaptive Behavior (+) |
| 402 | Plavnick 2011 - A2 | 4 | Mixed Age Groups | Mixed Gender | Mixed Diagnoses | Language (+/-) |
| 403 | Pollard 2012 | 3 | Mixed Age Groups | Mixed Gender | ASD | Cognitive (+/-) Social/Communication (+/-) Adaptive Behavior (+/-) |
| 404 | Raaymakers 2019 | 3 | Mixed Age Groups | Male | ASD | Cognitive (+/-) Language (+/-) |
| 405 | Rad 2019 | 48 | 0 to 5 years | Mixed Gender | Mixed Diagnoses | Language (+) Social/Communication (+) ASD Symptoms (+) |
| 406 | Rader 2014 | 3 | Mixed Age Groups | Mixed Gender | Mixed Diagnoses | Language (+/-) |
| 407 | Radley 2017 | 5 | 6 to 12 years | Male | ASD | Social/Communication (+/-) |
| 408 | Rapp 2004 - A2 | 3 | Mixed Age Groups | Mixed Gender | Mixed Diagnoses | Problem Behavior (+/-) |
| 409 | Rapp 2004 - A3 | 2 | Mixed Age Groups | Mixed Gender | Mixed Diagnoses | Problem Behavior (+) |
| 410 | Rapp 2007 - A2 | 2 | 6 to 12 years | Male | Mixed Diagnoses | Problem Behavior (+/-) |
| 411 | Reagon 2009 | 3 | Mixed Age Groups | Male | ASD | Language (+) |
| 412 | Reed 2007 - B | 48 | 0 to 5 years | Mixed Gender | ASD | Cognitive (+) Language (+) Social/Communication (+/-) Problem Behavior (+) Adaptive Behavior (+/-) ASD Symptoms (+) |
| 413 | Reed 2012 | 66 | 0 to 5 years | Mixed Gender | ASD | Cognitive (+) Adaptive Behavior (=) |
| 414 | Reeve 2007 | 4 | Mixed Age Groups | Mixed Gender | ASD | Social/Communication (+) Adaptive Behavior (+) |
| 415 | Reinecke 1999 | 3 | 0 to 5 years | Male | Mixed Diagnoses | Social/Communication (+) |
| 416 | Reitzel 2013 | 15 | 6 to 12 years | Unspecified | ASD | Problem Behavior (+) Adaptive Behavior (+/-) |
| 417 | Remington 2007 | 44 | 0 to 5 years | Unspecified | ASD | Cognitive (+) Language (+) Social/Communication (+) Problem Behavior (+) Adaptive Behavior (+) ASD Symptoms (+) |
| 418 | Ribeiro 2020 | 2 | 6 to 12 years | Male | ASD | Cognitive (+) Language (+) |
| 419 | Richman 1997 | 1 | 6 to 12 years | Male | Mixed Diagnoses | Social/Communication (+) Problem Behavior (+) |
| 420 | Richman 2001 | 1 | 13 to 18 years | Male | Mixed Diagnoses | Problem Behavior (+) |
| 421 | Rispoli 2011 | 2 | Mixed Age Groups | Male | Mixed Diagnoses | Cognitive (+) Problem Behavior (+) |
| 422 | Rispoli 2014 | 3 | 0 to 5 years | Male | ASD | Social/Communication (+/-) Problem Behavior (+) |
| 423 | Rivard 2014 - A1 | 37 | 0 to 5 years | Mixed Gender | ASD | Cognitive (-) Language (-) Social/Communication (-) Adaptive Behavior (-) ASD Symptoms (+) |
| 424 | Rivard 2014 - A2 | 93 | 0 to 5 years | Mixed Gender | ASD | Cognitive (+) Language (+) Social/Communication (+) Problem Behavior (+) Adaptive Behavior (+) ASD Symptoms (+) |
| 425 | Riviere 2011 | 2 | 6 to 12 years | Male | ASD | Adaptive Behavior (+) |
| 426 | Roane 2003 | 1 | 6 to 12 years | Male | Mixed Diagnoses | Problem Behavior (+) |
| 427 | Roane 2008 | 1 | 13 to 18 years | Female | Mixed Diagnoses | Problem Behavior (+) |
| 428 | Robinson 2010 | 1 | 13 to 18 years | Male | Mixed Diagnoses | Cognitive (+) Adaptive Behavior (+) |
| 429 | Robinson 2020 | 9 | Mixed Age Groups | Mixed Gender | Mixed Diagnoses | Cognitive (+/-) Language (+/-) Social/Communication (+/-) Adaptive Behavior (+/-) Emotional (+/-) |
| 430 | Romaniuk 2002 | 7 | Mixed Age Groups | Mixed Gender | Mixed Diagnoses | Problem Behavior (+) |
| 431 | Roncati 2019 | 3 | Mixed Age Groups | Male | ASD | Cognitive (+) Language (+) |
| 432 | Rose 2019 | 2 | 0 to 5 years | Mixed Gender | ASD | Social/Communication (+) Problem Behavior (+) |
| 433 | Rozenblat 2019 | 3 | 13 to 18 years | Male | ASD | Cognitive (+) Language (+) Social/Communication (+) |
| 434 | Sansoti 2008 | 3 | 6 to 12 years | Male | ASD | Social/Communication (+) |
| 435 | Sarokoff 2001 | 2 | 6 to 12 years | Male | Mixed Diagnoses | Language (+/-) Social/Communication (+/-) |
| 436 | Sawyer 2005 | 1 | 0 to 5 years | Male | ASD | Social/Communication (+) |
| 437 | Scattone 2008 | 1 | 6 to 12 years | Male | ASD | Language (+) |
| 438 | Scheithauer 2019 | 3 | Mixed Age Groups | Male | Mixed Diagnoses | Problem Behavior (+/-) |
| 439 | Scheithauer 2020 | 24 | Mixed Age Groups | Mixed Gender | ASD | Problem Behavior (+/-) |
| 440 | Schiff 2011 | 1 | 0 to 5 years | Male | ASD | Problem Behavior (+) Adaptive Behavior (+) |
| 441 | Schrandt 2009 | 4 | Mixed Age Groups | Mixed Gender | ASD | Social/Communication (+) Emotional (+) |
| 442 | Schumacher 2011 | 2 | Mixed Age Groups | Mixed Gender | ASD | Problem Behavior (+) |
| 443 | Seaver 2020 - A1 | 7 | Mixed Age Groups | Mixed Gender | Mixed Diagnoses | Language (+/-) |
| 444 | Seaver 2020 - A2 | 7 | Mixed Age Groups | Mixed Gender | Mixed Diagnoses | Language (+/-) |
| 445 | Seaver 2020 - A3 | 7 | Mixed Age Groups | Mixed Gender | Mixed Diagnoses | Language (+/-) |
| 446 | Shabani 2002 | 3 | 6 to 12 years | Male | ASD | Social/Communication (+/-) |
| 447 | Shabani 2006 | 1 | 13 to 18 years | Male | Mixed Diagnoses | Problem Behavior (+) Adaptive Behavior (+) |
| 448 | Sheinkopf 1998 | 22 | 0 to 5 years | Mixed Gender | ASD | Cognitive (+) ASD Symptoms (+) |
| 449 | Shillingsburg 2016 - A1 | 4 | Mixed Age Groups | Mixed Gender | ASD | Language (+) |
| 450 | Shillingsburg 2016 - A2 | 4 | Mixed Age Groups | Mixed Gender | ASD | Language (+) |
| 451 | Shillingsburg 2019 | 3 | Mixed Age Groups | Mixed Gender | ASD | Cognitive (+) Language (+) |
| 452 | Shipley-Benamou 2002 | 3 | 0 to 5 years | Mixed Gender | ASD | Cognitive (+) Adaptive Behavior (+) |
| 453 | Sidener 2005 | 2 | 6 to 12 years | Female | ASD | Problem Behavior (+) |
| 454 | Sigafoos 1996 - A2 | 2 | 6 to 12 years | Male | ASD | Social/Communication (+) Problem Behavior (+) |
| 455 | Silbaugh 2019 | 3 | Mixed Age Groups | Mixed Gender | Mixed Diagnoses | Problem Behavior (+/-) Adaptive Behavior (+/-) |
| 456 | Silla-Zaleski 2010 | 1 | 6 to 12 years | Male | Mixed Diagnoses | Problem Behavior (+) |
| 457 | Sivaraman 2018 | 4 | Mixed Age Groups | Male | ASD | Cognitive (+/-) Language (+/-) |
| 458 | Slifer 2002 | 2 | 6 to 12 years | Male | Mixed Diagnoses | Cognitive (+) Problem Behavior (+) |
| 459 | Slocum 2012 | 1 | 0 to 5 years | Male | ASD | Cognitive (+) |
| 460 | Smith 2000 - A | 6 | 0 to 5 years | Male | ASD | Cognitive (+/-) Language (+/-) Adaptive Behavior (+/-) Parent Stress (+) |
| 461 | Smith 2000 - B | 28 | 0 to 5 years | Mixed Gender | ASD | Cognitive (+) Language (+) Social/Communication (+) Adaptive Behavior (-) |
| 462 | Smith 2015 | 64 | 0 to 5 years | Mixed Gender | ASD | Cognitive (+) Language (+) Social/Communication (+) Problem Behavior (-) Adaptive Behavior (+/-) ASD Symptoms (+) |
| 463 | Somers 2014 | 2 | 6 to 12 years | Male | ASD | Cognitive (+) Language (+) |
| 464 | Stahmer 1992 | 3 | Mixed Age Groups | Mixed Gender | ASD | Problem Behavior (+) Adaptive Behavior (+/-) |
| 465 | Stanislaw 2020 | 61 | Unspecified | Mixed Gender | ASD | Cognitive (+) Language (+) Social/Communication (+) Adaptive Behavior (+) |
| 466 | Stanley 2018 | 3 | 13 to 18 years | Male | ASD | Cognitive (+) |
| 467 | Stasolla 2014 | 2 | 6 to 12 years | Male | ASD | Cognitive (+) Problem Behavior (+) Emotional (+) |
| 468 | Stauch 2018 - A | 5 | 13 to 18 years | Mixed Gender | Mixed Diagnoses | Language (+/-) Social/Communication (+/-) |
| 469 | Stauch 2018 - B1 | 2 | 13 to 18 years | Male | Mixed Diagnoses | Cognitive (+) Language (+) Social/Communication (+/-) Adaptive Behavior (+) |
| 470 | Stauch 2018 - B2 | 3 | 13 to 18 years | Male | Mixed Diagnoses | Cognitive (+) Social/Communication (+/-) Adaptive Behavior (+) |
| 471 | Stauch 2020 | 2 | 13 to 18 years | Male | Mixed Diagnoses | Cognitive (+) Social/Communication (+) Adaptive Behavior (+) |
| 472 | Strain 1994 | 3 | 0 to 5 years | Male | ASD | Social/Communication (+/-) |
| 473 | Stuesser 2020 | 3 | Mixed Age Groups | Mixed Gender | Mixed Diagnoses | Problem Behavior (+) |
| 474 | Suberman 2020 | 3 | 6 to 12 years | Mixed Gender | ASD | Language (+) |
| 475 | Sullivan 2020 - A1 | 3 | Mixed Age Groups | Mixed Gender | ASD | Social/Communication (+/-) Problem Behavior (+/-) |
| 476 | Sullivan 2020 - A2 | 3 | Mixed Age Groups | Mixed Gender | ASD | Social/Communication (+/-) Problem Behavior (+/-) |
| 477 | Susa 2012 | 1 | 6 to 12 years | Male | ASD | Language (+) |
| 478 | Sweeney-Kerwin 2007 | 2 | Mixed Age Groups | Male | ASD | Language (+) |
| 479 | Sy 2012 - A4 | 3 | Mixed Age Groups | Mixed Gender | Mixed Diagnoses | Cognitive (+) |
| 480 | Syed 2018 - A1 | 6 | 6 to 12 years | Male | ASD | Cognitive (+) Language (+) |
| 481 | Syed 2018 - A2 | 2 | 6 to 12 years | Male | ASD | Cognitive (+) Language (+) |
| 482 | Syed 2018 - A3 | 4 | 6 to 12 years | Male | ASD | Cognitive (+) Language (+) |
| 483 | Szabo 2019 | 3 | 6 to 12 years | Male | ASD | Social/Communication (+) Problem Behavior (+) Adaptive Behavior (+) |
| 484 | Szalwinski 2019 | 2 | 13 to 18 years | Mixed Gender | ASD | Cognitive (+) Problem Behavior (+) |
| 485 | Szmacinski 2018 | 2 | 6 to 12 years | Male | ASD | Cognitive (+/-) Language (+/-) |
| 486 | Takeuchi 2002 | 8 | Mixed Age Groups | Mixed Gender | ASD | Cognitive (+/-) |
| 487 | Tarbox 2003 | 1 | 6 to 12 years | Male | ASD | Social/Communication (+) Problem Behavior (+) |
| 488 | Tarbox 2007 | 2 | 0 to 5 years | Male | ASD | Problem Behavior (+) |
| 489 | Tarbox 2009 | 3 | Mixed Age Groups | Male | Mixed Diagnoses | Language (+/-) |
| 490 | Taylor 1995 - A1 | 3 | Mixed Age Groups | Mixed Gender | ASD | Cognitive (+) Language (+) |
| 491 | Taylor 1995 - A2 | 3 | Mixed Age Groups | Mixed Gender | ASD | Language (+/-) |
| 492 | Taylor 1995 - A3 | 3 | Mixed Age Groups | Mixed Gender | ASD | Cognitive (+) Language (+) |
| 493 | Taylor 1998 | 1 | 6 to 12 years | Male | ASD | Language (+) Social/Communication (+) |
| 494 | Taylor 1999 - A1 | 1 | 6 to 12 years | Male | ASD | Language (+/-) |
| 495 | Taylor 1999 - A2 | 1 | 6 to 12 years | Male | ASD | Language (+) Social/Communication (=) |
| 496 | Taylor 2004 | 3 | 13 to 18 years | Mixed Gender | ASD | Adaptive Behavior (+/-) |
| 497 | Taylor 2008 | 3 | Mixed Age Groups | Mixed Gender | ASD | Cognitive (+/-) Social/Communication (+/-) |
| 498 | Taylor 2012 | 3 | 0 to 5 years | Mixed Gender | ASD | Cognitive (+/-) Language (+/-) Social/Communication (+/-) |
| 499 | Taylor 2020 | 1 | 0 to 5 years | Male | ASD | Adaptive Behavior (+) |
| 500 | Taylor-Santa 2014 | 3 | 6 to 12 years | Mixed Gender | ASD | Cognitive (+) |
| 501 | Thiemann 2001 | 5 | 6 to 12 years | Male | ASD | Social/Communication (+/-) |
| 502 | Thiemann 2004 | 5 | 6 to 12 years | Male | ASD | Social/Communication (+/-) |
| 503 | Thorp 1995 | 3 | Mixed Age Groups | Male | ASD | Social/Communication (+/-) Adaptive Behavior (+/-) |
| 504 | Torres 2018 | 3 | Mixed Age Groups | Mixed Gender | ASD | Cognitive (+) Adaptive Behavior (+) |
| 505 | Toussaint 2016 | 3 | 0 to 5 years | Male | ASD | Cognitive (+/-) |
| 506 | Tsami 2019 | 5 | Mixed Age Groups | Mixed Gender | ASD | Language (+/-) Problem Behavior (+/-) |
| 507 | Tsami 2020 | 5 | Mixed Age Groups | Mixed Gender | ASD | Cognitive (+/-) Language (+/-) Problem Behavior (+/-) |
| 508 | Tung 2005 | 6 | Mixed Age Groups | Mixed Gender | ASD | Social/Communication (-) |
| 509 | Twarek 2010 | 3 | 0 to 5 years | Male | ASD | Cognitive (+) Adaptive Behavior (+) |
| 510 | Valentino 2015 | 3 | Mixed Age Groups | Male | ASD | Language (+/-) Social/Communication (+/-) |
| 511 | Valentino 2018 | 1 | 6 to 12 years | Male | ASD | Problem Behavior (+) |
| 512 | Varella 2015 | 1 | 0 to 5 years | Male | ASD | Cognitive (+) Language (+) |
| 513 | Vaughn 1997 | 1 | 6 to 12 years | Male | Mixed Diagnoses | Problem Behavior (+) Adaptive Behavior (+) |
| 514 | Vedora 2016 | 1 | 13 to 18 years | Male | ASD | Cognitive (+) |
| 515 | Vietze 2020 | 106 | 0 to 5 years | Mixed Gender | ASD | Cognitive (+) Language (+) Social/Communication (+) Adaptive Behavior (+) Emotional (+) ASD Symptoms (+/-) |
| 516 | Vismara 2007 | 3 | 0 to 5 years | Male | ASD | Cognitive (+/-) Language (+/-) Social/Communication (+/-) |
| 517 | Vismara 2009 - A | 8 | 0 to 5 years | Unspecified | ASD | Social/Communication (+) |
| 518 | Vismara 2009 - B | 29 | 0 to 5 years | Unspecified | ASD | Cognitive (+) Language (+) Social/Communication (+/-) |
| 519 | Vivanti 2014 | 57 | Mixed Age Groups | Mixed Gender | ASD | Cognitive (+) Language (+) Social/Communication (+/-) Adaptive Behavior (+/-) ASD Symptoms (+) |
| 520 | Vladescu 2016 | 1 | 6 to 12 years | Male | ASD | Language (+) |
| 521 | Volkert 2009 - A1 | 3 | Mixed Age Groups | Mixed Gender | Mixed Diagnoses | Problem Behavior (+/-) |
| 522 | Volkert 2009 - A2 | 3 | Mixed Age Groups | Male | Mixed Diagnoses | Problem Behavior (-) |
| 523 | Vosters 2020 | 3 | Mixed Age Groups | Male | ASD | Cognitive (+) Language (+) |
| 524 | Wacker 2013 | 17 | Mixed Age Groups | Mixed Gender | ASD | Problem Behavior (+) |
| 525 | Waters 2020 | 87 | Mixed Age Groups | Mixed Gender | ASD | Cognitive (+) Language (+) Social/Communication (+) Adaptive Behavior (+) |
| 526 | Watkins 2011 | 2 | 6 to 12 years | Mixed Gender | ASD | Problem Behavior (+) |
| 527 | Watkins 2019 | 4 | Mixed Age Groups | Mixed Gender | ASD | Language (+) Social/Communication (+) |
| 528 | Weiss 1999 | 20 | 0 to 5 years | Mixed Gender | ASD | Cognitive (+/-) Language (+/-) Social/Communication (+/-) Adaptive Behavior (+) ASD Symptoms (+/-) |
| 529 | Weiss 2006 | 20 | 0 to 5 years | Mixed Gender | ASD | Cognitive (+/-) Language (+/-) Social/Communication (+/-) Adaptive Behavior (+/-) ASD Symptoms (+/-) |
| 530 | Welsh 2019 | 3 | Mixed Age Groups | Mixed Gender | ASD | Cognitive (+) Language (+) Social/Communication (+) |
| 531 | Werntz 2019 | 3 | Mixed Age Groups | Mixed Gender | Mixed Diagnoses | Problem Behavior (+) |
| 532 | Whalen 2003 | 5 | 0 to 5 years | Mixed Gender | ASD | Cognitive (+/-) Social/Communication (+/-) |
| 533 | Whalen 2006 - A1 | 8 | 0 to 5 years | Mixed Gender | Mixed Diagnoses | Cognitive (+) Language (+) Social/Communication (+) |
| 534 | Whalen 2006 - A2 | 4 | 0 to 5 years | Unspecified | ASD | Language (+/-) Social/Communication (+/-) Problem Behavior (+/-) |
| 535 | Wheatley 2020 | 1 | 6 to 12 years | Male | Mixed Diagnoses | Problem Behavior (+) |
| 536 | Wichnick-Gillis 2019 | 3 | 6 to 12 years | Male | ASD | Language (+) Social/Communication (+) |
| 537 | Wilder 2020 - A | 2 | 0 to 5 years | Male | ASD | Problem Behavior (=) Adaptive Behavior (+) |
| 538 | Wilder 2020 - B | 3 | Mixed Age Groups | Male | ASD | Problem Behavior (+/-) |
| 539 | Williams 2000 | 2 | 0 to 5 years | Female | ASD | Cognitive (+/-) Language (+/-) Social/Communication (+/-) |
| 540 | Williams 2003 | 3 | Mixed Age Groups | Male | ASD | Cognitive (+/-) Language (+/-) Social/Communication (+/-) |
| 541 | Williams 2005 | 1 | 13 to 18 years | Male | ASD | Cognitive (+) |
| 542 | Williams 2006 | 6 | 6 to 12 years | Mixed Gender | ASD | Cognitive (+/-) Language (+/-) |
| 543 | Wiskow 2013 | 1 | 6 to 12 years | Male | Mixed Diagnoses | Problem Behavior (+) |
| 544 | Wolery 1988 | 3 | 6 to 12 years | Male | ASD | Cognitive (+) Language (+) |
| 545 | Wood 2018 | 32 | 0 to 5 years | Mixed Gender | ASD | Cognitive (+) Language (+) Social/Communication (+) Adaptive Behavior (+) |
| 546 | Yamamoto 2020 | 6 | 13 to 18 years | Mixed Gender | ASD | Language (+) Social/Communication (+) |
| 547 | Young 1994 | 4 | 0 to 5 years | Mixed Gender | ASD | Cognitive (+/-) Social/Communication (+/-) |
| 548 | Zachor 2007 | 39 | 0 to 5 years | Mixed Gender | ASD | Cognitive (+) Language (+) Social/Communication (+) ASD Symptoms (+/-) |
| 549 | Zachor 2010 | 78 | 0 to 5 years | Mixed Gender | ASD | Cognitive (+) Language (+) Social/Communication (+) Adaptive Behavior (+) ASD Symptoms (+/-) |
| 550 | Zambolin 2007 | 1 | 6 to 12 years | Male | ASD | Problem Behavior (+) |
| 551 | Zifferblatt 1977 | 12 | Mixed Age Groups | Male | ASD | Social/Communication (+/-) Adaptive Behavior (+/-) |

Table S2. Extraction data from the Comparison of ABA Techniques group. In the ‘Study Author & Year’ column, records contain letter labels when multiple articles with the same first author were published in the same year. Records contain letter and number labels when extracted articles had multiple parts and were split into discrete study records. In some cases, not all split study records were retained due to meeting exclusion criteria.

|  | Study Author & Year | Total Number of Participants | Age Group | Gender | Diagnosis | Measured Outcome one method improved (+), mixed (+/-), no change (=), no quantifiable measure (x) | Method/Intervention Category |
| --- | --- | --- | --- | --- | --- | --- | --- |
| 1 | Abel 2016 | 1 | 6 to 12 years | Male | ASD | Cognitive (+) Problem Behavior (+) | Reinforcement |
| 2 | Ahearn 2002 | 6 | Mixed Age Groups | Mixed Gender | ASD | Adaptive Behavior (+) | Stimulus Characteristics |
| 3 | Ahrens 2011 - A1 | 2 | Mixed Age Groups | Male | ASD | Language (=) Problem Behavior (=) | Teaching |
| 4 | Ahrens 2011 - A2 | 2 | 0 to 5 years | Male | ASD | Language (+) Problem Behavior (+) Adaptive Behavior (+) | Teaching |
| 5 | Alcalay 2019 | 4 | Mixed Age Groups | Male | ASD | Cognitive (+/-) | Reinforcement |
| 6 | Allison 2012 | 1 | 0 to 5 years | Male | ASD | Problem Behavior (=) Adaptive Behavior (=) | Reinforcement |
| 7 | Arbogast 2019 | 5 | 6 to 12 years | Mixed Gender | ASD | Cognitive (+/-) Language (+/-) Emotional (+/-) | Teaching |
| 8 | Arntzen 2002 | 3 | Mixed Age Groups | Male | Mixed Diagnoses | Language (+) | Teaching |
| 9 | Austin 2015 | 1 | 13 to 18 years | Male | Mixed Diagnoses | Social/Communication (+) Problem Behavior (+) | Reinforcement |
| 10 | Bancroft 2011 | 7 | Mixed Age Groups | Mixed Gender | ASD | Cognitive (+/-) | Teaching |
| 11 | Bernard-Opitz 2004 | 8 | 0 to 5 years | Unspecified | ASD | Social/Communication (+/-) Adaptive Behavior (+/-) | Comparing ABA Interventions |
| 12 | Bishop 2020 | 3 | Mixed Age Groups | Male | ASD | Language (=) | Teaching |
| 13 | Boudreau 2015 | 3 | 6 to 12 years | Unspecified | ASD | Cognitive (+/-) Language (+/-) | Reinforcement |
| 14 | Bowen 2012 | 2 | Mixed Age Groups | Mixed Gender | ASD | Language (=) | Teaching |
| 15 | Breeman 2020 | 1 | 13 to 18 years | Male | ASD | Cognitive (+) Language (+) | Teaching |
| 16 | Buckley 2005 - A | 1 | 0 to 5 years | Male | ASD | Problem Behavior (+) | Reinforcement |
| 17 | Buckley 2005 - A | 1 | 0 to 5 years | Male | ASD | Problem Behavior (+) | Stimulus Characteristics |
| 18 | Buckley 2005 - B | 1 | 6 to 12 years | Male | ASD | Problem Behavior (+) | Reinforcement |
| 19 | Campanaro 2020 | 3 | 6 to 12 years | Mixed Gender | ASD | Cognitive (+/-) | Reinforcement |
| 20 | Campanaro 2020 | 3 | 6 to 12 years | Mixed Gender | ASD | Cognitive (+/-) | Reinforcement |
| 21 | Campanaro 2020 | 3 | 6 to 12 years | Mixed Gender | ASD | Cognitive (+/-) | Reinforcement |
| 22 | Campanaro 2020 | 3 | 6 to 12 years | Mixed Gender | ASD | Cognitive (+/-) | Reinforcement |
| 23 | Carbone 2006 | 1 | 6 to 12 years | Female | ASD | Language (+) | Teaching |
| 24 | Carneiro 2019 | 4 | Mixed Age Groups | Male | ASD | Cognitive (+) Language (+) | Reinforcement |
| 25 | Carp 2012 | 4 | 0 to 5 years | Mixed Gender | ASD | Cognitive (+/-) Language (+/-) | Teaching |
| 26 | Carroll 2013 - A2 | 6 | Mixed Age Groups | Mixed Gender | ASD | Cognitive (+) Language (+) Social/Communication (+) | Teaching |
| 27 | Carroll 2013 - A3 | 3 | Mixed Age Groups | Mixed Gender | ASD | Cognitive (+/-) Language (+/-) | Teaching |
| 28 | Carroll 2013 - A3 | 3 | Mixed Age Groups | Mixed Gender | ASD | Cognitive (+/-) Language (+/-) | Teaching |
| 29 | Carroll 2013 - A3 | 3 | Mixed Age Groups | Mixed Gender | ASD | Cognitive (+/-) Language (+/-) | Teaching |
| 30 | Carroll 2015 - A | 5 | Mixed Age Groups | Mixed Gender | Mixed Diagnoses | Cognitive (+/-) Language (+/-) | Teaching |
| 31 | Carroll 2015 - B | 2 | 0 to 5 years | Male | ASD | Cognitive (+) Language (+) | Teaching |
| 32 | Carroll 2016 | 2 | 0 to 5 years | Male | ASD | Cognitive (+) | Reinforcement |
| 33 | Carroll 2016 | 2 | 0 to 5 years | Male | ASD | Cognitive (+) | Reinforcement |
| 34 | Carroll 2016 | 2 | 0 to 5 years | Male | ASD | Cognitive (+/-) | Reinforcement |
| 35 | Cengher 2020 - A1 | 3 | 0 to 5 years | Male | ASD | Cognitive (+/-) Language (+/-) Social/Communication (+/-) | Reinforcement |
| 36 | Cengher 2020 - A2 | 3 | 0 to 5 years | Male | ASD | Cognitive (+/-) Language (+/-) Social/Communication (+/-) | Reinforcement |
| 37 | Charlop 1986 | 2 | 6 to 12 years | Male | ASD | Social/Communication (+) | Teaching |
| 38 | Charlop-Christy 1996 | 4 | Mixed Age Groups | Mixed Gender | ASD | Problem Behavior (+) | Reinforcement |
| 39 | Charlop-Christy 1996 | 4 | Mixed Age Groups | Mixed Gender | ASD | Problem Behavior (=) | Reinforcement |
| 40 | Cihak 2008 | 3 | 6 to 12 years | Mixed Gender | ASD | Cognitive (+) | Stimulus Characteristics |
| 41 | Cihak 2011 | 4 | Mixed Age Groups | Mixed Gender | ASD | Adaptive Behavior (+/-) | Teaching |
| 42 | Cihon 2020 - B | 27 | Mixed Age Groups | Mixed Gender | ASD | Cognitive (+/-) Language (+/-) | Teaching |
| 43 | Cihon 2020 - B | 27 | Mixed Age Groups | Mixed Gender | ASD | Cognitive (+/-) Language (+/-) | Teaching |
| 44 | Cihon 2020 - B | 27 | Mixed Age Groups | Mixed Gender | ASD | Cognitive (+/-) Language (+/-) | Teaching |
| 45 | Cividini-Motta 2018 | 3 | Mixed Age Groups | Mixed Gender | ASD | Language (=) Social/Communication (=) Problem Behavior (+/-) | Comparing ABA Interventions |
| 46 | Cividini-Motta 2018 | 3 | Mixed Age Groups | Mixed Gender | ASD | Language (=) Social/Communication (=) Problem Behavior (+/-) | Comparing ABA Interventions |
| 47 | Cividini-Motta 2018 | 3 | Mixed Age Groups | Mixed Gender | ASD | Language (=) Social/Communication (=) Problem Behavior (=) | Comparing ABA Interventions |
| 48 | Clark 2004 | 2 | 6 to 12 years | Male | Mixed Diagnoses | Cognitive (+/-) Language (+/-) | Teaching |
| 49 | Clausen 2007 - A1 | 2 | 0 to 5 years | Male | ASD | Cognitive (+/-) Adaptive Behavior (+/-) | Teaching |
| 50 | Clausen 2007 - A2 | 2 | 0 to 5 years | Male | ASD | Cognitive (+/-) Adaptive Behavior (+) | Teaching |
| 51 | Clay 2018 | 1 | 6 to 12 years | Female | ASD | Problem Behavior (+) | Stimulus Characteristics |
| 52 | Clay 2018 | 1 | 6 to 12 years | Female | ASD | Problem Behavior (+) | Stimulus Characteristics |
| 53 | Clay 2018 | 1 | 6 to 12 years | Female | ASD | Problem Behavior (+) | Stimulus Characteristics |
| 54 | Colon 2019 - A2 | 3 | 13 to 18 years | Mixed Gender | ASD | Problem Behavior (+) | Teaching |
| 55 | Colon 2019 - A2 | 3 | 13 to 18 years | Mixed Gender | ASD | Problem Behavior (+/-) | Teaching |
| 56 | Colon 2019 - A2 | 3 | 13 to 18 years | Mixed Gender | ASD | Problem Behavior (+) | Teaching |
| 57 | Cubicciotti 2019 | 3 | Mixed Age Groups | Male | ASD | Cognitive (+/-) Language (+/-) | Stimulus Characteristics |
| 58 | Cubicciotti 2019 | 3 | Mixed Age Groups | Male | ASD | Cognitive (+/-) Language (+/-) | Stimulus Characteristics |
| 59 | Cubicciotti 2019 | 3 | Mixed Age Groups | Male | ASD | Cognitive (+/-) Language (+/-) | Stimulus Characteristics |
| 60 | Cubicciotti 2019 | 3 | Mixed Age Groups | Male | ASD | Cognitive (+/-) Language (+/-) | Stimulus Characteristics |
| 61 | Danov 2010 | 1 | 0 to 5 years | Male | ASD | Social/Communication (+/-) Problem Behavior (+) | Teaching |
| 62 | Davis 2012 | 1 | 0 to 5 years | Male | ASD | Language (+) | Stimulus Characteristics |
| 63 | Davis 2012 | 1 | 0 to 5 years | Male | ASD | Language (+) | Stimulus Characteristics |
| 64 | Delfs 2014 | 4 | Mixed Age Groups | Male | ASD | Language (+/-) | Teaching |
| 65 | DeRosa 2019 | 2 | Mixed Age Groups | Male | ASD | Problem Behavior (+/-) | Comparing ABA Interventions |
| 66 | Deshais 2020 - A1 | 2 | 0 to 5 years | Mixed Gender | ASD | Cognitive (+/-) Social/Communication (+/-) | Teaching |
| 67 | Deshais 2020 - A2 | 2 | 0 to 5 years | Mixed Gender | ASD | Cognitive (+/-) Social/Communication (+/-) | Teaching |
| 68 | Deshais 2020 - A3 | 3 | 0 to 5 years | Male | ASD | Cognitive (+/-) Social/Communication (+/-) | Teaching |
| 69 | Dittlinger 2011 | 3 | Mixed Age Groups | Male | ASD | Cognitive (+) Language (+) | Teaching |
| 70 | Donaldson 2011 | 3 | 0 to 5 years | Male | Mixed Diagnoses | Problem Behavior (=) | Reinforcement |
| 71 | Dozier 2001 | 2 | Mixed Age Groups | Mixed Gender | ASD | Cognitive (+/-) Adaptive Behavior (+/-) | Teaching |
| 72 | Dugan 2006 | 7 | 0 to 5 years | Mixed Gender | ASD | Cognitive (=) Adaptive Behavior (+) | Reinforcement |
| 73 | Dugan 2006 | 7 | 0 to 5 years | Mixed Gender | ASD | Cognitive (+) Adaptive Behavior (+) | Reinforcement |
| 74 | Dwiggins 2008 | 4 | Mixed Age Groups | Male | ASD | Cognitive (+) | Comparing ABA Interventions |
| 75 | Eikeseth 2009 - A | 2 | 0 to 5 years | Male | ASD | Cognitive (+) | Stimulus Characteristics |
| 76 | Fairchild 2020 | 2 | Mixed Age Groups | Male | ASD | Cognitive (+/-) Language (+/-) | Teaching |
| 77 | Fernell 2011 | 198 | 0 to 5 years | Unspecified | ASD | Adaptive Behavior (=) ASD Symptoms (=) | Comparing ABA Interventions |
| 78 | Ferris 2009 | 1 | 0 to 5 years | Male | ASD | Language (+) | Teaching |
| 79 | Fischetti 2012 | 3 | 6 to 12 years | Unspecified | ASD | Problem Behavior (+/-) Adaptive Behavior (+/-) | Comparing ABA Interventions |
| 80 | Fisher 2007 | 2 | 6 to 12 years | Mixed Gender | ASD | Cognitive (+) Language (+) | Teaching |
| 81 | Fisher 2000 | 1 | 13 to 18 years | Male | Mixed Diagnoses | Problem Behavior (+) | Stimulus Characteristics |
| 82 | Fisher 2018 | 4 | Mixed Age Groups | Mixed Gender | Mixed Diagnoses | Problem Behavior (+) | Comparing ABA Interventions |
| 83 | Fiske 2015 | 2 | Mixed Age Groups | Mixed Gender | ASD | Cognitive (+/-) | Reinforcement |
| 84 | Fossett 2006 | 2 | 6 to 12 years | Male | Mixed Diagnoses | Cognitive (+) Language (+) | Teaching |
| 85 | Fragale 2012 | 3 | Mixed Age Groups | Mixed Gender | ASD | Language (+) | Stimulus Characteristics |
| 86 | Francisco 2012 | 2 | 0 to 5 years | Female | Mixed Diagnoses | Social/Communication (+) | Teaching |
| 87 | Fulton 2020 | 3 | Mixed Age Groups | Male | Mixed Diagnoses | Problem Behavior (+/-) | Reinforcement |
| 88 | Gallant 2017 | 4 | 6 to 12 years | Male | ASD | Social/Communication (+/-) | Stimulus Characteristics |
| 89 | Geckeler 2000 | 3 | 6 to 12 years | Male | Mixed Diagnoses | Cognitive (=) | Reinforcement |
| 90 | Geckeler 2000 | 3 | 6 to 12 years | Male | Mixed Diagnoses | Cognitive (+) | Reinforcement |
| 91 | Geiger 2010 | 3 | 6 to 12 years | Male | ASD | Cognitive (+/-) | Teaching |
| 92 | Geiger 2010 | 3 | 6 to 12 years | Male | ASD | Cognitive (+/-) Adaptive Behavior (+/-) | Teaching |
| 93 | Gena 2005 | 3 | 0 to 5 years | Mixed Gender | ASD | Social/Communication (=) | Teaching |
| 94 | Gibbs 2018 | 2 | Mixed Age Groups | Mixed Gender | ASD | Cognitive (+) Problem Behavior (+) | Comparing ABA Interventions |
| 95 | Gorgan 2019 | 3 | Mixed Age Groups | Male | Mixed Diagnoses | Cognitive (+/-) Adaptive Behavior (+/-) | Comparing ABA Interventions |
| 96 | Gorgan 2019 | 3 | Mixed Age Groups | Male | Mixed Diagnoses | Cognitive (+/-) Adaptive Behavior (+/-) | Comparing ABA Interventions |
| 97 | Gorgan 2019 | 3 | Mixed Age Groups | Male | Mixed Diagnoses | Cognitive (+/-) Adaptive Behavior (+/-) | Comparing ABA Interventions |
| 98 | Graff 1998 | 1 | 0 to 5 years | Male | Mixed Diagnoses | Cognitive (+) Social/Communication (+/-) Problem Behavior (+) Adaptive Behavior (+) | Teaching |
| 99 | Graff 1999 | 4 | Mixed Age Groups | Male | Mixed Diagnoses | Cognitive (+) | Reinforcement |
| 100 | Graff 1999 | 4 | Mixed Age Groups | Male | Mixed Diagnoses | Cognitive (+/-) | Reinforcement |
| 101 | Greer 2010 | 3 | Mixed Age Groups | Male | ASD | Language (+/-) | Teaching |
| 102 | Grindle 2002 | 3 | Mixed Age Groups | Mixed Gender | ASD | Cognitive (+) Language (+) | Teaching |
| 103 | Grindle 2005 | 3 | Mixed Age Groups | Male | ASD | Cognitive (+/-) Language (+/-) | Teaching |
| 104 | Groskreutz 2011 | 1 | 0 to 5 years | Male | ASD | Problem Behavior (+) | Stimulus Characteristics |
| 105 | Grow 2011 | 3 | Mixed Age Groups | Mixed Gender | Mixed Diagnoses | Cognitive (+) Language (+) | Teaching |
| 106 | Grow 2014 | 2 | 0 to 5 years | Male | Mixed Diagnoses | Cognitive (+) Language (+) | Teaching |
| 107 | Grow 2016 | 1 | 6 to 12 years | Male | ASD | Cognitive (+) Language (+) | Teaching |
| 108 | Gutierrez Jr 2009 | 3 | 0 to 5 years | Mixed Gender | ASD | Cognitive (+/-) Language (+/-) | Teaching |
| 109 | Hanney 2019 | 2 | 0 to 5 years | Female | ASD | Cognitive (+) Language (+) | Stimulus Characteristics |
| 110 | Hannula 2020 - A1 | 3 | Mixed Age Groups | Mixed Gender | Mixed Diagnoses | Cognitive (+) | Stimulus Characteristics |
| 111 | Hannula 2020 - A2 | 3 | Mixed Age Groups | Male | Mixed Diagnoses | Cognitive (+) | Stimulus Characteristics |
| 112 | Haq 2015 | 3 | Mixed Age Groups | Mixed Gender | ASD | Cognitive (+) Language (+) | Teaching |
| 113 | Haq 2019 | 1 | 6 to 12 years | Male | ASD | Cognitive (+) Problem Behavior (+) | Comparing ABA Interventions |
| 114 | Hayward 2009 | 44 | 0 to 5 years | Mixed Gender | ASD | Cognitive (+) Language (+) Social/Communication (+) Adaptive Behavior (+) | Teaching |
| 115 | Heckaman 1998 | 4 | 6 to 12 years | Male | ASD | Cognitive (+) Language (+) Problem Behavior (+) | Teaching |
| 116 | Heckaman 1998 | 4 | 6 to 12 years | Male | ASD | Cognitive (+/-) Language (+/-) Problem Behavior (+/-) | Stimulus Characteristics |
| 117 | Hedquist 2020 | 3 | 13 to 18 years | Male | ASD | Cognitive (+) Problem Behavior (+) | Reinforcement |
| 118 | Humphreys 2013 | 2 | Mixed Age Groups | Mixed Gender | ASD | Language (+/-) | Stimulus Characteristics |
| 119 | Hundert 2014 | 3 | 0 to 5 years | Mixed Gender | ASD | Social/Communication (+) | Teaching |
| 120 | Huskens 2013 | 6 | 6 to 12 years | Male | ASD | Social/Communication (=) | Teaching |
| 121 | Ingvarsson 2011 | 3 | 0 to 5 years | Male | ASD | Language (+) | Teaching |
| 122 | Jeffries 2016 | 3 | 0 to 5 years | Male | ASD | Social/Communication (+) | Reinforcement |
| 123 | Jessel 2020 | 4 | Mixed Age Groups | Male | ASD | Cognitive (+/-) Language (+/-) | Teaching |
| 124 | Jobin 2020 | 4 | 0 to 5 years | Mixed Gender | #N/A | Language (+/-) Social/Communication (+/-) | Comparing ABA Interventions |
| 125 | Johnson 2004 | 1 | 6 to 12 years | Male | ASD | Language (+/-) Problem Behavior (+/-) | Reinforcement |
| 126 | Jorgenson 2020 | 5 | Mixed Age Groups | Mixed Gender | ASD | Language (+/-) Social/Communication (+/-) | Reinforcement |
| 127 | Jorgenson 2020 | 5 | Mixed Age Groups | Mixed Gender | ASD | Language (+) Social/Communication (+) | Reinforcement |
| 128 | Kalgotra 2019 - B | 70 | Mixed Age Groups | Mixed Gender | Mixed Diagnoses | Social/Communication (+) | Subject/Setting Characteristics |
| 129 | Karsten 2009 | 2 | 0 to 5 years | Male | ASD | Cognitive (+) Language (+) | Reinforcement |
| 130 | Karsten 2009 | 2 | 0 to 5 years | Male | ASD | Cognitive (+/-) Language (+/-) | Reinforcement |
| 131 | Kassardjian 2016 | 6 | Mixed Age Groups | Mixed Gender | ASD | Cognitive (+/-) | Reinforcement |
| 132 | Kelly 1998 | 1 | 0 to 5 years | Male | ASD | Cognitive (+) | Teaching |
| 133 | Keyl-Austin 2012 | 1 | 0 to 5 years | Male | ASD | Cognitive (+) | Stimulus Characteristics |
| 134 | Keyl-Austin 2012 | 1 | 0 to 5 years | Male | ASD | Cognitive (+) | Stimulus Characteristics |
| 135 | Knutson 2019 | 4 | Mixed Age Groups | Male | Mixed Diagnoses | Cognitive (+/-) Language (+/-) Problem Behavior (=) | Teaching |
| 136 | Knutson 2019 | 4 | Mixed Age Groups | Male | Mixed Diagnoses | Cognitive (+/-) Language (+/-) Problem Behavior (=) | Teaching |
| 137 | Knutson 2019 | 4 | Mixed Age Groups | Male | Mixed Diagnoses | Cognitive (+/-) Language (+/-) Problem Behavior (=) | Teaching |
| 138 | Knutson 2019 | 4 | Mixed Age Groups | Male | Mixed Diagnoses | Cognitive (+/-) Language (+/-) Problem Behavior (=) | Teaching |
| 139 | Kodak 2003 - A | 1 | 6 to 12 years | Female | Mixed Diagnoses | Problem Behavior (+) Adaptive Behavior (+/-) | Reinforcement |
| 140 | Kodak 2007 | 3 | Mixed Age Groups | Male | ASD | Cognitive (+) | Reinforcement |
| 141 | Kodak 2009 | 1 | 0 to 5 years | Male | ASD | Language (+) | Teaching |
| 142 | Kodak 2012 - A | 2 | 0 to 5 years | Male | ASD | Language (+) Problem Behavior (+) | Teaching |
| 143 | Kodak 2012 - A | 2 | 0 to 5 years | Male | ASD | Language (+/-) Problem Behavior (+/-) | Teaching |
| 144 | Kodak 2015 - A | 2 | 0 to 5 years | Mixed Gender | Mixed Diagnoses | Cognitive (+/-) Language (+/-) | Teaching |
| 145 | Kodak 2016 | 5 | Mixed Age Groups | Mixed Gender | ASD | Cognitive (+/-) Language (+/-) | Teaching |
| 146 | Kodak 2016 | 5 | Mixed Age Groups | Mixed Gender | ASD | Cognitive (+/-) Language (+/-) | Teaching |
| 147 | Kodak 2016 | 5 | Mixed Age Groups | Mixed Gender | ASD | Cognitive (+/-) Language (+/-) | Teaching |
| 148 | Kodak 2016 | 5 | Mixed Age Groups | Mixed Gender | ASD | Cognitive (+/-) Language (+/-) | Teaching |
| 149 | Kodak 2016 | 5 | Mixed Age Groups | Mixed Gender | ASD | Cognitive (+/-) Language (+/-) | Teaching |
| 150 | Kodak 2020 | 4 | Mixed Age Groups | Male | ASD | Language (+/-) | Stimulus Characteristics |
| 151 | Kodak 2020 | 4 | Mixed Age Groups | Male | ASD | Language (+/-) | Stimulus Characteristics |
| 152 | Kodak 2020 | 4 | Mixed Age Groups | Male | ASD | Language (+/-) | Stimulus Characteristics |
| 153 | Kodak 2020 | 4 | Mixed Age Groups | Male | ASD | Language (+/-) | Stimulus Characteristics |
| 154 | Kovshoff 2011 | 41 | Mixed Age Groups | Unspecified | ASD | Cognitive (+) Language (+) Social/Communication (+) Problem Behavior (+/-) Adaptive Behavior (+) | Reinforcement |
| 155 | Kroeger 2007 | 25 | Mixed Age Groups | Mixed Gender | ASD | Cognitive (=) Language (=) Social/Communication (+/-) Adaptive Behavior (+/-) | Comparing ABA Interventions |
| 156 | Landa 2011 | 45 | 0 to 5 years | Mixed Gender | ASD | Cognitive (+/-) Language (+/-) Social/Communication (+/-) | Teaching |
| 157 | Landa 2016 | 2 | 13 to 18 years | Male | ASD | Cognitive (+) Language (+) | Reinforcement |
| 158 | Landa 2020 | 4 | Mixed Age Groups | Mixed Gender | ASD | Cognitive (+) Language (+) Social/Communication (+) | Teaching |
| 159 | Lang 2010 | 1 | 0 to 5 years | Male | ASD | Problem Behavior (+) | Reinforcement |
| 160 | Lang 2010 | 1 | 0 to 5 years | Male | ASD | Problem Behavior (+) | Reinforcement |
| 161 | Leaf 2010 | 3 | 0 to 5 years | Mixed Gender | ASD | Cognitive (+) | Teaching |
| 162 | Leaf 2012 - A | 6 | Mixed Age Groups | Male | Mixed Diagnoses | Social/Communication (+) | Teaching |
| 163 | Leaf 2014 | 3 | 0 to 5 years | Male | ASD | Cognitive (=) | Reinforcement |
| 164 | Leaf 2014 | 3 | 0 to 5 years | Male | ASD | Cognitive (+) | Reinforcement |
| 165 | Leaf 2014 | 3 | 0 to 5 years | Male | ASD | Cognitive (+) | Reinforcement |
| 166 | Leaf 2016 - B | 3 | Mixed Age Groups | Mixed Gender | ASD | Cognitive (+/-) Language (+/-) | Teaching |
| 167 | Leaf 2020 | 28 | Mixed Age Groups | Unspecified | ASD | Cognitive (+/-) Language (+/-) | Teaching |
| 168 | Lee 2011 | 3 | 13 to 18 years | Male | Mixed Diagnoses | Cognitive (+/-) | Teaching |
| 169 | Lee 2011 | 3 | 13 to 18 years | Male | Mixed Diagnoses | Cognitive (+) | Teaching |
| 170 | Lepper 2013 | 3 | 0 to 5 years | Male | ASD | Language (+/-) | Teaching |
| 171 | Lepper 2013 | 3 | 0 to 5 years | Male | ASD | Language (=) | Teaching |
| 172 | Lim 2011 | 22 | 0 to 5 years | Mixed Gender | ASD | Language (=) | Teaching |
| 173 | Lin 2020 | 4 | 0 to 5 years | Mixed Gender | ASD | Cognitive (+/-) | Teaching |
| 174 | Lindgren 2015 | 107 | Mixed Age Groups | Mixed Gender | Mixed Diagnoses | Language (=) Problem Behavior (=) | Teaching |
| 175 | Lipschultz 2018 | 1 | 0 to 5 years | Male | ASD | Adaptive Behavior (+) | Teaching |
| 176 | Lipschultz 2018 | 1 | 0 to 5 years | Male | ASD | Adaptive Behavior (+) | Teaching |
| 177 | Love 2012 | 2 | 6 to 12 years | Male | ASD | Language (+) Problem Behavior (+/-) | Teaching |
| 178 | Love 2012 | 2 | 6 to 12 years | Male | ASD | Language (+/-) Problem Behavior (+/-) | Teaching |
| 179 | Luiselli 2000 | 16 | 0 to 5 years | Mixed Gender | ASD | Cognitive (=) Social/Communication (=) Adaptive Behavior (=) Emotional (=) | Subject/Setting Characteristics |
| 180 | Lydon 2011 | 5 | Mixed Age Groups | Male | ASD | Social/Communication (+) Adaptive Behavior (+) | Comparing ABA Interventions |
| 181 | Majdalany 2014 | 6 | 0 to 5 years | Male | Mixed Diagnoses | Language (+/-) | Teaching |
| 182 | Majdalany 2016 | 3 | 0 to 5 years | Male | ASD | Cognitive (+/-) Language (+/-) | Reinforcement |
| 183 | Marchese 2012 | 4 | 6 to 12 years | Mixed Gender | ASD | Language (+/-) | Teaching |
| 184 | Marcus 2009 | 3 | Mixed Age Groups | Mixed Gender | ASD | Cognitive (+) Language (+) | Teaching |
| 185 | Matson 1993 | 3 | 0 to 5 years | Male | ASD | Language (=) | Comparing ABA Interventions |
| 186 | McComas 2003 - A2 | 3 | 6 to 12 years | Mixed Gender | Mixed Diagnoses | Problem Behavior (+) | Reinforcement |
| 187 | McComas 2003 - A2 | 3 | 6 to 12 years | Mixed Gender | Mixed Diagnoses | Problem Behavior (=) | Reinforcement |
| 188 | McComas 2003 - A3 | 3 | 6 to 12 years | Male | Mixed Diagnoses | Problem Behavior (=) | Reinforcement |
| 189 | Mello 2018 | 88 | 0 to 5 years | Mixed Gender | Mixed Diagnoses | Cognitive (+/-) Language (+/-) Social/Communication (+) ASD Symptoms (+) | Subject/Setting Characteristics |
| 190 | Miliotis 2012 | 2 | 6 to 12 years | Mixed Gender | ASD | Language (+) | Stimulus Characteristics |
| 191 | Miller 2000 | 5 | Mixed Age Groups | Mixed Gender | Mixed Diagnoses | Cognitive (+) | Reinforcement |
| 192 | Mithaug 2003 | 4 | Mixed Age Groups | Mixed Gender | Mixed Diagnoses | Adaptive Behavior (+) | Teaching |
| 193 | Mohammadzaheri 2014 | 30 | 6 to 12 years | Mixed Gender | ASD | Language (+) Social/Communication (+) | Comparing ABA Interventions |
| 194 | Mohammadzaheri 2015 | 30 | 6 to 12 years | Mixed Gender | ASD | Language (+) Social/Communication (+) Problem Behavior (+) | Comparing ABA Interventions |
| 195 | Morrison 2002 | 4 | 0 to 5 years | Mixed Gender | ASD | Cognitive (+/-) Social/Communication (+/-) | Comparing ABA Interventions |
| 196 | Murphy 2019 | 5 | 6 to 12 years | Mixed Gender | ASD | Cognitive (x) Language (x) | Teaching |
| 197 | Neely 2020 - A | 3 | 0 to 5 years | Mixed Gender | ASD | Social/Communication (+/-) Problem Behavior (+/-) | Subject/Setting Characteristics |
| 198 | Neil 2020 | 3 | 0 to 5 years | Mixed Gender | Mixed Diagnoses | Cognitive (+/-) Language (+/-) | Teaching |
| 199 | Neil 2020 | 3 | 0 to 5 years | Mixed Gender | Mixed Diagnoses | Cognitive (+/-) Language (+/-) | Teaching |
| 200 | Neil 2020 | 3 | 0 to 5 years | Mixed Gender | Mixed Diagnoses | Cognitive (+/-) Language (+/-) | Teaching |
| 201 | Neil 2020 | 3 | 0 to 5 years | Mixed Gender | Mixed Diagnoses | Cognitive (+/-) Language (+/-) | Teaching |
| 202 | Neil 2020 | 3 | 0 to 5 years | Mixed Gender | Mixed Diagnoses | Cognitive (+/-) Language (+/-) | Teaching |
| 203 | Nelson 1980 | 20 | Mixed Age Groups | Mixed Gender | ASD | Cognitive (=) | Teaching |
| 204 | Northgrave 2019 | 2 | Mixed Age Groups | Mixed Gender | ASD | Cognitive (+) Language (+) | Reinforcement |
| 205 | Nottingham 2020 | 2 | 6 to 12 years | Male | ASD | Cognitive (+/-) Language (+/-) | Teaching |
| 206 | Nottingham 2020 | 2 | 6 to 12 years | Male | ASD | Cognitive (+/-) Language (+/-) | Teaching |
| 207 | Nottingham 2020 | 2 | 6 to 12 years | Male | ASD | Cognitive (+/-) Language (+/-) | Teaching |
| 208 | Odluyurt 2012 | 3 | 0 to 5 years | Mixed Gender | Mixed Diagnoses | Cognitive (+/-) | Teaching |
| 209 | Parry-Cruwys 2011 | 6 | Mixed Age Groups | Male | ASD | Cognitive (+/-) | Reinforcement |
| 210 | Partington 1994 | 1 | 6 to 12 years | Female | ASD | Language (+) | Stimulus Characteristics |
| 211 | Penrod 2008 | 4 | Mixed Age Groups | Mixed Gender | Mixed Diagnoses | Cognitive (+/-) Language (+/-) Adaptive Behavior (+/-) | Stimulus Characteristics |
| 212 | Penrod 2008 | 4 | Mixed Age Groups | Mixed Gender | Mixed Diagnoses | Cognitive (+/-) Language (+/-) Adaptive Behavior (+/-) | Stimulus Characteristics |
| 213 | Peterson 2019 - A | 4 | Mixed Age Groups | Mixed Gender | ASD | Cognitive (+/-) | Teaching |
| 214 | Piazza 2002 | 3 | 6 to 12 years | Mixed Gender | Mixed Diagnoses | Adaptive Behavior (+/-) | Stimulus Characteristics |
| 215 | Piper 2020 | 4 | Mixed Age Groups | Unspecified | ASD | Cognitive (+/-) | Reinforcement |
| 216 | Planer 2018 | 3 | 6 to 12 years | Male | Mixed Diagnoses | Adaptive Behavior (+) | Teaching |
| 217 | Planer 2018 | 3 | 6 to 12 years | Male | Mixed Diagnoses | Adaptive Behavior (+/-) | Teaching |
| 218 | Polick 2012 | 2 | 0 to 5 years | Male | ASD | Cognitive (+/-) Language (+/-) | Reinforcement |
| 219 | Polychronis 2004 | 4 | Mixed Age Groups | Male | Mixed Diagnoses | Cognitive (+/-) | Teaching |
| 220 | Protopopova 2020 | 5 | 6 to 12 years | Mixed Gender | ASD | Cognitive (+/-) Social/Communication (+/-) | Reinforcement |
| 221 | Quigley 2018 | 3 | 6 to 12 years | Mixed Gender | Mixed Diagnoses | Language (+/-) Social/Communication (+/-) | Teaching |
| 222 | Quigley 2018 | 3 | 6 to 12 years | Mixed Gender | Mixed Diagnoses | Language (+/-) Social/Communication (+/-) | Teaching |
| 223 | Quigley 2018 | 3 | 6 to 12 years | Mixed Gender | Mixed Diagnoses | Language (+/-) Social/Communication (+/-) | Teaching |
| 224 | Rad 2019 | 48 | 0 to 5 years | Mixed Gender | Mixed Diagnoses | Language (+) Social/Communication (+) ASD Symptoms (+) | Subject/Setting Characteristics |
| 225 | Reed 2007 - A | 27 | 0 to 5 years | Male | ASD | Cognitive (+) Adaptive Behavior (=) ASD Symptoms (+) | Teaching |
| 226 | Reed 2007 - A | 27 | 0 to 5 years | Male | ASD | Cognitive (+/-) Adaptive Behavior (+) | Comparing ABA Interventions |
| 227 | Reeve 2000 | 16 | 0 to 5 years | Mixed Gender | Mixed Diagnoses | Social/Communication (+) Problem Behavior (+) | Teaching |
| 228 | Reichow 2011 | 4 | 0 to 5 years | Mixed Gender | Mixed Diagnoses | Cognitive (+) Language (+) | Teaching |
| 229 | Richardson 2017 - A1 | 4 | Mixed Age Groups | Mixed Gender | ASD | Cognitive (+/-) Language (+/-) | Stimulus Characteristics |
| 230 | Richling 2019 - A1 | 4 | 6 to 12 years | Mixed Gender | Mixed Diagnoses | Cognitive (+) Language (+) | Teaching |
| 231 | Richling 2019 - A1 | 4 | 6 to 12 years | Mixed Gender | Mixed Diagnoses | Cognitive (+/-) Language (+/-) | Teaching |
| 232 | Richling 2019 - A1 | 4 | 6 to 12 years | Mixed Gender | Mixed Diagnoses | Cognitive (+) Language (+) | Teaching |
| 233 | Richling 2019 - A2 | 4 | 6 to 12 years | Mixed Gender | Mixed Diagnoses | Language (+) | Teaching |
| 234 | Richling 2019 - A2 | 4 | 6 to 12 years | Mixed Gender | Mixed Diagnoses | Language (+/-) | Teaching |
| 235 | Richling 2019 - A2 | 4 | 6 to 12 years | Mixed Gender | Mixed Diagnoses | Language (+) | Teaching |
| 236 | Richling 2019 - A3 | 4 | 6 to 12 years | Mixed Gender | Mixed Diagnoses | Language (+) | Teaching |
| 237 | Richling 2019 - A3 | 4 | 6 to 12 years | Mixed Gender | Mixed Diagnoses | Language (+/-) | Teaching |
| 238 | Richling 2019 - A3 | 4 | 6 to 12 years | Mixed Gender | Mixed Diagnoses | Language (+) | Teaching |
| 239 | Ringdahl 2002 | 1 | 6 to 12 years | Female | ASD | Problem Behavior (+) | Teaching |
| 240 | Roane 2005 | 2 | 13 to 18 years | Male | Mixed Diagnoses | Cognitive (+) | Reinforcement |
| 241 | Rodriguez 2012 | 3 | 13 to 18 years | Mixed Gender | ASD | Problem Behavior (+) | Comparing ABA Interventions |
| 242 | Rosales 2010 | 1 | 6 to 12 years | Male | ASD | Cognitive (+) Problem Behavior (+) | Stimulus Characteristics |
| 243 | Rosales 2014 | 2 | Mixed Age Groups | Mixed Gender | ASD | Cognitive (+) Language (+) | Stimulus Characteristics |
| 244 | Russel 2019 | 2 | Mixed Age Groups | Mixed Gender | ASD | Cognitive (+/-) Language (+/-) | Teaching |
| 245 | Saini 2015 | 4 | Mixed Age Groups | Male | ASD | Social/Communication (+/-) Problem Behavior (+) Adaptive Behavior (+/-) | Teaching |
| 246 | Saini 2016 | 3 | Mixed Age Groups | Mixed Gender | ASD | Problem Behavior (+) | Comparing ABA Interventions |
| 247 | Saini 2017 | 3 | Mixed Age Groups | Mixed Gender | ASD | Cognitive (+/-) | Reinforcement |
| 248 | Saini 2017 | 3 | Mixed Age Groups | Mixed Gender | ASD | Cognitive (+/-) | Reinforcement |
| 249 | Sallows 2005 | 23 | 0 to 5 years | Mixed Gender | ASD | Cognitive (+) Language (+) Social/Communication (+) Adaptive Behavior (+) ASD Symptoms (+) | Subject/Setting Characteristics |
| 250 | Saylor 2012 | 2 | Mixed Age Groups | Mixed Gender | ASD | Problem Behavior (+) | Stimulus Characteristics |
| 251 | Schnell 2018 | 3 | Mixed Age Groups | Mixed Gender | ASD | Cognitive (+/-) Language (+/-) | Teaching |
| 252 | Schnell 2018 | 3 | Mixed Age Groups | Mixed Gender | ASD | Cognitive (+/-) Language (+/-) | Teaching |
| 253 | Schnell 2018 | 3 | Mixed Age Groups | Mixed Gender | ASD | Cognitive (+/-) Language (+/-) | Teaching |
| 254 | Schreck 2000 | 5 | Mixed Age Groups | Mixed Gender | ASD | Cognitive (+) Language (+) Social/Communication (+) ASD Symptoms (x) | Subject/Setting Characteristics |
| 255 | Schreck 2000 | 5 | Mixed Age Groups | Mixed Gender | ASD | Cognitive (x) Language (x) Social/Communication (x) ASD Symptoms (x) | Subject/Setting Characteristics |
| 256 | Seaver 2014 - A2 | 6 | Mixed Age Groups | Male | ASD | Cognitive (+/-) | Teaching |
| 257 | Seaver 2014 - A3 | 4 | Mixed Age Groups | Male | ASD | Cognitive (+) | Teaching |
| 258 | Shawler 2020 | 2 | Mixed Age Groups | Mixed Gender | ASD | Social/Communication (+/-) Problem Behavior (+) | Teaching |
| 259 | Shawler 2020 | 2 | Mixed Age Groups | Mixed Gender | ASD | Social/Communication (+/-) Problem Behavior (+) | Teaching |
| 260 | Shawler 2020 | 2 | Mixed Age Groups | Mixed Gender | ASD | Social/Communication (+/-) Problem Behavior (+) | Teaching |
| 261 | Sherer 2001 | 5 | Mixed Age Groups | Male | ASD | Social/Communication (+/-) | Teaching |
| 262 | Slaton 2016 | 2 | Mixed Age Groups | Mixed Gender | Mixed Diagnoses | Cognitive (+) Problem Behavior (+) | Reinforcement |
| 263 | Slaton 2017 - A2 | 4 | Mixed Age Groups | Mixed Gender | Mixed Diagnoses | Social/Communication (+) Problem Behavior (+) | Teaching |
| 264 | Slocum 2011 | 4 | 6 to 12 years | Mixed Gender | Mixed Diagnoses | Cognitive (+/-) | Teaching |
| 265 | Slocum 2015 | 5 | Mixed Age Groups | Mixed Gender | Mixed Diagnoses | Problem Behavior (+) Adaptive Behavior (+) | Reinforcement |
| 266 | Smith 1997 | 21 | 0 to 5 years | Mixed Gender | Mixed Diagnoses | Cognitive (+) Language (+) Problem Behavior (+/-) | Teaching |
| 267 | Smith 2010 | 45 | Mixed Age Groups | Mixed Gender | ASD | Cognitive (+) Language (+) Social/Communication (+) Problem Behavior (+) Adaptive Behavior (+) ASD Symptoms (+) | Subject/Setting Characteristics |
| 268 | Soares 2020 | 10 | Mixed Age Groups | Mixed Gender | ASD | Cognitive (+/-) Problem Behavior (+) | Teaching |
| 269 | Soares 2020 | 10 | Mixed Age Groups | Mixed Gender | ASD | Cognitive (+/-) Problem Behavior (+) | Teaching |
| 270 | Soares 2020 | 10 | Mixed Age Groups | Mixed Gender | ASD | Cognitive (+) Problem Behavior (+/-) | Teaching |
| 271 | Soluaga 2008 | 5 | Mixed Age Groups | Mixed Gender | ASD | Cognitive (+/-) Language (+/-) | Teaching |
| 272 | Stock 2008 | 3 | 0 to 5 years | Mixed Gender | ASD | Language (+/-) | Teaching |
| 273 | Stock 2013 | 28 | 0 to 5 years | Mixed Gender | ASD | Cognitive (+) Language (+/-) Problem Behavior (+) Adaptive Behavior (+) Parent Stress (+/-) | Comparing ABA Interventions |
| 274 | Sy 2012 - A1 | 8 | Mixed Age Groups | Mixed Gender | Mixed Diagnoses | Cognitive (+) | Reinforcement |
| 275 | Sy 2012 - A1 | 8 | Mixed Age Groups | Mixed Gender | Mixed Diagnoses | Cognitive (+) | Reinforcement |
| 276 | Sy 2012 - A1 | 8 | Mixed Age Groups | Mixed Gender | Mixed Diagnoses | Cognitive (=) | Reinforcement |
| 277 | Sy 2012 - A2 | 3 | 0 to 5 years | Mixed Gender | Mixed Diagnoses | Cognitive (+/-) | Reinforcement |
| 278 | Sy 2012 - A3 | 2 | 0 to 5 years | Mixed Gender | Mixed Diagnoses | Cognitive (+/-) | Reinforcement |
| 279 | Thomas 2020 | 2 | 13 to 18 years | Mixed Gender | ASD | Cognitive (+/-) Social/Communication (+/-) Adaptive Behavior (+) | Teaching |
| 280 | Thrailkill 2018 | 1 | 6 to 12 years | Female | ASD | Cognitive (+) | Reinforcement |
| 281 | Tiger 2010 | 3 | Mixed Age Groups | Male | ASD | Cognitive (=) | Reinforcement |
| 282 | Tiger 2010 | 3 | Mixed Age Groups | Male | ASD | Cognitive (+/-) | Reinforcement |
| 283 | Tincani 2004 | 2 | Mixed Age Groups | Mixed Gender | ASD | Language (+/-) Adaptive Behavior (+/-) | Stimulus Characteristics |
| 284 | Toper-Korkmaz 2018 | 3 | Mixed Age Groups | Mixed Gender | ASD | Problem Behavior (+) | Teaching |
| 285 | Toper-Korkmaz 2018 | 3 | Mixed Age Groups | Mixed Gender | ASD | Problem Behavior (+) | Reinforcement |
| 286 | Ulke-Kurkcuoglu 2010 | 4 | Mixed Age Groups | Male | ASD | Cognitive (+) | Teaching |
| 287 | Ulke-Kurkcuoglu 2015 | 3 | Mixed Age Groups | Mixed Gender | ASD | Social/Communication (+/-) | Teaching |
| 288 | Vallinger-Brown 2014 | 3 | Mixed Age Groups | Male | ASD | Language (+/-) | Teaching |
| 289 | Van Laarhoven 2010 | 2 | 13 to 18 years | Male | Mixed Diagnoses | Cognitive (+/-) Adaptive Behavior (+/-) | Teaching |
| 290 | Vedora 2009 | 2 | 6 to 12 years | Male | ASD | Cognitive (+) Language (+) | Teaching |
| 291 | Vedora 2015 | 2 | 0 to 5 years | Male | ASD | Cognitive (+) Language (+) Adaptive Behavior (+) | Teaching |
| 292 | Vedora 2019 | 2 | 13 to 18 years | Male | ASD | Cognitive (+) Language (+) | Stimulus Characteristics |
| 293 | Verriden 2019 | 4 | Mixed Age Groups | Mixed Gender | ASD | Problem Behavior (+) Adaptive Behavior (+) Emotional (=) | Comparing ABA Interventions |
| 294 | Verriden 2019 | 4 | Mixed Age Groups | Mixed Gender | ASD | Problem Behavior (+) Adaptive Behavior (+) Emotional (+/-) | Comparing ABA Interventions |
| 295 | Vietze 2020 | 106 | 0 to 5 years | Mixed Gender | ASD | Cognitive (+) Language (+) Social/Communication (+/-) Adaptive Behavior (+) ASD Symptoms (+) | Subject/Setting Characteristics |
| 296 | Vietze 2020 | 106 | 0 to 5 years | Mixed Gender | ASD | Cognitive (+) Language (+) Social/Communication (+) Adaptive Behavior (+) ASD Symptoms (+) | Comparing ABA Interventions |
| 297 | Vignes 2007 | 2 | 13 to 18 years | Male | ASD | Language (+) | Teaching |
| 298 | Vladescu 2013 | 4 | Mixed Age Groups | Mixed Gender | ASD | Cognitive (+/-) Language (+/-) | Teaching |
| 299 | Volkert 2008 - A1 | 3 | Mixed Age Groups | Male | ASD | Cognitive (+/-) Language (+/-) | Teaching |
| 300 | Volkert 2008 - A2 | 3 | Mixed Age Groups | Male | Mixed Diagnoses | Cognitive (+) Language (+) | Reinforcement |
| 301 | Volkert 2008 - A2 | 3 | Mixed Age Groups | Male | Mixed Diagnoses | Cognitive (+/-) Language (+/-) | Reinforcement |
| 302 | Waldron-Soler 2000 | 3 | 0 to 5 years | Mixed Gender | Mixed Diagnoses | Cognitive (=) | Reinforcement |
| 303 | Waters 2009 | 2 | 6 to 12 years | Male | ASD | Problem Behavior (+) | Comparing ABA Interventions |
| 304 | Watts 2013 | 4 | Mixed Age Groups | Mixed Gender | ASD | Cognitive (+/-) | Teaching |
| 305 | Weston 2020 | 3 | Mixed Age Groups | Male | Mixed Diagnoses | Cognitive (+) | Reinforcement |
| 306 | Woods 2004 | 4 | 6 to 12 years | Mixed Gender | Mixed Diagnoses | Problem Behavior (+) | Reinforcement |
| 307 | Wunderlich 2017 | 5 | Mixed Age Groups | Mixed Gender | Mixed Diagnoses | Cognitive (+/-) Language (+/-) | Teaching |

Table S3. Extraction data from the Between-Groups Comparison group. In the ‘Study Author & Year’ column, records contain letter labels when multiple articles with the same first author were published in the same year. Records contain letter and number labels when extracted articles had multiple parts and were split into discrete study records. In some cases, not all split study records were retained due to meeting exclusion criteria.

|  | Study Author & Year | Total Numbers of Participants | Age | Gender | Diagnosis | Measured Outcome one improved more (+), mixed (+/-), no difference (=), regressed (-) | Intervention that Improved | Compared Intervention |
| --- | --- | --- | --- | --- | --- | --- | --- | --- |
| 1 | Birnbrauer 1993 | 14 | 0-5yrs | Mixed Genders | ASD | Cognitive (+/-) Language (+/-) Social/Communication (+/-) Problem Behavior (+/-) Adaptive Behavior (+/-) Parent Stress (+) | - - - - - EIBI | - - - - - TAU |
| 2 | Cohen 2006 | 42 | 0-5yrs | Mixed Genders | ASD | Cognitive (+) Language (+) Social/Communication (+) Adaptive Behavior (+) | EIBI EIBI EIBI EIBI | TAU TAU TAU TAU |
| 3 | Dawson 2010 | 45 | 0-5yrs | Mixed Genders | ASD | Cognitive (+) Language (+) Social/Communication (+) Problem Behavior (+/-) Adaptive Behavior (+) Autism Symptoms (+/-) | EIBI EIBI EIBI - EIBI - | TAU TAU TAU - TAU - |
| 4 | Devlin 2011 | 4 | 6-12yrs | Male | Mixed Diagnoses | Problem Behavior (+) | ABA | Other Intervention |
| 5 | Dixon 2018 - B | 34 | Mixed Age Groups | Mixed Genders | ASD | Cognitive (+) Language (+) Social/Communication (+) | ABA ABA ABA | TAU TAU TAU |
| 6 | Dugan 2006 | 7 | 0-5yrs | Mixed Genders | ASD | Cognitive (+) Adaptive Behavior (+) | ABA ABA | TAU TAU |
| 7 | Eikeseth 2002 | 23 | Mixed Age Groups | Mixed Genders | ASD | Cognitive (+) Language (+) Social/Communication (+) Adaptive Behavior (+) | I-ABA I-ABA I-ABA I-ABA | TAU TAU TAU TAU |
| 8 | Eikeseth 2007 | 25 | Mixed Age Groups | Mixed Genders | ASD | Cognitive (+) Language (+) Social/Communication (+) Adaptive Behavior (+) | I-ABA I-ABA I-ABA I-ABA | TAU TAU TAU TAU |
| 9 | Eikeseth 2012 | 59 | Mixed Age Groups | Mixed Genders | ASD | Social/Communication (+) Adaptive Behavior (+) | EIBI EIBI | TAU TAU |
| 10 | Eldevik 2006 | 28 | 0-5yrs | Mixed Genders | ASD | Cognitive (+) Language (+) Social/Communication (+) Problem Behavior (+) Adaptive Behavior (-) Autism Symptoms (+) | ABA ABA ABA ABA ABA ABA | TAU TAU TAU TAU TAU TAU |
| 11 | Eldevik 2011 | 43 | 0-5yrs | Mixed Genders | ASD | Cognitive (+) Social/Communication (+) Adaptive Behavior (+) | EIBI EIBI EIBI | TAU TAU TAU |
| 12 | Farrell 2005 | 17 | 0-5yrs | Mixed Genders | ASD | Cognitive (+) Social/Communication (+) Adaptive Behavior (+) | Other Intervention Other Intervention Other Intervention | I-ABA I-ABA I-ABA |
| 13 | Flanagan 2012 | 122 | 0-5yrs | Mixed Genders | ASD | Social/Communication (+) Adaptive Behavior (+) Autism Symptoms (+) | I-ABA I-ABA I-ABA | TAU TAU TAU |
| 14 | Grindle 2012 | 11 | Mixed Age Groups | Mixed Genders | ASD | Cognitive (+) Social/Communication (+) Adaptive Behavior (+) | ABA ABA ABA | TAU TAU TAU |
| 15 | Hilton 2005 - A1 | 10 | Mixed Age Groups | Mixed Genders | ASD | Language (+) Social/Communication (+/-) | ABA - | DIR - |
| 16 | Hilton 2005 - A2 | 10 | Mixed Age Groups | Mixed Genders | ASD | Cognitive (+/-) Language (+/-) Social/Communication (+/-) Adaptive Behavior (+/-) | - - - - | - - - - |
| 17 | Hilton 2007 | 2 | 0-5yrs | Male | Mixed Diagnoses | Language (+/-) Social/Communication (+/-) Problem Behavior (+) Adaptive Behavior (+/-) | - - DIR - | - - ABA - |
| 18 | Howard 2005 | 61 | 0-5yrs | Mixed Genders | ASD | Cognitive (+) Language (+) Social/Communication (+) Adaptive Behavior (+/-) | I-ABA I-ABA I-ABA - | Other Intervention Other Intervention Other Intervention - |
| 19 | Howard 2005 | 61 | 0-5yrs | Mixed Genders | ASD | Cognitive (+) Language (+) Social/Communication (+) Adaptive Behavior (+/-) | I-ABA I-ABA I-ABA - | Other Intervention Other Intervention Other Intervention - |
| 20 | Howard 2014 | 61 | 0-5yrs | Mixed Genders | ASD | Cognitive (+) Language (+) Social/Communication (+) Adaptive Behavior (-) | Other Intervention I-ABA Other Intervention Other Intervention | I-ABA Other Intervention I-ABA I-ABA |
| 21 | Howard 2014 | 61 | 0-5yrs | Mixed Genders | ASD | Cognitive (+) Language (+) Social/Communication (+) Adaptive Behavior (-) | I-ABA TAU TAU I-ABA | TAU I-ABA I-ABA TAU |
| 22 | Jansson 2016 | 71 | 0-5yrs | Mixed Genders | ASD | Adaptive Behavior (=) Autism Symptoms (=) | - - | - - |
| 23 | Kalgotra 2019 - A | 52 | Mixed Age Groups | Mixed Genders | Mixed Diagnoses | Cognitive (+) Language (+) Social/Communication (+) | ABA ABA ABA | TAU TAU TAU |
| 24 | Kalgotra 2019 - A | 52 | Mixed Age Groups | Mixed Genders | Mixed Diagnoses | Cognitive (+) Language (+) Social/Communication (+) | ABA ABA ABA | TAU TAU TAU |
| 25 | Kalgotra 2019 - B | 70 | Mixed Age Groups | Mixed Genders | Mixed Diagnoses | Social/Communication (+) | ABA | TAU |
| 26 | Kalgotra 2019 - B | 70 | Mixed Age Groups | Mixed Genders | Mixed Diagnoses | Social/Communication (+) | ABA | TAU |
| 27 | Kamio 2015 | 17 | Mixed Age Groups | Mixed Genders | ASD | Cognitive (=) Language (=) Social/Communication (=) Problem Behavior (=) Emotional (=) Autism Symptoms (=) Parent Stress (=) | - - - - - - - | - - - - - - - |
| 28 | Kamio 2015 | 17 | Mixed Age Groups | Mixed Genders | ASD | Cognitive (=) Language (=) Social/Communication (=) Problem Behavior (=) Emotional (=) Autism Symptoms (=) Parent Stress (=) | - - - - - - - | - - - - - - - |
| 29 | Koenig 2010 | 34 | 6-12yrs | Unspecified | ASD | Social/Communication (=) Autism Symptoms (+) | - ABA | - TAU |
| 30 | Kovshoff 2011 | 41 | Mixed Age Groups | Unspecified | ASD | Cognitive (+) Language (+) Social/Communication (+) Problem Behavior (+) Adaptive Behavior (+) | EIBI EIBI EIBI TAU EIBI | TAU TAU TAU EIBI TAU |
| 31 | Leaf 2017 - A | 15 | 0-5yrs | Unspecified | ASD | Social/Communication (+) Problem Behavior (+) | ABA ABA | TAU TAU |
| 32 | Lovaas 1987 | 40 | 0-5yrs | Unspecified | ASD | Cognitive (+) | I-ABA | TAU |
| 33 | Magiati 2007 | 44 | 0-5yrs | Mixed Genders | ASD | Cognitive (=) Language (=) Social/Communication (=) Adaptive Behavior (+/-) Autism Symptoms (=) | - - - - - | - - - - - |
| 34 | Mazza 2020 | 98 | Mixed Age Groups | Mixed Genders | ASD | Language (+) Social/Communication (+) Problem Behavior (=) Adaptive Behavior (+) Autism Symptoms (=) | Other Intervention Other Intervention - Other Intervention - | ABA ABA - ABA - |
| 35 | Mazza 2020 | 98 | Mixed Age Groups | Mixed Genders | ASD | Language (+) Social/Communication (+) Problem Behavior (=) Adaptive Behavior (+) Autism Symptoms (=) | Other Intervention Other Intervention - Other Intervention - | ABA ABA - ABA - |
| 36 | Novack 2018 | 28 | Mixed Age Groups | Mixed Genders | ASD | Cognitive (+) Language (+) | ABA ABA | TAU TAU |
| 37 | Openden 2005 | 37 | Mixed Age Groups | Mixed Genders | ASD | Language (+) Social/Communication (+) | ABA ABA | TAU TAU |
| 38 | Pechous 2000 | 14 | Mixed Age Groups | Mixed Genders | ASD | Adaptive Behavior (+) Parent Stress (+) | I-ABA TAU | TAU I-ABA |
| 39 | Peterson 2016 - A | 6 | Mixed Age Groups | Male | ASD | Adaptive Behavior (+) | ABA | Other Intervention |
| 40 | Peterson 2019 - B | 6 | 0-5yrs | Unspecified | ASD | Problem Behavior (+) Adaptive Behavior (+) | ABA ABA | TAU TAU |
| 41 | Peters-Scheffer 2010 | 34 | Mixed Age Groups | Mixed Genders | ASD | Cognitive (+) Social/Communication (+) Problem Behavior (+) Adaptive Behavior (+) Autism Symptoms (+) | ABA ABA TAU ABA TAU | TAU TAU ABA TAU ABA |
| 42 | Peters-Scheffer 2013 | 40 | Mixed Age Groups | Mixed Genders | ASD | Cognitive (+) Language (+) Social/Communication (+/-) Adaptive Behavior (+) Emotional (+) Autism Symptoms (+) Parent Stress (=) | ABA ABA - ABA ABA ABA - | TAU TAU - TAU TAU TAU - |
| 43 | Reed 2007 - B | 48 | 0-5yrs | Mixed Genders | ASD | Cognitive (+) Language (+) Social/Communication (+/-) Problem Behavior (+/-) Adaptive Behavior (+/-) Autism Symptoms (+) | I-ABA I-ABA - - - Nursery | Nursery Nursery - - - I-ABA |
| 44 | Reed 2007 - B | 48 | 0-5yrs | Mixed Genders | ASD | Cognitive (+) Language (+) Social/Communication (+/-) Problem Behavior (+/-) Adaptive Behavior (+) Autism Symptoms (+) | I-ABA I-ABA - - I-ABA I-ABA | Portage Portage - - Portage Portage |
| 45 | Reed 2012 | 66 | 0-5yrs | Mixed Genders | ASD | Cognitive (+) Adaptive Behavior (+) | ABA Nursery | Nursery ABA |
| 46 | Reed 2012 | 66 | 0-5yrs | Mixed Genders | ASD | Cognitive (+) Adaptive Behavior (+) | ABA ABA | Portage Portage |
| 47 | Reed 2012 | 66 | 0-5yrs | Mixed Genders | ASD | Cognitive (+) Adaptive Behavior (+) | ABA ABA | TAU TAU |
| 48 | Reitzel 2013 | 15 | 6-12yrs | Unspecified | ASD | Social/Communication (+) Problem Behavior (+) Adaptive Behavior (+/-) | TAU ABA - | ABA TAU - |
| 49 | Remington 2007 | 44 | 0-5yrs | Unspecified | ASD | Cognitive (+) Language (+) Social/Communication (+) Problem Behavior (+/-) Adaptive Behavior (+) Autism Symptoms (+/-) | EIBI EIBI EIBI - EIBI - | TAU TAU TAU - TAU - |
| 50 | Scheithauer 2020 | 24 | Mixed Age Groups | Mixed Genders | ASD | Problem Behavior (+) Parent Stress (-) | ABA - | TAU - |
| 51 | Sheinkopf 1998 | 22 | 0-5yrs | Mixed Genders | ASD | Cognitive (+) Autism Symptoms (+) | I-ABA I-ABA | TAU TAU |
| 52 | Smith 2000 - B | 28 | 0-5yrs | Mixed Genders | ASD | Cognitive (+) Language (+) Social/Communication (+) Adaptive Behavior (-) | I-ABA I-ABA I-ABA - | ABA ABA ABA - |
| 53 | Stanislaw 2020 | 61 | Unspecified | Mixed Genders | ASD | Cognitive (+) Language (+) Social/Communication (+) Adaptive Behavior (+) | ABA ABA ABA ABA | Other Intervention Other Intervention Other Intervention Other Intervention |
| 54 | Tung 2005 | 6 | Mixed Age Groups | Mixed Genders | ASD | Social/Communication (+) | TAU | ABA |
| 55 | Vivanti 2014 | 57 | Mixed Age Groups | Mixed Genders | ASD | Cognitive (+) Language (+) Social/Communication (+) Adaptive Behavior (+/-) Autism Symptoms (+) | EIBI EIBI TAU - TAU | TAU TAU EIBI - EIBI |
| 56 | Waters 2020 | 87 | Mixed Age Groups | Mixed Genders | ASD | Cognitive (+) Language (+) Social/Communication (+) Adaptive Behavior (+) | EIBI EIBI EIBI EIBI | TAU TAU TAU TAU |
| 57 | Zachor 2007 | 39 | 0-5yrs | Mixed Genders | ASD | Cognitive (+) Language (+) Social/Communication (+) Autism Symptoms (+) | ABA ABA ABA ABA | Other Intervention Other Intervention Other Intervention Other Intervention |
| 58 | Zachor 2010 | 78 | 0-5yrs | Mixed Genders | ASD | Cognitive (+/-) Language (+) Social/Communication (+/-) Adaptive Behavior (+/-) Autism Symptoms (+/-) | - Other Intervention - - - | - ABA - - - |
