# Supplementary material for: Applied Behavior Analysis in Children and Youth with Autism Spectrum Disorders: A Scoping Review
Source: Perspect Behav Sci. 2022 May 18;45(3):521–57. doi: 10.1007/s40614-022-00338-x (PMC9458805; doi:10.1007/s40614-022-00338-x)
Supplement: Supplementary file 4 — (DOCX 110 kb) [file 40614_2022_338_MOESM4_ESM.docx]

Appendix 4. References – Extracted Articles

Abel, E. A., Gadomski, M., & Brodhead, M. T. (2016). A brief report of time-on-task behavior in a child with autism: Comparing material and social reinforcement in the home environment. *Psi Chi Journal of Psychological Research*, *21*(3), 176–182. https://doi.org/10.24839/b21.3.176

Ahearn, W. H. (2003). Using simultaneous presentation to increase vegetable consumption in a mildly selective child with autism. *Journal of Applied Behavior Analysis*, *36*(3), 361–365. https://doi.org/10.1901/jaba.2003.36-361

Ahearn, W. H. (2002). Effect of two methods of introducing foods during feeding treatment on acceptance of previously rejected items. *Behavioral Interventions*, *17*(3), 111–127. https://doi.org/10.1002/bin.112

Ahearn, W. H., Clark, K. M., MacDonald, R. P. F., & Chung, B. I. (2007). Assessing and treating vocal stereotypy in children with autism. *Journal of Applied Behavior Analysis*, *40*(2), 263–275. https://doi.org/10.1901/jaba.2007.30-06

Ahrens, E. N., Lerman, D. C., Kodak, T., Worsdell, A. S., & Keegan, C. (2011). Further evaluation of response interruption and redirection as treatment for stereotypy. *Journal of Applied Behavior Analysis*, *44*(1), 95–108. https://doi.org/10.1901/jaba.2011.44-95

Akers, J. S., Higbee, T. S., Gerencser, K. R., & Pellegrino, A. J. (2018). An evaluation of group activity schedules to promote social play in children with autism. *Journal of Applied Behavior Analysis*, *51*(3), 553–570. https://doi.org/10.1002/jaba.474

Akers, J. S., Higbee, T. S., Pollard, J. S., Pellegrino, A. J., & Gerencser, K. R. (2016). An evaluation of photographic activity schedules to increase independent playground skills in young children with autism. *Journal of Applied Behavior Analysis*, *49*(4), 954–959. https://doi.org/10.1002/jaba.327

Alcalay, A., Ferguson, J. L., Cihon, J. H., Torres, N., Leaf, J. B., Leaf, R., McEachin, J., Schulze, K. A., & Rudrud, E. H. (2019). Comparing multiple stimulus preference assessments without replacement to in-the-moment reinforcer analysis on rate of responding. *Education and Training in Autism and Developmental Disabilities*, *54*(1), 69–82.

Alcantara, P. R. (1994). Effects of Videotape Instructional Package on Purchasing Skills of Children with Autism. *Exceptional Children*, *61*(1), 40–55. https://doi.org/10.1177/001440299406100105

Allan, A. C., Vladescu, J. C., Kisamore, A. N., Reeve, S. A., & Sidener, T. M. (2015). Evaluating the Emergence of Reverse Intraverbals in Children with Autism. *The Analysis of Verbal Behavior*, *31*(1), 59–75. https://doi.org/10.1007/s40616-014-0025-8

Allison, J., Wilder, D. A., Chong, I., Lugo, A., Pike, J., & Rudy, N. (2012). A comparison of differential reinforcement and noncontingent reinforcement to treat food selectivity in a child with autism. *Journal of Applied Behavior Analysis*, *45*(3), 613–617. https://doi.org/10.1901/jaba.2012.45-613

Almon-Morris, H., & Diakite, A. (2007). Teaching Emotions to Children with Autism: Identification, Demonstration, and Explanation of Occasioning Stimuli. *Journal of Precision Teaching and Celeration*, *23*, 19–22.

Alsharif, S. (2020). Establishing the correspondence between listening to one’s own voice and doing in children with autism spectrum disorder [ProQuest Information & Learning]. In *Dissertation Abstracts International: Section B: The Sciences and Engineering* (Vol. 81, Issues 12-B).

Alzrayer, N. M. (2020). The impact of an intraverbal webbing procedure on the emergence of advanced intraverbal skills in children with autism spectrum disorder. *Behavior Analysis in Practice*, *13*(4), 914–923. https://doi.org/10.1007/s40617-020-00410-5

Anderson, C. M., & Long, E. S. (2002). Use of a structured descriptive assessment methodology to identify variables affecting problem behavior. *Journal of Applied Behavior Analysis*, *35*(2), 137–154. https://doi.org/10.1901/jaba.2002.35-137

Anderson, C. M., & McMillan, K. (2001). Parental use of escape extinction and differential reinforcement to treat food selectivity. *Journal of Applied Behavior Analysis*, *34*(4), 511–515. https://doi.org/10.1901/jaba.2001.34-511

Anderson, E., Barretto, A., McLaughlin, T. F., & McQuaid, T. (2016). Effects of functional communication training with and without delays to decrease aberrant behaviour in a child with autism spectrum disorder. *Journal on Developmental Disabilities*, *22*(1), 101–110.

Anderson, S. R., Avery, D. L., Dipietro, E. K., Edwards, G. L., & Christian, W. P. (1987). Intensive home-based early intervention with autistic children. *Education and Treatment of Children*, *10*(4), 352–366.

Apple, A. L., Billingsley, F., Schwartz, I. S., & Carr, E. G. (2005). Effects of video modeling alone and with self-management on compliment-giving behaviors of children with high-functioning ASD. *Journal of Positive Behavior Interventions*, *7*(1), 33–46. https://doi.org/10.1177/10983007050070010401

Aravamudhan, S., & Awasthi, S. (2020). Behavioral interventions to treat speech sound disorders in children with autism. *Behavior Analysis in Practice*, *13*(1), 174–185. https://doi.org/10.1007/s40617-019-00362-5

Arbogast, N. R. (2019). The effects of speaker and listener training on prosodic skills of children with autism spectrum disorder [ProQuest Information & Learning]. In *Dissertation Abstracts International: Section B: The Sciences and Engineering* (Vol. 80, Issues 8-B(E)).

Argyropoulou, Z., & Papoudi, D. (2012). The training of a child with autism in a Greek preschool inclusive class through intensive interaction: A case study. *European Journal of Special Needs Education*, *27*(1), 99–114. https://doi.org/10.1080/08856257.2011.640489

Armstrong, A., Knapp, V. M., & McAdam, D. B. (2014). Functional analysis and treatment of the diurnal bruxism of a 16‐year‐aold girl with autism. *Journal of Applied Behavior Analysis*, *47*(2), 415–419. https://doi.org/10.1002/jaba.122

Arntzen, E., & Almås, I. K. (2002). Effects of mand-tact versus tact-only training on the acquisition of tacts. *Journal of Applied Behavior Analysis*, *35*(4), 419–422. https://doi.org/10.1901/jaba.2002.35-419

Asmus, J. M., Wacker, D. P., Harding, J., Berg, W. K., Derby, K. M., & Kocis, E. (1999). Evaluation of antecedent stimulus parameters for the treatment of escape-maintained aberrant behavior. *Journal of Applied Behavior Analysis*, *32*(4), 495–513. https://doi.org/10.1901/jaba.1999.32-495

Athens, E. S., Vollmer, T. R., Sloman, K. N., & Pipkin, C. S. P. (2008). An analysis of vocal stereotypy and therapist fading. *Journal of Applied Behavior Analysis*, *41*(2), 291–297. https://doi.org/10.1901/jaba.2008.41-291

Austin, J. E., & Tiger, J. H. (2015). Providing alternative reinforcers to facilitate tolerance to delayed reinforcement following functional communication training. *Journal of Applied Behavior Analysis*, *48*(3), 663–668. https://doi.org/10.1002/jaba.215

Axe, J. B., & Sainato, D. M. (2010). Matrix training of preliteracy skills with preschoolers with autism. *Journal of Applied Behavior Analysis*, *43*(4), 635–652. https://doi.org/10.1901/jaba.2010.43-635

Baker, M. J. (2000). Incorporating the Thematic Ritualistic Behaviors of Children with Autism into Games: Increasing Social Play Interactions with Siblings. *Journal of Positive Behavior Interventions*, *2*(2), 66–84. https://doi.org/10.1177/109830070000200201

Bancroft, S. L., Weiss, J. S., Libby, M. E., & Ahearn, W. H. (2011). A comparison of procedural variations in teaching behavior chains: Manual guidance, trainer completion, and no completion of untrained steps. *Journal of Applied Behavior Analysis*, *44*(3), 559–569. https://doi.org/10.1901/jaba.2011.44-559

Barkaia, A., Stokes, T. F., & Mikiashvili, T. (2017). Intercontinental telehealth coaching of therapists to improve verbalizations by children with autism. *Journal of Applied Behavior Analysis*, *50*(3), 582–589. https://doi.org/10.1002/jaba.391

Barnoy, E. L., Najdowski, A. C., Tarbox, J., Wilke, A. E., & Nollet, M. D. (2009). Evaluation of a multicomponent intervention for diurnal bruxism in a young child with autism. *Journal of Applied Behavior Analysis*, *42*(4), 845–848. https://doi.org/10.1901/jaba.2009.42-845

Bartman, S., & Freeman, N. (2003). Teaching Language to a Two-Year-Old with Autism. *Journal on Developmental Disabilities*, *10*(1), 47–53.

Beaulieu, L., Van Nostrand, M. E., Williams, A. L., & Herscovitch, B. (2018). Incorporating interview-informed functional analyses into practice. *Behavior Analysis in Practice*, *11*(4), 385–389. https://doi.org/10.1007/s40617-018-0247-7

Beglinger, L., & Smith, T. (2005). Concurrent validity of social subtype and IQ after early intensive behavioral intervention in children with autism: A preliminary investigation. *Journal of Autism and Developmental Disorders*, *35*(3), 295–303. https://doi.org/10.1007/s10803-005-3292-3

Belisle, J., Dixon, M. R., Alholai, A., Ellenberger, L., Stanley, C., & Galliford, M. (2020). Teaching children with autism to tact the private events of others. *Behavior Analysis in Practice*, *13*(1), 169–173. https://doi.org/10.1007/s40617-019-00334-9

Belisle, J., Dixon, M. R., Stanley, C. R., Munoz, B., & Daar, J. H. (2016). Teaching foundational perspective-taking skills to children with autism using the PEAK-T curriculum: single-reversal “I–You” deictic frames. *Journal of Applied Behavior Analysis*, *49*(4), 965–969. https://doi.org/10.1002/jaba.324

Belisle, J., Huggins, K., Doherty, M., Stanley, C. R., & Dixon, M. R. (2020). Generalized reflexive responding and cross-modal tactile transfer of stimulus function in children with autism. *Analysis of Verbal Behavior*, *36*(2), 233–250. https://doi.org/10.1007/s40616-020-00137-y

Belisle, J., Stanley, C. R., Alholail, A. M., Galliford, M. E., & Dixon, M. R. (2019). Abstraction of tactile properties by individuals with autism and down syndrome using a picture‐based communication system. *Journal of Applied Behavior Analysis*, *52*(2), 467–475. https://doi.org/10.1002/jaba.526

Belisle, J., Stanley, C. R., Schmick, A., Dixon, M. R., Alholail, A., Galliford, M. E., & Ellenberger, L. (2020). Establishing arbitrary comparative relations and referential transformations of stimulus function in individuals with autism. *Journal of Applied Behavior Analysis*, *53*(2), 938–955. https://doi.org/https://doi.org/10.1002/jaba.655

Ben-Itzchak, E., & Zachor, D. A. (2007). The effects of intellectual functioning and autism severity on outcome of early behavioral intervention for children with autism. *Research in Developmental Disabilities*, *28*(3), 287–303. https://doi.org/10.1016/j.ridd.2006.03.002

Bergstrom, R., Najdowski, A. C., Alvarado, M., & Tarbox, J. (2016). Teaching children with autism to tell socially appropriate lies. *Journal of Applied Behavior Analysis*, *49*(2), 405–410. https://doi.org/10.1002/jaba.295

Bergstrom, R., Najdowski, A. C., & Tarbox, J. (2012). Teaching children with autism to seek help when lost in public. *Journal of Applied Behavior Analysis*, *45*(1), 191–195. https://doi.org/10.1901/jaba.2012.45-191

Bergstrom, R., Najdowski, A. C., & Tarbox, J. (2014). A systematic replication of teaching children with autism to respond appropriately to lures from strangers. *Journal of Applied Behavior Analysis*, *47*(4), 861–865. https://doi.org/10.1002/jaba.175

Bernard-Opitz, V., Ing, S., & Kong, T. Y. (2004). Comparison of Behavioural and Natural Play Interventions for Young Children with Autism. *Autism*, *8*(3), 319–333. https://doi.org/10.1177/1362361304045212

Betz, A. M., Higbee, T. S., Kelley, K. N., Sellers, T. P., & Pollard, J. S. (2011). Increasing response variability of mand frames with script training and extinction. *Journal of Applied Behavior Analysis*, *44*(2), 357–362. https://doi.org/10.1901/jaba.2011.44-357

Betz, A., Higbee, T. S., & Reagon, K. A. (2008). Using joint activity schedules to promote peer engagement in preschoolers with autism. *Journal of Applied Behavior Analysis*, *41*(2), 237–241. https://doi.org/10.1901/jaba.2008.41-237

Bibby, P., Eikeseth, S., Martin, N. T., Mudford, O. C., & Reeves, D. (2002). Progress and outcomes for children with autism receiving parent-managed intensive interventions. *Research in Developmental Disabilities*, *23*(1), 81–104. https://doi.org/10.1016/S0891-4222(02)00095-1

Bicard, D. F., Horan, J., Plank, E., & Covington, T. (2010). May I Take a Message? Using General Case Programming to Teach Students with Disabilities to Take and Give Phone Messages. *Preventing School Failure*, *54*(3), 179–189. https://doi.org/10.1080/10459880903217630

Birnbrauer, J. S., & Leach, D. J. (1993). The Murdoch Early Intervention Program After 2 Years. *Behaviour Change*, *10*(2), 63–74. https://doi.org/10.1017/S0813483900005556

Bishop, S. K., Moore, J. W., Dart, E. H., Radley, K., Brewer, R., Barker, L., Quintero, L., Litten, S., Gilfeather, A., Newborne, B., & Toche, C. (2020). Further investigation of increasing vocalizations of children with autism with a speech‐generating device. *Journal of Applied Behavior Analysis*, *53*(1), 475–483. https://doi.org/10.1002/jaba.554

Bloom, S. E., Lambert, J. M., Dayton, E., & Samaha, A. L. (2013). Teacher-conducted trial-based functional analyses as the basis for intervention. *Journal of Applied Behavior Analysis*, *46*(1), 208–218. https://doi.org/10.1002/jaba.21

Boudreau, B. A., Vladescu, J. C., Kodak, T. M., Argott, P. J., & Kisamore, A. N. (2015). A comparison of differential reinforcement procedures with children with autism. *Journal of Applied Behavior Analysis*, *48*(4), 918–923. https://doi.org/10.1002/jaba.232

Bourret, J., Vollmer, T. R., & Rapp, J. T. (2004). Evaluation of a vocal mand assessment and vocal mand training procedures. *Journal of Applied Behavior Analysis*, *37*(2), 129–144. https://doi.org/10.1901/jaba.2004.37-129

Boutain, A. R., Sheldon, J. B., & Sherman, J. A. (2020). Evaluation of a telehealth parent training program in teaching self‐care skills to children with autism. *Journal of Applied Behavior Analysis*, *53*(3), 1259–1275. https://doi.org/10.1002/jaba.743

Bowen, C. N., Shillingsburg, M. A., & Carr, J. E. (2012). The effects of the question “What do you want?” on mand training outcomes of children with autism. *Journal of Applied Behavior Analysis*, *45*(4), 833–838. https://doi.org/10.1901/jaba.2012.45-833

Breeman, S. L., Vladescu, J. C., DeBar, R. M., Grow, L. L., & Marano, K. E. (2020). The effects of procedural integrity errors during auditory–visual conditional discrimination training: A preliminary investigation. *Behavioral Interventions*, *35*(2), 203–216. https://doi.org/10.1002/bin.1710

Brodhead, M. T., Courtney, W. T., & Thaxton, J. R. (2018). Using activity schedules to promote varied application use in children with autism. *Journal of Applied Behavior Analysis*, *51*(1), 80–86. https://doi.org/10.1002/jaba.435

Brodhead, M. T., Higbee, T. S., Gerencser, K. R., & Akers, J. S. (2016). The use of a discrimination-training procedure to teach mand variability to children with autism. *Journal of Applied Behavior Analysis*, *49*(1), 34–48. https://doi.org/10.1002/jaba.280

Buckley, J., Luiselli, J. K., Harper, J. M., & Shlesinger, A. (2020). Teaching students with autism spectrum disorder to tolerate haircutting. *Journal of Applied Behavior Analysis*, *53*(4), 2081–2089. https://doi.org/10.1002/jaba.713

Buckley, S. D., Strunck, P. G., & Newchok, D. K. (2005). A comparison of two multicomponent procedures to increase food consumption. *Behavioral Interventions*, *20*(2), 139–146. https://doi.org/10.1002/bin.188

Buckley, S. D., & Newchok, D. K. (2005). An Evaluation of Simultaneous Presentation and Differential Reinforcement with Response Cost to Reduce Packing. *Journal of Applied Behavior Analysis*, *38*(3), 405–409. https://doi.org/10.1901/jaba.2005.71-04

Buckley, S. D., & Newchok, D. K. (2006). Analysis and Treatment of Problem Behavior Evoked by Music. *Journal of Applied Behavior Analysis*, *39*(1), 141–144. https://doi.org/10.1901/jaba.2006.120-04

Budzińska, A., & Lubomirska, A. (2009). The use of applied behaviour analysis techniques in reducing self-injurious behaviours in a three-year-old autistic girl. *Acta Neuropsychologica*, *7*(3), 196–209.

Budzińska, A., Lubomirska, A., Wójcik, M., Krantz, P. J., & McClannahan, L. (2014). Use of scripts and script-fading procedures and activity schedules to develop spontaneous social interaction in a three-year-old girl with autism. *Health Psychology Report*, *2*(1), 67–71. https://doi.org/10.5114/hpr.2014.42791

Byrne, B. L., Rehfeldt, R. A., & Aguirre, A. A. (2014). Evaluating the effectiveness of the stimulus pairing observation procedure and multiple exemplar instruction on tact and listener responses in children with autism. *Analysis of Verbal Behavior*, *30*(2), 160–169. https://doi.org/10.1007/s40616-014-0020-0

Calise, J., Cautilli, J., & Galino, R. (2009). Can contingently imitating vocal response increase the frequency of vocal responses? *The Journal of Speech and Language Pathology – Applied Behavior Analysis*, *3*(2–3), 233–241. https://doi.org/10.1037/h0100240

Campanaro, A. M., Vladescu, J. C., Kodak, T., DeBar, R. M., & Nippes, K. C. (2020). Comparing skill acquisition under varying onsets of differential reinforcement: A  preliminary analysis. *Journal of Applied Behavior Analysis*, *53*(2), 690–706. https://doi.org/10.1002/jaba.615

Carbone, V. J., Lewis, L., Sweeney-Kerwin, E. J., Dixon, J., Louden, R., & Quinn, S. (2006). A comparison of two approaches for teaching VB functions: Total communication vs. vocal-alone. *The Journal of Speech and Language Pathology – Applied Behavior Analysis*, *1*(3), 181–192. https://doi.org/10.1037/h0100199

Carbone, V. J., Sweeney-Kerwin, E. J., Attanasio, V., & Kasper, T. (2010). Increasing the vocal responses of children with autism and developmental disabilities using manual sign mand training and prompt delay. *Journal of Applied Behavior Analysis*, *43*(4), 705–709. https://doi.org/10.1901/jaba.2010.43-705

Carlile, K. A., DeBar, R. M., Reeve, S. A., Reeve, K. F., & Meyer, L. S. (2018). Teaching help‐seeking when lost to individuals with autism spectrum disorder. *Journal of Applied Behavior Analysis*, *51*(2), 191–206. https://doi.org/10.1002/jaba.447

Carneiro, A. C. C., Flores, E. P., da Silva Barros, R., & de Souza, C. B. A. (2019). Evaluating the use of programmed reinforcement in a correction procedure with children diagnosed with autism. *Psicologia: Reflexão e Crítica*, *32*. https://doi.org/10.1186/s41155-019-0134-3

Carnett, A., Ingvarsson, E. T., Bravo, A., & Sigafoos, J. (2020). Teaching children with autism spectrum disorder to ask “where” questions using a speech‐generating device. *Journal of Applied Behavior Analysis*, *53*(3), 1383–1403. https://doi.org/10.1002/jaba.663

Carp, C. L., Peterson, S. P., Arkel, A. J., Petursdottir, A. I., & Ingvarsson, E. T. (2012). A further evaluation of picture prompts during auditory-visual conditional discrimination training. *Journal of Applied Behavior Analysis*, *45*(4), 737–751. https://doi.org/10.1901/jaba.2012.45-737

Carroll, R. A., Joachim, B. T., St. Peter, C. C., & Robinson, N. (2015). A comparison of error‐correction procedures on skill acquisition during discrete‐trial instruction. *Journal of Applied Behavior Analysis*, *48*(2), 257–273. https://doi.org/10.1002/jaba.205

Carroll, R. A., & Kodak, T. (2015). Using instructive feedback to increase response variability during intraverbal training for children with autism spectrum disorder. *Analysis of Verbal Behavior*, *31*(2), 183–199. https://doi.org/10.1007/s40616-015-0039-x

Carroll, R. A., Kodak, T., & Adolf, K. J. (2016). Effect of delayed reinforcement on skill acquisition during discrete‐trial instruction: Implications for treatment‐integrity errors in academic settings. *Journal of Applied Behavior Analysis*, *49*(1), 176–181. https://doi.org/10.1002/jaba.268

Carroll, R. A., Kodak, T., & Fisher, W. W. (2013). An evaluation of programmed treatment-integrity errors during discrete-trial instruction. *Journal of Applied Behavior Analysis*, *46*(2), 379–394. https://doi.org/10.1002/jaba.49

Carter, C. M. (2001). Using Choice with Game Play to Increase Language Skills and Interactive Behaviors in Children with Autism. *Journal of Positive Behavior Interventions*, *3*(3), 131–151. https://doi.org/10.1177/109830070100300302

Casey, S. D., Perrin, C. J., Merical, C. L., Lecomte, J. M., Milligan, J., & Walsh-Czekalski, M. (2008). Increasing bite acceptance and reducing food refusal in a child with autism: Moving beyond the clinic. *Journal of Behavior Analysis in Health, Sports, Fitness and Medicine*, *1*(1), 34–44. https://doi.org/10.1037/h0100366

Cassella, M. D., Sidener, T. M., Sidener, D. W., & Progar, P. R. (2011). Response interruption and redirection for vocal stereotypy in children with autism: A systematic replication. *Journal of Applied Behavior Analysis*, *44*(1), 169–173. https://doi.org/10.1901/jaba.2011.44-169

Cengher, M., & Fienup, D. M. (2020). Presession attention affects the acquisition of tacts and intraverbals. *Journal of Applied Behavior Analysis*, *53*(3), 1742–1767. https://doi.org/10.1002/jaba.657

Chan, J. M., & O’Reilly, M. F. (2008). A Social Stories^TM^ intervention package for students with autism in inclusive classroom settings. *Journal of Applied Behavior Analysis*, *41*(3), 405–409. https://doi.org/10.1901/jaba.2008.41-405

Charania, S. M., LeBlanc, L. A., Sabanathan, N., Ktaech, I. A., Carr, J. E., & Gunby, K. (2010). Teaching effective hand raising to children with autism during group instruction. *Journal of Applied Behavior Analysis*, *43*(3), 493–497. https://doi.org/10.1901/jaba.2010.43-493

Charlop, M. H., & Milstein, J. P. (1989). Teaching autistic children conversational speech using video modeling. *Journal of Applied Behavior Analysis*, *22*(3), 275–285. https://doi.org/10.1901/jaba.1989.22-275

Charlop, M. H., & Trasowech, J. E. (1991). Increasing autistic children’s daily spontaneous speech. *Journal of Applied Behavior Analysis*, *24*(4), 747–761. https://doi.org/10.1901/jaba.1991.24-747

Charlop, M. H., Schreibman, L., & Thibodeau, M. G. (1985). Increasing spontaneous verbal responding in autistic children using a time delay procedure. *Journal of Applied Behavior Analysis*, *18*(2), 155–166. https://doi.org/10.1901/jaba.1985.18-155

Charlop, M. H., & Walsh, M. E. (1986). Increasing autistic children’s spontaneous verbalizations of affection: an assessment of time delay and peer modeling procedures. *Journal of Applied Behavior Analysis*, *19*(3), 307–314. https://doi.org/10.1901/jaba.1986.19-307

Charlop-Christy, M. H., & Daneshvar, S. (2003). Using Video Modeling to Teach Perspective Taking to Children with Autism. *Journal of Positive Behavior Interventions*, *5*(1), 12–21. https://doi.org/10.1177/10983007030050010101

Charlop-Christy, M. H., & Haymes, L. K. (1996). Using obsessions as reinforcers with and without mild reductive procedures to decrease inappropriate behaviors of children with autism. *Journal of Autism and Developmental Disorders*, *26*(5), 527–546. https://doi.org/10.1007/BF02172274

Charlop-Christy, M. H., & Haymes, L. K. (1998). Using objects of obsession as token reinforcers for children with autism. *Journal of Autism and Developmental Disorders*, *28*(3), 189–198. https://doi.org/10.1023/A:1026061220171

Charlop-Christy, M. H., Carpenter, M., Le, L., LeBlanc, L. A., & Kellet, K. (2002). Using the picture exchange communication system (PECS) with children with autism: Assessment of PECS acquisition, speech, social-communicative behavior, and problem behavior. *Journal of Applied Behavior Analysis*, *35*(3), 213–231. https://doi.org/10.1901/jaba.2002.35-213

Charlop-Christy, M. H., & Kelso, S. E. (2003). Teaching Children With Autism Conversational Speech Using a Cue Card / Written Script Program. *Children*, *26*(2), 108–127.

Cihak, D. F. (2011). Comparing pictorial and video modeling activity schedules during transitions for students with autism spectrum disorders. *Research in Autism Spectrum Disorders*, *5*(1), 433–441. https://doi.org/10.1016/j.rasd.2010.06.006

Cihak, D. F., & Foust, J. L. (2008). Comparing Number Lines and Touch Points to Teach Addition Facts to Students With Autism. *Focus on Autism and Other Developmental Disabilities*, *23*(3), 131–137. https://doi.org/10.1177/1088357608318950

Cihon, J. H., Ferguson, J. L., Leaf, J. B., Leaf, R., McEachin, J., & Taubman, M. (2019). Use of a level system with flexible shaping to improve synchronous engagement. *Behavior Analysis in Practice*, *12*(1), 44–51. https://doi.org/10.1007/s40617-018-0254-8

Cihon, J. H., Ferguson, J. L., Leaf, J. B., Milne, C. M., Leaf, R., & McEachin, J. (2020). A randomized clinical trial of three prompting systems to teach tact relations. *Journal of Applied Behavior Analysis*, *53*(2), 727–743. https://doi.org/10.1002/jaba.617

Cihon, J. H., Milne, C. M., Ferguson, J. L., Leaf, J. B., & Leaf, R. (2020). Fad treatments in autism intervention: An evaluation of fidget spinners. *Education and Training in Autism and Developmental Disabilities*, *55*(4), 466–475.

Cividini‐Motta, C., Garcia, A. R., Livingston, C., & MacNaul, H. L. (2019). The effect of response interruption and redirection with and without a differential reinforcement of alternative behavior component on stereotypy and appropriate responses. *Behavioral Interventions*, *34*(1), 3–18. https://doi.org/10.1002/bin.1654

Clark, K. M., & Green, G. (2004). Comparison of two procedures for teaching dictated-word/symbol relations to learners with autism. *Journal of Applied Behavior Analysis*, *37*(4), 503–507. https://doi.org/10.1901/jaba.2004.37-503

Clausen, K. A., Alden-Anderson, E., Stephenson, K., Mueller, A., & Klatt, K. P. (2007). The effects of enthusiasm on skill acquisition by children with autism. *The Journal of Speech and Language Pathology – Applied Behavior Analysis*, *2*(1), 32–45. https://doi.org/10.1037/h0100205

Clay, C. J., Clohisy, A. M., Ball, A. M., Haider, A. F., Schmitz, B. A., & Kahng, S. (2018). Further evaluation of presentation format of competing stimuli for treatment of automatically maintained challenging behavior. *Behavior Modification*, *42*(3), 382–397. https://doi.org/10.1177/0145445517740322

Cohen, H., Amerine-Dickens, M., & Smith, T. (2006). Early Intensive Behavioral Treatment: Replication of the UCLA Model in a Community Setting. *Journal of Developmental & Behavioral Pediatrics*, *27*(2), S145–S155. https://doi.org/10.1097/00004703-200604002-00013

Cohenour, J. M., Volkert, V. M., & Allen, K. D. (2018). An experimental demonstration of AAB renewal in children with autism spectrum disorder. *Journal of the Experimental Analysis of Behavior*, *110*(1), 63–73. https://doi.org/10.1002/jeab.443

Colón, C. L., & Ahearn, W. H. (2019). An analysis of treatment integrity of response interruption and redirection. *Journal of Applied Behavior Analysis*, *52*(2), 337–354. https://doi.org/10.1002/jaba.537

Colón, C. L., Ahearn, W. H., Clark, K. M., & Masalsky, J. (2012). The Effects of Verbal Operant Training and Response Interruption and Redirection on Appropriate and Inappropriate Vocalizations. *Journal of Applied Behavior Analysis*, *45*(1), 107–120. https://doi.org/10.1901/jaba.2012.45-107

Conallen, K., & Reed, P. (2017). Children with autism spectrum disorder: Teaching conversation involving feelings about events. *Journal of Intellectual Disability Research*, *61*(3), 279–291. https://doi.org/10.1111/jir.12339

Conine, D. E., Vollmer, T. R., Barlow, M. A., Grauerholz‐Fisher, E., Dela Rosa, C. M., & Petronelli, A. K. (2020). Assessment and treatment of response to name for children with autism spectrum disorder: Toward an efficient intervention model. *Journal of Applied Behavior Analysis*, *53*(4), 2024–2052. https://doi.org/10.1002/jaba.737

Conine, D. E., Vollmer, T. R., & Bolívar, H. A. (2020). Response to name in children with autism: Treatment, generalization, and maintenance. *Journal of Applied Behavior Analysis*, *53*(2), 744–766. https://doi.org/10.1002/jaba.635

Connolly, S. C., Millians, M., Peterman, R., & Shillingsburg, M. A. (2016). The clinical application of applied behavior analysis in a child with partial fetal alcohol syndrome: A case study. *Clinical Case Studies*, *15*(3), 225–242. https://doi.org/10.1177/1534650116632298

Contreras, B. P., & Betz, A. M. (2016). Using lag schedules to strengthen the intraverbal repertoires of children with autism. *Journal of Applied Behavior Analysis*, *49*(1), 3–16. https://doi.org/10.1002/jaba.271

Cook, J. L., Rapp, J. T., & Schulze, K. A. (2015). Differential negative reinforcement of other behavior to increase wearing of a medical bracelet. *Journal of Applied Behavior Analysis*, *48*(4), 901–906. https://doi.org/10.1002/jaba.228

Coolican, J., Smith, I. M., & Bryson, S. E. (2010). Brief parent training in pivotal response treatment for preschoolers with autism. *Journal of Child Psychology and Psychiatry and Allied Disciplines*, *51*(12), 1321–1330. https://doi.org/10.1111/j.1469-7610.2010.02326.x

Cornelius Habarad, S. M. (2015). The power of the mand: Utilizing the mand repertoire to decrease problem behavior. *Behavioral Development Bulletin*, *20*(2), 158–162. https://doi.org/10.1037/h0101310

Cox, A. D., Virues‐Ortega, J., Julio, F., & Martin, T. L. (2017). Establishing motion control in children with autism and intellectual disability: Applications for anatomical and functional MRI. *Journal of Applied Behavior Analysis*, *50*(1), 8–26. https://doi.org/10.1002/jaba.351

Coyle, C., & Cole, P. (2004). A videotaped self-modelling and self-monitoring treatment program to decrease off-task behaviour in children with autism. *Journal of Intellectual and Developmental Disability*, *29*(1), 3–16. https://doi.org/10.1080/08927020410001662642

Crowley, J. G., Peterson, K. M., Fisher, W. W., & Piazza, C. C. (2020). Treating food selectivity as resistance to change in children with autism spectrum disorder. *Journal of Applied Behavior Analysis*, *53*(4), 2002–2023. https://doi.org/10.1002/jaba.711

Cubicciotti, J. E., Vladescu, J. C., Reeve, K. F., Carroll, R. A., & Schnell, L. K. (2019). Effects of stimulus presentation order during auditory–visual conditional discrimination training for children with autism spectrum disorder. *Journal of Applied Behavior Analysis*, *52*(2), 541–556. https://doi.org/10.1002/jaba.530

Danov, S. E., Hartman, E., McComas, J. J., & Symons, F. J. (2010). Evaluation of two communicative response modalities for a child with autism and self-injury. *The Journal of Speech and Language Pathology – Applied Behavior Analysis*, *5*(1), 70–79. https://doi.org/10.1037/h0100263

Daou, N. (2014). Conducting behavioral research with children attending nonbehavioral intervention programs for autism: The case of Lebanon. *Behavior Analysis in Practice*, *7*(2), 78–90. https://doi.org/10.1007/s40617-014-0017-0

Dass, T. K., Kisamore, A. N., Vladescu, J. C., Reeve, K. F., Reeve, S. A., & Taylor‐Santa, C. (2018). Teaching children with autism spectrum disorder to tact olfactory stimuli. *Journal of Applied Behavior Analysis*, *51*(3), 538–552. https://doi.org/10.1002/jaba.470

Davis, B. J., Kahng, S., & Coryat, K. (2012). Manipulating motivating operations to facilitate the emergence of mands for a child with autism. *Analysis of Verbal Behavior*, *28*, 145–150. https://doi.org/10.1007/BF03393116

Dawson, G., Rogers, S., Munson, J., Smith, M., Winter, J., Greenson, J., Donaldson, A., & Varley, J. (2010). Randomized, Controlled Trial of an Intervention for Toddlers With Autism: The Early Start Denver Model. *PEDIATRICS*, *125*(1), e17–e23. https://doi.org/10.1542/peds.2009-0958

Day-Watkins, J., Murray, R., & Connell, J. E. (2014). Teaching helping to adolescents with autism. *Journal of Applied Behavior Analysis*, *47*(4), 850–855. https://doi.org/10.1002/jaba.156

Delfs, C. H., Conine, D. E., Frampton, S. E., Shillingsburg, M. A., & Robinson, H. C. (2014). Evaluation of the efficiency of listener and tact instruction for children with autism. *Journal of Applied Behavior Analysis*, *47*(4), 793–809. https://doi.org/10.1002/jaba.166

Demiri, V. (2004). Teaching social skills to children with autism using social stories: An empirical study. [ProQuest Information & Learning]. In *Dissertation Abstracts International: Section B: The Sciences and Engineering* (Vol. 65, Issues 5-B).

DeQuinzio, J. A., Taylor, B. A., & Tomasi, B. J. (2018). Observational learning and children with autism: Discrimination training of known and unknown stimuli. *Journal of Applied Behavior Analysis*, *51*(4), 802–818. https://doi.org/10.1002/jaba.481

DeQuinzio, J. A., & Taylor, B. A. (2015). Teaching children with autism to discriminate the reinforced and nonreinforced responses of others: Implications for observational learning. *Journal of Applied Behavior Analysis*, *48*(1), 38–51. https://doi.org/10.1002/jaba.192

DeQuinzio, J. A., Townsend, D. B., Sturmey, P., & Poulson, C. L. (2007). Generalized imitation of facial models by children with autism. *Journal of Applied Behavior Analysis*, *40*(4), 755–759. https://doi.org/10.1901/jaba.2007.755-759

DeRosa, N. M., Novak, M. D., Morley, A. J., & Roane, H. S. (2019). Comparing response blocking and response interruption/redirection on levels of motor stereotypy: Effects of data analysis procedures. *Journal of Applied Behavior Analysis*, *52*(4), 1021–1033. https://doi.org/https://doi.org/10.1002/jaba.644

Deshais, M. A., & Vollmer, T. R. (2020). A preliminary investigation of fixed and repetitive models during object imitation training. *Journal of Applied Behavior Analysis*, *53*(2), 973–996. https://doi.org/https://doi.org/10.1002/jaba.661

DeSouza, A. A., Fisher, W. W., & Rodriguez, N. M. (2019). Facilitating the emergence of convergent intraverbals in children with autism. *Journal of Applied Behavior Analysis*, *52*(1), 28–49. https://doi.org/10.1002/jaba.520

Devlin, S., Healy, O., Leader, G., & Hughes, B. M. (2011). Comparison of behavioral intervention and sensory-integration therapy in the treatment of challenging behavior. *Journal of Autism and Developmental Disorders*, *41*, 1303–1320. https://doi.org/10.1007/s10803-010-1149-x

Dickes, N. R., & Kodak, T. (2015). Evaluating the emergence of reverse intraverbals following intraverbal training in young children with autism spectrum disorder. *Behavioral Interventions*, *30*(3), 169–190. https://doi.org/10.1002/bin.1412

DiGennaro, F. D., Martens, B. K., & Kleinmann, A. E. (2007). a Comparison of Performance Feedback Procedures on Teachers’ Treatment Implementation Integrity and Students’ Inappropriate Behavior in Special Education Classrooms. *Journal of Applied Behavior Analysis*, *40*(3), 447–461. https://doi.org/10.1901/jaba.2007.40-447

Dittlinger, L. H., & Lerman, D. C. (2011). Further analysis of picture interference when teaching word recognition to children with autism. *Journal of Applied Behavior Analysis*, *44*(2), 341–349. https://doi.org/10.1901/jaba.2011.44-341

Dixon, M. R., Belisle, J., Stanley, C. R., & Rowsey, K. (2018). Student outcomes after 1 year of front line staff implementation of the PEAK curriculum. *Behavioral Interventions*, *33*(2), 185–195. https://doi.org/10.1002/bin.1516

Dixon, M. R., & Cummings, A. (2001). Self-control in children with autism: Response allocation during delays to reinforcement. *Journal of Applied Behavior Analysis*, *34*(4), 491–495. https://doi.org/10.1901/jaba.2001.34-491

Dixon, M. R., McCord, B. E., & Belisle, J. (2018). A demonstration of higher‐order response class development in children. *Journal of Applied Behavior Analysis*, *51*(3), 590–595. https://doi.org/10.1002/jaba.456

Dixon, M. R., Peach, J., Daar, J. H., & Penrod, C. (2017). Teaching complex verbal operants to children with autism and establishing generalization using the peak curriculum. *Journal of Applied Behavior Analysis*, *50*(2), 317–331. https://doi.org/10.1002/jaba.373

Doenyas, C., Şimdi, E., Özcan, E. Ç., Çataltepe, Z., & Birkan, B. (2014). Autism and tablet computers in Turkey: Teaching picture sequencing skills via a web-based iPad application. *International Journal of Child-Computer Interaction*, *2*(1), 60–71. https://doi.org/10.1016/j.ijcci.2014.04.002

Dolezal, D. N., & Kurtz, P. F. (2010). Evaluation of combined-antecedent variables on functional analysis results and treatment of problem behavior in a school setting. *Journal of Applied Behavior Analysis*, *43*(2), 309–314. https://doi.org/10.1901/jaba.2010.43-309

Donaldson, J. M., & Vollmer, T. R. (2011). an Evaluation and Comparison of Time-Out Procedures With and Without Release Contingencies. *Journal of Applied Behavior Analysis*, *44*(4), 693–705. https://doi.org/10.1901/jaba.2011.44-693

Dowdy, A., & Tincani, M. (2020). Assessment and treatment of high‐risk challenging behavior of adolescents with autism in an aquatic setting. *Journal of Applied Behavior Analysis*, *53*(1), 305–314. https://doi.org/10.1002/jaba.590

Dowdy, A., Tincani, M., Nipe, T., & Weiss, M. J. (2018). Effects of reinforcement without extinction on increasing compliance with nail cutting: A systematic replication. *Journal of Applied Behavior Analysis*, *51*(4), 924–930. https://doi.org/10.1002/jaba.484

Dozier, C. L., Carr, J. E., Enloe, K., Landaburu, H., Eastridge, D., & Kellum, K. K. (2001). Using fixed-time schedules to maintain behavior: A preliminary investigation. *Journal of Applied Behavior Analysis*, *34*(3), 337–340. https://doi.org/10.1901/jaba.2001.34-337

Drasgow, E., Halle, J. W., & Ostrosky, M. M. (1998). Effects of Differential Reinforcement on the Generalization of a Replacement Mand in Three Children With Severe Language Delays. *Journal of Applied Behavior Analysis*, *31*(3), 357–374. https://doi.org/10.1901/jaba.1998.31-357

Drasgow, E., Halle, J. W., & Phillips, B. (2001). Effects of different social partners on the discriminated requesting of a young child with autism and severe language delays. *Research in Developmental Disabilities*, *22*(2), 125–139. https://doi.org/10.1016/S0891-4222(01)00062-2

Dufour, M., & Lanovaz, M. J. (2020). Increasing compliance with wearing a medical device in children with autism. *Journal of Applied Behavior Analysis*, *53*(2), 1089–1096. https://doi.org/10.1002/jaba.628

Dugan, K. T. (2006). Facilitating independent behaviors in children with autism employing picture activity schedules. In *ProQuest Dissertations and Theses*.

Dupere, S., MacDonald, R. P. F., & Ahearn, W. H. (2013). Using video modeling with substitutable loops to teach varied play to children with autism. *Journal of Applied Behavior Analysis*, *46*(3), 662–668. https://doi.org/10.1002/jaba.68

Dupuis, D. L., Lerman, D. C., Tsami, L., & Shireman, M. L. (2015). Reduction of aggression evoked by sounds using noncontingent reinforcement and time‐out. *Journal of Applied Behavior Analysis*, *48*(3), 669–674. https://doi.org/10.1002/jaba.220

Durand, V. M. (1999). Functional communication training using assistive devices: Recruiting natural communities of reinforcement. *Journal of Applied Behavior Analysis*, *32*(3), 247–267. https://doi.org/10.1901/jaba.1999.32-247

Dwiggins, G. A. (2009). Promoting efficiency: A comparison of two teaching protocols in the education of children with autism. [ProQuest Information & Learning]. In *Dissertation Abstracts International Section A: Humanities and Social Sciences* (Vol. 69, Issues 9-A).

Easterbrooks, S. R., & Handley, C. M. (2006). Behavior change in a student with a dual diagnosis of deafness and pervasive developmental disorder: a case study. *American Annals Of The Deaf*, *150*(5), 401–407. https://doi.org/10.1353/aad.2006.0001

Eby, C. M., Greer, R. D., Tullo, L. D., Baker, K. A., & Pauly, R. (2010). Effects of multiple exemplar instruction on transformation of stimulus function across written and vocal spelling responses by students with autism. *The Journal of Speech and Language Pathology – Applied Behavior Analysis*, *5*(1), 20–31. https://doi.org/10.1037/h0100262

Egan, C. E., & Barnes-Holmes, D. (2009). Emergence of tacts following mand training in young children with autism. *Journal of Applied Behavior Analysis*, *42*(3), 691–696. https://doi.org/10.1901/jaba.2009.42-691

Eikeseth, S., & Hayward, D. W. (2009). The discrimination of object names and object sounds in children with autism: A procedure for teaching verbal comprehension. *Journal of Applied Behavior Analysis*, *42*(4), 807–812. https://doi.org/10.1901/jaba.2009.42-807

Eikeseth, S., Hayward, D., Gale, C., Gitlesen, J. P., & Eldevik, S. (2009). Intensity of supervision and outcome for preschool aged children receiving early and intensive behavioral interventions: A preliminary study. *Research in Autism Spectrum Disorders*, *3*(1), 67–73. https://doi.org/10.1016/j.rasd.2008.04.003

Eikeseth, S., Klintwall, L., Jahr, E., & Karlsson, P. (2012). Outcome for children with autism receiving early and intensive behavioral intervention in mainstream preschool and kindergarten settings. *Research in Autism Spectrum Disorders*, *6*(2), 829–835. https://doi.org/10.1016/j.rasd.2011.09.002

Eikeseth, S., Smith, T., Jahr, E., & Eldevik, S. (2002). Intensive behavioral treatment at school for 4- to 7-year-old children with autism: A 1-year comparison controlled study. *Behavior Modification*, *26*(1), 49–68. https://doi.org/10.1177/0145445502026001004

Eikeseth, S., Smith, T., Jahr, E., & Eldevik, S. (2007). Outcome for Children with Autism who Began Intensive Behavioral Treatment Between Ages 4 and 7: A Comparison Controlled Study. *Behavior Modification*, *31*(3), 264–278. https://doi.org/10.1177/0145445506291396

Eldevik, S., Eikeseth, S., Jahr, E., & Smith, T. (2006). Effects of low-intensity behavioral treatment for children with autism and mental retardation. *Journal of Autism and Developmental Disorders*, *36*(2), 211–224. https://doi.org/10.1007/s10803-005-0058-x

Eldevik, S., Hastings, R. P., Jahr, E., & Hughes, J. C. (2012). Outcomes of behavioral intervention for children with autism in mainstream pre-school settings. *Journal of Autism and Developmental Disorders*, *42*(2), 210–220. https://doi.org/10.1007/s10803-011-1234-9

Ellingson, S. A., Miltenberger, R. G., Stricker, J., Galensky, T. L., & Garlinghouse, M. (2000). Functional assessment and intervention for challenging behaviors in the classroom by general classroom teachers. *Journal of Positive Behavior Interventions*, *2*(2), 85–97. https://doi.org/10.1177/109830070000200202

Elliott, C., & Dillenburger, K. (2016). The effect of choice on motivation for young children on the autism spectrum during discrete trial teaching. *Journal of Research in Special Educational Needs*, *16*(3), 187–198. https://doi.org/10.1111/1471-3802.12073

Ervin, R. A., DuPaul, G. J., Kern, L., & Friman, P. C. (1998). Classroom-Based Functional and Adjunctive Assessments: Proactive Approaches to Intervention Selection for Adolescents with Attention Deficit Hyperactivity Disorder. *Journal of Applied Behavior Analysis*, *31*(1), 65–78. https://doi.org/10.1901/jaba.1998.31-65

Esch, B. E., Carr, J. E., & Grow, L. L. (2009). Evaluation of an enhanced stimulus-stimulus pairing procedure to increase early vocalizations of children with autism. *Journal of Applied Behavior Analysis*, *42*(2), 225–241. https://doi.org/10.1901/jaba.2009.42-225

Evans, C. P. (2013). The efficacy of a social skills group in the treatment of children and adolescents with an autism spectrum disorder. [ProQuest Information & Learning]. In *Dissertation Abstracts International: Section B: The Sciences and Engineering* (Vol. 74, Issues 4-B(E)).

Ezzeddine, E. W., DeBar, R. M., Reeve, S. A., & Townsend, D. B. (2020). Using video modeling to teach play comments to dyads with ASD. *Journal of Applied Behavior Analysis*, *53*(2), 767–781. https://doi.org/10.1002/jaba.621

Fabrizio, M. A., Schirmer, K., King, A., Diakite, A., & Stovel, L. (2007). Precision teaching a foundational motor skill to a child with autism. *Journal of Precision Teaching & Celeration*, *23*, 16–18.

Fairchild, L. A., Gadke, D. L., Stratton, K. K., Mathis, E. S., & Clarke, A. B. (2020). Evaluating the Influence of Intraverbal Topography in Conditional Discrimination Procedures. *Journal of Autism and Developmental Disorders*, *50*(2), 665–669. https://doi.org/10.1007/s10803-019-04275-8

Falcomata, T. S., Muething, C. S., Silbaugh, B. C., Adami, S., Hoffman, K., Shpall, C., & Ringdahl, J. E. (2018). Lag schedules and functional communication training: Persistence of mands and relapse of problem behavior. *Behavior Modification*, *42*(3), 314–334. https://doi.org/10.1177/0145445517741475

Falcomata, T. S., Roane, H. S., Feeney, B. J., & Stephenson, K. M. (2010). Assessment and treatment of elopement maintained by access to stereotypy. *Journal of Applied Behavior Analysis*, *43*(3), 513–517. https://doi.org/10.1901/jaba.2010.43-513

Farber, R. S., Dube, W. V, & Dickson, C. A. (2016). A sorting‐to‐matching method to teach compound matching to sample. *Journal of Applied Behavior Analysis*, *49*(2), 294–307. https://doi.org/10.1002/jaba.290

Farrell, P., Trigonaki, N., & Webster, D. (2005). An exploratory evaluation of two early intervention programmes for young children with autism. *Educational and Child Psychology*, *22*(4), 29–40.

Ferguson, J. L., Leaf, J. A., Cihon, J. H., Milne, C. M., Leaf, J. B., McEachin, J., & Leaf, R. (2020). Practical functional assessment: A case study replication and extension with a child diagnosed with autism spectrum disorder. *Education & Treatment of Children*, *43*(2), 171–185. https://doi.org/ /10.1007/s43494-020-00015-1

Ferguson, J. L., Majeski, M. J., McEachin, J., Leaf, R., Cihon, J. H., & Leaf, J. B. (2020). Evaluating discrete trial teaching with instructive feedback delivered in a dyad  arrangement via telehealth. *Journal of Applied Behavior Analysis*, *53*(4), 1876–1888. https://doi.org/10.1002/jaba.773

Fernell, E., Hedvall, A., Westerlund, J., Hoglund Carlsson, L., Eriksson, M., Barnevik Olsson, M., Holm, A., Norrelgen, F., Kjellmer, L., & Gillberg, C. (2011). Early intervention in 208 Swedish preschoolers with autism spectrum disorder. A prospective naturalistic study. *Research in Developmental Disabilities*, *32*(6), 2092–2101. https://doi.org/10.1016/j.ridd.2011.08.002

Ferris, K. J., & Fabrizio, M. A. (2009). Comparison of error correction procedures involving a speech-generating device to teach a child with autism new tacts. *The Journal of Speech and Language Pathology – Applied Behavior Analysis*, *3*(2–3), 185–198. https://doi.org/10.1037/h0100246

Fienup, D. M., & Doepke, K. (2008). Evaluation of a changing criterion intervention to increase fluent responding with an elementary age student with autism. *International Journal of Behavioral Consultation and Therapy*, *4*(3), 297–303. https://doi.org/10.1037/h0100859

Finn, H. E., Miguel, C. F., & Ahearn, W. H. (2012). The emergence of untrained mands and tacts in children with autism. *Journal of Applied Behavior Analysis*, *45*(2), 265–280. https://doi.org/10.1901/jaba.2012.45-265

Fischer, J. (2017). Using video modeling to teach first and second person pronouns to children with autism. [ProQuest Information & Learning]. In *Dissertation Abstracts International: Section B: The Sciences and Engineering* (Vol. 77, Issues 10-B(E)).

Fischer, J. L., Howard, J. S., Sparkman, C. R., & Moore, A. G. (2010). Establishing generalized syntactical responding in young children with autism. *Research in Autism Spectrum Disorders*, *4*(1), 76–88. https://doi.org/10.1016/j.rasd.2009.07.009

Fischetti, A. T., Wilder, D. A., Myers, K., Leon-Enriquez, Y., Sinn, S., & Rodriguez, R. (2012). An evaluation of evidence-based interventions to increase compliance among children with autism. *Journal of Applied Behavior Analysis*, *45*(4), 859–863. https://doi.org/10.1901/jaba.2012.45-859

Fisher, W. W., Felber, J. M., Phillips, L. A., Craig, A. R., Paden, A. R., & Niemeier, J. J. (2019). Treatment of resistance to change in children with autism. *Journal of Applied Behavior Analysis*, *52*(4), 974–993. https://doi.org/10.1002/jaba.588

Fisher, W. W., Greer, B. D., Fuhrman, A. M., Saini, V., & Simmons, C. A. (2018). Minimizing resurgence of destructive behavior using behavioral momentum theory. *Journal of Applied Behavior Analysis*, *51*(4), 831–853. https://doi.org/10.1002/jaba.499

Fisher, W. W., Kodak, T., & Moore, J. W. (2007). Embedding an identity-matching task within a prompting hierarchy to facilitate acquisition of conditional discriminations in children with autism. *Journal of Applied Behavior Analysis*, *40*(3), 489–499. https://doi.org/10.1901/jaba.2007.40-489

Fisher, W. W., O’Connor, J. T., Kurtz, P. F., DeLeon, I. G., & Gotjen, D. L. (2000). The effects of noncontingent delivery of high- and low-preference stimuli on attention-maintained destructive behavior. *Journal of Applied Behavior Analysis*, *33*(1), 79–83. https://doi.org/10.1901/jaba.2000.33-79

Fisher, W. W., Pawich, T. L., Dickes, N., Paden, A. R., & Toussaint, K. (2014). Increasing the saliency of behavior–consequence relations for children with autism who exhibit persistent errors. *Journal of Applied Behavior Analysis*, *47*(4), 738–748. https://doi.org/10.1002/jaba.172

Fisher, W. W., Retzlaff, B. J., Akers, J. S., DeSouza, A. A., Kaminski, A. J., & Machado, M. A. (2019). Establishing initial auditory‐visual conditional discriminations and emergence of initial tacts in young children with autism spectrum disorder. *Journal of Applied Behavior Analysis*, *52*(4), 1089–1106. https://doi.org/10.1002/jaba.586

Fiske, K. E., Isenhower, R. W., Bamond, M. J., Delmolino, L., Sloman, K. N., & LaRue, R. H. (2015). Assessing the value of token reinforcement for individuals with Autism. *Journal of Applied Behavior Analysis*, *48*(2), 448–453. https://doi.org/10.1002/jaba.207

Flanagan, H. E., Perry, A., & Freeman, N. L. (2012). Effectiveness of large-scale community-based Intensive Behavioral Intervention: A waitlist comparison study exploring outcomes and predictors. *Research in Autism Spectrum Disorders*, *6*(2), 673–682. https://doi.org/10.1016/j.rasd.2011.09.011

Fossett, B., & Mirenda, P. (2006). Sight word reading in children with developmental disabilities: A comparison of paired associate and picture-to-text matching instruction. *Research in Developmental Disabilities*, *27*(4), 411–429. https://doi.org/10.1016/j.ridd.2005.05.006

Fragale, C. L., O’Reilly, M. F., Aguilar, J., Pierce, N., Lang, R., Sigafoos, J., & Lancioni, G. (2012). The influence of motivating operations on generalization probes of specific mands by children with autism. *Journal of Applied Behavior Analysis*, *45*(3), 565–577. https://doi.org/10.1901/jaba.2012.45-565

Frampton, S. E., & Shillingsburg, M. A. (2020). Promoting the development of verbal responses using instructive feedback. *Journal of Applied Behavior Analysis*, *53*(2), 1029–1041. https://doi.org/https://doi.org/10.1002/jaba.659

Frampton, S. E., & Shillingsburg, M. A. (2018). Teaching children with autism to explain how: A case for problem solving? *Journal of Applied Behavior Analysis*, *51*(2), 236–254. https://doi.org/10.1002/jaba.445

Francisco, M. T., Borrero, J. C., & Sy, J. R. (2008). Evaluation of absolute and relative reinforcer value using progressive-ratio schedules. *Journal of Applied Behavior Analysis*, *41*(2), 189–202. https://doi.org/10.1901/jaba.2008.41-189

Francisco, M. T., & Hanley, G. P. (2012). An evaluation of progressively increasing intertrial intervals on the acquisition and generalization of three social skills. *Journal of Applied Behavior Analysis*, *45*(1), 137–142. https://doi.org/10.1901/jaba.2012.45-137

Fritz, J. N., Jackson, L. M., Stiefler, N. A., Wimberly, B. S., & Richardson, A. R. (2017). Noncontingent reinforcement without extinction plus differential reinforcement of alternative behavior during treatment of problem behavior. *Journal of Applied Behavior Analysis*, *50*(3), 590–599. https://doi.org/10.1002/jaba.395

Fu, S. B., Penrod, B., Fernand, J. K., Whelan, C. M., Griffith, K., & Medved, S. (2015). The effects of modeling contingencies in the treatment of food selectivity in children with autism. *Behavior Modification*, *39*(6), 771–784. https://doi.org/10.1177/0145445515592639

Fulton, C. J., Tiger, J. H., Meitzen, H. M., & Effertz, H. M. (2020). A comparison of accumulated and distributed reinforcement periods with children exhibiting escape‐maintained problem behavior. *Journal of Applied Behavior Analysis*, *53*(2), 782–795. https://doi.org/10.1002/jaba.622

Gale, C. M., Eikeseth, S., & Rudrud, E. (2011). Functional assessment and behavioural intervention for eating difficulties in children with autism: A study conducted in the natural environment using parents and ABA tutors as therapists. *Journal of Autism and Developmental Disorders*, *41*(10), 1383–1396. https://doi.org/10.1007/s10803-010-1167-8

Gallant, E. E., Reeve, S. A., Brothers, K. J., & Reeve, K. F. (2017). Auditory script location does not affect acquisition and maintenance of vocal initiations by children with autism. *Behavioral Interventions*, *32*(2), 103–120. https://doi.org/10.1002/bin.1467

Gamby, T. E. (2002). Remediating generalization deficits in children with autism: An empirical investigation. [ProQuest Information & Learning]. In *Dissertation Abstracts International: Section B: The Sciences and Engineering* (Vol. 62, Issues 12-B).

Ganz, J. B., & Flores, M. M. (2008). Effects of the use of visual strategies in play groups for children with autism spectrum disorders and their peers. *Journal of Autism and Developmental Disorders*, *38*(5), 926–940. https://doi.org/10.1007/s10803-007-0463-4

Garcia, D., Dukes, C., Brady, M. P., Scott, J., & Wilson, C. L. (2016). Using modeling and rehearsal to teach fire safety to children with autism. *Journal of Applied Behavior Analysis*, *49*(3), 699–704. https://doi.org/10.1002/jaba.331

Garcia‐Albea, E., Reeve, S. A., Brothers, K. J., & Reeve, K. F. (2014). Using audio script fading and multiple‐exemplar training to increase vocal interactions in children with autism. *Journal of Applied Behavior Analysis*, *47*(2), 325–343. https://doi.org/10.1002/jaba.125

Garfinkle, A. N., & Schwartz, I. S. (2002). Peer Imitation: Increasing social interactions in children with autism and other developmental disabilities in inclusive preschool classrooms. *Topics in Early Childhood Special Education*, *22*(1), 26–38. https://doi.org/10.1177/027112140202200103

Garfinkle, A. N. (2000). Using theory-of-mind to increase social competence in young children with autism: A model for praxis in early childhood special education. [ProQuest Information & Learning]. In *Dissertation Abstracts International Section A: Humanities and Social Sciences* (Vol. 60, Issues 7-A).

Geckeler, A. S., Libby, M. E., Graff, R. B., & Ahearn, W. H. (2000). Effects of reinforcer choice measured in single-operant and concurrent-schedule procedures. *Journal of Applied Behavior Analysis*, *33*(3), 347–351. https://doi.org/10.1901/jaba.2000.33-347

Geiger, K. B., LeBlanc, L. A., Dillon, C. M., & Bates, S. L. (2010). An evaluation of preference for video and in vivo modeling. *Journal of Applied Behavior Analysis*, *43*(2), 279–283. https://doi.org/10.1901/jaba.2010.43-279

Gena, A. (2006). The effects of prompting and social reinforcement on establishing social interactions with peers during the inclusion of four children with autism in preschool. *International Journal of Psychology*, *41*(6), 541–554. https://doi.org/10.1080/00207590500492658

Gena, A., Couloura, S., & Kymissis, E. (2005). Modifying the affective behavior of preschoolers with autism using in-vivo or video modeling and reinforcement contingencies. *Journal of Autism and Developmental Disorders*, *35*(5), 545–556. https://doi.org/10.1007/s10803-005-0014-9

Gevarter, C., O’Reilly, M. F., Kuhn, M., Mills, K., Ferguson, R., Watkins, L., Sigafoos, J., Lang, R., Rojeski, L., & Lancioni, G. E. (2016). Increasing the vocalizations of individuals with autism during intervention with a speech‐generating device. *Journal of Applied Behavior Analysis*, *49*(1), 17–33. https://doi.org/10.1002/jaba.270

Ghaemmaghami, M., Hanley, G. P., Jessel, J., & Landa, R. (2018). Shaping complex functional communication responses. *Journal of Applied Behavior Analysis*, *51*(3), 502–520. https://doi.org/10.1002/jaba.468

Gibbs, A. R., Tullis, C. A., Thomas, R., & Elkins, B. (2018). The effects of noncontingent music and response interruption and redirection on vocal stereotypy. *Journal of Applied Behavior Analysis*, *51*(4), 899–914. https://doi.org/10.1002/jaba.485

Glodowski, K. R., & Rodriguez, N. M. (2019). The effects of scenic picture prompts on variability during the acquisition of intraverbal categorization for children with autism. *Analysis of Verbal Behavior*, *35*(2), 134–148. https://doi.org/10.1007/s40616-019-00120-2

Gokey, K. M., Wilder, D. A., Welch, T., Collier, A., & Mathisen, D. (2013). Fading a concurrent activity during self‐control training for children with autism. *Journal of Applied Behavior Analysis*, *46*(4), 827–831. https://doi.org/10.1002/jaba.77

Goldstein, H., & Cisar, C. L. (1992). Promoting Interaction During Sociodramatic Play: Teaching Scripts To Typical Preschoolers and Classmates With Disabilities. *Journal of Applied Behavior Analysis*, *25*(2), 265–280. https://doi.org/10.1901/jaba.1992.25-265

Goldstein, H., Kaczmarek, L., Pennington, R., & Shafer, K. (1992). Peer-mediated intervention: Attending to, commenting on, and acknowledging the behavior of preschoolers with autism. *Journal of Applied Behavior Analysis*, *25*(2), 289–305. https://doi.org/10.1901/jaba.1992.25-289

Gomes, S. R., Reeve, S. A., Brothers, K. J., Reeve, K. F., & Sidener, T. M. (2020). Establishing a generalized repertoire of initiating bids for joint attention in children with autism. *Behavior Modification*, *44*(3), 394–428. https://doi.org/10.1177/0145445518822499

Gongola, L. C. (2009). The influence of a differential reinforcement of other behaviors (DRO) protocol with an embedded token economy to reduce challenging behaviors among children with autism. [ProQuest Information & Learning]. In *Dissertation Abstracts International Section A: Humanities and Social Sciences* (Vol. 69, Issues 11-A).

Gorgan, E. M., & Kodak, T. (2019). Comparison of interventions to treat prompt dependence for children with developmental disabilities. *Journal of Applied Behavior Analysis*, *52*(4), 1049–1063. https://doi.org/10.1002/jaba.638

Graff, R. B., Green, G., & Libby, M. E. (1998). Effects of two levels of treatment intensity on a young child with severe disabilities. *Behavioral Interventions*, *13*(1), 21–41. https://doi.org/10.1002/(SICI)1099-078X(199802)13:1<21::AID-BIN2>3.3.CO;2-3

Graff, R. B., & Libby, M. E. (1999). A comparison of presession and within-session reinforcement choice. *Journal of Applied Behavior Analysis*, *32*(2), 161–173. https://doi.org/10.1901/jaba.1999.32-161

Grannan, L., & Rehfeldt, R. A. (2012). Emergent intraverbal responses via tact and match-to-sample instruction. *Journal of Applied Behavior Analysis*, *45*(3), 601–605. https://doi.org/10.1901/jaba.2012.45-601

Granpeesheh, D., Tarbox, J., Dixon, D. R., Carr, E., & Herbert, M. (2009). Retrospective analysis of clinical records in 38 cases of recovery from autism. *Annals of Clinical Psychiatry*, *21*(4), 195–204.

Green, G., Brennan, L. C., & Fein, D. (2002). Intensive behavioral treatment for a toddler at high risk for autism. *Behavior Modification*, *26*(1), 69–102. https://doi.org/10.1177/0145445502026001005

Greener-Wooten, S. (2016). An Evaluation of the effects of multiple-exemplar training on the emergence of a generalized autoclitic repertoire in young children with autism. [ProQuest Information & Learning]. In *Dissertation Abstracts International: Section B: The Sciences and Engineering* (Vol. 77, Issues 2-B(E)).

Greer, R. D., & Du, L. (2010). Generic instruction versus intensive tact instruction and the emission of spontaneous speech. *The Journal of Speech and Language Pathology – Applied Behavior Analysis*, *5*(1), 1–19. https://doi.org/10.1037/h0100261

Grey, I. M., Honan, R., McClean, B., & Daly, M. (2005). Evaluating the effectiveness of teacher training in Applied Behaviour Analysis. *Journal of Intellectual Disabilities : JOID*, *9*(3), 209–227. https://doi.org/10.1177/1744629505056695

Grindle, C. F., Hastings, R. P., Saville, M., Hughes, J. C., Huxley, K., Kovshoff, H., Griffith, G. M., Walker-Jones, E., Devonshire, K., & Remington, B. (2012). Outcomes of a behavioral education model for children with autism in a mainstream school setting. *Behavior Modification*, *36*(3), 298–319. https://doi.org/10.1177/0145445512441199

Grindle, C. F., & Remington, B. (2005). Teaching Children with Autism when Reward is Delayed. The Effects of Two Kinds of Marking Stimuli. *Journal of Autism and Developmental Disorders*, *35*(6), 839–850. https://doi.org/10.1007/s10803-005-0029-2

Grindle, C. F., & Remington, B. (2002). Discrete-trial training for autistic children when reward is delayed: A comparison of conditioned cue value and response marking. *Journal of Applied Behavior Analysis*, *35*(2), 187–190. https://doi.org/10.1901/jaba.2002.35-187

Groskreutz, M. P., Groskreutz, N. C., & Higbee, T. S. (2011). Response competition and stimulus preference in the treatment of automatically reinforced behavior: A comparison. *Journal of Applied Behavior Analysis*, *44*(1), 211–215. https://doi.org/10.1901/jaba.2011.44-211

Groskreutz, M. P., Peters, A., Groskreutz, N. C., & Higbee, T. S. (2015). Increasing play‐based commenting in children with autism spectrum disorder using a novel script‐frame procedure. *Journal of Applied Behavior Analysis*, *48*(2), 442–447. https://doi.org/10.1002/jaba.194

Groskreutz, N. C., Groskreutz, M. P., Bloom, S. E., & Slocum, T. A. (2014). Generalization of negatively reinforced mands in children with autism. *Journal of Applied Behavior Analysis*, *47*(3), 560–579. https://doi.org/10.1002/jaba.151

Groskreutz, N. C., Karsina, A. I. I., Miguel, C. F., & Groskreutz, M. P. (2010). Using complex auditory-visual samples to produce emergent relations in children with autism. *Journal of Applied Behavior Analysis*, *43*(1), 131–136. https://doi.org/10.1901/jaba.2010.43-131

Grow, L. L., Carr, J. E., Kodak, T. M., Jostad, C. M. ., & Kisamore, A. N. (2011). A comparison of methods for teaching receptive labeling to children with autism spectrum disorders. *Journal of Applied Behavior Analysis*, *44*(3), 475–498. https://doi.org/10.1901/jaba.2011.44-475

Grow, L. L., Kodak, T., & Carr, J. E. (2014). A comparison of methods for teaching receptive labeling to children with autism spectrum disorders: A systematic replication. *Journal of Applied Behavior Analysis*, *47*(3), 600–605. https://doi.org/10.1002/jaba.141

Grow, L. L., & Van Der Hijde, R. (2017). A comparison of procedures for teaching receptive labeling of sight words to a child with autism spectrum disorder. *Behavior Analysis Practice*, *10*(1), 62–66. https://doi.org/10.1007/s40617-016-0133-0

Gunby, K. V, Carr, J. E., & Leblanc, L. A. (2010). Teaching abduction-prevention skills to children with autism. *Journal of Applied Behavior Analysis*, *43*(1), 107–112. https://doi.org/10.1901/jaba.2010.43-107

Gunby, K. V, & Rapp, J. T. (2014). The use of behavioral skills training and in situ feedback to protect children with autism from abduction lures. *Journal of Applied Behavior Analysis*, *47*(4), 856–860. https://doi.org/10.1002/jaba.173

Gunby, K. V, Rapp, J. T., & Bottoni, M. M. (2018). A progressive model for teaching children with autism to follow gaze shift. *Journal of Applied Behavior Analysis*, *51*(3), 694–701. https://doi.org/10.1002/jaba.479

Gunning, C., Holloway, J., & Grealish, L. (2020). An evaluation of parents as behavior change agents in the preschool life skills program. *Journal of Applied Behavior Analysis*, *53*(2), 889–917. https://doi.org/10.1002/jaba.660

Guptill, D. (2019). Teaching adolescent students with autism spectrum disorders to respond to and generate disguised mands [ProQuest Information & Learning]. In *Dissertation Abstracts International: Section B: The Sciences and Engineering* (Vol. 80, Issues 8-B(E)).

Gutierrez, A. J., Hale, M. N., O’Brien, H. A., Fischer, A. J., Durocher, J. S., & Alessandri, M. (2009). Evaluating the effectiveness of two commonly used discrete trial procedures for teaching receptive discrimination to young children with autism spectrum disorders. *Research in Autism Spectrum Disorders*, *3*(3), 630–638. https://doi.org/10.1016/j.rasd.2008.12.005

Gutierrez, A. J., Vollmer, T. R., Dozier, C. L., Borrero, J. C., Rapp, J. T., Bourret, J. C., & Gadaire, D. (2007). Manipulating establishing operations to verify and establish stimulus control during mand training. *Journal of Applied Behavior Analysis*, *40*(4), 645–658. https://doi.org/10.1901/jaba.2007.645-658

Guzinski, E. M., Cihon, T. M., & Eshleman, J. (2012). The effects of tact training on stereotypic vocalizations in children with autism. *Analysis of Verbal Behavior*, *28*(1), 101–110. https://doi.org/10.1007/BF03393110

Hanley, G. P., Jin, C. S., Vanselow, N. R., & Hanratty, L. A. (2014). Producing meaningful improvements in problem behavior of children with autism via synthesized analyses and treatments. *Journal of Applied Behavior Analysis*, *47*(1), 16–36. https://doi.org/10.1002/jaba.106

Hanney, N. M., Carr, J. E., & LeBlanc, L. A. (2019). Teaching children with autism spectrum disorder to tact auditory stimuli. *Journal of Applied Behavior Analysis*, *52*(3), 733–738. https://doi.org/10.1002/jaba.605

Hannula, C., Jimenez-Gomez, C., Wu, W., Brewer, A. T., Kodak, T., Gilroy, S. P., Hutsell, B. A., Alsop, B., & Podlesnik, C. A. (2020). Quantifying errors of bias and discriminability in conditional-discrimination performance in children diagnosed with autism spectrum disorder. *Learning and Motivation*, *71*. https://doi.org/10.1016/j.lmot.2020.101659

Haq, S. S., & Aranki, J. (2019). Comparison of traditional and embedded DTT on problem behavior and responding to instructional targets. *Behavior Analysis in Practice*, *12*(2), 396–400. https://doi.org/10.1007/s40617-018-00324-3

Haq, S. S., Kodak, T., Kurtz‐Nelson, E., Porritt, M., Rush, K., & Cariveau, T. (2015). Comparing the effects of massed and distributed practice on skill acquisition for children with autism. *Journal of Applied Behavior Analysis*, *48*(2), 454–459. https://doi.org/10.1002/jaba.213

Harris, S. L., Handleman, J. S., Gordon, R., Kristoff, B., & Fuentes, F. (1991). Changes in cognitive and Language functioning of Preschool children with autism. *Journal of Autism and Developmental Disorders*, *21*(3), 281–290. https://doi.org/10.1007/BF02207325

Hartz, R. M., Luiselli, J. K., & Harper, J. M. (2020). Behavioral treatment of spitting in a child with autism spectrum disorder: Functional analysis, intervention evaluation, and maintenance assessment. *Behavioral Interventions*, *35*(4), 595–603. https://doi.org/10.1002/bin.1739

Hatzenbuhler, E. G., Molteni, J. D., & Axe, J. B. (2019). Increasing Play Skills in Children with Autism Spectrum Disorder via Peer-Mediated Matrix Training. *Education and Treatment of Children*, *42*(3), 295–319. https://doi.org/doi:10.1353/etc.2019.0014.

Hayward, D., Eikeseth, S., Gale, C., & Morgan, S. (2009). Assessing progress during treatment for young children with autism receiving intensive behavioural interventions. *Autism : The International Journal of Research and Practice*, *13*(6), 613–633. https://doi.org/10.1177/1362361309340029

Heckaman, K. A., Alber, S., Hooper, S., & Heward, W. L. (1998). A Comparison of Least-to-Most Prompts and Progressive Time Delay on the Disruptive Behavior of Students with Autism Author ( s ): Kelly A . Heckaman , Sheila Alber , Sonya Hooper and William L . Heward. *Journal of Behavioral Education*, *8*(2), 171–201. https://doi.org/https://doi.org/10.1023/A:1022883523915

Hedquist, C. B., & Roscoe, E. M. (2020). A comparison of differential reinforcement procedures for treating automatically reinforced behavior. *Journal of Applied Behavior Analysis*, *53*(1), 284–295. https://doi.org/10.1002/jaba.561

Heldt, J., & Schlinger, H. D. J. (2012). Increased variability in tacting under a Lag 3 schedule of reinforcement. *Analysis of Verbal Behavior*, *28*(1), 131–136. https://doi.org/10.1007/BF03393114

Hicks, S. C., Bethune, K. S., Wood, C. L., Cooke, N. L., & Mims, P. J. (2011). Effects of direct instruction on the acquisition of prepositions by students with intellectual disabilities. *Journal of Applied Behavior Analysis*, *44*(3), 675–679. https://doi.org/10.1901/jaba.2011.44-675

Hill, K. E., Griffith, K. R., & Miguel, C. F. (2020). Using equivalence‐based instruction to teach piano skills to children. *Journal of Applied Behavior Analysis*, *53*(1), 188–208. https://doi.org/10.1002/jaba.547

Hilton, J. C., & Seal, B. C. (2007). Brief report: Comparative ABA and DIR trials in twin brothers with autism. *Journal of Autism and Developmental Disorders*, *37*, 1197–1201. https://doi.org/10.1007/s10803-006-0258-z

Hilton, J. C. (2005). Communication skills of young children diagnosed with autism: Comparative effectiveness of applied behavior analysis and developmental, individual-difference, relationship-based interventions [James Madison University]. In *ProQuest Dissertations and Theses*.

Hoch, H., McComas, J. J., Johnson, L., Faranda, N., & Guenther, S. (2002). The effects of magnitude and quality of reinforcement on choice responding during play activities. *Journal of Applied Behavior Analysis*, *35*(2), 171–181. https://doi.org/10.1901/jaba.2002.35-171

Hoch, H., McComas, J. J., Thompson, A. L., & Paone, D. (2002). Concurrent reinforcement schedules: Behavior change and maintenance without extinction. *Journal of Applied Behavior Analysis*, *35*(2), 155–169. https://doi.org/10.1901/jaba.2002.35-155

Hoffman, K., & Falcomata, T. S. (2014). An evaluation of resurgence of appropriate communication in individuals with autism who exhibit severe problem behavior. *Journal of Applied Behavior Analysis*, *47*(3), 651–656. https://doi.org/10.1002/jaba.144

Hood, S. A., Luczynski, K. C., & Mitteer, D. R. (2017). Toward meaningful outcomes in teaching conversation and greeting skills with individuals with autism spectrum disorder. *Journal of Applied Behavior Analysis*, *50*(3), 459–486. https://doi.org/10.1002/jaba.388

Hood, S. A., Olsen, A. E., Luczynski, K. C., & Randle, F. A. (2020). Improving accepting and giving compliments with individuals with developmental disabilities. *Journal of Applied Behavior Analysis*, *53*(2), 1013–1028. https://doi.org/https://doi.org/10.1002/jaba.662

Horner, R. H., Day, H. M., & Day, J. R. (1997). Using neutralizing routines to reduce problem behaviors. *Journal of Applied Behavior Analysis*, *30*(4), 601–614. https://doi.org/10.1901/jaba.1997.30-601

Horton, J. A. (2020). The effects of the social-listener protocol on the observing, helping, and vocal behavior of children with ASD [ProQuest Information & Learning]. In *Dissertation Abstracts International Section A: Humanities and Social Sciences* (Vol. 81, Issues 9-A).

Howard, J. S., Sparkman, C. R., Cohen, H. G., Green, G., & Stanislaw, H. (2005). A comparison of intensive behavior analytic and eclectic treatments for young children with autism. *Research in Developmental Disabilities*, *26*(4), 359–383. https://doi.org/10.1016/j.ridd.2004.09.005

Howard, J. S., Stanislaw, H., Green, G., Sparkman, C. R., & Cohen, H. G. (2014). Comparison of behavior analytic and eclectic early interventions for young children with autism after three years. *Research in Developmental Disabilities*, *35*(12), 3326–3344. https://doi.org/10.1016/j.ridd.2014.08.021

Humphreys, T., Polick, A. S., Howk, L. L., Thaxton, J. R., & Ivancic, A. P. (2013). An evaluation of repeating the discriminative stimulus when using least-to-most prompting to teach intraverbal behavior to children with autism. *Journal of Applied Behavior Analysis*, *46*(2), 534–538. https://doi.org/10.1002/jaba.43

Hundert, J., Rowe, S., & Harrison, E. (2014). The combined effects of social script training and peer buddies on generalized peer interaction of children with ASD in inclusive classrooms. *Focus on Autism and Other Developmental Disabilities*, *29*(4), 206–215. https://doi.org/10.1177/1088357614522288

Huskens, B., Reijers, H., & Didden, R. (2012). Staff training effective in increasing learning opportunities for school-aged children with autism spectrum disorders. *Developmental Neurorehabilitation*, *15*(6), 435–447. https://doi.org/10.3109/17518423.2012.705910

Huskens, B., Verschuur, R., Gillesen, J., Didden, R., & Barakova, E. (2013). Promoting question-asking in school-aged children with autism spectrum disorders: effectiveness of a robot intervention compared to a human-trainer intervention. *Developmental Neurorehabilitation*, *16*(5), 345–356. https://doi.org/10.3109/17518423.2012.739212

Hutchison-Harris, J. (2004). Does first year treatment intensity predict outcome in young autistic children receiving Lovaas ABA intervention? [ProQuest Information & Learning]. In *Dissertation Abstracts International: Section B: The Sciences and Engineering* (Vol. 65, Issues 5-B).

Ilg, J., Jebrane, A., Paquet, A., Rousseau, M., Dutray, B., Wolgensinger, L., & Clément, C. (2018). Evaluation of a French parent-training program in young children with autism spectrum disorder. *Psychologie Française*, *63*(2), 181–199. https://doi.org/10.1016/j.psfr.2016.12.004

Ingenmey, R., & Van Houten, R. (1991). Using time delay to promote spontaneous speech in an autistic child. *Journal of Applied Behavior Analysis*, *24*(3), 591–596. https://doi.org/10.1901/jaba.1991.24-591

Ingersoll, B., & Gergans, S. (2007). The effect of a parent-implemented imitation intervention on spontaneous imitation skills in young children with autism. *Research in Developmental Disabilities*, *28*(2), 163–175. https://doi.org/10.1016/j.ridd.2006.02.004

Ingersoll, B., & Schreibman, L. (2006). Teaching reciprocal imitation skills to young children with autism using a naturalistic behavioral approach: Effects on language, pretend play, and joint attention. *Journal of Autism and Developmental Disorders*, *36*(4), 487–505. https://doi.org/10.1007/s10803-006-0089-y

Ingersoll, B., Schreibman, L., & Stahmer, A. (2001). Brief Report: Differential Treatment Outcomes for Children with Autistic Spectrum Disorder Based on Level of Peer Social Avoidance. *Journal of Autism and Developmental Disorders*, *31*(3), 343–349. https://doi.org/10.1023/A:1010703521704

Ingvarsson, E. T., & Hollobaugh, T. (2011). A comparison of prompting tactics to establish intraverbals in children with autism. *Journal of Applied Behavior Analysis*, *44*(3), 659–664. https://doi.org/10.1901/jaba.2011.44-659

Ingvarsson, E. T., & Hollobaugh, T. (2010). Acquisition of intraverbal behavior: Teaching children with autism to mand for answers to questions. *Journal of Applied Behavior Analysis*, *43*(1), 1–17. https://doi.org/10.1901/jaba.2010.43-1

Jahr, E. (2001). Teaching children with autism to answer novel wh-questions by utilizing a multiple exemplar strategy. *Research in Developmental Disabilities*, *22*(5), 407–423. https://doi.org/10.1016/S0891-4222(01)00081-6

Jahr, E., & Eldevik, S. (2007). Changes in solitary play following acquisition of cooperative play by children with autism. *The Journal of Speech and Language Pathology – Applied Behavior Analysis*, *2*(2), 182–189. https://doi.org/10.1037/h0100215

Jansson, B. S., Miniscalco, C., Westerlund, J., Kantzer, A.-K., Fernell, E., & Gillberg, C. (2016). Children who screen positive for autism at 2.5 years and receive early intervention: A prospective naturalistic 2-year outcome study. *Neuropsychiatric Disease and Treatment*, *12*, 2255–2263. https://doi.org/10.2147/NDT.S108899

Jeffries, T., Crosland, K., & Miltenberger, R. (2016). Evaluating a tablet application and differential reinforcement to increase eye contact in children with autism. *Journal of Applied Behavior Analysis*, *49*(1), 182–187. https://doi.org/10.1002/jaba.262

Jessel, J., Hanley, G. P., & Ghaemmaghami, M. (2016). A translational evaluation of transitions. *Journal of Applied Behavior Analysis*, *49*(2), 359–376. https://doi.org/10.1002/jaba.283

Jessel, J., Ingvarsson, E. T., Metras, R., Kirk, H., & Whipple, R. (2018). Achieving socially significant reductions in problem behavior following the interview‐informed synthesized contingency analysis: A summary of 25 outpatient applications. *Journal of Applied Behavior Analysis*, *51*(1), 130–157. https://doi.org/10.1002/jaba.436

Jessel, J., Ingvarsson, E. T., Whipple, R., & Kirk, H. (2017). Increasing on‐task behavior of an adolescent with autism using momentary differential reinforcement. *Behavioral Interventions*, *32*(3), 248–254. https://doi.org/10.1002/bin.1480

Jessel, J., Ma, S., Spartinos, J., & Villanueva, A. (2020). Transitioning from rich to lean reinforcement as a form of error correction. *Journal of Applied Behavior Analysis*, *53*(4), 2108–2125. https://doi.org/10.1002/jaba.717

Jin, C. S., Hanley, G. P., & Beaulieu, L. (2013). An individualized and comprehensive approach to treating sleep problems in young children. *Journal of Applied Behavior Analysis*, *46*(1), 161–180. https://doi.org/10.1002/jaba.16

Jobin, A. (2020). Varied treatment response in young children with autism: A relative comparison of structured and naturalistic behavioral approaches. *Autism: The International Journal of Research & Practice*, *24*(2), 338–351. https://doi.org/10.1177/1362361319859726

Johnson, L., McComas, J., Thompson, A., & Symons, F. J. (2004). Obtained versus programmed reinforcement: Practical considerations in the treatment of escape-reinforced aggression. *Journal of Applied Behavior Analysis*, *37*(2), 239–242. https://doi.org/10.1901/jaba.2004.37-239

Jones, E. A., Carr, E. G., & Feeley, K. M. (2006). Multiple effects of joint attention intervention for children with autism. *Behavior Modification*, *30*(6), 782–834. https://doi.org/10.1177/0145445506289392

Jones, E. A., & Feeley, K. M. (2009). Parent implemented joint attention intervention for preschoolers with autism. *The Journal of Speech and Language Pathology – Applied Behavior Analysis*, *4*(1), 74–89. https://doi.org/10.1037/h0100251

Jones, E. A., Feeley, K. M., & Takacs, J. (2007). Teaching spontaneous responses to young children with autism. *Journal of Applied Behavior Analysis*, *40*(3), 565–570. https://doi.org/10.1901/jaba.2007.40-565

Jones, J., Lerman, D. C., & Lechago, S. (2014). Assessing stimulus control and promoting generalization via video modeling when teaching social responses to children with autism. *Journal of Applied Behavior Analysis*, *47*(1), 37–50. https://doi.org/10.1002/jaba.81

Jorgenson, C. D., Clay, C. J., & Kahng, S. (2020). Evaluating preference for and reinforcing efficacy of a therapy dog to increase verbal statements. *Journal of Applied Behavior Analysis*, *53*(3), 1419–1431. https://doi.org/10.1002/jaba.668

Jung, S., Sainato, D. M., & Davis, C. A. (2008). Using high-probability request sequences to increase social interactions in young children with autism. *Journal of Early Intervention*, *30*(3), 163–187. https://doi.org/10.1177/1053815108317970

Kahlow, T. A., Sidener, T. M., Kisamore, A. N., & Reeve, K. F. (2019). Teaching the mand “when?” to children with autism spectrum disorder. *Analysis of Verbal Behavior*, *35*(2), 221–234. https://doi.org/10.1007/s40616-019-00115-z

Kalgotra, R., Warwal, J. S., & Teji, V. (2019). Social development of children with mild and moderate Intellectual Disabilities at special schools in India. *Life Span and Disability*, *22*(1), 29–53.

Kalgotra, R., & Warwal, J. S. (2019). Effect of intervention in teaching listening and speaking skills on children with intellectual disabilities. *Journal of Educational, Cultural and Psychological Studies*, *20*, 21–39.

Kamio, Y., Haraguchi, H., Miyake, A., & Hiraiwa, M. (2015). Brief report: Large individual variation in outcomes of autistic children receiving low-intensity behavioral interventions in community settings. *Child and Adolescent Psychiatry and Mental Health*, *9*. https://doi.org/10.1186/s13034-015-0039-6

Karanth, P., Shaista, S., & Srikanth, N. (2010). Efficacy of Communication DEALL-An Indigenous Early Intervention Program for Children with Autism Spectrum Disorders. *Indian Journal of Pediatrics*, *77*, 957–962. https://doi.org/10.1007/s12098-010-0144-8

Karsten, A. M., & Carr, J. E. (2009). The effects of differential reinforcement of unprompted responding on the skill acquisition of children with autism. *Journal of Applied Behavior Analysis*, *42*(2), 327–334. https://doi.org/10.1901/jaba.2009.42-327

Kassardjian, A., Leaf, J. A., Leaf, J. B., Townley-Cochran, D., Alcalay, A., Milne, C., Dale, S., Tsuji, K., Leaf, R., Taubman, M., & McEachin, J. (2016). Evaluation of graduated vs all-or-none contingencies on rate tasks for individuals diagnosed with autism. *Education and Training in Autism and Developmental Disabilities*, *51*(4), 434–446.

Kay, J. C., Kisamore, A. N., Vladescu, J. C., Sidener, T. M., Reeve, K. F., Taylor‐Santa, C., & Pantano, N. A. (2020). Effects of exposure to prompts on the acquisition of intraverbals in children with autism spectrum disorder. *Journal of Applied Behavior Analysis*, *53*(1), 493–507. https://doi.org/10.1002/jaba.606

Keen, D., Sigafoos, J., & Woodyatt, G. (2001). Replacing Prelinguistic Behaviors with Functional Communication. *Journal of Autism and Developmental Disorders*, *31*(4), 385–398. https://doi.org/10.1023/A:1010612618969

Keintz, K. S., Miguel, C. F., Kao, B., & Finn, H. E. (2011). Using conditional discrimination training to produce emergent relations between coins and their values in children with autism. *Journal of Applied Behavior Analysis*, *44*(4), 909–913. https://doi.org/10.1901/jaba.2011.44-909

Kelley, M. E., Lerman, D. C., & Van Camp, C. M. (2002). The effects of competing reinforcement schedules on the acquisition of functional communication. *Journal of Applied Behavior Analysis*, *35*(1), 59–63. https://doi.org/10.1901/jaba.2002.35-59

Kelley, M. E., Shillingsburg, M. A., Castro, M. J., Addison, L. R., & LaRue, R. H. J. (2007). Further evaluation of emerging speech in children with developmental disabilities: Training verbal behavior. *Journal of Applied Behavior Analysis*, *40*(3), 431–445. https://doi.org/10.1901/jaba.2007.40-431

Kelly, S., Green, G., & Sidman, M. (1998). Visual identity matching and auditory-visual matching: A procedural note. *Journal of Applied Behavior Analysis*, *31*(2), 237–243. https://doi.org/10.1901/jaba.1998.31-237

Keohane, D.-D., Luke, N., & Greer, R. D. (2008). The things we care to see: The effects of rotated protocol immersion on the emergence of early observing responses. *Journal of Early and Intensive Behavior Intervention*, *5*(1), 23–39. https://doi.org/10.1037/h0100408

Kerr, K. P., Campbell, A., & McGrory, S. (2002). The Saplings Model of Education: Case Studies in Autism. *Journal of Precision Teaching & Celeration*, *18*(2), 49–60.

Keyl-Austin, A. A., Samaha, A. L., Bloom, S. E., & Boyle, M. A. (2012). Effects of preference and reinforcer variation on within-session patterns of responding. *Journal of Applied Behavior Analysis*, *45*(3), 637–641. https://doi.org/10.1901/jaba.2012.45-637

Kisamore, A. N., Karsten, A. M., & Mann, C. C. (2016). Teaching multiply controlled intraverbals to children and adolescents with autism spectrum disorders. *Journal of Applied Behavior Analysis*, *49*(4), 826–847. https://doi.org/10.1002/jaba.344

Kittenbrink, R. L. (2016). The effects of peer to peer mand training on unprompted mand frequency for children with autism and intellectual/ developmental disabilities. [ProQuest Information & Learning]. In *Dissertation Abstracts International Section A: Humanities and Social Sciences* (Vol. 77, Issues 4-A(E)).

Knutson, S. C., Kodak, T., Costello, D. R., & Cliett, T. (2019). Comparison of task interspersal ratios on efficiency of learning and problem behavior for children with autism spectrum disorder. *Journal of Applied Behavior Analysis*, *52*(2), 355–369. https://doi.org/10.1002/jaba.527

Kobari-Wright, V. V, & Miguel, C. F. (2014). The Effects of Listener Training on the Emergence of Categorization and Speaker Behavior in Children with Autism. *Journal of Applied Behavior Analysis*, *47*(2), 431–436. https://doi.org/10.1002/jaba.115

Kodak, T., Campbell, V., Bergmann, S., LeBlanc, B., Kurtz‐Nelson, E., Cariveau, T., Haq, S., Zemantic, P., & Mahon, J. (2016). Examination of efficacious, efficient, and socially valid error‐correction procedures to teach sight words and prepositions to children with autism spectrum disorder. *Journal of Applied Behavior Analysis*, *49*(3), 532–547. https://doi.org/10.1002/jaba.310

Kodak, T., & Clements, A. (2009). Acquisition of mands and tacts with concurrent echoic training. *Journal of Applied Behavior Analysis*, *42*(4), 839–843. https://doi.org/10.1901/jaba.2009.42-839

Kodak, T., Clements, A., Paden, A. R., LeBlanc, B., Mintz, J., & Toussaint, K. A. (2015). Examination of the relation between an assessment of skills and performance on auditory–visual conditional discriminations for children with autism spectrum disorder. *Journal of Applied Behavior Analysis*, *48*(1), 52–70. https://doi.org/10.1002/jaba.160

Kodak, T., Fuchtman, R., & Paden, A. (2012). A comparison of intraverbal training procedures for children with autism. *Journal of Applied Behavior Analysis*, *45*(1), 155–160. https://doi.org/10.1901/jaba.2012.45-155

Kodak, T., Halbur, M., Bergmann, S., Costello, D. R., Benitez, B., Olsen, M., Gorgan, E., & Cliett, T. (2020). A comparison of stimulus set size on tact training for children with autism spectrum disorder. *Journal of Applied Behavior Analysis*, *53*(1), 265–283. https://doi.org/10.1002/jaba.553

Kodak, T., Lerman, D. C., & Call, N. (2007). Evaluating the influence of postsession reinforcement on choice of reinforcers. *Journal of Applied Behavior Analysis*, *40*(3), 515–527. https://doi.org/10.1901/jaba.2007.40-515

Kodak, T., Miltenberger, R. G., & Romaniuk, C. (2003). A comparison of differential reinforcement and noncontingent reinforcement for the treatment of a child’s multiply controlled problem behavior. *Behavioral Interventions*, *18*(4), 267–278. https://doi.org/10.1002/bin.143

Kodak, T., Miltenberger, R. G., & Romaniuk, C. (2003). The effects of differential negative reinforcement of other behavior and noncontingent escape on compliance. *Journal of Applied Behavior Analysis*, *36*(3), 379–382. https://doi.org/10.1901/jaba.2003.36-379

Kodak, T., & Paden, A. R. (2015). A comparison of intraverbal and listener training for children with autism spectrum disorder. *Analysis of Verbal Behavior*, *31*, 137–144. https://doi.org/10.1007/s40616-015-0033-3

Kodak, T., Paden, A., & Dickes, N. (2012). Training and generalization of peer-directed mands with non-vocal children with autism. *Analysis of Verbal Behavior*, *28*, 119–124. https://doi.org/10.1007/BF03393112

Koegel, L. K., Camarata, S. M., Valdez-Menchaca, M., & Koegel, R. L. (1998). Setting generalization of question-asking by children with autism. *American Journal on Mental Retardation*, *102*(4), 346–357. https://doi.org/10.1352/0895-8017(1998)102<0346:SGOQBC>2.0.CO;2

Koegel, L. K., Koegel, R. L., Hurley, C., & Frea, W. D. (1992). Improving Social Skills and Disruptive Behavior in Children With Autism Through Self-Management. *Journal of Applied Behavior Analysis*, *25*(2), 341–353. https://doi.org/10.1901/jaba.1992.25-341

Koegel, L. K., Koegel, R. L., Shoshan, Y., & McNerney, E. (1999). Pivotal response intervention II: Preliminary long-term outcome data. *Journal of the Association for Persons with Severe Handicaps*, *24*(3), 186–198. https://doi.org/10.2511/rpsd.24.3.186

Koegel, R. L., & Frea, W. D. (1993). Treatment of Social Behavior in Autism Through the Modification of Pivotal Social Skills. *Journal of Applied Behavior Analysis*, *26*(3), 369–377. https://doi.org/10.1901/jaba.1993.26-369

Koenig, K., Williams White, S., Pachler, M., Lau, M., Lewis, M., Klin, A., & Scahill, L. (2010). Promoting social skill development in children with pervasive developmental disorders: A feasibility and efficacy study. *Journal of Autism and Developmental Disorders*, *40*, 1209–1218. https://doi.org/10.1007/s10803-010-0979-x

Kovshoff, H., Hastings, R. P., & Remington, B. (2011). Two-year outcomes for children with autism after the cessation of early intensive behavioral intervention. *Behavior Modification*, *35*(5), 427–450. https://doi.org/10.1177/0145445511405513

Krantz, P. J., & McClannahan, L. E. (1998). Social interaction skills for children with autism: A script-fading procedure for beginning readers. *Journal of Applied Behavior Analysis*, *31*(2), 191–202. https://doi.org/10.1901/jaba.1998.31-191

Kroeger, K. A., & Nelson, W. M. 3rd. (2006). A language programme to increase the verbal production of a child dually diagnosed with Down syndrome and autism. *Journal of Intellectual Disability Research*, *50*(2), 101–108. https://doi.org/10.1111/j.1365-2788.2005.00734.x

Kroeger, K. A., Schultz, J. R., & Newsom, C. (2007). A comparison of two group-delivered social skills programs for young children with autism. *Journal of Autism and Developmental Disorders*, *37*, 808–817. https://doi.org/10.1007/s10803-006-0207-x

Kuhn, L. R., Bodkin, A. E., Devlin, S. D., & Doggett, R. A. (2008). Using pivotal response training with peers in special education to facilitate play in two children with autism. *Education and Training in Developmental Disabilities*, *43*(1), 37–45.

Kuhn, S. A. C., Lerman, D. C., Vorndran, C. M., & Addison, L. (2006). Analysis of factors that affect responding in a two-response chain in children with developmental disabilities. *Journal of Applied Behavior Analysis*, *39*(3), 263–280. https://doi.org/10.1901/jaba.2006.118-05

Kuntz, E. M., Santos, A. V, & Kennedy, C. H. (2020). Functional analysis and intervention of perseverative speech in students with high-functioning autism and related neurodevelopmental disabilities. *Journal of Applied Behavior Analysis*, *53*(4), 2421–2428. https://doi.org/10.1002/jaba.669

Kuoch, H., & Mirenda, P. (2003). Social Story Interventions for Young Children with Autism Spectrum Disorders. *Focus on Autism and Other Developmental Disabilities*, *18*(4), 219–227. https://doi.org/10.1177/10883576030180040301

Lafasakis, M., & Sturmey, P. (2007). Training parent implementation of discrete-trial teaching: Effects on generalization of parent teaching and child correct responding. *Journal of Applied Behavior Analysis*, *40*(4), 685–689. https://doi.org/10.1901/jaba.2007.685-689

Lambert‐Lee, K. A., Jones, R., O’Sullivan, J., Hastings, R. P., Douglas‐Cobane, E., Esther, T. J., Hughes, C., & Griffith, G. (2015). Translating evidence‐based practice into a comprehensive educational model within an autism‐specific special school. *British Journal of Special Education*, *42*(1), 69–86. https://doi.org/10.1111/1467-8578.12090

Landa, R. J., Holman, K. C., O’Neill, A. H., & Stuart, E. A. (2011). Intervention targeting development of socially synchronous engagement in toddlers with autism spectrum disorder: a randomized controlled trial. *Journal of Child Psychology and Psychiatry*, *52*(1), 13–21. https://doi.org/10.1111/j.1469-7610.2010.02288.x

Landa, R. K., Frampton, S. E., & Shillingsburg, M. A. (2020). Teaching children with autism to mand for social information. *Journal of Applied Behavior Analysis*, *53*(4), 2271–2286. https://doi.org/10.1002/jaba.733

Landa, R. K., Hansen, B., & Shillingsburg, M. A. (2017). Teaching mands for information using ‘when’ to children with autism. *Journal of Applied Behavior Analysis*, *50*(3), 538–551. https://doi.org/10.1002/jaba.387

Landa, R., & Hanley, G. P. (2016). An evaluation of multiple‐schedule variations to reduce high‐rate requests in the picture exchange communication system. *Journal of Applied Behavior Analysis*, *49*(2), 388–393. https://doi.org/10.1002/jaba.285

Lane, K. L., Thompson, A., Reske, C. L., Gable, L. M., & Barton-Arwood, S. (2006). Reducing skin picking via competing activities. *Journal of Applied Behavior Analysis*, *39*(4), 459–462. https://doi.org/10.1901/jaba.2006.62-05

Lang, R., Davis, T., O’Reilly, M., Machalicek, W., Rispoli, M., Sigafoos, J., Lancioni, G., & Regester, A. (2010). Functional analysis and treatment of elopement across two school settings. *Journal of Applied Behavior Analysis*, *43*(1), 113–118. https://doi.org/10.1901/jaba.2010.43-113

Lang, R., O’Reilly, M., Sigafoos, J., Lancioni, G. E., Machalicek, W., Rispoli, M., & White, P. (2009). Enhancing the effectiveness of a play intervention by abolishing the reinforcing value of stereotypy: A pilot study. *Journal of Applied Behavior Analysis*, *42*(4), 889–894. https://doi.org/10.1901/jaba.2009.42-889

Lanovaz, M. J., Rapp, J. T., & Ferguson, S. (2013). Assessment and treatment of vocal stereotypy associated with television: A pilot study. *Journal of Applied Behavior Analysis*, *46*(2), 544–548. https://doi.org/10.1002/jaba.35

Lanovaz, M. J., Rapp, J. T., & Ferguson, S. (2012). The utility of assessing musical preference before implementation of noncontingent music to reduce vocal stereotypy. *Journal of Applied Behavior Analysis*, *45*(4), 845–851. https://doi.org/10.1901/jaba.2012.45-845

Lanovaz, M. J., Sladeczek, I. E., & Rapp, J. T. (2011). Effects of music on vocal stereotypy in children with autism. *Journal of Applied Behavior Analysis*, *44*(3), 647–651. https://doi.org/10.1901/jaba.2011.44-647

Lasater, M. W., & Brady, M. P. (1995). Effects of Video Self-Modeling and Feedback on Task Fluency: A Home-Based Intervention. *Education and Treatment of Children*, *18*(4), 389–407.

Leaf, J. B., Cihon, J. H., Alcalay, A., Mitchell, E., Townley‐Cochran, D., Miller, K., Leaf, R., Taubman, M., & McEachin, J. (2017). Instructive feedback embedded within group instruction for children diagnosed with autism spectrum disorder. *Journal of Applied Behavior Analysis*, *50*(2), 304–316. https://doi.org/10.1002/jaba.375

Leaf, J. B., Cihon, J. H., Ferguson, J. L., Milne, C. M., Leaf, R., & McEachin, J. (2020). Comparing error correction to errorless learning: A randomized clinical trial. *Analysis of Verbal Behavior*, *36*(1), 1–20. https://doi.org/10.1007/s40616-019-00124-y

Leaf, J. B., Dale, S., Kassardjian, A., Tsuji, K. H., Taubman, M., McEachin, J. J., Leaf, R. B., & Oppenheim-Leaf, M. L. (2014). Comparing different classes of reinforcement to increase expressive language for individuals with autism. *Education and Training in Autism and Developmental Disabilities*, *49*(4), 533–546.

Leaf, J. B., Leaf, J. A., Milne, C., Taubman, M., Oppenheim-Leaf, M., Torres, N., Townley-Cochran, D., Leaf, R., McEachin, J., & Yoder, P. (2017). An evaluation of a behaviorally based social skills group for individuals diagnosed with autism spectrum disorder. *Journal of Autism and Developmental Disorders*, *47*(2), 243–259. https://doi.org/10.1007/s10803-016-2949-4

Leaf, J. B., Oppenheim-Leaf, M. L., Call, N. A., Sheldon, J. B., Sherman, J. A., Taubman, M., McEachin, J., Dayharsh, J., & Leaf, R. (2012). Comparing the teaching interaction procedure to social stories for people with autism. *Journal of Applied Behavior Analysis*, *45*(2), 281–298. https://doi.org/10.1901/jaba.2012.45-281

Leaf, J. B., Oppenheim-Leaf, M. L., Leaf, R., Courtemanche, A. B., Taubman, M., McEachin, J., Sheldon, J. B., & Sherman, J. A. (2012). Observational effects on the preferences of children with autism. *Journal of Applied Behavior Analysis*, *45*(3), 473–483. https://doi.org/10.1901/jaba.2012.45-473

Leaf, J. B., Oppenheim‐Leaf, M. L., Townley‐Cochran, D., Leaf, J. A., Alcalay, A., Milne, C., Kassardjian, A., Tsuji, K., Dale, S., Leaf, R., Taubman, M., & McEachin, J. (2016). Changing preference from tangible to social activities through an observation procedure. *Journal of Applied Behavior Analysis*, *49*(1), 49–57. https://doi.org/10.1002/jaba.276

Leaf, J. B., Sheldon, J. B., & Sherman, J. A. (2010). Comparison of simultaneous prompting and no-no prompting in two-choice discrimination learning with children with autism. *Journal of Applied Behavior Analysis*, *43*(2), 215–228. https://doi.org/10.1901/jaba.2010.43-215

Leaf, J. B., Townley‐Cochran, D., Mitchell, E., Milne, C., Alcalay, A., Leaf, J., Leaf, R., Taubman, M., McEachin, J., & Oppenheim‐Leaf, M. L. (2016). Evaluation of multiple‐alternative prompts during tact training. *Journal of Applied Behavior Analysis*, *49*(2), 399–404. https://doi.org/10.1002/jaba.289

LeBlanc, L. A., Miguel, C. F., Cummings, A. R., Goldsmith, T. R., & Carr, J. E. (2003). The effects of three stimulus-equivalence testing conditions on emergent US geography relations of children diagnosed with autism. *Behavioral Interventions*, *18*, 279–289. https://doi.org/10.1002/bin.144

LeBlanc, L. A., Coates, A. M., Daneshvar, S., Charlop-Christy, M. H., Morris, C., & Lancaster, B. M. (2003). Using video modeling and reinforcement to teach perspective-taking skills to children with autism. *Journal of Applied Behavior Analysis*, *36*(2), 253–257. https://doi.org/10.1901/jaba.2003.36-253

Lechago, S. A., Carr, J. E., Grow, L. L., Love, J. R., & Almason, S. M. (2010). Mands for information generalize across establishing operations. *Journal of Applied Behavior Analysis*, *43*(3), 381–395. https://doi.org/10.1901/jaba.2010.43-381

Lechago, S. A., Howell, A., Caccavale, M. N., & Peterson, C. W. (2013). Teaching “how?” mand‐for‐information frames to children with autism. *Journal of Applied Behavior Analysis*, *46*(4), 781–791. https://doi.org/10.1002/jaba.71

Ledbetter-Cho, K., Lang, R., Davenport, K., Moore, M., Lee, A., Howell, A., Drew, C., Dawson, D., Charlop, M. H., Falcomata, T., & O’Reilly, M. (2015). Effects of Script Training on the Peer-to-Peer Communication of Children with Autism Spectrum Disorder. *Journal of Applied Behavior Analysis*, *48*(4), 785–799. https://doi.org/10.1002/jaba.240

Lee, G. T., & Singer-Dudek, J. (2012). Effects of fluency versus accuracy training on endurance and retention of assembly tasks by four adolescents with developmental disabilities. *Journal of Behavioral Education*, *21*(1), 1–17. https://doi.org/10.1007/s10864-011-9142-9

Lee, R., & Sturmey, P. (2006). The Effects of Lag Schedules and Preferred Materials on Variable Responding in Students with Autism. *Journal of Autism and Developmental Disorders*, *36*(3), 421–428. https://doi.org/10.1007/s10803-006-0080-7

Lepper, T. L., Petursdottir, A. I., & Esch, B. E. (2013). Effects of operant discrimination training on the vocalizations of nonverbal children with autism. *Journal of Applied Behavior Analysis*, *46*(3), 656–661. https://doi.org/10.1002/jaba.55

Leung, J.-P., & Wu, K.-I. (1997). Teaching Receptive Naming of Chinese Characters to Children with Autism by Incorporating Echolalia. *Journal of Applied Behavior Analysis*, *30*(1), 59–68. https://doi.org/10.1901/jaba.1997.30-59

Levingston, H. B., Neef, N. A., & Cihon, T. M. (2009). The Effects of Teaching Precurrent Behaviors on Children’s Solution of Multiplication and Division Word Problems. *Journal of Applied Behavior Analysis*, *42*(2), 361–367. https://doi.org/10.1901/jaba.2009.42-361

Liber, D. B., Frea, W. D., & Symon, J. B. G. (2008). Using Time-delay to Improve Social Play Skills with Peers for Children with Autism. *Journal of Autism and Developmental Disorders*, *38*, 312–323. https://doi.org/10.1007/s10803-007-0395-z

Lim, H. A., & Draper, E. (2011). The effects of music therapy incorporated with applied behavior analysis verbal behavior approach for children with autism spectrum disorders. *Journal of Music Therapy*, *48*(4), 532–550. https://doi.org/10.1093/jmt/48.4.532

Lin, F. Y., & Zhu, J. (2020). Comparison of two discrimination methods in teaching Chinese children with autism. *Journal of Applied Behavior Analysis*, *53*(2), 1145–1152. https://doi.org/https://doi.org/10.1002/jaba.652

Lindgren, S., Wacker, D., Suess, A., Schieltz, K., Pelzel, K., Kopelman, T., Lee, J., Romani, P., & Waldron, D. (2016). Telehealth and Autism: Treating Challenging Behavior at Lower Cost. *Pediatrics*, *137*, S167–S175. https://doi.org/10.1542/peds.2015-2851O

Lipschultz, J. L., Wilder, D. A., Ertel, H., & Enderli, A. (2018). The effects of high‐p and low‐p instruction similarity on compliance among young children. *Journal of Applied Behavior Analysis*, *51*(4), 866–878. https://doi.org/10.1002/jaba.482

Loftin, R. L., Odom, S. L., & Lantz, J. F. (2008). Social Interaction and Repetitive Motor Behaviors. *Journal of Autism and Developmental Disorders*, *38*, 1124–1135. https://doi.org/10.1007/s10803-007-0499-5

Longano, J. M., & Greer, R. D. (2015). Is the source of reinforcement for naming multiple conditioned reinforcers for observing responses? *Analysis of Verbal Behavior*, *31*(1), 96–117. https://doi.org/10.1007/s40616-014-0022-y

Lora, C. C., Kisamore, A. N., Reeve, K. F., & Townsend, D. B. (2020). Effects of a problem‐solving strategy on the independent completion of vocational tasks by adolescents with autism spectrum disorder. *Journal of Applied Behavior Analysis*, *53*(1), 175–187. https://doi.org/10.1002/jaba.558

Loughrey, T. O., Betz, A. M., Majdalany, L. M., & Nicholson, K. (2014). Using instructive feedback to teach category names to children with autism. *Journal of Applied Behavior Analysis*, *47*(2), 425–430. https://doi.org/10.1002/jaba.123

Lovaas, O. I. (1987). Behavioral treatment and normal educational and intellectual functioning in young autistic children. *Journal of Consulting and Clinical Psychology*, *55*(1), 3–9. https://doi.org/10.1037/0022-006X.55.1.3

Love, J. J., Miguel, C. F., Fernand, J. K., & LaBrie, J. K. (2012). The effects of matched stimulation and response interruption and redirection on vocal stereotypy. *Journal of Applied Behavior Analysis*, *45*(3), 549–564. https://doi.org/10.1901/jaba.2012.45-549

Love, S. R., Matson, J. L., & West, D. (1990). Mothers as Effective Therapists for Autistic Children’s Phobias. *Journal of Applied Behavior Analysis*, *23*(3), 379–385. https://doi.org/10.1901/jaba.1990.23-379

Luiselli, J. K., O’Malley Cannon, B., Ellis, J. T., & Sisson, R. W. (2000). Home-Based Behavioral Intervention for Young Children with Autism/Pervasive Developmental Disorder: A Preliminary Evaluation of Outcome in Relation to Child Age and Intensity of Service Delivery. *Autism*, *4*(4), 426–438. https://doi.org/10.1177/1362361300004004007

Luiselli, J. K., Wolongevicz, J., Egan, P., Amirault, D., Sciaraffa, N., & Treml, T. (1999). The Family Support Program: Description of a preventive, community-based behavioral intervention for children with pervasive developmental disorders. *Child & Family Behavior Therapy*, *21*(1), 1–18. https://doi.org/10.1300/J019v21n01_01

Luk, H. Y. E., Witts, B. N., & Schulze, K. A. (2019). Lag-lag schedules in response variability research and programming: A feasibility study. *Behavior Analysis: Research and Practice*, *19*(2), 176–189. https://doi.org/10.1037/bar0000102

Lydon, H., Healy, O., & Leader, G. (2011). A comparison of Video Modeling and Pivotal Response Training to teach pretend play skills to children with Autism Spectrum Disorder. *Research in Autism Spectrum Disorders*, *5*(2), 872–884. https://doi.org/10.1016/j.rasd.2010.10.002

Lydon, H., Healy, O., Leader, G., & Keohane, D.-D. (2009). The effects of intensive tact instruction on three verbal operants in non-instructional settings for two children with autism. *The Journal of Speech and Language Pathology – Applied Behavior Analysis*, *3*(2–3), 173–184. https://doi.org/10.1037/h0100242

Lyons, E. A., Rue, H. C., Luiselli, J. K., & DiGennaro, F. D. (2007). Brief functional analysis and supplemental feeding for postmeal rumination in children with developmental disabilities. *Journal of Applied Behavior Analysis*, *40*(4), 743–747. https://doi.org/10.1901/jaba.2007.743-747

MacDonald, R., Parry-Cruwys, D., Dupere, S., & Ahearn, W. (2014). Assessing progress and outcome of early intensive behavioral intervention for toddlers with autism. *Research in Developmental Disabilities*, *35*(12), 3632–3644. https://doi.org/10.1016/j.ridd.2014.08.036

MacDonald, R., Sacramone, S., Mansfield, R., Wiltz, K., & Ahearn, W. H. (2009). Using video modeling to teach reciprocal pretend play to children with autism. *Journal of Applied Behavior Analysis*, *42*(1), 43–55. https://doi.org/10.1901/jaba.2009.42-43

MacDuff, J. L., Ledo, R., McClannahan, L. E., & Krantz, P. J. (2007). Using scripts and script-fading procedures to promote bids for joint attention by young children with autism. *Research in Autism Spectrum Disorders*, *1*(4), 281–290. https://doi.org/10.1016/j.rasd.2006.11.003

Mace, A. B., Shapiro, E. S., & Mace, F. C. (1998). Effects of warning stimuli for reinforcer withdrawal and task onset on self-injury. *Journal of Applied Behavior Analysis*, *31*(4), 679–682. https://doi.org/10.1901/jaba.1998.31-679

Mace, F. C., Pratt, J. L., Prager, K. L., & Pritchard, D. (2011). An evaluation of three methods of saying “no” to avoid an escalating response class hierarchy. *Journal of Applied Behavior Analysis*, *44*(1), 83–94. https://doi.org/10.1901/jaba.2011.44-83

Magee, S. K., & Ellis, J. (2001). The detrimental effects of physical restraint as a consequence for inappropriate classroom behavior. *Journal of Applied Behavior Analysis*, *34*(4), 501–504. https://doi.org/10.1901/jaba.2001.34-501

Magiati, I., Charman, T., & Howlin, P. (2007). A two-year prospective follow-up study of community-based early intensive behavioural intervention and specialist nursery provision for children with autism spectrum disorders. *Journal of Child Psychology and Psychiatry*, *48*(8), 803–812. https://doi.org/10.1111/j.1469-7610.2007.01756.x

Magiati, I., Moss, J., Charman, T., & Howlin, P. (2011). Patterns of change in children with Autism Spectrum Disorders who received community based comprehensive interventions in their pre-school years: A seven year follow-up study. *Research in Autism Spectrum Disorders*, *5*(3), 1016–1027. https://doi.org/10.1016/j.rasd.2010.11.007

Maione, L., & Mirenda, P. (2006). Effects of Video Modeling and Video Feedback on Peer-Directed Social Language Skills of a Child With Autism. *Journal of Positive Behavior Interventions*, *8*(2), 106–118. https://doi.org/10.1177/10983007060080020201

Majdalany, L. M., Wilder, D. A., Greif, A., Mathisen, D., & Saini, V. (2014). Comparing massed‐trial instruction, distributed‐trial instruction, and task interspersal to teach tacts to children with autism spectrum disorders. *Journal of Applied Behavior Analysis*, *47*(3), 657–662. https://doi.org/10.1002/jaba.149

Majdalany, L., Wilder, D. A., Smeltz, L., & Lipschultz, J. (2016). The effect of brief delays to reinforcement on the acquisition of tacts in children with autism. *Journal of Applied Behavior Analysis*, *49*(2), 411–415. https://doi.org/10.1002/jaba.282

Mancil, G. R., Conroy, M. A., Nakao, T., & Alter, P. J. (2006). Functional communication training in the natural environment: A pilot investigation with a young child with autism spectrum disorder. *Education and Treatment of Children*, *29*(4), 615–633.

Mancil, G. R. (2008). Effects of a modified milieu therapy intervention on the social communicative behaviors of young children with autism spectrum disorders. [ProQuest Information & Learning]. In *Dissertation Abstracts International Section A: Humanities and Social Sciences* (Vol. 68, Issues 9-A).

Marchese, N. V, Carr, J. E., LeBlanc, L. A., Rosati, T. C., & Conroy, S. A. (2012). The effects of the question “What is this?” on tact-training outcomes of children with autism. *Journal of Applied Behavior Analysis*, *45*(3), 539–547. https://doi.org/10.1901/jaba.2012.45-539

Marckel, J. M., Neef, N. A., & Ferreri, S. J. (2006). A Preliminary Analysis of Teaching Improvisation With the Picture Exchange Communication System To Children With Autism. *Journal of Applied Behavior Analysis*, *39*(1), 109–115. https://doi.org/10.1901/jaba.2006.131-04

Marcus, A., & Wilder, D. A. (2009). A comparison of peer video modeling and self video modeling to teach textual responses in children with autism. *Journal of Applied Behavior Analysis*, *42*(2), 335–341. https://doi.org/10.1901/jaba.2009.42-335

Marion, C., Martin, G. L., Yu, C. T., Buhler, C., & Kerr, D. (2012). Teaching Children with Autism Spectrum Disorder to Mand “Where?” *Journal of Behavioral Education*, *21*(4), 273–294. https://doi.org/10.1007/s10864-012-9148-y

Marion, C., Martin, G. L., Yu, C. T., Buhler, C., Kerr, D., & Claeys, A. (2012). Teaching children with autism spectrum disorder to mand for information using “which?” *Journal of Applied Behavior Analysis*, *45*(4), 865–870. https://doi.org/10.1901/jaba.2012.45-865

Marzullo-Kerth, D., Reeve, S. A., Reeve, K. F., & Townsend, D. B. (2011). Using multiple-exemplar training to teach a generalized repertoire or sharing to children with autism. *Journal of Applied Behavior Analysis*, *44*(2), 279–294. https://doi.org/10.1901/jaba.2011.44-279

Matson, J. L., Sevin, J. A., Box, M. L., Francis, K. L., & Sevin, B. M. (1993). An evaluation of two methods for increasing self-initiated verbalizations in autistic children. *Journal of Applied Behavior Analysis*, *26*(3), 389–398. https://doi.org/10.1901/jaba.1993.26-389

Matson, J. L., Sevin, J. A., Fridley, D., & Love, S. R. (1990). Increasing spontaneous language in three autistic children. *Journal of Applied Behavior Analysis*, *23*(2), 227–233. https://doi.org/10.1901/jaba.1990.23-227

Matsushita, H., & Sonoyama, S. (2010). Teaching ball-throwing skills to a boy with Asperger’s disorder: A case study. *Japanese Journal of Special Education*, *47*(6), 495–508. https://doi.org/10.6033/tokkyou.47.495

Matsuzaki, A., & Yamamoto, J. (2012). Effects of an early intervention program on preverbal communication in a child with autism: Developmental and behavioral analysis with a multiple-baseline design. *Japanese Journal of Special Education*, *49*(6), 657–669. https://doi.org/10.6033/tokkyou.49.657

Mazza, M., Pino, M. C., Vagnetti, R., Filocamo, A., Attanasio, M., Calvarese, A., & Valenti, M. (2021). Intensive intervention for adolescents with autism spectrum disorder: comparison of  three rehabilitation treatments. *International Journal of Psychiatry in Clinical Practice*, *25*(1), 28–36. https://doi.org/10.1080/13651501.2020.1800042

McComas, J. J., Thompson, A., & Johnson, L. (2003). The effects of presession attention on problem behavior maintained by different reinforcers. *Journal of Applied Behavior Analysis*, *36*(3), 297–307. https://doi.org/10.1901/jaba.2003.36-297

McComas, J., Hoch, H., Paone, D., & El-Roy, D. (2000). Escape behavior during academic tasks: A preliminary analysis of idiosyncratic establishing operations. *Journal of Applied Behavior Analysis*, *33*(4), 479–493. https://doi.org/10.1901/jaba.2000.33-479

McDonald, J., Moore, D. W., & Anderson, A. (2012). Comparison of functional assessment methods targeting aggressive and stereotypic behaviour in a child with autism. *The Australian Educational and Developmental Psychologist*, *29*(1), 52–65. https://doi.org/10.1017/edp.2012.9

McDonald, M. E., & Hemmes, N. S. (2003). Increases in social initiation toward an adolescent with autism: Reciprocity effects. *Research in Developmental Disabilities*, *24*(6), 453–465. https://doi.org/10.1016/j.ridd.2003.04.001

McGarrell, M., Healy, O., Leader, G., O’Connor, J., & Kenny, N. (2009). Six reports of children with autism spectrum disorder following intensive behavioral intervention using the Preschool Inventory of Repertoires for Kindergarten (PIRK®). *Research in Autism Spectrum Disorders*, *3*(3), 767–782. https://doi.org/10.1016/j.rasd.2009.02.006

McGee, G. G., Almeida, M. C., Sulzer-Azaroff, B., & Feldman, R. S. (1992). Promoting reciprocal interactions via peer incidental teaching. *Journal of Applied Behavior Analysis*, *25*(1), 117–126. https://doi.org/10.1901/jaba.1992.25-117

Meier, A. E., Fryling, M. J., & Wallace, M. D. (2012). Using high-probability foods to increase the acceptance of low-probability foods. *Journal of Applied Behavior Analysis*, *45*(1), 149–153. https://doi.org/10.1901/jaba.2012.45-149

Mello, C., Rivard, M., Terroux, A., & Mercier, C. (2018). Differential responses to early behavioural intervention in young children with autism spectrum disorders as a function of features of intellectual disability. *Journal on Developmental Disabilities*, *23*(3), 5–17.

Miguel, C. F., & Kobari-Wright, V. V. (2013). The effects of tact training on the emergence of categorization and listener behavior in children with autism. *Journal of Applied Behavior Analysis*, *46*(3), 669–673. https://doi.org/10.1002/jaba.62

Miguel, C. F., Yang, H. G., Finn, H. E., & Ahearn, W. H. (2009). Establishing derived textual control in activity schedules with children with autism. *Journal of Applied Behavior Analysis*, *42*(3), 703–709. https://doi.org/10.1901/jaba.2009.42-703

Miliotis, A., Sidener, T. M., Reeve, K. F., Carbone, V., Sidener, D. W., Rader, L., & Delmolino, L. (2012). An evaluation of the number of presentations of target sounds during stimulus-stimulus pairing trials. *Journal of Applied Behavior Analysis*, *45*(4), 809–813. https://doi.org/10.9101/jaba.2012.45-809

Miller, N., & Neuringer, A. (2000). Reinforcing variability in adolescents with autism. *Journal of Applied Behavior Analysis*, *33*(2), 151–165. https://doi.org/10.1901/jaba.2000.33-151

Miller, S. A., Rodriguez, N. M., & Rourke, A. J. (2015). Do mirrors facilitate acquisition of motor imitation in children diagnosed with autism? *Journal of Applied Behavior Analysis*, *48*(1), 194–198. https://doi.org/10.1002/jaba.187

Ming, S., Mulhern, T., Stewart, I., Moran, L., & Bynum, K. (2018). Training class inclusion responding in typically developing children and individuals with autism. *Journal of Applied Behavior Analysis*, *51*(1), 53–60. https://doi.org/10.1002/jaba.429

Mithaug, D. K., & Mithaug, D. E. (2003). Effects of teacher-directed versus student-directed instruction on self-management of young children with disabilities. *Journal of Applied Behavior Analysis*, *36*(1), 133–136. https://doi.org/10.1901/jaba.2003.36-133

Mitteer, D. R., Romani, P. W., Greer, B. D., & Fisher, W. W. (2015). Assessment and treatment of pica and destruction of holiday decorations. *Journal of Applied Behavior Analysis*, *48*(4), 912–917. https://doi.org/10.1002/jaba.255

Moes, D. R., & Frea, W. D. (2002). Contextualized Behavioral Support in Early Intervention for Children with Autism and Their Families. *Journal of Autism and Developmental Disorders*, *32*(6), 519–533. https://doi.org/10.1023/A:1021298729297

Mohammadzaheri, F., Koegel, L. K., Rezaee, M., & Rafiee, S. M. (2014). A randomized clinical trial comparison between pivotal response treatment (PRT) and structured applied behavior analysis (ABA) intervention for children with autism. *Journal Of Autism And Developmental Disorders*, *44*, 2769–2777. https://doi.org/10.1007/s10803-014-2137-3

Mohammadzaheri, F., Koegel, L. K., Rezaei, M., & Bakhshi, E. (2015). A Randomized Clinical Trial Comparison Between Pivotal Response Treatment (PRT) and Adult-Driven Applied Behavior Analysis (ABA) Intervention on Disruptive Behaviors in Public School Children with Autism. *Journal of Autism and Developmental Disorders*, *45*(9), 2899–2907. https://doi.org/10.1007/s10803-015-2451-4

Morrison, L., Kamps, D., Garcia, J., & Parker, D. (2001). Peer Mediation and Monitoring Strategies to Improve Initiations and Social Skills for Students with Autism. *Journal of Positive Behavior Interventions*, *3*(4), 237–250. https://doi.org/10.1177/109830070100300405

Morrison, R. S., Sainato, D. M., Benchaaban, D., & Endo, S. (2002). Increasing play skills of children with autism using activity schedules and correspondence training. *Journal of Early Intervention*, *25*(1), 58–72. https://doi.org/10.1177/105381510202500106

Muething, C. S., Falcomata, T. S., Ferguson, R., Swinnea, S., & Shpall, C. (2018). An evaluation of delay to reinforcement and mand variability during functional communication training. *Journal of Applied Behavior Analysis*, *51*(2), 263–275. https://doi.org/10.1002/jaba.441

Murphy, C., & Barnes-Holmes, D. (2010). Establishing complex derived manding with children with and without a diagnosis of autism. *The Psychological Record*, *60*(3), 489–504. https://doi.org/10.1007/bf03395723

Murphy, C., & Barnes-Holmes, D. (2010). Establishing five derived mands in three adolescent boys with autism. *Journal of Applied Behavior Analysis*, *43*(3), 537–541. https://doi.org/10.1901/jaba.2010.43-537

Murphy, C., & Barnes-Holmes, D. (2009). Derived more-less relational mands in children diagnosed with autism. *Journal of Applied Behavior Analysis*, *42*(2), 253–268. https://doi.org/10.1901/jaba.2009.42-253

Murphy, C., Barnes-Holmes, D., & Barnes-Holmes, Y. (2005). Derived manding in children with autism: Synthesizing Skinner’s verbal behavior with relational frame theory. *Journal of Applied Behavior Analysis*, *38*(4), 445–462. https://doi.org/10.1901/jaba.2005.97-04

Murphy, C., Lyons, K., Kelly, M., Barnes-Holmes, Y., & Barnes-Holmes, D. (2019). Using the Teacher IRAP (T-IRAP) interactive computerized programme to teach complex flexible relational responding with children with diagnosed autism spectrum disorder. *Behavior Analysis in Practice*, *12*(1), 52–65. https://doi.org/10.1007/s40617-018-00302-9

Najdowski, A. C., St. Clair, M., Fullen, J. A., Child, A., Persicke, A., & Tarbox, J. (2018). Teaching children with autism to identify and respond appropriately to the preferences of others during play. *Journal of Applied Behavior Analysis*, *51*(4), 890–898. https://doi.org/10.1002/jaba.494

Najdowski, A. C., Wallace, M. D., Doney, J. K., & Ghezzi, P. M. (2003). Parental assessment and treatments of food selectivity in natural settings. *Journal of Applied Behavior Analysis*, *36*(3), 383–386. https://doi.org/10.1901/jaba.2003.36-383

Najdowski, A. C., Wallace, M. D., Penrod, B., Tarbox, J., Reagon, K., & Higbee, T. S. (2008). Caregiver-conducted experimental functional analyses of inappropriate mealtime behavior. *Journal of Applied Behavior Analysis*, *41*(3), 459–465. https://doi.org/10.1901/jaba.2008.41-459

Najdowski, A. C., Wallace, M. D., Reagon, K., Penrod, B., Higbee, T. S., & Tarbox, J. (2010). Utilizing a home-based parent training approach in the treatment of food selectivity. *Behavioral Interventions*, *25*(2), 89–107. https://doi.org/10.1002/bin.298

Napolitano, D. A., Blakkman, L. A., Kohl, L. B., Vallese, H. M., & McAdam, D. B. (2007). The use of functional communication training to reduce pica. *The Journal of Speech and Language Pathology – Applied Behavior Analysis*, *2*(1), 25–31. https://doi.org/10.1037/h0100206

Napolitano, D. A., Smith, T., Zarcone, J. R., Goodkin, K., & McAdam, D. B. (2010). Increasing response diversity in children with autism. *Journal of Applied Behavior Analysis*, *43*(2), 265–271. https://doi.org/10.1901/jaba.2010.43-265

Nasr, M. W. (2016). Restricted and repetitive behaviors as strengths, not weaknesses: Evaluating the use of social stories that embed restricted interests on the social skills of children with autism spectrum disorder. [ProQuest Information & Learning]. In *Dissertation Abstracts International Section A: Humanities and Social Sciences* (Vol. 77, Issues 5-A(E)).

Neely, L., Graber, J., Kunnavatana, S., & Cantrell, K. (2020). Impact of language on behavior treatment outcomes. *Journal of Applied Behavior Analysis*, *53*(2), 796–810. https://doi.org/10.1002/jaba.626

Neely, L., Hong, E. R., Kawamini, S., Umana, I., & Kurz, I. (2020). Intercontinental telehealth to train Japanese interventionists in incidental teaching for children with autism. *Journal of Behavioral Education*, *29*(2), 433–448. https://doi.org/10.1007/s10864-020-09377-3

Neely, L., Rispoli, M., Boles, M., Morin, K., Gregori, E., Ninci, J., & Hagan-Burke, S. (2019). Interventionist acquisition of incidental teaching using pyramidal training via telehealth. *Behavior Modification*, *43*(5), 711–733. https://doi.org/10.1177/0145445518781770

Neil, N., Hansford, R., Young, K., & Zwick, L. (2020). The effect of instructional pacing on skill acquisition and maintenance for children with developmental disabilities. *Journal on Developmental Disabilities*, *25*(1), 1–15.

Nelson, C., McDonnell, A. P., Johnston, S. S., Crompton, A., & Nelson, A. R. (2007). Keys to play: A strategy to increase the social interactions of young children with autism and their typically developing peers. *Education and Training in Developmental Disabilities*, *42*(2), 165–181.

Nelson, D. L., Gergenti, E., & Hollander, A. C. (1980). Extra prompts versus no extra prompts in self-care training of autistic children and adolescents. *Journal of Autism and Developmental Disorders*, *10*(3), 311–321. https://doi.org/10.1007/BF02408290

Newman, B., Buffington, D. M., & Hemmes, N. S. (1996). Self-Reinforcement Used to Increase the Appropriate Conversation of Autistic Teenagers. *Education and Training in Mental Retardation and Developmental Disabilities*, *31*(4), 304–309.

Newman, B., Buffington, D. M., O’grady, M. A., Mcdonald, M. E., Poulson, C. L., & Hemmes, N. S. (1995). Self-Management of Schedule following in Three Teenagers with Autism. *Behavioral Disorders*, *20*(3), 190–196. https://doi.org/10.1177/019874299502000304

Newman, B., Reinecke, D. R., & Meinberg, D. L. (2000). Self-management of varied responding in three students with autism. *Behavioral Interventions*, *15*(2), 145–151. https://doi.org/10.1002/(SICI)1099-078X(200004/06)15:2<145::AID-BIN50>3.0.CO;2-3

Newman, B., Tuntigian, L., Ryan, C. S., & Reinecke, D. R. (1997). Self-management of a DRO procedure by three students with autism. *Behavioral Interventions*, *12*(3), 149–156. https://doi.org/10.1002/(SICI)1099-078X(199707)12:3<149::AID-BRT173>3.0.CO;2-M

Nikopoulous, C. K., & Keenan, M. (2004). Effects of video modeling on social initiations by children with autism. *Journal of Applied Behavior Analysis*, *37*(1), 93–96. https://doi.org/10.1901/jaba.2004.37-93

Noel, C. R., & Getch, Y. Q. (2016). Noncontingent reinforcement in after-school settings to decrease classroom disruptive behavior for students with autism spectrum disorder. *Behavior Analysis in Practice*, *9*, 261–265. https://doi.org/10.1007/s40617-016-0117-0

Norman, J. M., Collins, B. C., & Schuster, J. W. (2001). Using an Instructional Package Including Video Technology to Teach Self-Help Skills to Elementary Students with Mental Disabilities. *Journal of Special Education Technology*, *16*(3), 5–18. https://doi.org/10.1177/016264340101600301

Normand, M. P., & Beaulieu, L. (2011). Further evaluation of response-independent delivery of preferred stimuli and child compliance. *Journal of Applied Behavior Analysis*, *44*(3), 665–669. https://doi.org/10.1901/jaba.2011.44-665

Northgrave, J., Vladescu, J. C., DeBar, R. M., Toussaint, K. A., & Schnell, L. K. (2019). Reinforcer choice on skill acquisition for children with autism spectrum disorder: A systematic replication. *Behavior Analysis in Practice*, *12*(2), 401–406. https://doi.org/10.1007/s40617-018-0246-8

Nottingham, C. L., Vladescu, J. C., DeBar, R. M., Deshais, M., & DeQuinzio, J. (2020). The influence of instructive feedback presentation schedule: A replication with children with autism spectrum disorder. *Journal of Applied Behavior Analysis*, *53*(4), 2287–2302. https://doi.org/10.1002/jaba.706

Nottingham, C. L., Vladescu, J. C., Kodak, T., & Kisamore, A. N. (2017). Incorporating multiple secondary targets into learning trials for individuals with autism spectrum disorder. *Journal of Applied Behavior Analysis*, *50*(3), 653–661. https://doi.org/10.1002/jaba.396

Novack, M. N., Hong, E., Dixon, D. R., & Granpeesheh, D. (2019). An evaluation of a mobile application designed to teach receptive language skills to children with autism spectrum disorder. *Behavior Analysis in Practice*, *12*(1), 66–77. https://doi.org/10.1007/s40617-018-00312-7

Nuzzolo-Gomez, R., Leonard, M. A., Ortiz, E., Rivera, C. M., & Greer, R. D. (2002). Teaching Children with Autism to Prefer Books or Toys Over Stereotypy or Passivity. *Journal of Positive Behavior Interventions*, *4*(2), 80–87. https://doi.org/10.1177/109830070200400203

O’Connor, J., Barnes-Holmes, Y., & Barnes-Holmes, D. (2011). Establishing contextual control over symmetry and asymmetry performances in typically developing children and children with autism. *The Psychological Record*, *61*(2), 287–312. https://doi.org/10.1007/BF03395761

Odluyurt, S., Tekin-Iftar, E., & Adalioglu, I. (2012). Does treatment integrity matter in promoting learning among children with developmental disabilities? *Topics in Early Childhood Special Education*, *32*(3), 143–150. https://doi.org/10.1177/0271121410394208

Ogletree, B. T., Fischer, M. A., & Sprous, J. (1995). An Innovative Language Treatment for a Child with High-Functioning Autism. *Focus on Autistic Behavior*, *10*(3), 1–9. https://doi.org/10.1177/108835769501000301

Olsson, M. B., Westerlund, J., Lundström, S., Giacobini, M., Fernell, E., & Gillberg, C. (2015). “Recovery” from the diagnosis of autism—And then? *Neuropsychiatric Disease and Treatment*, *11*, 999–1005. https://doi.org/https://doi.org/10.2147/NDT.S78707

Openden, D. A. (2005). Pivotal response treatment for multiple families of children with autism: Probable efficacy and effectiveness of a group parent education workshop [University of California, Santa Barbara]. In *ProQuest Dissertations and Theses*.

O’Reilly, M., Aguilar, J., Fragale, C., Lang, R., Edrisinha, C., Sigafoos, J., Lancioni, G., & Didden, R. (2012). Effects of a motivating operation manipulation on the maintenance of mands. *Journal of Applied Behavior Analysis*, *45*(2), 443–447. https://doi.org/10.1901/jaba.2012.45-443

Paden, A. R., Kodak, T., Fisher, W. W., Gawley-Bullington, E. M., & Bouxsein, K. J. (2012). Teaching children with autism to engage in peer-directed mands using a picture exchange communication system. *Journal of Applied Behavior Analysis*, *45*(2), 425–429. https://doi.org/10.1901/jaba.2012.45-425

Pane, H. M., Sidener, T. M., Vladescu, J. C., & Nirgudkar, A. (2015). Evaluating function-based social stories^TM^ with children with autism. *Behavior Modification*, *39*(6), 912–931. https://doi.org/10.1177/0145445515603708

Park, H. L., Du, L., & Choi, J. (2020). Education for children with ASD in South Korea: A case study. *Behavior Analysis in Practice*, *13*(4), 838–848. https://doi.org/10.1007/s40617-020-00453-8

Parry-Cruwys, D. E., Neal, C. M., Ahearn, W. H., Wheeler, E. E., Premchander, R., Loeb, M. B., & Dube, W. V. (2011). Resistance to disruption in a classroom setting. *Journal of Applied Behavior Analysis*, *44*(2), 363–367. https://doi.org/10.1901/jaba.2011.44-363

Partington, J. W., Sundberg, M. L., Newhouse, L., & Spengler, S. M. (1994). Overcoming an autistic child’s failure to acquire a tact repertoire. *Journal of Applied Behavior Analysis*, *27*(4), 733–734. https://doi.org/10.1901/jaba.1994.27-733

Passage, M., Tincani, M., & Hantula, D. A. (2012). Teaching self-control with qualitatively different reinforcers. *Journal of Applied Behavior Analysis*, *45*(4), 853–857. https://doi.org/10.1901/jaba.2012.45-853

Patel, M. R., Piazza, C. C., Kelly, M. L., Ochsner, C. A., & Santana, C. M. (2001). Using a fading procedure to increase fluid consumption in a child with feeding problems. *Journal of Applied Behavior Analysis*, *34*(3), 357–360. https://doi.org/10.1901/jaba.2001.34-357

Pechous, E. A. (2001). Young children with autism and intensive behavioral programs: Effects on the primary attachment relationship. In *Dissertation Abstracts International: Section B: The Sciences and Engineering* (Vol. 61, Issues 11-B). California School of Professional Psychology, Fresno.

Peisley, M., Foster, T. M., & Sargisson, R. J. (2020). Reinforcing the prospective remembering of children with autism spectrum disorder. *Journal of Applied Behavior Analysis*, *53*(1), 121–133. https://doi.org/10.1002/jaba.546

Penrod, B., Gardella, L., & Fernand, J. (2012). An evaluation of a progressive high-probability instructional sequence combined with low-probability demand fading in the treatment of food selectivity. *Journal of Applied Behavior Analysis*, *45*(3), 527–537. https://doi.org/10.1901/jaba.2012.45-527

Penrod, B., Wallace, M. D., & Dyer, E. J. (2008). Assessing potency of high- and low-preference reinforcers with respect to response rate and response patterns. *Journal of Applied Behavior Analysis*, *41*(2), 177–188. https://doi.org/10.1901/jaba.2008.41-177

Perez, B. C., Bacotti, J. K., Peters, K. P., & Vollmer, T. R. (2020). An extension of commonly used toilet‐training procedures to children with autism spectrum disorder. *Journal of Applied Behavior Analysis*, *53*(4), 2360–2375. https://doi.org/10.1002/jaba.727

Pérez-González, L. A., García-Asenjo, L., Williams, G., & Carnerero, J. J. (2007). Emergence of Intraverbal Antonyms in Children with Pervasive Developmental Disorder. *Journal of Applied Behavior Analysis*, *40*(4), 697–701. https://doi.org/10.1901/jaba.2007.697–701

Perrin, C. J., & Neef, N. A. (2012). Further analysis of variables that affect self-control with aversive events. *Journal of Applied Behavior Analysis*, *45*(2), 299–313. https://doi.org/10.1901/jaba.2012.45-299

Perry, A., Cummings, A., Dunn Geier, J., Freeman, N. L., Hughes, S., LaRose, L., Managhan, T., Reitzel, J.-A., & Williams, J. (2008). Effectiveness of Intensive Behavioral Intervention in a large, community-based program. *Research in Autism Spectrum Disorders*, *2*(4), 621–642. https://doi.org/10.1016/j.rasd.2008.01.002

Peters, L. C., & Thompson, R. H. (2015). Teaching children with autism to respond to conversation partners’ interest. *Journal of Applied Behavior Analysis*, *48*(3), 544–562. https://doi.org/10.1002/jaba.235

Peterson, C., Lerman, D. C., & Nissen, M. A. (2016). Reinforcer choice as an antecedent versus consequence. *Journal of Applied Behavior Analysis*, *49*(2), 286–293. https://doi.org/10.1002/jaba.284

Peterson, K. M., Piazza, C. C., Ibañez, V. F., & Fisher, W. W. (2019). Randomized controlled trial of an applied behavior analytic intervention for food selectivity in children with autism spectrum disorder. *Journal of Applied Behavior Analysis*, *52*(4), 895–917. https://doi.org/10.1002/jaba.650

Peterson, K. M., Piazza, C. C., & Volkert, V. M. (2016). A comparison of a modified sequential oral sensory approach to an applied behavior-analytic approach in the treatment of food selectivity in children with autism spectrum disorder. *Journal of Applied Behavior Analysis*, *49*(3), 485–511. https://doi.org/10.1002/jaba.332

Peterson, S. P., Rodriguez, N. M., & Pawich, T. L. (2019). Effects of modeling rote versus varied responses on response variability and skill acquisition during discrete‐trial instruction. *Journal of Applied Behavior Analysis*, *52*(2), 370–385. https://doi.org/10.1002/jaba.528

Peters-Scheffer, N., Didden, R., Mulders, M., & Korzilius, H. (2010). Low intensity behavioral treatment supplementing preschool services for young children with autism spectrum disorders and severe to mild intellectual disability. *Research in Developmental Disabilities*, *31*(6), 1678–1684. https://doi.org/10.1016/j.ridd.2010.04.008

Peters-Scheffer, N., Didden, R., Mulders, M., & Korzilius, H. (2013). Effectiveness of low intensity behavioral treatment for children with autism spectrum disorder and intellectual disability. *Research in Autism Spectrum Disorders*, *7*(9), 1012–1025. https://doi.org/10.1016/j.rasd.2013.05.001

Petursdottir, A.-L., McComas, J., McMaster, K., & Horner, K. (2007). The effects of scripted peer tutoring and programming common stimuli on social interactions of a student with autism spectrum disorder. *Journal of Applied Behavior Analysis*, *40*(2), 353–357. https://doi.org/10.1901/jaba.2007.160-05

Peyton, R. T., Lindauer, S. E., & Richman, D. M. (2005). The Effects of Directive and Nondirective Prompts on Noncompliant Vocal Behavior Exhibited by a Child with Autism. *Journal of Applied Behavior Analysis*, *38*(2), 251–255. https://doi.org/10.1901/jaba.2005.125-04

Phillips, C. L., & Vollmer, T. R. (2012). Generalized instruction following with pictorial prompts. *Journal of Applied Behavior Analysis*, *45*(1), 37–54. https://doi.org/10.1901/jaba.2012.45-37

Phillips, C. L., Vollmer, T. R., & Porter, A. (2019). An evaluation of textual prompts and generalized textual instruction‐following. *Journal of Applied Behavior Analysis*, *52*(4), 1140–1160. https://doi.org/https://doi.org/10.1002/jaba.649

Piazza, C. C., Patel, M. R., Santana, C. M., Goh, H.-L., Delia, M. D., & Lancaster, B. M. (2002). An evaluation of simultaneous and sequential presentation of preferred and nonpreferred food to treat food selectivity. *Journal of Applied Behavior Analysis*, *35*(3), 259–270. https://doi.org/10.1901/jaba.2002.35-259

Pierce, K. L., & Schreibman, L. (1994). Teaching daily living skills to children with autism in unsupervised settings through pictorial self-management. *Journal of Applied Behavior Analysis*, *27*(3), 471–481. https://doi.org/10.1901/jaba.1994.27-471

Pierce, K., & Schreibman, L. (1995). Increasing complex social behaviors in children with autism: Effects of peer-implemented pivotal response training. *Journal of Applied Behavior Analysis*, *28*(3), 285–295. https://doi.org/10.1901/jaba.1995.28-285

Pierce, K., & Schreibman, L. (1997). Multiple Peer Use of Pivotal Response Training To Increase Social Behaviors of Classmates with Autism: Results from Trained and Untrained Peers. *Journal of Applied Behavior Analysis*, *30*(1), 157–160. https://doi.org/10.1901/jaba.1997.30-157

Piper, A., Borrero, J. C., & Becraft, J. L. (2020). Differential reinforcement‐of‐low‐rate procedures: A systematic replication with students with autism spectrum disorder. *Journal of Applied Behavior Analysis*, *53*(2), 1058–1070. https://doi.org/10.1002/jaba.631

Pisman, M. D., & Luczynski, K. C. (2020). Caregivers can implement play‐based instruction without disrupting child preference. *Journal of Applied Behavior Analysis*, *53*(3), 1702–1725. https://doi.org/10.1002/jaba.705

Pitts, L., Gent, S., & Hoerger, M. L. (2019). Reducing pupils’ barriers to learning in a special needs school: Integrating applied behaviour analysis into key stages 1–3. *British Journal of Special Education*, *46*(1), 94–112. https://doi.org/10.1111/1467-8578.12251

Plaisance, L., Lerman, D. C., Laudont, C., & Wu, W. (2016). Inserting mastered targets during error correction when teaching skills to children with autism. *Journal of Applied Behavior Analysis*, *49*(2), 251–264. https://doi.org/10.1002/jaba.292

Planer, J., DeBar, R., Progar, P., Reeve, K., & Sarokoff, R. (2018). Evaluating tasks within a high‐probability request sequence in children with autism spectrum disorder. *Behavioral Interventions*, *33*(4), 380–390. https://doi.org/10.1002/bin.1634

Plavnick, J. B., & Ferreri, S. J. (2011). Establishing verbal repertoires in children with autism using function-based video modeling. *Journal of Applied Behavior Analysis*, *44*(4), 747–766. https://doi.org/10.1901/jaba.2011.44-747

Plavnick, J. B., Ferreri, S. J., & Maupin, A. N. (2010). The effects of self-monitoring on the procedural integrity of a behavioral intervention for young children with developmental disabilities. *Journal of Applied Behavior Analysis*, *43*(2), 315–320. https://doi.org/10.1901/jaba.2010.43-315

Polick, A. S., Carr, J. E., & Hanney, N. M. (2012). A comparison of general and descriptive praise in teaching intraverbal behavior to children with autism. *Journal of Applied Behavior Analysis*, *45*(3), 593–599. https://doi.org/10.1901/jaba.2012.45-593

Pollard, J. S., Betz, A. M., & Higbee, T. S. (2012). Script fading to promote unscripted bids for joint attention in children with autism. *Journal of Applied Behavior Analysis*, *45*(2), 387–393. https://doi.org/10.1901/jaba.2012.45-387

Polychronis, S. C., Mcdonnell, J., Johnson, J. W., Riesen, T., & Jameson, M. (2004). A Comparison of Two Trial Distribution Schedules in Embedded Instruction. *Focus on Autism and Other Developmental Disabilities*, *19*(3), 140–151. https://doi.org/10.1177/10883576040190030201

Protopopova, A., Matter, A. L., Harris, B. N., Wiskow, K. M., & Donaldson, J. M. (2020). Comparison of contingent and noncontingent access to therapy dogs during academic tasks in children with autism spectrum disorder. *Journal of Applied Behavior Analysis*, *53*(2), 811–834. https://doi.org/10.1002/jaba.619

Quigley, J., Griffith, A. K., & Kates-McElrath, K. (2018). A comparison of modeling, prompting, and a multi-component intervention for teaching play skills to children with developmental disabilities. *Behavior Analysis in Practice*, *11*(4), 315–326. https://doi.org/10.1007/s40617-018-0225-0

Raaymakers, C. (2019). The role of causal relational frames and “Why” question answering [ProQuest Information & Learning]. In *Dissertation Abstracts International: Section B: The Sciences and Engineering* (Vol. 80, Issues 1-B(E)).

Rad, F., Mihailescu, I., Nedelcu, M. C., Buica, A., Stancu, M., Andrei, E., Irimie, A., Anghel, C., & Dobrescu, I. (2019). The outcome of a sample of pre-schoolers diagnosed with ASD comorbid with ADHD after one year of Applied Behavioural Analysis. *Journal of Evidence-Based Psychotherapies*, *19*(2), 109–118. https://doi.org/10.24193/jebp.2019.2.16

Rader, L., Sidener, T. M., Reeve, K. F., Sidener, D. W., Delmolino, L., Miliotis, A., & Carbone, V. (2014). Stimulus-stimulus pairing of vocalizations: A systematic replication. *Analysis of Verbal Behavior*, *30*(1), 69–74. https://doi.org/10.1007/s40616-014-0012-0

Radley, K. C., Dart, E. H., Moore, J. W., Battaglia, A. A., & LaBrot, Z. C. (2017). Promoting accurate variability of social skills in children with autism spectrum disorder. *Behavior Modification*, *41*(1), 84–112. https://doi.org/10.1177/0145445516655428

Rapp, J. T. (2007). Further evaluation of methods to identify matched stimulation. *Journal of Applied Behavior Analysis*, *40*(1), 73–88. https://doi.org/10.1901/jaba.2007.142-05

Rapp, J. T., Vollmer, T. R., St. Peter, C., Dozier, C. L., & Cotnoir, N. M. (2004). Analysis of response allocation in individuals with multiple forms of stereotyped behavior. *Journal of Applied Behavior Analysis*, *37*(4), 481–501. https://doi.org/10.1901/jaba.2004.37-481

Reagon, K. A., & Higbee, T. S. (2009). Parent-implemented script fading to promote play-based verbal initiations in children with autism. *Journal of Applied Behavior Analysis*, *42*(3), 659–664. https://doi.org/10.1901/jaba.2009.42-659

Reed, P., & Osborne, L. (2012). Impact of severity of autism and intervention time‐input on child outcomes: Comparison across several early interventions. *British Journal of Special Education*, *39*(3), 130–136. https://doi.org/10.1111/j.1467-8578.2012.00549.x

Reed, P., Osborne, L. A., & Corness, M. (2007). The Real-World Effectiveness of Early Teaching Interventions for Children with Autism Spectrum Disorder. *Exceptional Children*, *73*(4), 417–433. https://doi.org/10.1177/001440290707300402

Reed, P., Osborne, L. A., & Corness, M. (2007). Brief report: Relative effectiveness of different home-based behavioral approaches to early teaching intervention. *Journal of Autism and Developmental Disorders*, *37*(9), 1815–1821. https://doi.org/10.1007/s10803-006-0306-8

Reeve, C. E., & Carr, E. G. (2000). Prevention of Severe Behavior Problems in Children with Developmental Disorders. *Journal of Positive Behavior Interventions*, *2*(3), 144–160. https://doi.org/10.1177/109830070000200303

Reeve, S. A., Reeve, K. F., Townsend, D. B., & Poulson, C. L. (2007). Establishing a generalized repertoire of helping behavior in children with autism. *Journal of Applied Behavior Analysis*, *40*(1), 123–136. https://doi.org/10.1901/jaba.2007.11-05

Reichow, B., & Wolery, M. (2011). Comparison of progressive prompt delay with and without instructive feedback. *Journal of Applied Behavior Analysis*, *44*(2), 327–340. https://doi.org/10.1901/jaba.2011.44-327

Reinecke, D. R., Newman, B., & Meinberg, D. L. (1999). Self-management of sharing in three pre-schoolers with autism. *Education and Training in Mental Retardation and Developmental Disabilities*, *34*(3), 312–317.

Reitzel, J., Summers, J., Lorv, B., Szatmari, P., Zwaigenbaum, L., Georgiades, S., & Duku, E. (2013). Pilot randomized controlled trial of a Functional Behavior Skills Training program for young children with Autism Spectrum Disorder who have significant early learning skill impairments and their families. *Research in Autism Spectrum Disorders*, *7*(11), 1418–1432. https://doi.org/10.1016/j.rasd.2013.07.025

Remington, B., Hastings, R. P., Kovshoff, H., degli Espinosa, F., Jahr, E., Brown, T., Alsford, P., Lemaic, M., & Ward, N. (2007). Early intensive behavioral intervention: outcomes for children with autism and their parents after two years. *American Journal of Mental Retardation*, *112*(6), 418–438. https://doi.org/10.1352/0895-8017(2007)112[418:EIBIOF]2.0.CO;2

Ribeiro, D. M., & Miguel, C. F. (2020). Using multiple‐tact training to produce emergent visual categorization in children with autism. *Journal of Applied Behavior Analysis*, *53*(3), 1768–1779. https://doi.org/10.1002/jaba.687

Richardson, A. R., Lerman, D. C., Nissen, M. A., Luck, K. M., Neal, A. E., Bao, S., & Tsami, L. (2017). Can pictures promote the acquisition of sight‐word reading? An evaluation of two potential instructional strategies. *Journal of Applied Behavior Analysis*, *50*(1), 67–86. https://doi.org/10.1002/jaba.354

Richling, S. M., Williams, W. L., & Carr, J. E. (2019). The effects of different mastery criteria on the skill maintenance of children with developmental disabilities. *Journal of Applied Behavior Analysis*, *52*(3), 701–717. https://doi.org/10.1002/jaba.580

Richman, D. M., Berg, W. K., Wacker, D. P., Stephens, T., Rankin, B., & Kilroy, J. (1997). Using Pretreatment and Posttreatment Assessments To Enhance and Evaluate Existing Treatment Packages. *Journal of Applied Behavior Analysis*, *30*(4), 709–712.

Richman, D. M., Lindauer, S. E., Crosland, K. A., McKerchar, T. L., & Morse, P. S. (2001). Functional analysis and treatment of breath holding maintained by nonsocial reinforcement. *Journal of Applied Behavior Analysis*, *34*(4), 531–534. https://doi.org/10.1901/jaba.2001.34-531

Ringdahl, J. E., Kitsukawa, K., Andelman, M. S., Call, N., Winborn, L., Barretto, A., & Reed, G. K. (2002). Differential reinforcement with and without instructional fading. *Journal of Applied Behavior Analysis*, *35*(3), 291–294. https://doi.org/10.1901/jaba.2002.35-291

Rispoli, M., Camargo, S., Machalicek, W., Lang, R., & Sigafoos, J. (2014). Functional communication training in the treatment of problem behavior maintained by access to rituals. *Journal of Applied Behavior Analysis*, *47*(3), 580–593. https://doi.org/10.1002/jaba.130

Rispoli, M., O’Reilly, M., Lang, R., Machalicek, W., Davis, T., Lancioni, G., & Sigafoos, J. (2011). Effects of motivating operations on problem and academic behavior in classrooms. *Journal of Applied Behavior Analysis*, *44*(1), 187–192. https://doi.org/10.1901/jaba.2011.44-187

Rivard, M., Terroux, A., & Mercier, C. (2014). Effectiveness of early behavioral intervention in public and mainstream settings: The case of preschool-age children with autism spectrum disorders. *Research in Autism Spectrum Disorders*, *8*(9), 1031–1043. https://doi.org/10.1016/j.rasd.2014.05.010

Riviere, V., Becquet, M., Peltret, E., Facon, B., & Darcheville, J.-C. (2011). Increasing compliance with medical examination requests directed to children with autism: Effects of a high-probability request procedure. *Journal of Applied Behavior Analysis*, *44*(1), 193–197. https://doi.org/10.1901/jaba.2011.44-193

Roane, H. R., Kelly, M. L., & Fisher, W. W. (2003). The effects of noncontingent access to food on the rate of object mouthing across three settings. *Journal of Applied Behavior Analysis*, *36*(4), 579–582. https://doi.org/10.1901/jaba.2003.36-579

Roane, H. S., Call, N. A., & Falcomata, T. S. (2005). A preliminary analysis of adaptive responding under open and closed economies. *Journal of Applied Behavior Analysis*, *38*(3), 335–348. https://doi.org/10.1901/jaba.2005.85-04

Roane, H. S., & Kelley, M. E. (2008). Decreasing problem behavior associated with a walking program for an individual with developmental and physical disabilities. *Journal of Applied Behavior Analysis*, *41*(3), 423–428. https://doi.org/10.1901/jaba.2008.41-423

Robinson, K.-A., & Smith, V. (2010). A specific vocational training program for an adolescent with autism. *Developmental Disabilities Bulletin*, *38*(1–2), 93–109.

Robison, M. A., Mann, T. B., & Ingvarsson, E. T. (2020). Life skills instruction for children with developmental disabilities. *Journal of Applied Behavior Analysis*, *53*(1), 431–448. https://doi.org/10.1002/jaba.602

Rodriguez, N. M., Thompson, R. H., Schlichenmeyer, K., & Stocco, C. S. (2012). Functional analysis and treatment of arranging and ordering by individuals with an autism spectrum disorder. *Journal of Applied Behavior Analysis*, *45*(1), 1–22. https://doi.org/10.1901/jaba.2012.45-1

Romaniuk, C., Miltenberger, R., Conyers, C., Jenner, N., Jurgens, M., & Ringenberg, C. (2002). The influence of activity choice on problem behaviors maintained by escape versus attention. *Journal of Applied Behavior Analysis*, *35*(4), 349–362. https://doi.org/10.1901/jaba.2002.35-349

Roncati, A. L., Souza, A. C., & Miguel, C. F. (2019). Exposure to a specific prompt topography predicts its relative efficiency when teaching intraverbal behavior to children with autism spectrum disorder. *Journal of Applied Behavior Analysis*, *52*(3), 739–745. https://doi.org/10.1002/jaba.568

Rosales, R., Maderitz, C., & Garcia, Y. A. (2014). Comparison of simple and complex auditory‐visual conditional discrimination training. *Journal of Applied Behavior Analysis*, *47*(2), 437–442. https://doi.org/10.1002/jaba.121

Rosales, R., Worsdell, A., & Trahan, M. (2010). Comparison of methods for varying item presentation during noncontingent reinforcement. *Research in Autism Spectrum Disorders*, *4*(3), 367–376. https://doi.org/10.1016/j.rasd.2009.10.004

Rose, J. C., & Beaulieu, L. (2019). Assessing the generality and durability of interview‐informed functional analyses and treatment. *Journal of Applied Behavior Analysis*, *52*(1), 271–285. https://doi.org/10.1002/jaba.504

Rozenblat, E., Reeve, K. F., Townsend, D. B., Reeve, S. A., & DeBar, R. M. (2019). Teaching joint attention skills to adolescents and young adults with autism using multiple exemplars and script‐fading procedures. *Behavioral Interventions*, *34*(4), 504–524. https://doi.org/10.1002/bin.1682

Russell, S. M., & Reinecke, D. (2019). Mand acquisition across different teaching methodologies. *Behavioral Interventions*, *34*(1), 127–135. https://doi.org/10.1002/bin.1643

Saini, V., Fisher, W. W., & Pisman, M. D. (2017). Persistence during and resurgence following noncontingent reinforcement implemented with and without extinction. *Journal of Applied Behavior Analysis*, *50*(2), 377–392. https://doi.org/10.1002/jaba.380

Saini, V., Greer, B. D., Fisher, W. W., Lichtblau, K. R., DeSouza, A. A., & Mitteer, D. R. (2016). Individual and combined effects of noncontingent reinforcement and response blocking on automatically reinforced problem behavior. *Journal of Applied Behavior Analysis*, *49*(3), 693–698. https://doi.org/10.1002/jaba.306

Saini, V., Gregory, M. K., Uran, K. J., & Fantetti, M. A. (2015). Parametric analysis of response interruption and redirection as treatment for stereotypy. *Journal of Applied Behavior Analysis*, *48*(1), 96–106. https://doi.org/10.1002/jaba.186

Sallows, G. O., & Graupner, T. D. (2005). Intensive behavioral treatment for children with autism: four-year outcome and predictors. *American Journal of Mental Retardation*, *110*(6), 417–438. https://doi.org/10.1352/0895-8017(2005)110[417:IBTFCW]2.0.CO;2

Sansosti, F. J., & Powell-Smith, K. A. (2008). Using Computer-Presented Social Stories and Video Models to Increase the Social Communication Skills of Children With High-Functioning Autism Spectrum Disorders. *Journal of Positive Behavior Interventions*, *10*(3), 162–178. https://doi.org/10.1177/1098300708316259

Sarokoff, R. A., Taylor, B. A., & Poulson, C. L. (2001). Teaching children with autism to engage in conversational exchanges: Script fading with embedded textual stimuli. *Journal of Applied Behavior Analysis*, *34*(1), 81–84. https://doi.org/10.1901/jaba.2001.34-81

Sawyer, L. M. C., Luiselli, J. K., Ricciardi, J. N., & Gower, J. L. (2005). Teaching a child with autism to share among peers in an integrated preschool classroom: Acquisition, maintenance, and social validation. *Education and Treatment of Children*, *28*(1), 1–10.

Saylor, S., Sidener, T. M., Reeve, S. A., Fetherston, A., & Progar, P. R. (2012). Effects of three types of noncontingent auditory stimulation on vocal stereotypy in children with autism. *Journal of Applied Behavior Analysis*, *45*(1), 185–190. https://doi.org/10.1901/jaba.2012.45-185

Scattone, D., & Billhofer, B. (2008). Teaching sign language to a nonvocal child with autism. *The Journal of Speech and Language Pathology – Applied Behavior Analysis*, *3*(1), 78–85. https://doi.org/10.1037/h0100234

Scheithauer, M., Call, N. A., Lomas Mevers, J., McCracken, C. E., & Scahill, L. (2020). A Feasibility Randomized Clinical Trial of a Structured Function-Based Intervention for Elopement in Children with Autism Spectrum Disorder. *Journal of Autism and Developmental Disorders*, *51*(8), 2866–2875. https://doi.org/10.1007/s10803-020-04753-4

Scheithauer, M., Muething, C. S., Silva, M. R., Gerencser, K. R., Krantz, J., & Call, N. A. (2019). Using caregiver report on the impact of challenging behavior exhibited by children with autism spectrum disorder to guide treatment development and outcomes. *International Journal of Developmental Disabilities*, *65*(4), 265–276. https://doi.org/10.1080/20473869.2018.1428521

Schiff, A., Tarbox, J., Lanagan, T., & Farag, P. (2011). Establishing compliance with liquid medication administration in a child with autism. *Journal of Applied Behavior Analysis*, *44*(2), 381–385. https://doi.org/10.1901/jaba.2011.44-381

Schnell, L. K., Vladescu, J. C., Kodak, T., & Nottingham, C. L. (2018). Comparing procedures on the acquisition and generalization of tacts for children with autism spectrum disorder. *Journal of Applied Behavior Analysis*, *51*(4), 769–783. https://doi.org/10.1002/jaba.480

Schrandt, J. A., Townsend, D. B., & Poulson, C. L. (2009). Teaching empathy skills to children with autism. *Journal of Applied Behavior Analysis*, *42*(1), 17–32. https://doi.org/10.1901/jaba.2009.42-17

Schreck, K. A., Metz, B., Mulick, J. A., & Smith, A. (2000). Making it fit: A provocative look at models of early intensive behavioral intervention for children with autism. *The Behavior Analyst Today*, *1*(3), 27–32. https://doi.org/10.1037/h0099886

Schumacher, B. I., & Rapp, J. T. (2011). Evaluation of the immediate and subsequent effects of response interruption and redirection on vocal stereotypy. *Journal of Applied Behavior Analysis*, *44*(3), 681–685. https://doi.org/10.1901/jaba.2011.44-681

Seaver, J. L., & Bourret, J. C. (2014). An evaluation of response prompts for teaching behavior chains. *Journal of Applied Behavior Analysis*, *47*(4), 777–792. https://doi.org/10.1002/jaba.159

Seaver, J. P., & Bourret, J. C. (2020). Producing mands in concurrent operant environments. *Journal of Applied Behavior Analysis*, *53*(1), 366–384. https://doi.org/10.1002/jaba.592

Shabani, D. B., & Fisher, W. W. (2006). Stimulus Fading and Differential Reinforcement for the Treatment of Needle Phobia in a Youth with Autism. *Journal of Applied Behavior Analysis*, *39*(4), 449–452. https://doi.org/10.1901/jaba.2006.30-05

Shabani, D. B., Katz, R. C., Wilder, D. A., Beauchamp, K., Taylor, C. R., & Fischer, K. J. (2002). Increasing social initiations in children with autism: Effects of a tactile prompt. *Journal of Applied Behavior Analysis*, *35*(1), 79–83. https://doi.org/10.1901/jaba.2002.35-79

Shawler, L. A., Dianda, M., & Miguel, C. F. (2020). A comparison of response interruption and redirection and competing items on vocal stereotypy and appropriate vocalizations. *Journal of Applied Behavior Analysis*, *53*(1), 355–365. https://doi.org/10.1002/jaba.596

Sheinkopf, S. J., & Siegel, B. (1998). Home-based behavioral treatment of young children with autism. *Journal of Autism and Developmental Disorders*, *28*(1), 15–23. https://doi.org/10.1023/A:1026054701472

Sherer, M., Pierce, K. L., Paredes, S., Kisacky, K. L., Ingersoll, B., & Schreibman, L. (2001). Enhancing Conversation Skills in Children with Autism Via Video Technology: Which Is Better, “Self” or “Other” as a Model? *Behavior Modification*, *25*(1), 140–158. https://doi.org/10.1177/0145445501251008

Shillingsburg, M. A., Gayman, C. M., & Walton, W. (2016). Using textual prompts to teach mands for information using “who?”. *Analysis of Verbal Behavior*, *32*(1), 1–14. https://doi.org/10.1007/s40616-016-0053-7

Shillingsburg, M. A., Marya, V., Bartlett, B. L., & Thompson, T. M. (2019). Teaching mands for information using speech generating devices: A replication and extension. *Journal of Applied Behavior Analysis*, *52*(3), 756–771. https://doi.org/10.1002/jaba.579

Shipley-Benamou, R., Lutzker, J. R., & Taubman, M. (2002). Teaching Daily Living Skills to Children with Autism Through Instructional Video Modeling. *Journal of Positive Behavior Interventions*, *4*(3), 166–177. https://doi.org/10.1177/10983007020040030501

Sidener, T. M., Carr, J. E., & Firth, A. M. (2005). Superimposition and withholding of edible consequences as treatment for automatically reinforced stereotypy. *Journal of Applied Behavior Analysis*, *38*(1), 121–124. https://doi.org/10.1901/jaba.2005.58-04

Sigafoos, J., & Meikle, B. (1996). Functional Communication Training for the Treatment of Multiply Determined Challenging Behavior in Two Boys with Autism. *Behavior Modification*, *20*(1), 60–84. https://doi.org/10.1177/01454455960201003

Silbaugh, B. C., & Swinnea, S. (2019). Failure to Replicate the Effects of the High-Probability Instructional Sequence on Feeding in Children With Autism and Food Selectivity. *Behavior Modification*, *43*(5), 734–762. https://doi.org/10.1177/0145445518785111

Silla-Zaleski, V. A., & Vesloski, M. J. (2010). Using DRO, behavioral momentum, and self-regulation to reduce scripting by an adolescent with autism. *The Journal of Speech and Language Pathology – Applied Behavior Analysis*, *5*(1), 80–87. https://doi.org/10.1037/h0100264

Sivaraman, M., & Bhabu, P. (2018). Establishment of exclusion responding in children with autism spectrum disorder. *Behavioral Interventions*, *33*(4), 414–426. https://doi.org/10.1002/bin.1647

Slaton, J. D., & Hanley, G. P. (2016). Effects of multiple versus chained schedules on stereotypy and item engagement. *Journal of Applied Behavior Analysis*, *49*(4), 927–946. https://doi.org/10.1002/jaba.345

Slaton, J. D., Hanley, G. P., & Raftery, K. J. (2017). Interview‐informed functional analyses: A comparison of synthesized and isolated components. *Journal of Applied Behavior Analysis*, *50*(2), 252–277. https://doi.org/10.1002/jaba.384

Slifer, K. J., Koontz, K. L., & Cataldo, M. F. (2002). Operant-contingency-based preparation of children for functional magnetic resonance imaging. *Journal of Applied Behavior Analysis*, *35*(2), 191–194. https://doi.org/10.1901/jaba.2002.35-191

Slocum, S. K., Miller, S. J., & Tiger, J. H. (2012). Using a blocked-trials procedure to teach identity matching to a child with autism. *Journal of Applied Behavior Analysis*, *45*(3), 619–624. https://doi.org/10.1901/jaba.2012.45-619

Slocum, S. K., & Tiger, J. H. (2011). An assessment of the efficiency of and child preference for forward and backward chaining. *Journal of Applied Behavior Analysis*, *44*(4), 793–805. https://doi.org/10.1901/jaba.2011.44-793

Slocum, S. K., & Vollmer, T. R. (2015). A comparison of positive and negative reinforcement for compliance to treat problem behavior maintained by escape. *Journal of Applied Behavior Analysis*, *48*(3), 563–574. https://doi.org/10.1002/jaba.216

Smith, I. M., Koegel, R. L., Koegel, L. K., Openden, D. A., Fossum, K. L., & Bryson, S. E. (2010). Effectiveness of a Novel Community-Based Early Intervention Model for Children With Autistic Spectrum Disorder. *American Journal on Intellectual and Developmental Disabilities*, *115*(6), 504–523. https://doi.org/10.1352/1944-7558-115.6.504

Smith, T., Buch, G. A., & Gamby, T. E. (2000). Parent-directed, intensive early intervention for children with pervasive developmental disorder. *Research in Developmental Disabilities*, *21*(4), 297–309. https://doi.org/10.1016/S0891-4222(00)00043-3

Smith, T., Eikeseth, S., Klevstrand, M., & Lovaas, O. I. (1997). Intensive Behavioral Treatment for Preschoolers With Severe Mental Retardation and Pervasive Developmental Disorder. *American Journal on Mental Retardation*, *102*(3), 238–249. https://doi.org/10.1352/0895-8017(1997)102<0238:IBTFPW>2.0.CO;2

Smith, T., Groen, A. D., & Wynn, J. W. (2000). Randomized Trial of Intensive Early Intervention for Children With Pervasive Developmental Disorder. *American Journal on Mental Retardation*, *105*(4), 269–285. https://doi.org/10.1352/0895-8017(2000)105<0269:RTOIEI>2.0.CO;2

Smith, T., Klorman, R., & Mruzek, D. W. (2015). Predicting outcome of community-based early intensive behavioral intervention for children with autism. *Journal of Abnormal Child Psychology*, *43*(7), 1271–1282. https://doi.org/10.1007/s10802-015-0002-2

Soares, E., Miller, T., Ciffone, K., & Read, K. (2020). E-books for children with autism: Best read alone or with a therapist? *Child Language Teaching and Therapy*, *36*(1), 19–32. https://doi.org/10.1177/0265659020903795

Soluaga, D., Leaf, J. B., Taubman, M., McEachin, J., & Leaf, R. (2008). A comparison of flexible prompt fading and constant time delay for five children with autism. *Research in Autism Spectrum Disorders*, *2*(4), 753–765. https://doi.org/10.1016/j.rasd.2008.03.005

Somers, A., Sidener, T. M., DeBar, R. M., & Sidener, D. W. (2014). Establishing concurrent mands for items and mands for information about location in children with autism. *Analysis of Verbal Behavior*, *30*(1), 29–35. https://doi.org/10.1007/s40616-014-0007-x

Stahmer, A. C., & Schreibman, L. (1992). Teaching children with autism appropriate play in unsupervised environments using a self-management treatment package. *Journal of Applied Behavior Analysis*, *25*(2), 447–459. https://doi.org/10.1901/jaba.1992.25-447

Stanislaw, H., Howard, J., & Martin, C. (2020). Helping parents choose treatments for young children with autism: A comparison of  applied behavior analysis and eclectic treatments. *Journal of the American Association of Nurse Practitioners*, *32*(8), 571–578. https://doi.org/10.1097/JXX.0000000000000290

Stanley, C. R., Belisle, J., & Dixon, M. R. (2018). Equivalence‐based instruction of academic skills: Application to adolescents with autism. *Journal of Applied Behavior Analysis*, *51*(2), 352–359. https://doi.org/10.1002/jaba.446

Stasolla, F., Perilli, V., & Damiani, R. (2014). Self monitoring to promote on-task behavior by two high functioning boys with autism spectrum disorders and symptoms of ADHD. *Research in Autism Spectrum Disorders*, *8*(5), 472–479. https://doi.org/10.1016/j.rasd.2014.01.007

Stauch, T. A., & Plavnick, J. B. (2020). Teaching vocational and social skills to adolescents with autism using video modeling. *Education & Treatment of Children*, *43*(2), 137–151. https://doi.org/10.1007/s43494-020-00020-4

Stauch, T. A., Plavnick, J. B., Sankar, S., & Gallagher, A. C. (2018). Teaching social perception skills to adolescents with autism and intellectual disabilities using video‐based group instruction. *Journal of Applied Behavior Analysis*, *51*(3), 647–666. https://doi.org/10.1002/jaba.473

Stauch, T. A. (2018). Video modeling and matrix training: Effects on acquisition and generalization of social skills by adolescents with autism spectrum disorder [ProQuest Information & Learning]. In *Dissertation Abstracts International Section A: Humanities and Social Sciences* (Vol. 79, Issues 12-A(E)).

Stock, R. A., Schulze, K. A., & Mirenda, P. (2008). A Comparison of Stimulus-Stimulus Pairing, Standard Echoic Training, and Control Procedures on the Vocal Behavior of Children With Autism. *The Analysis of Verbal Behavior*, *24*, 123–133. https://doi.org/10.1007/BF03393061

Stock, R., Mirenda, P., & Smith, I. M. (2013). Comparison of community-based verbal behavior and pivotal response treatment programs for young children with autism spectrum disorder. *Research in Autism Spectrum Disorders*, *7*(9), 1168–1181. https://doi.org/10.1016/j.rasd.2013.06.002

Strain, P. S., Kohler, F. W., Storey, K., & Danko, C. D. (1994). Teaching Preschoolers with Autism to Self-Monitor Their Social Interactions: An Analysis of Results in Home and School Settings. *Journal of Emotional and Behavioral Disorders*, *2*(2), 78–88. https://doi.org/10.1177/106342669400200202

Stuesser, H. A., & Roscoe, E. M. (2020). An evaluation of differential reinforcement with stimulus fading as an intervention for medical compliance. *Journal of Applied Behavior Analysis*, *53*(3), 1606–1621. https://doi.org/10.1002/jaba.685

Suberman, R., & Cividini‐Motta, C. (2020). Teaching caregivers to implement mand training using speech generating devices. *Journal of Applied Behavior Analysis*, *53*(2), 1097–1110. https://doi.org/10.1002/jaba.630

Sullivan, W. E., Saini, V., DeRosa, N. M., Craig, A. R., Ringdahl, J. E., & Roane, H. S. (2020). Measurement of nontargeted problem behavior during investigations of resurgence. *Journal of Applied Behavior Analysis*, *53*(1), 249–264. https://doi.org/10.1002/jaba.589

Susa, C., & Schlinger, H. D. J. (2012). Using a lag schedule to increase variability of verbal responding in an individual with autism. *Analysis of Verbal Behavior*, *28*(1), 125–130. https://doi.org/10.1007/bf03393113

Sweeney-Kerwin, E. J., Carbone, V. J., O’Brien, L., Zecchin, G., & Janecky, M. N. (2007). Transferring control of the mand to the motivating operation in children with autism. *Analysis of Verbal Behavior*, *23*, 89–102. https://doi.org/10.1007/BF03393049

Sy, J. R., & Vollmer, T. R. (2012). Discrimination acquisition in children with developmental disabilities under immediate and delayed reinforcement. *Journal of Applied Behavior Analysis*, *45*(4), 667–684. https://doi.org/10.1901/jaba.2012.45-667

Syed, N. Y. (2018). Conditioned seeing as related to bidirectional naming for unfamiliar stimuli with third through fifth grade students diagnosed with autism [ProQuest Information & Learning]. In *Dissertation Abstracts International: Section B: The Sciences and Engineering* (Vol. 79, Issues 7-B(E)).

Szabo, T. G. (2019). Acceptance and Commitment Training for reducing inflexible behaviors in children with autism. *Journal of Contextual Behavioral Science*, *12*, 178–188. https://doi.org/10.1016/j.jcbs.2019.03.001

Szalwinski, J., Thomason‐Sassi, J. L., Moore, E., & McConnell, K. (2019). Effects of decreasing intersession interval duration on graduated exposure treatment during simulated routine dental care. *Journal of Applied Behavior Analysis*, *52*(4), 944–955. https://doi.org/10.1002/jaba.642

Szmacinski, N. J., DeBar, R. M., Sidener, T. M., & Sidener, D. W. (2018). Fading an auditory model by volume to teach mands to children with autism spectrum disorder. *Journal of Developmental and Physical Disabilities*, *30*(5), 653–668. https://doi.org/10.1007/s10882-018-9610-4

Takeuchi, K., Kubota, H., & Yamamoto, J. (2002). Intensive Supervision for Families Conducting Home-Based Behavioral Treatment for Children with Autism in Malaysia. *The Japanese Journal of Special Education*, *39*(6), 155–164. https://doi.org/10.6033/tokkyou.39.155

Tarbox, J., Madrid, W., Aguilar, B., Jacobo, W., & Schiff, A. (2009). Use of chaining to increase complexity of echoics in children with autism. *Journal of Applied Behavior Analysis*, *42*(4), 901–906. https://doi.org/10.1901/jaba.2009.42-901

Tarbox, R. S. F., Tarbox, J., Ghezzi, P. M., Wallace, M. D., & Yoo, J. H. (2007). The effects of blocking mouthing of leisure items on their effectiveness as reinforcers. *Journal of Applied Behavior Analysis*, *40*(4), 761–765. https://doi.org/10.1901/jaba.2007.761-765

Tarbox, R. S. F., Wallace, M. D., & Williams, L. (2003). Assessment and treatment of elopement: A replication and extension. *Journal of Applied Behavior Analysis*, *36*(2), 239–244. https://doi.org/10.1901/jaba.2003.36-239

Taylor, B. A., & Harris, S. L. (1995). Teaching children with autism to seek information: acquisition of novel information and generalization of responding. *Journal of Applied Behavior Analysis*, *28*(1), 3–14. https://doi.org/10.1901/jaba.1995.28-3

Taylor, B. A., Levin, L., & Jasper, S. (1999). Increasing Play-Related Statements in Children with Autism Toward Their Siblings: Effects of Video Modeling. *Journal of Developmental and Physical Disabilities*, *11*(3), 253–264. https://doi.org/10.1023/A:1021800716392

Taylor, B. A., DeQuinzio, J. A., & Stine, J. (2012). Increasing observational learning of children with autism: A preliminary analysis. *Journal of Applied Behavior Analysis*, *45*(4), 815–820. https://doi.org/10.1901/jaba.2012.45-815

Taylor, B. A., & Hoch, H. (2008). Teaching children with autism to respond to and initiate bids for joint attention. *Journal of Applied Behavior Analysis*, *41*(3), 377–391. https://doi.org/10.1901/jaba.2008.41-377

Taylor, B. A., Hughes, C. E., Richard, E., Hoch, H., & Coello, A. R. (2004). Teaching teenagers with autism to seek assistance when lost. *Journal of Applied Behavior Analysis*, *37*(1), 79–82. https://doi.org/10.1901/jaba.2004.37-79

Taylor, B. A., & Levin, L. (1998). Teaching a student with autism to make verbal initiations: Effects of a tactile prompt. *Journal of Applied Behavior Analysis*, *31*(4), 651–654. https://doi.org/10.1901/jaba.1998.31-651

Taylor, T. (2020). Increasing food texture and teaching chewing for a clinical case within the home setting in Australia. *Learning and Motivation*, *71*. https://doi.org/10.1016/j.lmot.2020.101651

Taylor‐Santa, C., Sidener, T. M., Carr, J. E., & Reeve, K. F. (2014). A discrimination training procedure to establish conditioned reinforcers for children with autism. *Behavioral Interventions*, *29*(2), 157–176. https://doi.org/10.1002/bin.1384

Thiemann, K. S., & Goldstein, H. (2004). Effects of Peer Training and Written Text Cueing on Social Communication of School-Age Children With Pervasive Developmental Disorder. *Journal of Speech, Language, and Hearing Research*, *47*(1), 126–144. https://doi.org/10.1044/1092-4388(2004/012)

Thiemann, K. S., & Goldstein, H. (2001). Social stories, written text cues, and video feedback: Effects on social communication of children with autism. *Journal of Applied Behavior Analysis*, *34*(4), 425–446. https://doi.org/10.1901/jaba.2001.34-425

Thomas, E. M., DeBar, R. M., Vladescu, J. C., & Townsend, D. B. (2020). A comparison of video modeling and video prompting by adolescents with ASD. *Behavior Analysis in Practice*, *13*(1), 40–52. https://doi.org/10.1007/s40617-019-00402-0

Thorp, D. M., Stahmer, A. C., & Schreibman, L. (1995). Effects of sociodramatic play training on children with autism. *Journal of Autism and Developmental Disorders*, *25*(3), 265–282. https://doi.org/10.1007/BF02179288

Thrailkill, E. A., Kimball, R. T., Kelley, M. E., Craig, A. R., & Podlesnik, C. A. (2018). Greater reinforcement rate during training increases spontaneous recovery. *Journal of the Experimental Analysis of Behavior*, *109*(1), 238–252. https://doi.org/10.1002/jeab.307

Tiger, J. H., Toussain, K. A., & Roath, C. T. (2010). An evaluation of the value of choice-making opportunities in single-operant arrangements: Simple fixed- and progressive-ratio schedules. *Journal of Applied Behavior Analysis*, *43*(3), 519–524. https://doi.org/10.1901/jaba.2010.43-519

Tincani, M. (2004). Comparing the Picture Exchange Communication System and Sign Language Training for Children With Autism. *Focus on Autism and Other Developmental Disabilities*, *19*(3), 152–163. https://doi.org/10.1177/10883576040190030301

Toper‐Korkmaz, O., Lerman, D. C., & Tsami, L. (2018). Effects of toy removal and number of demands on vocal stereotypy during response interruption and redirection. *Journal of Applied Behavior Analysis*, *51*(4), 757–768. https://doi.org/10.1002/jaba.497

Torres, R., DeBar, R. M., Reeve, S. A., Meyer, L. S., & Covington, T. M. (2018). The effects of a video-enhanced schedule on exercise behavior. *Behavior Analysis in Practice*, *11*(2), 85–96. https://doi.org/10.1007/s40617-018-0224-1

Toussaint, K. A., Kodak, T., & Vladescu, J. C. (2016). An evaluation of choice on instructional efficacy and individual preferences among children with autism. *Journal of Applied Behavior Analysis*, *49*(1), 170–175. https://doi.org/10.1002/jaba.263

Tsami, L., & Lerman, D. C. (2020). Transfer of treatment effects from combined to isolated conditions during functional communication training for multiply controlled problem behavior. *Journal of Applied Behavior Analysis*, *53*(2), 649–664. https://doi.org/10.1002/jaba.629

Tsami, L., Lerman, D., & Toper‐Korkmaz, O. (2019). Effectiveness and acceptability of parent training via telehealth among families around the world. *Journal of Applied Behavior Analysis*, *52*(4), 1113–1129. https://doi.org/10.1002/jaba.645

Tung, R. (2005). A comparison: The effects of discrete trial training on children with autism. *ProQuest Dissertations and Theses*. https://doi.org/10.31979/etd.xumc-dkf5

Twarek, M., Cihon, T., & Eshleman, J. (2010). The effects of fluent levels of big 6 + 6 skill elements on functional motor skills with children with autism. *Behavioral Interventions*, *25*(4), 275–293. https://doi.org/10.1002/bin.317

Ulke-Kurkcuoglu, B. (2015). A comparison of least-to-most prompting and video modeling for teaching pretend play skills to children with autism spectrum disorder. *Kuram ve Uygulamada Eğitim Bilimleri*, *15*(2), 499–517. https://doi.org/10.12738/estp.2015.2.2541

Ulke-Kurkcuoglu, B., & Kircaali-Iftar, G. (2010). A comparison of the effects of providing activity and material choice to children with autism spectrum disorders. *Journal of Applied Behavior Analysis*, *43*(4), 717–721. https://doi.org/10.1901/jaba.2010.43-717

Valentino, A. L., Conine, D. E., Delfs, C. H., & Furlow, C. M. (2015). Use of a modified chaining procedure with textual prompts to establish intraverbal storytelling. *Analysis of Verbal Behavior*, *31*(1), 39–58. https://doi.org/10.1007/s40616-014-0023-x

Valentino, A. L., LeBlanc, L. A., & Raetz, P. B. (2018). Evaluation of stimulus intensity fading on reduction of rapid eating in a child with autism. *Journal of Applied Behavior Analysis*, *51*(1), 177–182. https://doi.org/10.1002/jaba.433

Vallinger-Brown, M., & Rosales, R. (2014). An investigation of stimulus pairing and listener training to establish emergent intraverbals in children with autism. *Analysis of Verbal Behavior*, *30*(2), 148–159. https://doi.org/10.1007/s40616-014-0014-y

Van Laarhoven, T., Kraus, E., Karpman, K., Nizzi, R., & Valentino, J. (2010). A Comparison of Picture and Video Prompts to Teach Daily Living Skills to Individuals With Autism. *Focus on Autism and Other Developmental Disabilities*, *25*(4), 195–208. https://doi.org/10.1177/1088357610380412

Varella, A. A. B., & de Souza, D. G. (2015). Using class‐specific compound consequences to teach dictated and printed letter relations to a child with autism. *Journal of Applied Behavior Analysis*, *48*(3), 675–679. https://doi.org/10.1002/jaba.224

Vaughn, B. J., Clarke, S., & Dunlap, G. (1997). Assessment-based intervention for severe behavior problems in a natural family context. *Journal of Applied Behavior Analysis*, *30*(4), 713–716. https://doi.org/10.1901/jaba.1997.30-713

Vedora, J., & Barry, T. (2016). The use of picture prompts and prompt delay to teach receptive labeling. *Journal of Applied Behavior Analysis*, *49*(4), 960–964. https://doi.org/10.1002/jaba.336

Vedora, J., Barry, T., & Ward-Horner, J. C. (2019). Sample first versus comparison first stimulus presentations: Preliminary findings for two individuals with autism. *Behavior Analysis in Practice*, *12*(2), 423–429. https://doi.org/10.1007/s40617-018-00299-1

Vedora, J., & Grandelski, K. (2015). A comparison of methods for teaching receptive language to toddlers with autism. *Journal of Applied Behavior Analysis*, *48*(1), 188–193. https://doi.org/10.1002/jaba.167

Vedora, J., Meunier, L., & Mackay, H. (2009). Teaching Intraverbal Behavior to Children with Autism: A Comparison of Textual and Echoic Prompts. *The Analysis of Verbal Behavior*, *25*, 79–86. https://doi.org/10.1007/BF03393072

Verriden, A. L., & Roscoe, E. M. (2019). An evaluation of a punisher assessment for decreasing automatically reinforced problem behavior. *Journal of Applied Behavior Analysis*, *52*(1), 205–226. https://doi.org/10.1002/jaba.509

Vietze, P., & Lax, L. E. (2020). Early intervention ABA for toddlers with ASD: Effect of age and amount. *Current Psychology: A Journal for Diverse Perspectives on Diverse Psychological Issues*, *39*(4), 1234–1244. https://doi.org/10.1007/s12144-018-9812-z

Vignes, T. (2007). A Comparison of Topography-based and Selection-based Verbal Behavior in Typically Developed Children and Developmentally Disabled Persons with Autism. *The Analysis of Verbal Behavior*, *23*, 113–122. https://doi.org/10.1007/BF03393051

Vismara, L. A., & Lyons, G. L. (2007). Using Perseverative Interests to Elicit Joint Attention Behaviors in Young Children With Autism: Theoretical and Clinical Implications for Understanding Motivation. *Journal of Positive Behavior Interventions*, *9*(4), 214–228. https://doi.org/10.1177/10983007070090040401

Vismara, L. A., Young, G. S., Stahmer, A. C., Griffith, E. M., & Rogers, S. J. (2009). Dissemination of Evidence-Based Practice: Can We Train Therapists from a Distance? *Journal of Autism and Developmental Disorders*, *39*, 1636–1651. https://doi.org/10.1007/s10803-009-0796-2

Vismara, L. A., Colombi, C., & Rogers, S. J. (2009). Can one hour per week of therapy lead to lasting changes in young children with autism? *Autism: The International Journal of Research & Practice*, *13*(1), 93–115. https://doi.org/10.1177/1362361307098516

Vivanti, G., Paynter, J., Duncan, E., Fothergill, H., Dissanayake, C., Rogers, S. J., & the Victorian ASELCC Team. (2014). Effectiveness and Feasibility of the Early Start Denver Model Implemented in a Group-Based Community Childcare Setting. *Journal of Autism and Developmental Disorders*, *44*(12), 3140–3153. https://doi.org/10.1007/s10803-014-2168-9

Vladescu, J. C., & Kodak, T. (2016). The effect of a multiple-schedule arrangement on mands of a child with autism. *Behavioral Interventions*, *31*(1), 3–11. https://doi.org/10.1002/bin.1422

Vladescu, J. C., & Kodak, T. M. (2013). Increasing instructional efficiency by presenting additional stimuli in learning trials for children with autism spectrum disorders. *Journal of Applied Behavior Analysis*, *46*(4), 805–816. https://doi.org/10.1002/jaba.70

Volkert, V. M., Lerman, D. C., Call, N. A., & Trosclair-Lasserre, N. (2009). An evaluation of resurgence during treatment with functional communication training. *Journal of Applied Behavior Analysis*, *42*(1), 145–160. https://doi.org/10.1901/jaba.2009.42-145

Volkert, V. M., Lerman, D. C., Trosclair, N., Addison, L., & Kodak, T. (2008). An exploratory analysis of task-interspersal procedures while teaching object labels to children with autism. *Journal of Applied Behavior Analysis*, *41*(3), 335–350. https://doi.org/10.1901/jaba.2008.41-335

Vosters, M. E., & Luczynski, K. C. (2020). Emergent completion of multistep instructions via joint control. *Journal of Applied Behavior Analysis*, *53*(3), 1432–1451. https://doi.org/10.1002/jaba.670

Wacker, D. P., Lee, J. F., Dalmau, Y. C. P., Kopelman, T. G., Lindgren, S. D., Kuhle, J., Pelzel, K. E., Dyson, S., Schieltz, K. M., & Waldron, D. B. (2013). Conducting functional communication training via telehealth to reduce the problem behavior of young children with autism. *Journal of Developmental and Physical Disabilities*, *25*(1), 35–48. https://doi.org/10.1007/s10882-012-9314-0

Waldron-Soler, K. M., Martella, R. C., Marchand-Martella, N. E., & Ebey, T. L. (2000). Effects of choice of stimuli as reinforcement for task responding in preschoolers with and without developmental disabilities. *Journal of Applied Behavior Analysis*, *33*(1), 93–96. https://doi.org/10.1901/jaba.2000.33-93

Waters, C. F., Amerine Dickens, M., Thurston, S. W., Lu, X., & Smith, T. (2020). Sustainability of early intensive behavioral intervention for children with autism spectrum disorder in a community setting. *Behavior Modification*, *44*(1), 3–26. https://doi.org/10.1177/0145445518786463

Waters, M. B., Lerman, D. C., & Hovanetz, A. N. (2009). Separate and combined effects of visual schedules and extinction plus differential reinforcement on problem behavior occasioned by transitions. *Journal of Applied Behavior Analysis*, *42*(2), 309–313. https://doi.org/10.1901/jaba.2009.42-309

Watkins, L., O’Reilly, M., Kuhn, M., & Ledbetter‐Cho, K. (2019). An interest‐based intervention package to increase peer social interaction in young children with autism spectrum disorder. *Journal of Applied Behavior Analysis*, *52*(1), 132–149. https://doi.org/10.1002/jaba.514

Watkins, N., Paananen, L., Rudrud, E., & Rapp, J. T. (2011). Treating vocal stereotypy with environmental enrichment and response cost. *Clinical Case Studies*, *10*(6), 440–448. https://doi.org/10.1177/1534650111429377

Watts, A. C., Wilder, D. A., Gregory, M. K., Leon, Y., & Ditzian, K. (2013). The effect of rules on differential reinforcement of other behavior. *Journal of Applied Behavior Analysis*, *46*(3), 680–684. https://doi.org/10.1002/jaba.53

Weiss, M. J. (1999). Differential rates of skill acquisition and outcomes of early intensive behavioral intervention for autism. *Behavioral Interventions*, *14*(1), 3–22. https://doi.org/10.1002/(SICI)1099-078X(199901/03)14:1<3::AID-BIN25>3.0.CO;2-F

Weiss, M. J., & Delmolino, L. (2006). The relationship between early learning rates and treatment outcome for children with autism receiving intensive home-based applied behavior analysis. *The Behavior Analyst Today*, *7*(1), 96–110. https://doi.org/10.1037/h0100140

Welsh, F., Najdowski, A. C., Strauss, D., Gallegos, L., & Fullen, J. A. (2019). Teaching a perspective‐taking component skill to children with autism in the natural environment. *Journal of Applied Behavior Analysis*, *52*(2), 439–450. https://doi.org/10.1002/jaba.523

Werntz, E. J. (2020). An evaluation of a more-adaptive treatment of elopement using modern technology for children with developmental disabilities [ProQuest Information & Learning]. In *Dissertation Abstracts International Section A: Humanities and Social Sciences* (Vol. 81, Issues 2-A).

Weston, R., Davis, T., & Ross, R. K. (2020). Evaluating preference and performance in accumulated versus distributed response–reinforcer arrangements. *Behavior Modification*, *44*(6), 909–926. https://doi.org/10.1177/0145445519868793

Whalen, C., Liden, L., Ingersoll, B., Dallaire, E., & Liden, S. (2006). Behavioral improvements associated with computer-assisted instruction for children with developmental disabilities. *The Journal of Speech and Language Pathology – Applied Behavior Analysis*, *1*(1), 11–26. https://doi.org/10.1037/h0100182

Whalen, C., & Schreibman, L. (2003). Joint attention training for children with autism using behavior modification procedures. *Journal of Child Psychology and Psychiatry*, *44*(3), 456–468. https://doi.org/10.1111/1469-7610.00135

Wheatley, T. L., Goulet, M., Mann, K., & Lanovaz, M. J. (2020). Differential negative reinforcement of other behavior to increase compliance with wearing an anti‐strip suit. *Journal of Applied Behavior Analysis*, *53*(2), 1153–1161. https://doi.org/10.1002/jaba.632

Wichnick‐Gillis, A. M., Vener, S. M., & Poulson, C. L. (2019). Script Fading for Children with Autism: Generalization of Social Initiation Skills from School to Home. *Journal of Applied Behavior Analysis*, *52*(2), 451–466. https://doi.org/10.1002/jaba.534

Wilder, D. A., Ertel, H., Hodges, A. C., Thomas, R., & Luong, N. (2020). The use of auditory feedback and edible reinforcement to decrease toe walking among children with autism. *Journal of Applied Behavior Analysis*, *53*(1), 554–562. https://doi.org/10.1002/jaba.607

Wilder, D. A., Ertel, H., & Thomas, R. (2020). Further analysis of modifications to the three‐step guided compliance procedure to enhance compliance among children with autism. *Journal of Applied Behavior Analysis*, *53*(4), 2339–2348. https://doi.org/10.1002/jaba.721

Williams, G., Carnerero, J. J., & Pérez-González, L. A. (2006). Generalization of tacting actions in children with autism. *Journal of Applied Behavior Analysis*, *39*(2), 233–237. https://doi.org/10.1901/jaba.2006.175-04

Williams, G., Donley, C. R., & Keller, J. W. (2000). Teaching children with autism to ask questions about hidden objects. *Journal of Applied Behavior Analysis*, *33*(4), 627–630. https://doi.org/10.1901/jaba.2000.33-627

Williams, G., Pérez-González, L. A., & Queiroz, A. B. M. (2005). Using a combined blocking procedure to teach color discrimination to a child with autism. *Journal of Applied Behavior Analysis*, *38*(4), 555–558. https://doi.org/10.1901/jaba.2005.65-04

Williams, G., Pérez-González, L. A., & Vogt, K. (2003). The role of specific consequences in the maintenance of three types of questions. *Journal of Applied Behavior Analysis*, *36*(3), 285–296. https://doi.org/10.1901/jaba.2003.36-285

Wiskow, K. M., & Klatt, K. P. (2013). The effects of awareness training on tics in a young boy with Tourette syndrome, Asperger syndrome, and attention deficit hyperactivity disorder. *Journal of Applied Behavior Analysis*, *46*(3), 695–698. https://doi.org/10.1002/jaba.59

Wolery, M., Gast, D. L., Kirk, K., & Schuster, J. (1988). Fading extra-stimulus prompts with autistic children using time delay. *Education and Treatment of Children*, *11*(1), 29–44.

Wood, S., Christian, M. P., & Sampson, A. (2018). Audit of outcomes following a community‐based early intensive behaviour intervention program for children with autism in Australia. *Australian Journal of Psychology*, *70*(3), 217–224. https://doi.org/10.1111/ajpy.12193

Woods, D. W., & Himle, M. B. (2004). Creating tic suppression: Comparing the effects of verbal instruction to differential reinforcement. *Journal of Applied Behavior Analysis*, *37*(3), 417–420. https://doi.org/10.1901/jaba.2004.37-417

Wunderlich, K. L., & Vollmer, T. R. (2017). Effects of serial and concurrent training on receptive identification tasks: A systematic replication. *Journal of Applied Behavior Analysis*, *50*(3), 641–652. https://doi.org/10.1002/jaba.401

Yamamoto, S., & Isawa, S. (2020). Effects of textual prompts and feedback on social niceties of adolescents with autism spectrum disorder in a simulated workplace. *Journal of Applied Behavior Analysis*, *53*(3), 1404–1418. https://doi.org/10.1002/jaba.667

Young, J. M., Krantz, P. J., McClannahan, L. E., & Poulson, C. L. (1994). Generalized imitation and response-class formation in children with autism. *Journal of Applied Behavior Analysis*, *27*(4), 685–697. https://doi.org/10.1901/jaba.1994.27-685

Zachor, D. A., Ben-Itzchak, E., Rabinovich, A.-L., & Lahat, E. (2007). Change in autism core symptoms with intervention. *Research in Autism Spectrum Disorders*, *1*(4), 304–317. https://doi.org/10.1016/j.rasd.2006.12.001

Zachor, D. A., & Itzchak, E. Ben. (2010). Treatment approach, autism severity and intervention outcomes in young children. *Research in Autism Spectrum Disorders*, *4*(3), 425–432. https://doi.org/10.1016/j.rasd.2009.10.013

Zambolin, K., Fabrizio, M., Ferris, K., Barclay, S., & Carrier, D. (2007). Tracking teachers’ behavior to concurrently decrease punishment use with and problem behavior in a child with autism while decreasing the child’s frequency of negative statements. *Journal of Precision Teaching & Celeration*, *23*, 27–29.

Zifferblatt, S. M., Burton, S. D., Homer, R., & White, T. (1977). Establishing generalization effects among autistic children. *Journal of Autism and Childhood Schizophrenia*, *7*(4), 337–347. https://doi.org/10.1007/BF01540392
