# Supplementary material for: Applied Behavior Analysis in Children and Youth with Autism Spectrum Disorders: A Scoping Review
Source: Perspect Behav Sci. 2022 May 18;45(3):521–57. doi: 10.1007/s40614-022-00338-x (PMC9458805; doi:10.1007/s40614-022-00338-x)
Supplement: Supplementary file 5 — (DOCX 38 kb) [file 40614_2022_338_MOESM5_ESM.docx]

Appendix 5.

*Additional Tables*

*Table S4. Most common outcomes in the ABA impact group measured by age category. Italicized and underlined values indicate most frequently measured outcome. “% improved” represents the proportion of the corresponding outcome measure that showed improvement.*

| **AGE** | **0-5** | | **6-12** | | **13-18** | | **Mixed** | |
| --- | --- | --- | --- | --- | --- | --- | --- | --- |
|  | *n (%)* | *% Improved* | *n (%)* | *% Improved* | *n (%)* | *% Improved* | *n (%)* | *% Improved* |
| **COGNITIVE** | 83 (22%) | 55 (66%) | 43 (19%) | 33 (77%) | *18 (26%)* | 15 (83%) | 78(20%) | 48 (62%) |
| **LANGUAGE** | *85 (23%)* | 58 (68%) | *56 (25%)* | 40 (71%) | 13 (19%) | 10 (77%) | *110(28%)* | 67 (61%) |
| **SOCIAL/COMMUNICATION** | 84 (23%) | 48 (57%) | 43 (19%) | 28 (65%) | 13 (19%) | 9 (69%) | 80 (20%) | 52 (65%) |
| **PROBLEM BEHAVIOR** | 40 (11%) | 26 (65%) | *56 (25%)* | 48(86%) | 11 (16%) | 9 (82%) | 67 (17%) | 43 (64%) |
| **ADAPTIVE BEHAVIOR** | 55 (15%) | 37 (67%) | 21 (9%) | 15 (71%) | 14 (20%) | 12 (86%) | 46 (12%) | 31 (67%) |
| **EMOTIONAL** | 4 (1%) | 4 (100%) | 4 (2%) | 4 (100%) | 1 (1%) | 1(100%) | 7 (2%) | 5 (71%) |
| **AUTISM SYMPTOMS** | 21 (6%) | 15 (71%) | 3 (1%) | 1 (33%) | 0 (0%) | N/A | 8 (2%) | 6 (75%) |
| **TOTAL** | 372 |  | 226 |  | 70 |  | 396 |  |

*Table S5. Findings in the ABA impact group categorized by diagnosis.*

|  | **COGNITIVE** | | | | | **LANGUAGE** | | | | | **SOCIAL/COMMUNICATION** | | | | |
| --- | --- | --- | --- | --- | --- | --- | --- | --- | --- | --- | --- | --- | --- | --- | --- |
| **DIAGNOSIS** | Improved | Regressed | Mixed | No Change | Total | Improved | Regressed | Mixed | No Change | Total | Improved | Regressed | Mixed | No Change | Total |
| **ASD** | 131 | 2 | 58 | 2 | 193 | 150 | 2 | 65 | 4 | 221 | 118 | 2 | 67 | 1 | 188 |
| **%** | 68% | 1% | 30% | 1% |  | 68% | 1% | 29% | 2% |  | 63% | 1% | 36% | 1% |  |
| **MIXED** | 20 | 0 | 9 | 0 | 29 | 25 | 0 | 18 | 0 | 43 | 21 | 1 | 12 | 0 | 34 |
| **%** | 69% | 0% | 31% | 0% |  | 58% | 0% | 42% | 0% |  | 62% | 3% | 35% | 0% |  |
| **Total** | 151 | 2 | 67 | 2 | 222 | 175 | 2 | 83 | 4 | 264 | 139 | 3 | 79 | 1 | 222 |
|  | **PROBLEM BEHAVIOR** | | | | | **ADAPTIVE BEHAVIOR** | | | | | **EMOTIONAL** | | | | |
| **DIAGNOSIS** | Improved | Regressed | Mixed | No Change | Total | Improved | Regressed | Mixed | No Change | Total | Improved | Regressed | Mixed | No Change | Total |
| **ASD** | 83 | 1 | 25 | 4 | 113 | 75 | 3 | 28 | 4 | 110 | 13 | 0 | 1 | 0 | 14 |
| **%** | 73% | 1% | 22% | 4% |  | 68% | 3% | 25% | 4% |  | 93% | N/A | 7% | N/A |  |
| **MIXED** | 43 | 3 | 15 | 0 | 61 | 21 | 0 | 6 | 0 | 27 | 1 | 0 | 1 | 0 | 2 |
| **%** | 70% | 5% | 25% | 0% |  | 78% | 0% | 22% | 0% |  | 50% | N/A | 50% | N/A |  |
| **Total** | 126 | 4 | 40 | 4 | 174 | 96 | 3 | 34 | 4 | 137 | 14 | 0 | 2 | 0 | 16 |
|  | **AUTISM SYMPTOMS** | | | | |  |  |  |  |  |  |  |  |  |  |
| **DIAGNOSIS** | Improved | Regressed | Mixed | No Change | Total |  |  |  |  |  |  |  |  |  |  |
| **ASD** | 19 | 0 | 6 | 4 | 29 |  |  |  |  |  |  |  |  |  |  |
| **%** | 66% | N/A | 21% | 14% |  |  |  |  |  |  |  |  |  |  |  |
| **MIXED** | 3 | 0 | 0 | 0 | 3 |  |  |  |  |  |  |  |  |  |  |
| **%** | 100% | 0% | 0% | 0% |  |  |  |  |  |  |  |  |  |  |  |
| **Total** | 22 | 0 | 6 | 4 | 32 |  |  |  |  |  |  |  |  |  |  |

*Table S6. Most common outcomes in the ABA impact group measured by diagnosis category. Italicized and underlined values indicate the most frequently measured outcome. “% improved” represents the proportion of the corresponding outcome measure that showed improvement.*

| **DIAGNOSIS** | **ASD** | | **MIXED** | |
| --- | --- | --- | --- | --- |
|  | *n (%)* | *% Improved* | *n (%)* | *% Improved* |
| **COGNITIVE** | 193 (22%) | 131 (68%) | 29 (15%) | 20 (69%) |
| **LANGUAGE** | *221 (25%)* | 150 (68%) | 43 (22%) | 25 (58%) |
| **SOCIAL COMMUNICATION** | 188 (22%) | 118 (63%) | 34 (17%) | 21 (62%) |
| **PROBLEM BEHAVIOR** | 113 (13%) | 83 (73%) | *61 (31%)* | 43 (70%) |
| **ADAPTIVE BEHAVIOR** | 110 (13%) | 75 (68%) | 27 (14%) | 21 (78%) |
| **EMOTIONAL** | 14 (2%) | 13 (93%) | 2 (1%) | 1 (50%) |
| **AUTISM SYMPTOMS** | 29 (3%) | 19 (66%) | 3 (2%) | 3 (100%) |
| **Total** | 868 |  | 199 |  |

*Table S7. Outcome measure findings in the ABA impact group with respect to the follow-up condition, the measurement of mastery/criterion, and the assessment of generalization. For clarity, study records coded as retrospective (N = 2) were excluded from the presented table, which accounts for the small discrepancies in totals indicated with asterisks across the follow-up section of the table.*

|  | **COGNITIVE** | | | **LANGUAGE** | | | **SOCIAL/COMMUNICATION** | | | **PROBLEM BEHAVIOR** | | | **ADAPTIVE BEHAVIOR** | | | **EMOTIONAL** | | | **AUTISM SYMPTOMS** | | |
| --- | --- | --- | --- | --- | --- | --- | --- | --- | --- | --- | --- | --- | --- | --- | --- | --- | --- | --- | --- | --- | --- |
|  | No follow up | Follow Up | Total (n) | No follow up | Follow Up | Total (n) | No follow up | Follow Up | Total (n) | No follow up | Follow Up | Total (n) | No follow up | Follow Up | Total (n) | No follow up | Follow Up | Total (n) | No follow up | Follow Up | Total (n) |
| **Improved** | 45% | 54% | 152 | 51% | 49% | 176 | 41% | 59% | 139 | 53% | 47% | 126 | 32% | 67% | 96 | 36% | 64% | 14 | 0% | 100% | 22 |
| **Regressed** | 0% | 100% | 2 | 0% | 100% | 2 | 67% | 33% | 3 | 75% | 25% | 4 | 0% | 100% | 3 | N/A | N/A | 0 | N/A | N/A | 0 |
| **Mixed** | 54% | 46% | 67 | 54% | 46% | 83 | 46% | 53% | 79 | 73% | 27% | 40 | 35% | 65% | 34 | 50% | 50% | 2 | 0% | 100% | 6 |
| **No Change** | 0% | 100% | 2 | 25% | 75% | 4 | 100% | 0% | 1 | 50% | 50% | 4 | 0% | 100% | 4 | N/A | N/A | 0 | 0% | 100% | 4 |
| **Total** | 105 | 117 | 223* | 136 | 129 | 265 | 96 | 125 | 222* | 101 | 73 | 174 | 43 | 93 | 137* | 6 | 10 | 16 | 0 | 32 | 32 |
|  | No mastery/  criterion | Mastery/  criterion | Total (n) | No mastery/  criterion | Mastery/  criterion | Total (n) | No mastery/  criterion | Mastery/  criterion | Total (n) | No mastery/  criterion | Mastery/  criterion | Total (n) | No mastery/  criterion | Mastery/  criterion | Total (n) | No mastery/  criterion | Mastery/  criterion | Total (n) | No mastery/  criterion | Mastery/  criterion | Total (n) |
| **Improved** | 51% | 49% | 152 | 56% | 44% | 176 | 71% | 29% | 139 | 86% | 14% | 126 | 75% | 25% | 96 | 57% | 43% | 14 | 100% | 0% | 22 |
| **Regressed** | 100% | 0% | 2 | 100% | 0% | 2 | 100% | 0% | 3 | 100% | 0% | 4 | 100% | 0% | 3 | N/A | N/A | 0 | N/A | N/A | 0 |
| **Mixed** | 54% | 46% | 67 | 55% | 45% | 83 | 75% | 25% | 79 | 90% | 10% | 40 | 79% | 21% | 34 | 50% | 50% | 2 | 67% | 33% | 6 |
| **No Change** | 50% | 50% | 2 | 100% | 0% | 4 | 100% | 0% | 1 | 100% | 0% | 4 | 75% | 25% | 4 | N/A | N/A | 0 | 100% | 0% | 4 |
| **Total** | 117 | 106 | 223 | 150 | 115 | 265 | 161 | 61 | 222 | 152 | 22 | 174 | 105 | 32 | 137 | 9 | 7 | 16 | 30 | 2 | 32 |
|  | No generali-zation | Generali-zation | Total (n) | No generali-zation | Generali-zation | Total (n) | No generali-zation | Generali-zation | Total (n) | No general-zation | Generali-zation | Total (n) | No generali-zation | Generali-zation | Total (n) | No generali-zation | Generali-zation | Total (n) | No generali-zation | Generali-zation | Total (n) |
| **Improved** | 71% | 29% | 152 | 61% | 39% | 176 | 54% | 46% | 139 | 76% | 24% | 126 | 75% | 25% | 96 | 86% | 14% | 14 | 100% | 0% | 22 |
| **Regressed** | 100% | 0% | 2 | 100% | 0% | 2 | 100% | 0% | 3 | 100% | 0% | 4 | 100% | 0% | 3 | N/A | N/A | 0 | N/A | N/A | 0 |
| **Mixed** | 63% | 37% | 67 | 60% | 40% | 83 | 58% | 42% | 79 | 83% | 17% | 40 | 65% | 35% | 34 | 50% | 50% | 2 | 100% | 0% | 6 |
| **No Change** | 50% | 50% | 2 | 75% | 25% | 4 | 100% | 0% | 1 | 100% | 0% | 4 | 75% | 25% | 4 | N/A | N/A | 0 | 100% | 0% | 4 |
| **Total** | 153 | 70 | 223 | 163 | 102 | 265 | 125 | 97 | 222 | 137 | 37 | 174 | 100 | 37 | 137 | 13 | 3 | 16 | 32 | 0 | 32 |

*Table S8. Analysis of various technique categories amongst the measured outcomes in the Comparison of ABA Techniques group.*

| *n (%)* | | | | | | | |
| --- | --- | --- | --- | --- | --- | --- | --- |
|  | **COGNITIVE** | **LANGUAGE** | **SOCIAL/**  **COMMUNICATION** | **PROBLEM BEHAVIOR** | **ADAPTIVE BEHAVIOR** | **EMOTIONAL** | **AUTISM SYMPTOMS** |
| **Teaching** | 96 (52%) | 99 (64%) | 27 (47%) | 29 (38%) | 18 (39%) | 1 (25%) | 1 (10%) |
| **Stimulus Characteristics** | 18 (10%) | 22 (14%) | 1 (2%) | 9 (12%) | 5 (11%) | 0 (0%) | 0 (0%) |
| **Reinforcement** | 53 (29%) | 16 (10%) | 8 (14%) | 22 (29%) | 6 (13%) | 0 (0%) | 0 (0%) |
| **Subject/Setting Characteristics** | 7 (4%) | 7 (5%) | 10 (18%) | 2 (3%) | 4 (9%) | 1 (25%) | 7 (70%) |
| **Comparing ABA Interventions** | 11 (6%) | 10 (6%) | 11 (19%) | 15 (19%) | 13 (28%) | 2 (50%) | 2 (20%) |
| **Total** | 185 | 154 | 57 | 77 | 46 | 4 | 10 |

*Table S9. Between groups comparison. This table categorizes the various outcome measures into three sections. The first section displays type of change in that occurred in each outcome. The second section displays which intervention improved. The third section categorizes the intervention that was compared to section two.*

|  | **Cognitive** | **Language** | **Social/**  **Communication** | **Problem Behavior** | **Adaptive Behavior** | **Emotional** | **Autism Symptoms** |
| --- | --- | --- | --- | --- | --- | --- | --- |
| **Comparisons** | ***n (%)*** | ***n (%)*** | ***n (%)*** | ***n (%)*** | ***n (%)*** | ***n (%)*** | ***n (%)*** |
| **Improvement** | 33 (85%) | 28 (82%) | 33 (73%) | 9 (50%) | 25 (63%) | 1 (33%) | 10 (53%) |
| **Mixed** | 3 (8%) | 3 (9%) | 8 (18%) | 5 (28%) | 10 (25%) | 0 (0%) | 3 (16%) |
| **No Difference** | 3 (8%) | 3 (9%) | 4 (9%) | 4 (22%) | 1 (3%) | 2 (67%) | 6 (32%) |
| **Regression** | 0 (0%) | 0 (0%) | 0 (0%) | 0 (0%) | 4 (10%) | 0 (0%) | 0 (0%) |
| **Total** | 39 | 34 | 45 | 18 | 40 | 3 | 19 |
| **Improved Intervention** | **n (%)** | **n (%)** | **n (%)** | **n (%)** | **n (%)** | **n (%)** | **n (%)** |
| **ABA** | 14 (42%) | 10 (36%) | 12 (36%) | 6 (67%) | 10 (36%) | 1 (100%) | 4 (40%) |
| **EIBI** | 7 (21%) | 6 (21%) | 7 (21%) | 0 (0%) | 7 (25%) | 0 (0%) | 0 (%) |
| **I-ABA** | 10 (30%) | 8 (29%) | 6 (18%) | 0 (0%) | 6 (21%) | 0 (0%) | 3 (30%) |
| **TAU** | 0 (0%) | 1 (4%) | 4 (12%) | 2 (22%) | 0 (0%) | 0 (0%) | 2 (20%) |
| **Nursery** | 0 (0%) | 0 (0%) | 0 (0%) | 0 (0%) | 1 (4%) | 0 (0%) | 1 (10%) |
| **Portage** | 0 (0%) | 0 (0%) | 0 (0%) | 0 (0%) | 0 (0%) | 0 (0%) | 0 (0%) |
| **DIR** | 0 (0%) | 0 (0%) | 0 (0%) | 1 (11%) | 0 (0%) | 0 (0%) | 0 (0%) |
| **Other** | 2 (6%) | 3 (11%) | 4 (12%) | 0 (0%) | 4 (14%) | 0 (0%) | 0 (0%) |
| **Total** | 33 | 28 | 33 | 9 | 28 | 1 | 10 |
| **Compared Intervention** | **n (%)** | **n (%)** | **n (%)** | **n (%)** | **n (%)** | **n (%)** | **n (%)** |
| **ABA** | 1 (3%) | 4 (14%) | 5 (15%) | 2 (22%) | 3 (11%) | 0 (0%) | 1 (10%) |
| **EIBI** | 0 (%) | 0 (0%) | 1 (3%) | 1 (11%) | 0 (0%) | 0 (0%) | 1 (10%) |
| **I-ABA** | 2 (6%) | 1 (4%) | 3 (9%) | 0 (0%) | 2 (7%) | 0 (0%) | 1 (10%) |
| **TAU** | 22 (67%) | 15 (54%) | 20 (61%) | 5 (56%) | 19 (68%) | 1 (100%) | 5(50%) |
| **Nursery** | 2 (6%) | 1 (4%) | 0 (%) | 0 (0%) | 0 (%) | 0 (0%) | 0 (0%) |
| **Portage** | 2 (6%) | 1 (4%) | 0 (%) | 0 (0%) | 2 (7%) | 0 (0%) | 1 (10%) |
| **DIR** | 0 (0%) | 1 (4%) | 0 (%) | 0 (0%) | 0 (0%) | 0 (0%) | 0 (0%) |
| **Other** | 4 (12%) | 5 (18%) | 4 (12%) | 1 (11%) | 2(7%) | 0 (0%) | 1 (10 %) |
| **Total** | 33 | 28 | 33 | 9 | 28 | 1 | 10 |
